# Coexistence of *bla*_OXA-48_ and Truncated *bla*_NDM-1_ on Different Plasmids in a *Klebsiella pneumoniae* Isolate in China

**DOI:** 10.3389/fmicb.2017.00133

**Published:** 2017-02-02

**Authors:** Lianyan Xie, Yi Dou, Kaixin Zhou, Yue Chen, Lizhong Han, Xiaokui Guo, Jingyong Sun

**Affiliations:** ^1^Department of Clinical Microbiology, Ruijin Hospital, Shanghai Jiaotong University School of MedicineShanghai, China; ^2^Department of Burns and Plastic Surgery, Ruijin Hospital, Shanghai Jiaotong University School of MedicineShanghai, China; ^3^Department of Medical Microbiology and Parasitology, Institutes of Medical Sciences, Shanghai Jiaotong University School of MedicineShanghai, China

**Keywords:** carbapenemase, OXA-48, NDM-1, coexistence, plasmid

## Abstract

**Objectives:** To describe the genetic environment, transferability, and antibiotic susceptibility of one clinical *Klebsiella pneumoniae* isolate harboring both *bla*_OXA-48_ and *bla*_NDM-1_ on different plasmids from a Chinese hospital.

**Methods:** The isolate was subjected to antimicrobial susceptibility testing and multilocus sequence typing using Etest and PCR. The plasmids harboring *bla*_OXA-48_ and *bla*_NDM-1_ were analyzed through conjugation experiments, S1-nuclease pulsed-field gel electrophoresis, and hybridization with specific probes. Plasmid DNA was sequenced using Pacbio RS II and annotated using RAST.

**Results:** *K. pneumoniae* RJ119, carrying both *bla*_OXA-48_ and *bla*_NDM-1_, was resistant to almost all carbapenems, cephalosporins, fluoroquinolone, and aminoglycosides and belonged to ST307. *bla*_OXA-48_ was located on a 61,748-bp IncL/M conjugative plasmid, which displayed overall nucleotide identity (99%) to pKPN-E1-Nr.7. *bla*_NDM-1_ was located on a 335,317-bp conjugative plasmid, which was a fusion of a *bla*_NDM-1_-harboring InA/C plasmid pNDM-US (140,825 bp, 99% identity) and an IncFIB plasmid pKPN-c22 (178,563 bp, 99% identity). The transconjugant RJ119-1 harboring *bla*_NDM-1_ was susceptible to carbapenem, and there was an insertion of IS*10* into the *bla*_NDM-1_ gene.

**Conclusion:** This is the first report of the coexistence of *bla*_OXA-48_ and *bla*_NDM-1_ in one *K. pneumoniae* clinical isolate in China. OXA-48 in RJ119 contributed to the majority to its high resistance to carbapenems, whereas NDM-1 remained unexpressed, most likely due to the insertion of IS*10*. Our results provide new insight for the relationship between genetic diagnosis and clinical treatment. They also indicate that increased surveillance of *bla*_OXA-48_ is urgently needed in China.

## Introduction

The emergence of carbapenem-resistant *Enterobacteriaceae* (CRE) has become a challenge to clinical therapy because of the rapid worldwide dissemination of multidrug resistance. The most important carbapenemase genes in *Enterobacteriaceae* are those encoding for KPC, VIM, IMP, NDM, and OXA-48 (Queenan and Bush, 2007). NDM-1 was first reported in 2008 as being produced by a *Klebsiella pneumoniae* isolate from a Swedish patient who had returned from India (Yong et al., 2009). Alarmingly, it was often found on large conjugative plasmids along with additional antibiotic resistance determinants (Hudson et al., 2014). NDM-1-producing *K. pneumoniae* isolates are considered as endemic in many countries including India, Pakistan, Bangladesh (Nordmann and Poirel, 2014) and show sporadic spread in the USA (Rasheed et al., 2013), Canada (Lowe et al., 2013), Colombia (Ocampo et al., 2016), Greece (Spyropoulou et al., 2016), Singapore (Ling et al., 2015), and China (Qu et al., 2015). OXA-48 was first identified in a *K. pneumoniae* isolate from Turkey in 2001 (Poirel et al., 2003). Since then, endemic spread of OXA-48-producing *K. pneumoniae* isolates has been reported in countries such as Turkey, Morocco, Libya, Egypt, Tunisia, and India (Nordmann and Poirel, 2014). The coexistence of genes for at least two classes of carbapenemases in *K. pneumoniae* also has been reported worldwide, including KPC-3 and VIM-2 in Italy, KPC-2 and VIM-24 in Colombia, NDM-1 and KPC-2 in Brazil, and NDM-1 and OXA-181 in Singapore (Lee et al., 2016).

We studied the genetic environment, transferability, and antibiotic susceptibility of a clinical isolate of *K. pneumoniae* harboring the *bla*_OXA-48_ and *bla*_NDM-1_ genes on different plasmids, which was isolated from a Chinese hospital.

## Materials and Methods

### Bacterial Isolates and Antimicrobial Susceptibility Testing

*Klebsiella pneumoniae* RJ119, carrying both the *bla*_OXA-48_ and *bla*_NDM-1_ genes, was isolated from the wound of a female burn patient at Ruijin Hospital, Shanghai, in September 2015. The previous travel history of the patient was not recorded. The genes were detected by the Cepheid Xpert Carba-R assay (Cepheid, Sunnyvale, CA, USA), a qualitative diagnostic test that was designed for rapid detection and differentiation of the *bla*_KPC_, *bla*_NDM_, *bla*_V IM_, *bla*_OXA-48_, and *bla*_IMP-1_ genes. The minimum inhibitory concentrations (MICs) of ceftazidime, cefotaxime, ceftriaxone, cefepime, aztreonam, piperacillin/tazobactam, ertapenem, meropenem, imipenem, trimethoprim/sulfamethoxazole, ciprofloxacin, and amikacin in the different strains isolated from the patient were determined by using the Etest (bioMérieux, France), and the results were interpreted according to the guidelines of the Clinical and Laboratory Standards Institute [CLSI] (2014). The CLSI does not have interpretative criteria for tigecycline; therefore, European Committee on Antimicrobial Susceptibility Testing (EUCAST) breakpoints were used^1^. *Escherichia coli* ATCC 25922 was used as a quality control reference strain.

This study was approved by Ruijin Hospital Ethics Committee (Shanghai Jiao Tong University School of Medicine), and the Review Board exempted the requirement for informed consent because this retrospective study only focused on bacteria and did not affect the patients.

### Multilocus Sequence Typing (MLST)

Multilocus sequence typing (MLST) was performed on seven housekeeping genes according to the guidelines given in the *K. pneumoniae* MLST website^2^.

### Conjugation Experiments, S1-Nuclease Pulsed-Field Gel Electrophoresis (S1-PFGE), and Southern Hybridization

The transferability of *bla*_OXA-48_ and *bla*_NDM-1_ was assessed in broth culture using *E. coli* J53 Azr (sodium azide-resistant) as the recipient. Transconjugants were selected on MacConkey agar containing sodium azide (100 mg/L) and meropenem (0.5 mg/L) or ceftazidime (1 mg/L) and were confirmed to have *bla*_OXA-48_ and *bla*_NDM-1_ by PCR analysis. Plasmid DNA of the parental and transconjugant isolates was visualized by S1-PFGE (Barton et al., 1995) and then transferred to positively charged nylon membranes (Roche Applied Science, Penzberg, Germany). The membranes were hybridized with digoxigenin-labeled *bla*_OXA-48_- and *bla*_NDM-1_-specific probes, respectively.

### DNA Preparation and Sequencing

Plasmid DNA was extracted from the RJ119 strain using the QIAGEN Midi Kit (Qiagen, Hilden, Germany). DNA was sequenced using Pacbio RS II (Pacific Biosciences, Menlo Park, CA, USA). The reads were *de novo* assembled using HGAP 3.0 of SMRT^TM^ Pipe (**Supplementary Images 1**). Protein-coding genes were initially identified and annotated using RAST (Aziz et al., 2008). Insertion elements (IS) and antimicrobial resistance genes were identified using IS Finder^3^ and ResFinder^4^, respectively. PlasmidFinder^5^ was used to detect and type the plasmid. BLAST^6^ searches were used to identify related plasmids carrying *bla*_OXA-48_ and *bla*_NDM-1_ to guide PCR-based gap closure and Sanger sequencing to assemble contigs into complete plasmids.

### Verification of the Fusion Region

To confirm the fusion region, four overlapping PCR were employed with in-house-designed primers P1 (5′-AAATGCGGCTAAGGTTGTGA-3′), P2 (5′-CCCTTCAGGCGTGATTCATA-3′), P3 (5′-GGCAAAGGCGACAAGAAGGA-3′), P4 (5′-GACAGCAGGCTCAGAAGACG-3′), P5 (5′-AGTAGCGGAGCAGGAAGGAC-3′) and P6 (5′-GTTCGGAGATGGAGGGTCAA-3′), respectively (**Figure 3**).

### Nucleotide Sequence Accession Numbers

The complete sequence of plasmids pRJ119-NDM1 and pRJ119-OXA48 in *K. pneumoniae* RJ119 has been deposited in GenBank under the accession numbers KX636095 and KX636096, respectively (**Supplementary Datasheet File 1**).

## Results

### Antimicrobial Susceptibility Testing and MLST

*Klebsiella pneumoniae* RJ119 belonged to ST307, exhibiting resistance or intermediate resistance to cephalosporins, β-lactam/β-lactamase inhibitor combinations, carbapenems, fluoroquinolones, aminoglycosides, trimethoprim/sulfamet-hoxazole, and tigecycline, as shown in **Table 1**.

**Table 1:** Antibiotic susceptibilities of *Klebsiella pneumoniae* RJ119 and its transconjugants.

	Minimal inhibitory concentrations (μg/mL)								
	
Isolates	CAZ	CTX	TX	FEP	ATM	PTC	ETP	MEM	IPM	STX	CIP	AK	TGC
RJ119	64	>256	>256	6	32	>256	4	2	3	>32	>32	>256	0.75
RJ119-1	32	>256	>256	4	16	6	0.094	0.094	0.75	>32	1.0	>256	0.38
RJ119-2	0.5	1.5	0.25	0.25	0.047	48	1.5	0.5	4	0.047	0.008	0.50	0.19


*CAZ, ceftazidime; CTX, cefotaxime; TX, ceftriaxone; FEP, cefepime; ATM, aztreonam; PTC, piperacillin/tazobactam; ETP, ertapenem; MEM, meropenem; IPM, imipenem; STX, trimethoprim/sulfamethoxazole; CIP, ciprofloxacin; AK, amikacin; TGC, tigecycline.*

### Conjugation Experiments, S1-PFGE, and Southern Hybridization

**FIGURE 1:**
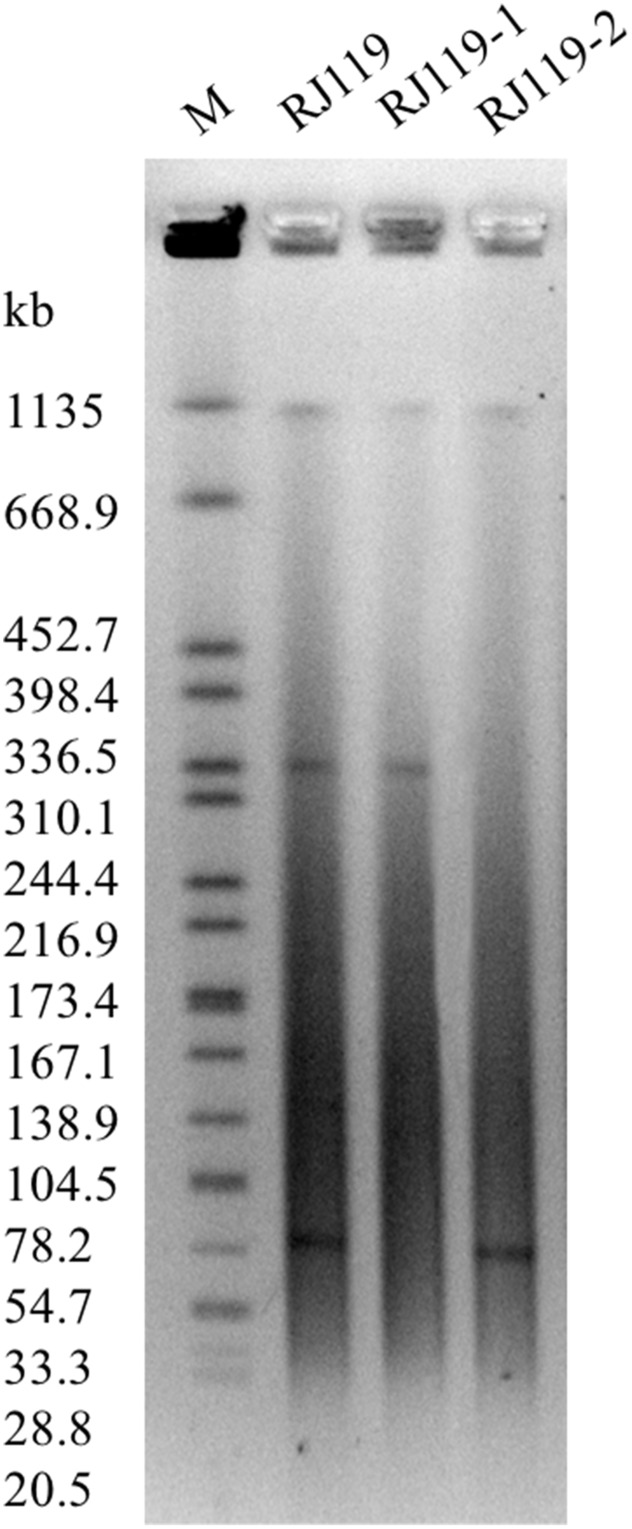
**S1-nuclease pulsed-field gel electrophoresis (S1-PFGE) profiles of *Klebsiella pneumoniae* RJ119 and its transconjugants RJ119-1, RJ119-2.** M, *Salmonella enterica* serotype Braenderup H9812 was digested with *Xba*I and used as a molecular size marker.

In the conjugation experiments, two phenotypically different transconjugants (RJ119-1 and RJ119-2), each of which carried one plasmid, were obtained. Phenotypic testing of the transconjugants showed that RJ119-1 was resistant to cephalosporins but susceptible to carbapenems, whereas RJ119-2 was susceptible to cephalosporins but resistant to carbapenems (**Table 1**). S1-PFGE revealed that RJ119 harbored two plasmids, and the transconjugants RJ119-1 and RJ119-2 each contained a single plasmid. Southern hybridization analysis revealed *bla*_NDM-1_ located on a 330-kb (pRJ119-NDM1) plasmid and *bla*_OXA-48_ on a 60-kb (pRJ119-OXA48) plasmid (**Figure 1**).

### Complete Nucleotide Sequence of Plasmids and Verification of the Fusion Region

**FIGURE 2:**
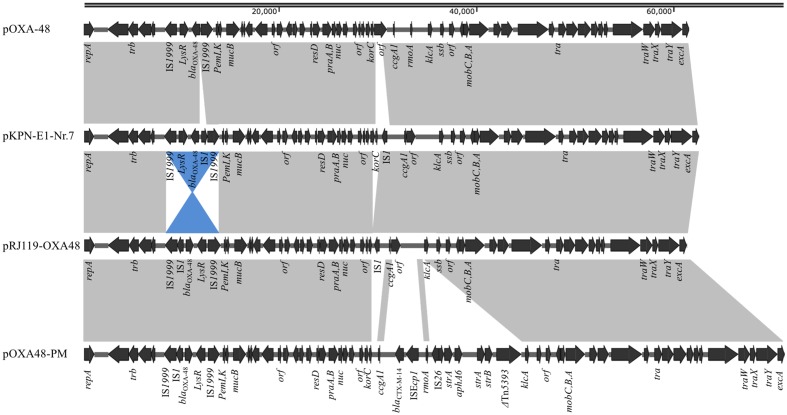
**Plasmid structures of *bla*_OXA-48_-harboring IncL/M plasmids pOXA-48 (JN626286), pKPN-E1-Nr.7 (KM406491), pRJ119-OXA48 (KX636096, this study), and pOXA48-PM (KP025948).** Gray shading indicates shared regions of homology, whereas blue shading indicates inversely displayed regions of homology. Open reading frames (ORFs) are indicated by arrows.

pRJ119-OXA48 is a 61,748-bp plasmid that belongs to the IncL/M incompatibility group (**Figure 2**). A BLAST search against all complete sequences indicated that pRJ119-OXA48 displayed overall nucleotide identity (99%) to pKPN-E1-Nr.7, a *bla*_OXA-48_-harboring IncL/M plasmid from a *K. pneumoniae* isolate from Switzerland (Carattoli et al., 2015). The main difference between the plasmids pRJ119-OXA48 and pKPN-E1-Nr.7 was that the Tn*1999.2* element (IS*1999-LysR*- *bla*_OXA-48_-IS*1*-IS*1999*) harboring *bla*_OXA-48_ in pRJ119-OXA48 was in the opposite orientation in pKPN-E1-Nr.7, and there was a deletion of *korC* (transcriptional repressor) in pR119-OXA48 (**Figure 2**), which was confirmed by PCR and Sanger sequencing. No resistance genes other than *bla*_OXA-48_ were identified in pR119-OXA48.

**FIGURE 3:**
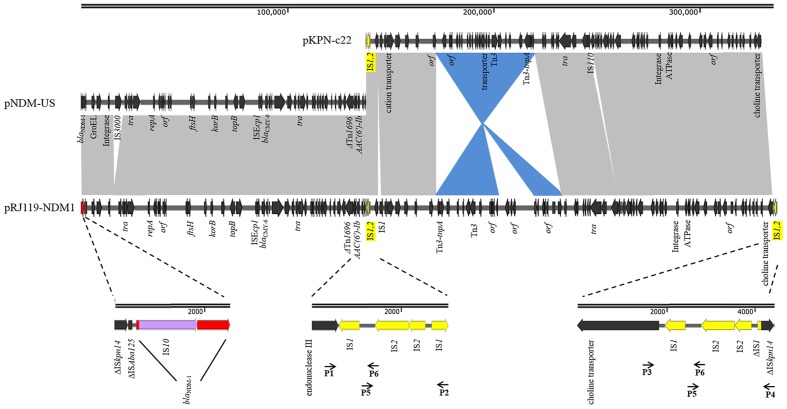
**Comparisons of pRJ119-NDM1 (KX636095, this study), pNDM-US (CP006661), and pKPN-c22 (CP009879).** Gray shading indicates shared regions of homology, whereas blue shading indicates inversely displayed regions of homology. ORFs are indicated by arrows. The IS*1*-IS*2*-ΔIS*1* structure is indicated by yellow arrows, and four overlapping PCR amplicons are shown by lines with primer names. *bla*_NDM-1_ is indicated by red arrows, and IS*10* is indicated by purple arrows in the amplification of the *bla*_NDM-1_ region.

pRJ119-NDM1 had a length of 335,317 bp and displayed query coverage (44%) and overall nucleotide identity (99%) to pNDM-US, as well as query coverage (55%) and overall nucleotide identity (99%) to pKPN-c22, according to the BLAST search (Conlan et al., 2014; Hudson et al., 2014) (**Figure 3**). These two different plasmids had two identical IS*1*-IS*2*-ΔIS*1* structures (2,381-bp). The first half of pRJ119-NDM1 was a close variant of the previously characterized pNDM-US, without the insertion sequence IS*3000*, and it harbored a variety of antimicrobial resistance genes including *aac(6’)Ib-cr*, *bla*_NDM_, and *bla*_CMY -6_, *sul1*. Among them, the *bla*_NDM-1_ gene cluster was arranged sequentially as IS*Aba14*, IS*Aba125*, *bla*_NDM-1_, *ble*_MBL_, *trpF*, and *tat*. However, it is noteworthy that the *bla*_NDM-1_ region found in pRJ119-NDM1 was different from that of pNDM-US; there was an insertion (IS*10*) in the *bla*_NDM-1_ gene. The second part of the pRJ119-NDM1 plasmid showed 99% identity to pKPN-c22 and harbored *aac(6’)Ib-cr*, *aac(3)-IIa*, *strA*, *strB, bla*_OXA-1_,*bla*_CTX-M-15_, *bla*_TEM-1B_, *QnrB66*, *catB3*, *sul2*, *tet(A)*, and *dfrA14*.

## Discussion

Carbapenemase genes often spread worldwide through clonal expansion in several pathogenic strains (Lee et al., 2016). Over 50% of NDM-producing *K. pneumoniae* isolates from India belong to either ST11 or ST147 (Lascols et al., 2013), and ST11 has also been associated with OXA-48-like enzymes from isolates found in Argentina, Turkey, and Spain (Oteo et al., 2013). The *K. pneumoniae* RJ119 isolate in our study belonged to ST307, which is not the part of the major clonal complex (**Supplementary Images 2**) and most prevalent in Pakistan and Korea (Habeeb et al., 2013; Park et al., 2015).

Thus far, OXA-48, in contrast to KPC-2 or NDM-1, has been rarely reported in China. The molecular epidemiology of OXA-48 in European and North Africa showed that in 92.5% of isolates, the *bla*_OXA-48_ was located on a self-conjugative IncL/M type plasmid (Potron et al., 2013). In our study, pRJ119-OXA48 showed high identity with the scaffold of other previously sequenced plasmids carrying *bla*_OXA-48_. However, we found a few differences, each discernible as a distinct DNA mobility event, between pRJ119-OXA48 and other *bla*_OXA-48_-harboring plasmids, including pOXA-48 (Poirel et al., 2012), pKPN-E1-Nr.7 (Carattoli et al., 2015), and pOXA48-PM (Chen et al., 2015) (**Figure 2**). Three interesting observations were made: (i) there were two IS*1*s in pKPN-E1-Nr.7, which were not present in pOXA-48; (ii) Tn*1999.2* in pKPN-E1-Nr.7 was only the inversion of the sequence in pRJ119-OXA48, and the *korC* gene was not present in pRJ119-OXA48; and (iii) the sequence of Tn*1999.2* was homologous to that in pOXA48-PM. Therefore, we speculated that pRJ119-OXA48 was an existence form in the evolution of *bla*_OXA-48_. Recent studies have reported the emergence of OXA-48-producing *K. pneumoniae* isolates and nosocomial outbreak of infections in Taiwan and Beijing, respectively (Ma et al., 2015; Guo et al., 2016). *bla*_OXA-48_ found in Taiwan was located on a 160-kb IncA/C plasmid, which was identical to the pKP_OXA_-48N1 plasmid found in France (Berger et al., 2013). *bla*_OXA-48_ found in Beijing was located on the IncL/M plasmid. These data from epidemiological investigations, coupled with those of our current report, show that the *bla*_OXA-48_ genes found in mainland China and Taiwan are from two different genetic backgrounds, and emphasize the need for increased surveillance of *bla*_OXA-48_ in China.

In China, *bla*_NDM-1_ was first identified in *Acinetobacter baumannii*. Subsequently, more *bla*_NDM-1_ genes were discovered in Enterobacteriaceae, especially in *K. pneumoniae*, and the gene was found carried on plasmids of various sizes (Qin et al., 2014; Qu et al., 2015; Zhang et al., 2015). In our study, *bla*_NDM-1_ was located on a 335-kb large plasmid. Based on the BLAST results, we suspected that it might be the fusion of a *bla*_NDM-1_-harboring InA/C plasmid, pNDM-US, and an IncFIB plasmid, pKPN-c22 (**Figure 3**). Previous studies have suggested that IncX3-type plasmids contributed to the dissemination of *bla*_NDM-1_ in China (Ho et al., 2012; Qu et al., 2015; Yang et al., 2015). However, in our study, the first half of pRJ119-NDM1 (contain *bla*_NDM-1_) was found belonging to the IncA/C group and identical to pNDM-US, the second part of pRJ119-NDM1 showed 99% identity to pKPN-c22, a plasmid detected in the USA. We speculated that the pRJ119-NDM1 plasmid might have been introduced from America after the fusion. Moreover, IS*1*-IS*2*-ΔIS*1*, the repeat sequence joint, might play an important role in the fusion of the two plasmids. The fusion of these two plasmids, each harboring an extraordinary number of resistance genes, might enhance antibiotic resistance. However, notably, the transconjugant RJ119-1, harboring truncated *bla*_NDM-1_, was susceptible to carbapenem, which might have been due to the insertion of IS*10* into the gene. Molecular-based diagnostic methods such as PCR, microarrays, and sequence-based diagnostics are now used in clinical applications due to their celerity and accuracy and are beginning to permeate clinical diagnostic laboratories in many countries (Okeke et al., 2011). However, in the case of our present study (unexpressed *bla*_NDM-1_ due to the insertion of IS*10*), because the PCR results did not match with the actual clinical situation, they may mislead clinical treatment.

## Conclusion

This is the first report on the coexistence of *bla*_OXA-48_ and *bla*_NDM-1_ in one *K. pneumoniae* clinical isolate in China. The production of OXA-48 in RJ119 contributed to the majority to its high resistance to carbapenems, whereas NDM-1 remained unexpressed, most likely due to the insertion of IS*10*. This study provides insight on the relationship between genetic diagnosis and clinical treatment in cases similar to the one in this study. Our findings also indicate the possibility of further spread of *bla*_OXA-48_ in China and emphasize the need for intensive surveillance and precautions.

## Author Contributions

JS and YD designed experiments; LX and KZ carried out experiments; JS, LH, and XG analyzed experimental results; YC and YD analyzed sequencing data; LX and JS wrote the manuscript.

## Conflict of Interest Statement

The authors declare that the research was conducted in the absence of any commercial or financial relationships that could be construed as a potential conflict of interest.

## Footnotes

1 http://www.eucast.org/clinical_breakpoints

2 http://www.pasteur.fr/-recherche/genopole/PF8/mlst/Kpneumoniae.html

3 https://www-is.biotoul.fr/

4 https://cge.cbs.dtu.dk/services/ResFinder/

5 https://cge.cbs.dtu.dk/services/PlasmidFinder/

6 http://blast.ncbi.nlm.nih.gov/Blast.cgi
