# Supplementary material for: Coexistence of blaOXA-48 and Truncated blaNDM-1 on Different Plasmids in a Klebsiella pneumoniae Isolate in China
Source: Front Microbiol. 2017 Feb 2;8:133. doi: 10.3389/fmicb.2017.00133 (PMC5288367; doi:10.3389/fmicb.2017.00133)
Supplement: Supplementary file 1 [file Data_Sheet_1.DOCX]

From: [gb-admin@ncbi.nlm.nih.gov](mailto:gb-admin@ncbi.nlm.nih.gov)

Date: 2016-08-01 22:55:47

To: [13671578899@126.com](mailto:13671578899@126.com),[xielianyansjtu@126.com](mailto:xielianyansjtu@126.com)

Subject: GenBank KX636095-KX636096

Dear GenBank Submitter:

Thank you for your direct submission of sequence data to GenBank. We

have provided GenBank accession numbers for your nucleotide sequences:

RJ119-1 C119-1 KX636095

RJ119-2 C119-2 KX636096

The GenBank accession numbers should appear in any publication that

reports or discusses these data, as it gives the community a unique label

with which they may retrieve your data from our on-line servers. You may

prepare and submit your manuscript before your accessions are released in

GenBank.

Submissions are not automatically deposited into GenBank after being

accessioned. Each sequence record is individually examined and processed

by the GenBank annotation staff to ensure that it is free of errors or

problems.

You have not requested a specific release date for your sequence data.

Therefore, your record(s) will be released to the public database once

they are processed. If this is not what you intended, please contact

us as soon as possible with the correct release date.

Since the flatfile record is a display format only and is not an editable

format of the data, do not make changes directly to a flatfile. For

complete information about different methods to update a sequence record,

see: http://www.ncbi.nlm.nih.gov/Genbank/update.html

Any inquiries about your submission should be sent to [gb-admin@ncbi.nlm.nih.gov](mailto:gb-admin@ncbi.nlm.nih.gov)

For more information about the submission process or the available

submission tools, please contact GenBank User Support at

[info@ncbi.nlm.nih.gov](mailto:info@ncbi.nlm.nih.gov).

Please reply using the original subject line.

This will allow for faster processing of your correspondence.

Sincerely,

Mark A. Landree, PhD

Contractor

The GenBank Direct Submission Staff

Bethesda, Maryland USA

*******************************************************************

[gb-admin@ncbi.nlm.nih.gov](mailto:gb-admin@ncbi.nlm.nih.gov) (for updates/replies to GenBank entries)

[info@ncbi.nlm.nih.gov](mailto:info@ncbi.nlm.nih.gov) (for general questions regarding GenBank)

www.ncbi.nlm.nih.gov/books/NBK51157/ GenBank Submissions Handbook

LOCUS pRJ119-NDM1 335317 bp DNA linear 18-SEP-2016

DEFINITION Klebsiella pneumonia strain RJ119 plasmid pRJ119-NDM1.complete

sequence.

ACCESSION

VERSION

KEYWORDS .

SOURCE Klebsiella pneumonia

ORGANISM Klebsiella pneumonia

Unclassified.

REFERENCE 1 (bases 1 to 335317)

AUTHORS xie,l. and sun,j.

TITLE Coexistence of blaOXA-48 and truncated-blaNDM-1 on different

plasmids in a Klebsiella pneumonia isolate in China

JOURNAL Unpublished

REFERENCE 2 (bases 1 to 335317)

AUTHORS xie,l. and sun,j.

TITLE Direct Submission

JOURNAL Submitted (18-SEP-2016) Department of Clinical Microbiology, Ruijin

Hospital, Shanghai Jiaotong University School of Medicine,

Department of Clinical Microbiology, Ruijin Hospital, Shanghai

Jiaotong University School of Medicine, 197 Ruijin 2nd Rd,

Shanghai, Shanghai 200025, China

COMMENT ##Assembly-Data-START##

Assembly Method :: SMRT v. HGAP3.0

Sequencing Technology :: PacBio

##Assembly-Data-END##

FEATURES Location/Qualifiers

source 1..335317

/organism="Klebsiella pneumonia"

/mol_type="genomic DNA"

/plasmid="pRJ119-NDM1"

CDS 38..304

/codon_start=1

/product="Mobile element protein"

/translation="MATLERLLSLLSVFDVVVWMTDGWPMYESRLKGKLHVISKRYTQ

RIERHNLNLRQHLARLGRKSLSFSKSVELHDKVIGHYLNIKHYQ"

CDS complement(328..444)

/codon_start=1

/product="Microsomal dipeptidase"

/translation="MILIWFTVAKIWGEFKHEVRQFQKPYDNQQAEQISLMV"

CDS 499..612

/codon_start=1

/product="hypothetical protein"

/translation="MMELPNIMHPVAKLSTALAAALMLSADESPNDFYQNH"

CDS 682..1890

/codon_start=1

/product="Mobile element protein"

/translation="MCELDILHDSLYQFCPELHLKRLNSLTLACHALLDCKTLTLTEL

GRNLPTKARTKHNIKRIDRLLGNRHLHKERLAVYRWHASFICSGNTMPIVLVDWSDIR

EQKRLMVLRASVALHGRSVTLYEKAFPLSEQCSKKAHDQFLADLASILPSNTTPLIVS

DAGFKVPWYKSVEKLGWYWLSRVRGKVQYADLGAENWKPISNLHDMSSSHSKTLGYKR

LTKSNPISCQILLYKSRSKGRKNQRSTRTHCHHPSPKIYSASAKEPWILATNLPVEIR

TPKQLVNIYSKRMQIEETFRDLKSPAYGLGLRHSRTSSSERFDIMLLIALMLQLTCWL

AGVHAQKQGWDKHFQANTVRNRNVLSTVRLGMEVLRHSGYTITREDLLVAATLLAQNL

FTHGYALGKL"

CDS 2656..3021

/codon_start=1

/product="bleomycin resistance protein"

/translation="MADHVTPNLPSRDFDVTEAFYAKLGFATSWKDRGWMILQRGGLQ

LEFFPYPDLDPATSSFGCCLRLDDLDAMVALVNAAGAEEKSTGWPRFKAPQLEASGLR

IGYLIDPDCTLVRLIQNPD"

CDS 3026..3664

/codon_start=1

/product="Phosphoribosylanthranilate isomerase"

/translation="MPAKIKICGISTPEALDATIAARADYAGLVFYPASPRAVTSNVA

GALTSRAAGQIAMVGLFVDADDAVIADALVAAKLNALQLHGSESPERVAQLRARFGKP

VWKALPVASASDVARAAAYAGAADLILFDAKTPKGALPGGMGLAFDWSLLAGYRGALP

WGLAGGLNPTNVAEAIARTGAPLVDTSSGVESAPGVKDTDKITNFAFAVRLA"

CDS complement(3675..4706)

/codon_start=1

/product="Cytochrome c-type biogenesis protein DsbD,

protein-disulfide reductase"

/translation="MTASTIRLRRWLAGLALLLALPATSAVAQDFELPPVDEVFVLSA

QATAPDRIEVRWRIADGYYLYRHRTSVKADAAFTGATMALPKGKAYRDEFFGDVETYR

KELLGTLTGTPAAGASATTLTVKYQGCADAGVCYPPQTRTLKVALPGEAGAGGFGCKA

RGLHDYVVKNGSADHPNAEVKFALGDVVNTMIGCTNGETIMLCHDTSLPRPYSLGFRV

QGTEGLWMDVNKSIYLEGKSPQPHRWEPAEGWFAKYDHPLWKRYADLAAGAGHGGMDW

FVIHAFVEALKAKAPMPIDIYDALAWSAITPLSEQSIAEGNRTLDFPDFTRGQWRTRK

PIFALNDAY"

CDS complement(4711..5040)

/codon_start=1

/product="Periplasmic divalent cation tolerance protein

CutA"

/translation="MPVSALICFCTCPDADSAERIATALVAERLAACVNLLPGLRSVY

RWQRKVEAAAEVLLLVKTSAEAYPALQERLRQLHPYELPELLAVEAASGLPEYLQWLA

AESRPVN"

CDS 5234..5524

/codon_start=1

/product="Heat shock protein 60 family co-chaperone GroES"

/translation="MSNIKPLHDRVVIKRMEEEKLSAGGIVIPDSATEKPIKGEVVAV

GTGKVLDNGQVRAPQVKVGDKVLFGKYSGTEVKLDGVELLVVKEDDLFAILG"

CDS 5580..7220

/codon_start=1

/product="Heat shock protein 60 family chaperone GroEL"

/translation="MAAKDIRFGEDARSKMVRGVNVLANAVKATLGPKGRNVVLQKSY

GAPTITKDGVSVAKEIELADAFENMGAQMVKEVASKTSDNAGDGTTTATVLAQAFIRE

GMKAVAAGMNPMDLKRGIDQAVKAAVGELKSLSKPSSTSKEIAQVGAISANSDANIGD

LIAQAMDKVGKEGVITVEEGSGLDNELDVVEGMQFDRGYLSPYFVNNQQSMSADLDDP

FILLYDKKISNVRDLLPVLEGVAKAGKPLLIVAEEVEGEALATLVVNTIRGIVKVCAV

KAPGFGDRRKAMLEDMAILTGGVVISEEVGLSLEKATIKDLGRAKKIQVSKENTTIID

GAGEGAGIEARIKQIKAQIEETSSDYDREKLQERVAKLAGGVAVIKVGAATEVEMKEK

KARVEDALHATRAAVEEGIVPGGGVALIRAKAAIAGIKGVNEDQNHGIQIALRAMEAP

LREIVTNAGDEPSVILNRVVEGSGAFGYNAANGEFGDMIEFGILDPTKVTRTALQNAA

SIAGLMITTEAMVAEAPKKDEPAMPAGGGMGGMGGMDF"

CDS 7409..8014

/codon_start=1

/product="transposase InsA"

/translation="MPRLTAPRRQAEVCAPLPAAHAAARYARHLPERTLLYALVQAHY

PDFIARLEAEDRPLPEYVREEFETYLRCGVLEHGFLRVVCEHCRAERLVAYSCKKRGL

CPSCGARRMAESARHLVDEVFGPRPVRQWVLSFPYPLRFLFASKPEAIGPVLGIVHRV

IAGWLADQAGVPRDTAQCGVVTLIQRFGSALNLKCNPNPHL"

CDS 8079..10481

/codon_start=1

/product="Putative insecticidal toxin complex"

/translation="MGLLSSLTKPNGATVSYEYDAAHRLVAETDAQGNRRELELNDLG

NPVEERLLDALGQTRWIERRIFNEIGWLSSVSDAYSNQSSFSYDVVANLIQETSPSGN

THSYKYDGFHHRTQTTDPLGKVTQVLYKDTGDVYRVSDPRSRLTYYSYNGFGEVTQVR

SPDTGTTDITYDEAGNVATRKTAKGQTTSYSYDALNRIIETSSDVAGESPILYGYDEA

TSPYGIGRLTSVDDGNGVRRFGYTPEGWLAYETWETHGQSLTTQYQYDGAGLVTKITY

PSGREVSYTRDSAGDVIEVTTTQAGTTTNLASQIERAPFGPVTSMVRGNGISESRTLD

LDYRVTGIDAARVHSLVYRYTPDSLISAIDDNLSSSVNQSLGYDAVGRITSAEGIYGV

LGYGYDATGNRTSITTDGLSQSYTINYMNNWLVKAGQTSRSYDANGNLTKQGADTFTY

DSQNRLVAATVAGVTVSYTYNHLDQRVTKTLNGHTRLLVYDLAGNLIEELDAATGDVL

AEYIWLDGTPLGFVQSGQTYQVHVDHLGTPKALTDVSGQVVWKASYSPFGKASIIIQG

PTFNLRFPGQYYDAETGFHYNWRRYYDPATGRYITSDPLGLIDGVNTYGYVHGNPMSN

TDPTGEFAFVGAGIGAGLELLSQLIENNGSWKCVSWSKVGIAGAIGAIGGGWASGVFR

HASSGKSWFKLSQKWSNVSPRVRKVQGVPRGNELHHWAIQRNGKFGKYVPDSIKNHPW

NLKSIPRDIHQNIHGNGPTPYSAFGRWWHGTPEWAKVAQASPVSGGLADSINDEGCGC

AN"

CDS 10453..10893

/codon_start=1

/product="hypothetical protein"

/translation="MMRDAVVQIEFPALLVSSKKRSLFVVASESEFGKCTIQSLRNGY

FELMDIYDSEGRHYKIDEVASYKPLSPFWYWPVEIVMYGSRLFKANFNAVLISNLDCK

ELKSELCDLAKKYRSNLDSGVGIEKIMEEMESARTIKELIKVFG"

CDS 11172..11516

/codon_start=1

/product="hypothetical protein"

/translation="MNTMSPITAKYTIQYKQAVEQKCNTALSVEQLNSKAFTNVVQAM

VSSETVDRMGLDAAGGSLQDTLSVIGKNVTCSDLNAPFKALLDDKDFTRKHQHLSKVL

HTWNEVVSQSKP"

CDS complement(11532..12134)

/codon_start=1

/product="hypothetical protein"

/translation="MYKSLEDLMAGIYEMAAPDGIICNEVSKFLAANQVKPEDISSGI

WFFLWLKSAPKDAKPIQKEIPGFGVVLNMPTYGGTLNEAITGLIENECRQIELAEGHP

SLEAWVESVLAGRDDGEENEAAANIVDLLNGCHDLLDTAIINYLFKVGKINEQEILSL

IYYGELCDHSLVGTPFSTLHIPSENNVHERNIIPIKKGAS"

CDS 12404..12685

/codon_start=1

/product="hypothetical protein"

/translation="MIFDVRATFEVALQTDTHLVLIDLDQGASVTNDADAVIAWLAAN

LEGGIGKRKVYYRDTDGRFDELKVNAGAFAGFAPCSEGQQTTLAGMLGQ"

CDS 12984..13520

/codon_start=1

/product="hypothetical protein"

/translation="MLSKKVFFISQAEAERLEPVPGAAMISITDPDKSPAALGQWGQL

YRDSFYDGGYSENTIHTMKAAFRMNYASYIDSSQAEKLSTFLDGLVGSGIDQIFVHCY

YGESRSGAVALYLQNKHGFTPNKPITKPNRTVYELLCNPTKFEPLMQSYETQHMEEEL

PLHLKIWDFLLVAVGLRR"

CDS 13523..14533

/codon_start=1

/product="Mobile element protein"

/translation="MHDTNNDKEELVSHAKVNVPAEQSIRGELLPSSSSLRNIQDNPA

VAYLVSLGSKRSRQTMSSFLNIVAKMIGFQNLRDCAWSSMRRHHILAVLEMLGDAGKA

PATINTYLSALKGVALEAWTMKQIDTDSFQHIKQVRSVRGSRLPKGRALERHEIRSLF

FTCESDSSAKGLRDAAILGVLLGCGLRRSEIVALDMGSMIYKDRALKVLGKGNKERMA

YVPGGAWKRLDKWVEEVRGTHEGPLFPRIRRFDDVTGERMSDQAIYHILETRRVEAGL

EMFAPHDLRRTFASSMLDNGEDIVTVKDAMGHSSIATTQKYDRRGDERLKRASQRLDI

AD"

CDS 14538..15407

/codon_start=1

/product="DNA polymerase III"

/translation="MNDEELELARAEAMKADRCFSKGRLRDEFRMKPKPGVEPVSFYK

NGYGGQFGVYRIADCQPMRRRGCSPASQKQIRAQSILSVKARMRSNLAKASVMAQRWV

ALEPLVLDTETTGLGERDQVIELAVTDIRGAVLLCTRLRPTVEIDPQAMGVHGITETE

LSNEPTWTQVAPALARLLSGRHLVIFNSSFDSRMLRQTASAFGDQLSWWQEQNCLCAM

KLAADAFGSTNRHGTISLADATCEAGVSWKGRAHSAATDAIATADLVTEIAKVQRDLM

VQLQELQSKGNLE"

CDS 15404..15895

/codon_start=1

/product="hypothetical protein"

/translation="MTEQSYGESLKFFSDWQKDPAKRTGLNVQHTLTRGEYPTVSIEI

APIRASGSSPDWKSKITVQLTRGELTAFCSVLFGLRSKAEGSYHGDAKNKSFAVYNNG

KAGVAIILSERGNQLQNFINDDDRMELAVFAVRQLSNAWKVTPSDAIALLRQSAWMDR

NLS"

CDS 16341..17087

/codon_start=1

/product="DNA replication terminus site-binding protein"

/translation="MENEPVNKIVVTEQTGREALELAAHSYRDLHINPDYSQKSARRT

VGVLWFSPSRIGVADEIAATVERINAAKAGIEEFIISTYPTRQERFEALRADCPGVMT

LHLYRQIRCYTNGDIDSIRFTWQRKDSLKKPVKEELLQRIREELERSGPDYQLPLEQL

IQKIASTPEPYLRERREVKVQPVANVMAAGVLKTVTAPMPLIVLQDKDVQLKLLRNFD

ASEQRKTRSDKAASEILGTFGGVTIESFPG"

CDS 17240..18280

/codon_start=1

/product="hypothetical protein"

/translation="MARKLVEFDDVAAAAQKLKDAGKRPTVIAIRDIIGKGSFTTIST

YLKQWSEEHSLDEELVEVVLPESVMSDAELFLQKIYTVAKASADEQLERERELLRQKE

IEYQEDMQQAVDMANDATERAELLEEQLEALTNKKSELDAALGKAENSLSLKSAELER

SLADIDKLEKRIVELEGKLEAKAADLTRAQDHLEQAKSENRSLSQKLATTEGELEEQK

GKSIEQTEKLRAAQEQKTSLKEQLENVQAKLAQSQDSLATAKAHGESAERECQRLSGE

VEKLDAKLSSAEAEARALVQDKGMMAGQLQEKDQQAKSLEQRLNDALTKISGLEQELA

KAGKGGKKKEEN"

CDS 18280..18558

/codon_start=1

/product="hypothetical protein"

/translation="MARKAKYSEEWRHRAAALQTKIEEAMTLATSSIGDYRWLHRLHS

WVTEVAQGKAPDWWTDLDCEVSLPREEKRISTFLSTQKKRITLQMCLS"

CDS 18567..20078

/codon_start=1

/product="putative DNA helicase"

/translation="MKQLPPDTPEQSLITQYKGPRLVVKAYAGTGKTTTLVKYAHNNL

DSRILYLAYNRAIRDEAREKFPANVDCKTSHQLAYATIGRGYQHKLSGNLRLTDIAQA

VNTKNWTFAKDILDTLNAFMCSADMRILYTHFARADTGKVLTSKQERYQIQVVEGAEL

IWKRMTNVQDPFPTVHDCYLKQYQLGMPNLSRRYTTILFDEAQDANPVTSSIVLQQNC

KVILVGDRHQQIYRFRGANNALDSKELMNADQLYLTHSFRFGPNVSLVANALLELKGE

TRPVVGRGPADQVLMFLPGDVGHRAILHRTVMGVIETALSATESGAQVFWVGGIDAYQ

INELQDLYWFSMAEPDRVKNKKLLDEYEDYFEYQEVAKATKDPEMMRAVKIINSYDEI

PERLTTLRRNTVKEEFGADITVSTAHRCKGLEWDFVQLYDDFPDVLDPELDPMARDDE

INLLYVASTRAMRILALNSAVEMVIRYITQKRMVEKQMKMAAEATEVEEDTTK"

CDS 20189..21229

/codon_start=1

/product="IncF plasmid conjugative transfer pilus assembly

protein TraF"

/translation="MGKYVRETMKKSPLNLLLLAALTLGASHQAWAQDGTRPGFYERK

EEGWFWYKEEPKEPEKKPEKPKPKPVAEAKPTQPKPAAPLPSGPEMFSAEWFRENLPK

YKDLAWNNPTVENVRTFLYLQRFAIDRSEQFSDATELAVVGDPFLDEITRRPAATFAS

QQVDRDAGNAKNMLLKSVAERVGIFFFYKSDDDYSDLQAPLIKMLEQGEGFSIIPVSM

DGKPLPSGLFPHYKTDEGHAKQLGIVTFPAVYLASPDGQFAPIGQGPMSLPELNHRIL

VAAKRNGWVTDEEFNRTRPVLNLENNIAERLASPELGSDLKQLSQASGDKDNFVPPEQ

LMKYIRDKLQEN"

CDS 21231..22664

/codon_start=1

/product="IncF plasmid conjugative transfer pilus assembly

protein TraH"

/translation="MVTHKTLKRSLLALSVAASLVMAPTGAIAANGLQSQMDKLFNEM

SNTTPPGVYESQRRGVLAGGRFTAKTRIFDENLVSFAPPSWKAGCGGVDLFGGSLSFI

NADQIVQLLRAVAANAKGYAFQLALDNVFPDGAKWIENFQKKVQALNQHLGNSCQLAQ

GFVNDLTSGMDLKHKTDASITATTSGLYEDFFGSKQETSGKSPLEELKANKPDEYNKM

IGNIVWKQLKSNNANTWFQYGDNTLLEAIMSLTGTVIIGDLVNDPNSTGTGAKTTPLT

TLPGNKITLSDLISGGSVEIYSCDSDTTNCLSAGSSNKTVVLKGIKNQITDMLLGTSS

TPGVIYKYATNSGTLTDPEKAFVSNLPGGIGTIVRNLSVLSQDGANLFATESSGAIAL

TMMYSFSEEFFRAARIAMANSKSPYKKEALELLAQSQQQIRAEYTILSSQYGDLASQI

EKYNNLLDNIRKQKYMLATLSNPPSTN"

CDS 22677..26291

/codon_start=1

/product="IncF plasmid conjugative transfer protein TraG"

/translation="MGSFSIHSIGDSAFLEQILIAVSMITGTGDFEKMVSIGLLLGVL

MICIQSVFQGAKQINLQQVLVGWILYACFFGPTTTVTIEDAYTGQVRVVANVPIGVGF

AGGVISNVGYTITNLFETGYGVIVPNVTESHFSETLKLLNDVRRRAYDTGVFTALNSA

NGGGYVDVRRSWNNYIRECTLTKVDLNLMSLDELMNRSTDSALRFNSQLYGTRLYLST

ANPDGADYTCTDGWVAISTATANLSSPVVVDALNSLLGIDTSTGDNALTKLTDSLQAM

GATTTSSIDYLKAAVLEPLYYEAAAGRYQDLQDYGSALMVNQAIQQRNTQWAAEQSMF

MTVVRPMLTFFEGFIYAITPIIAFIIVMGSFGLQLAGKYVQTILWIQLWMPVLSIINL

FVHTAASNEMSSLSAGGLNSMYALSSTGDVLQHWIATGGMLAAATPVISLFIVTGSTY

AFTSLASRISGSDHVDEKMQTPDLLKQGPVMQSQPAYNHNQFSGAIANGAESMISTFS

LGSTLASGVSSAQALQSQKSEAFQSTLGRGFSDGVSQDQAYSRLSNVGRNVSSQNTAQ

SQLINQQAKNFMDKFQVDDSHSDAVKGAFAMQAMGTLDVDEAASMLMPMVGKARAAMK

AAAGVKSNSTALVPAGGNGESGGGSDVLDIKAQAKGATESSTQDSSSWSASDVSQFMK

GVSYSQTDSQALTNQLAQGFSRSGSESFKQTWGDSLSQNLSKSASELVSASDTFTTMS

QLQNQMGSMTNTDFKTLGGAVAQTPAAMNQLNDYFRNAAPQSVKDEAASLQQRYQAYG

MSPQVAQAAARMTAMTNSKNYEQGKELGGYQAALQAINTASGRNGAFSGDAYGNNGIE

GPNVQGLPGQVQGAVGSGPNIPTGFRENVAGMAGTNPASEAGQLPTNSPLVQNEHAAG

TSALHNQAQQTERNVSAPELKKAQDNLMNSLPEMSWSASAWGAWDNSSDWMGRRAEQA

GGALIAGGQAGADAFSRAMDQMRTMTPEQRDQFIAATQRGDQAVQEEFGWAGDAMVGM

AKLGRNVMGAAASGYDAAKEWLTGKSDLSEAAKGMSIEERGAFYAAALSSAAEAGGGA

AQQFMNQYGDEFKETMQSIAQSRYGLTESQAAVYAESFDTNEGRMNQAVQNLKMEYAE

RNPDGSPMMQGGQPVLSQQNEEFTDKLVNVLQNSTEAGDRSGSYLTAVRGYNIANQRF

"

CDS complement(26329..26691)

/codon_start=1

/product="hypothetical protein"

/translation="MKHVVNILLLGMVLLGIAMMADTPWGLGVALAPFGVWGARFLFL

VHKSLWAAVIFWGGIAYFQWQVALVVGALFGLTCFIRVARSAYKEAPPTRRRKKQTGG

FQDSYDFDQRFHIGAGDE"

CDS 27407..27679

/codon_start=1

/product="hypothetical protein"

/translation="MDKCQLIDIPSDPEKKREWIKYKLKIQGLSLAALGRKHKTSRQV

VSTALYKPSPRWEHEIATALGVKPSEIWPERYDEEHEIPLRHKEAS"

CDS 27679..28212

/codon_start=1

/product="hypothetical protein"

/translation="MKNKAKALVLSAALLSSTANAIDLSGTIFDKAAKAYNLDPLLVY

SVALAESASGRGNGSISPWPWTLRVPGLPFYAKSEDQAKAKLAEFQQQYGRAIDVGFM

QVSIRWNGHRVSSPADLLDPETNVMVGAEVLSEAIQSSPNDLELGVGRYHAWEDEIRA

RNYGSRVLAIYRNLRDL"

CDS 28223..28831

/codon_start=1

/product="hypothetical protein"

/translation="MLELDIIGAWDARAVNLDQEEADRNVYEFDLTLWNLLSTLAKER

PDDAASQFSLGMDTVQKLSLATPSQLEALASGVLISFKLETAEQNIITRLSGDYDPVV

FINHSVDEFDAAYWLLFNRVASRDPEMAKEVFGVSRELAELVAKATDSQLRHMSGTTV

THFTLRFAPSIIEEILDDSREELTHPVLKKLQQSLQGRGRWR"

CDS 28879..29379

/codon_start=1

/product="Transcriptional activator"

/translation="MALAGYITKIIMIETGLTYKQVRRLYQDLERDGYTLERKSRTFR

GGATLIHSHTSKIQASLLMQLYFNIGGEAVLRSVNIKALNKAFRMYHAIRKEVPGMKG

ARWAPFDITDAWCLASELRSGDAMLEVCDNCKCTYFTSVNQRTCVECPFCKEQGRHGG

GEKECA"

CDS complement(29439..29717)

/codon_start=1

/product="hypothetical protein"

/translation="MKGVLNELEEEYKKREEEEKEKRAQAERIMSDMESCGVDIGLLN

EMFTSRSEPDNAKYSKDGVSWSGQGRRPDAFKGLGAVELERYRIPQKK"

CDS complement(29860..30153)

/codon_start=1

/product="hypothetical protein"

/translation="MAGSKWDGKISNLNISEQAFPELYRELSQMQHKARSDRLRALAL

LGLYSLRFSGNIGSFEQQSSESQPAQHQNSPVQTDAKLNSQRDSLKGKLMGSV"

CDS complement(30170..31153)

/codon_start=1

/product="Rod shape determination protein"

/translation="MSQFVLGLDIGYSNLKMAMGYKGEEARTVVMPVGAGPLELMPQQ

LTGGAGTCIQVVIDGEKWVAGVEPDRLQGWERELHGDYPSTNPYKALFYAALLMSEQK

EIDVLVTGLPVSQYMDVERREALKARLEGEHQITPKRSVAVKSVVVVPQPAGAYMDVV

SSTKDEDLLEIIQGGKTVVIDPGFFSVDWVALEEGEVRYHSSGTSLKAMSVLLQETDR

LIQEDHGGAPGIEKIEKAIRAGKAEIFLYGEKVSIKDYFKKASTKVAQNALIPMRKSM

REDGMDADVVLLAGGGAEAYQDAAKELFPKSRIVLPNESVASNARGFWFCG"

CDS 31829..32191

/codon_start=1

/product="hypothetical protein"

/translation="MYWNAHKSAREEASEDEQGRVGTRVRILGVSLVAEWYRNRFVEQ

VPGQKKRVLSTHIKKGRGHAYSMSHFKKEPVWAQELIQQVETRYAVLRQRATALAKIR

RALNEYERQLNKTHSDEV"

CDS 32196..32483

/codon_start=1

/product="hypothetical protein"

/translation="MTASVAATELAKLGKCEAMIKKVASHPRPALSKRPQSPQGTDST

LRGEFAHFRYEAAALRFMSGTAGAKRRIYQLVFSATVAAGALTMLAAWTTS"

CDS 32468..33568

/codon_start=1

/product="plasmid replication protein RepA"

/translation="MDHQLESIDGTIMSKRTKDKDLEKLDVIKDSPQMSLFEIIESPA

KKDDYSNTIEIYDALPKYIWDQKREHEDLSNAVVTRQCTIRGQHFTVKVKPAIIEKDD

GRTVLIYAGQREEILEDALRKLAVNGKGHIIEGKAGVMFTLYELQKELSKMGHGYNLN

EIKEAIQVCRGATLECISDDGEAFISSSFFPMVGLTTRGEFRKKGGNARCYVQFNPLV

NESIMNLSFRQYNYKIGMQIRSPLARYIYKRMSHYWTQASPDSPYTPSLISFLTQSPR

ELSPRMPENVRAMKLALEALIKQEVISDYDANQIKDGRRVIDVRYVIRPHENFVKQVM

ASNKRKQQTELRAIKQGMIDHDIIDEPQRKGR"

CDS 33897..34082

/codon_start=1

/product="hypothetical protein"

/translation="MGDLHVMRFDMWVRCTKAGSEPLDIWELCTIDEAEQRLTDQDKP

KKAVKMTPIVHKVIVLT"

CDS 34355..34519

/codon_start=1

/product="hypothetical protein"

/translation="MWITVDKIQGYVGALHGNLWVRCTATYGFVARNKYENASNINAL

NDNSERLTCF"

CDS complement(34605..35039)

/codon_start=1

/product="hypothetical protein"

/translation="MLDFVFLVFLGLGISMVLVRRSSRKGMDLELTWLQSLKVVLVRG

AVALGLGFGIGRLVLMGMQYGFLSESVLREHALVSVAVVLICGLGSFVAFHWIIGRIS

GRSISVVSVIKAVVYESGYFVLSMLILLVILVLFGLAYETFL"

CDS complement(35057..36172)

/codon_start=1

/product="hypothetical protein"

/translation="MLRRENQGDLFDDQVVLPSLESVLTGISSSQRCDDRTTYFSAPD

VDLSLYDHIIVCLSGGKDSIAAYLRLVDMGVDKSKVEFWHHDVDGQEGSSLMDWAFMR

DYCRQLGEELGVPMYFSWLEGGFEGEMLKDNAYSHPHRVETPEGLLVLPRDHKRSKPG

TRLRFPQQSPSLQTRWCSSALKIDVGRRALNNQERFKGKKILFITGERREESANRSKY

NQLEAHACDRRYGKTARLVDAWRPVLHWTEEEVWEVIERHRILAPVPYRLGWSRSSCM

TCIYNSQRIWSTIRHYWPERAGKIAQYEQTFGVTVSRKKIDVIDLGSAVAPIQISDVE

ALEQVSREDYTLPIFVPEGQKWVLPGGAFGREACGSD"

CDS 36277..36402

/codon_start=1

/product="hypothetical protein"

/translation="MISTRKQSKGGFSVRLVQGQPVLLIENHQEVVFWCGYGLRG"

CDS 36440..37048

/codon_start=1

/product="hypothetical protein"

/translation="MKTIKRFIVWVNYGLEGWSIFGSSDDWDEAVSIRSEAIDECNID

EEDIILAENKNELVVKPAAKQMTEWHRELEAVLMTLDDCQMECDGMTWAVSHLLNDAG

VPHDCMYGFVRNEQTKDIVTPHFWVVLDDGWLVDLRLRMWLGDHDNIPHGVFHPDNEP

GFFYKGDPVQNHKGMRLGKAVLDIMTDGKISHVKVPERQDGE"

CDS 37053..37574

/codon_start=1

/product="Mobile element protein"

/translation="MEFDYDKSVSNAHLEAAGWGMDAFNHSNPFESHVIYVRDYRNDH

IRLFTIKKADFDTIKLPLHLTSDMLASVIAEFVSKAAKGKLNTKESDTLAPALVGYAK

STETYRSWRRVSGTTERLHMVINIYAGSGLLRPFIARAPETVLTTQELLVFSSQVKNM

DVSNHPEWFRGLR"

CDS 37577..38098

/codon_start=1

/product="hypothetical protein"

/translation="MKAVLWIFVLIIAPFVIAKVDQWRKRGIGDTWAWWKSENMPYEL

RSATLFLSEQDISTTQPVPMHGRVDQVYKAKNGVLIPLDTKLRQVNHIYESDIIQLSV

YRVILSHKYKAPVAKYGYVRTVVETADGDRVRYIKTNLLSEKEVVKLWHRYQSIRSGQ

VKTSCSCGGKFHM"

CDS 38195..38668

/codon_start=1

/product="hypothetical protein"

/translation="MAQLKHNRFVMLGSLVATVVAAANGLSFLTLMAGGMTLIQFMAW

KQQDAVRSRFKAAKDAFEALNVIAFDKHWVGSTATVAKVSNMITPPERLDKPWAVQVL

AVTKGGTWFAVDLQVTGTDKVQMLSLHQLSEKAAKTMLAFDLEVYEKFFGKPDVA"

CDS 38658..38972

/codon_start=1

/product="hypothetical protein"

/translation="MLHEDNVMEKVKSVDAVEFTAADKKAMTHAAYAQCFFILAQVMA

FPSLGVAGAMVAALCGMMPWLTKFAKEAPSKAFGMVMASLCLAPVYGKLCELVVHAIQ

GA"

CDS 38977..39831

/codon_start=1

/product="hypothetical protein"

/translation="MGVVRVPYLLAELKERGCADESALAQVMQPGCRIGEEDLRKLAA

NLGLEVSELAPAPENAANTRFKAKLRGGLASFLFEYDGCFRHAEGSSHAEMLGIEQED

DIGLPSRAADAMLLEKMLYQVIARAKYMLGKIDSKFVRSEQAIEFREQLAPGIFRPGY

RGFRFKEAAAGDLPTVMIDGRKFNCVASIARAHGLDPVTVRRRIADTRKAADKLSNDE

WKLILAKKKGKGKPFTYLDRTYSNIAQFCREHQLNTNLVYQKVKDRADSADEEFWGQI

IETCKRKD"

CDS 39831..40790

/codon_start=1

/product="Periplasmic serine proteases (ClpP class)"

/translation="MAENKEIPWEKELIEKYMFTLHKEQVKDRRWRTMLRVLRASGFV

LLMIGFIILASNPGGMPWQSAKAGAPHTAYINIRGEIAAGTLADADHLIPSIQAAFDN

PNSQAVVLRINSPGGSPVQAGRIYEEVKAQRALHPEKKVYAIIDDIGASGGYYIASAA

DEIYADRASLVGSIGVISSGFGFTGLMDRLGIERRAITSGEHKALLDPFSPLTSDMKK

FWEGVLSKTHQQFIERVKAGRGDRLKDAPEVFSGLLWNGEQAKEIGLIDGLGSLNSVA

RDVIHQSNLVDYTPTEDIIRRLTQRAKLEASSFVQELSAVKVY"

CDS 40806..41666

/codon_start=1

/product="Periplasmic thiol:disulfide interchange protein

DsbA"

/translation="MTEQSTKSLRLGVFAAIILGLVGTGFGIYQLVKEKDLAQEIANV

KFTVNQVKDAEGVTFKSKAEFEAAVAESINKFVAQKQQADIDQKYAQFEAAPEKVEDG

KHIYGDLGARFTLVEFSDMECPFCKQFHDTPKQIVDASKGNVNWQWKHMPLDFHNPAA

HKEALAAECIAEQKGNRGFWVFVNEIFHHSKGNGAGVSDLASVVTGVGADLDAFRECL

SSGKHEDKVQADIQKAKSYGVNGTPATFVVDNQTGKSQLLGGAQPAQAIMAVMRKMMI

ESQQDDSANQ"

CDS 41700..42128

/codon_start=1

/product="hypothetical protein"

/translation="MIKITTKLGCLLAGLLVLSACSSVPQTSNEYTKALDDTKQVCAA

CALVGNDLLVALNKSCDKPITPETLTSVMNSNSMFAAMMAINSIGGTDLYQVYRDAAI

DTLRCNEMDTWPERTKVRFQQPDMQKALALRVSVRQQNAN"

CDS 42186..42545

/codon_start=1

/product="conserved hypothetical protein"

/translation="MELQEAKNALDSLHPHKASAPLRLVIHQPGGIGGTPTVGVKAIH

AGFDWDSNTILIYPEEQLTRLTPDEVAAITKSVSKGQSWHSYQQFKKYREQLAEATEE

INRLRAELGRYQNNGRG"

CDS 42710..42991

/codon_start=1

/product="hypothetical protein"

/translation="MTLCSVVTAYLLVTYHRWGLMTARVWLLLSACLNGYAVYLSSHN

IQLVVALLSSLFIALWMLKTLEQPAVKGTYKVIADLHRQLWGMLKGQTQ"

CDS 42988..43506

/codon_start=1

/product="DNA primase"

/translation="MTTNTQNANANQVRSLRDMLVPALLFYVVMTAMFVGLDAFMDKP

TSMNLPFMPFLVSMVSFTSDARRAWDWRNGTKVVAVLTAVAMLLAFIYQLAVGEVNLL

GVGIYPATAILLLAITWVIRAIGKTAPFQFLGRHLARFGASKWVQRTAAVIVLAGGLA

ITVYAYWLNHGS"

CDS 43509..43736

/codon_start=1

/product="hypothetical protein"

/translation="MIIATKSGLLVAAELIKEEAGYWLLQPRDQKTPVRVNKQDDNKR

AFTHMGDALRWAGDPELAKQFDAEGEEHANS"

CDS 43705..44580

/codon_start=1

/product="hypothetical protein"

/translation="MPRGKNMQIRDYMTKLFEAFGDVEEVTREMLLEQAELIHTISDK

CQSTGLFLDSQVRFNQFVQEIEADDNVEDRLLHAWCWVMDRIVKAPTSFHMDGAVILT

MPLVARYLPPVEREPETIVVNLDEDYKAPVGNQTLCELIMERRHWPQGATCATQEADG

EILYWDAPVQVVEEGRKAAGKHGMMAEIGLKHQVDFWFSDMAETRLATDWNTAVITPH

CLLLSYLDVLQKNKVPFDEGVRLAAEWVTQLGGESRKDTEEEPEADATVLSLGRATAH

CFKPYPDTQNFYYEA"

CDS 44606..44797

/codon_start=1

/product="hypothetical protein"

/translation="MRYQVFKTKEGGLPVFTAPWYWLASAIAHWSSLNWDACRIVDSK

ADKTMLCWAKALPAAKKME"

CDS 44800..45327

/codon_start=1

/product="micrococcal nuclease (SNase-like)"

/translation="MRFGFAKKTGISAFLSAMLVLAPHVWAETFTAKVVGVSDGDTVK

VLTEQSCDTGKDCRSGKIQYRVRLAEIDTPEKKQPYGSKAKQALSDLVFGRMIKVEQI

DKDRYSRLVANLYVDGKWVNAEMVRSGSAWVYRQYAKTPELFKLETEAKADKRGLWAL

PESERTPPWEWRRKH"

CDS 45385..45657

/codon_start=1

/product="DNA-binding protein HU-beta"

/translation="MNKSELIMKVAEDADISKAKAEAAVNALINSVKEVLKAGGTVAL

TGFGTFHVKERAARTGRNPQTGENIQIAAANIPGFKAGKGLKDSVN"

CDS 45746..46039

/codon_start=1

/product="hypothetical protein"

/translation="MRLSSTQKDVLFILYAIEAGGKAEPVPGVKILEMINSARQSGIH

GTNFRTSCHTLVENGLLNKYRNASLKLAFRLTDDGRERAGEIYRKRLEEVQDK"

CDS complement(46066..46368)

/codon_start=1

/product="Transcriptional regulator, ArsR family"

/translation="MARTLDQMLATEKPEVVAKAQKAATEMLLNIHLAELRDRMNLTQ

GEIAASLGVRQPTVSEMEKPGRDLKLSSIKRYVEASGGKLRLDVELPDGTHYGFAV"

CDS complement(46373..46723)

/codon_start=1

/product="conserved hypothetical protein"

/translation="MWVIETTDTFDEWFDALDDTDRANVLASMMVLRDRGPMLSRPYA

DTVNGSSYSNMKELRVQSKGDPIRAFFAFDPKRKGILLCAGNKTGDEKRFYEVMIPIA

DREFAAHLDKLKKE"

CDS 46886..47434

/codon_start=1

/product="hypothetical protein"

/translation="MNNLPLLLDAREAIDYYHQHPDMTDAEKAYVVAFLSGEGRSNSQ

IREELGIEKVYTVTHLKRAGTLSEEELTLWLRNPRKITLGHVRAVAKLPISKREKLLR

DLLHTRTPVHTYEAIAKGKEVDRDADIKRLETLMSDATGRPIKIRYNPAKRSGELTLG

FFTLDDLDDVCKALGFDPSEQM"

CDS 47608..47775

/codon_start=1

/product="hypothetical protein"

/translation="MRSSGKEPDASLSGIAWKAQLIVAHERGRIDSLAVPLDARLIAK

NLIDEILAEEN"

CDS 47775..47969

/codon_start=1

/product="hypothetical protein"

/translation="MDEKITYEEMLEQLDQKGIRVTNGARRLYVALNNGVKAEVLGNC

GPATISLVDGMIVVEEQTLH"

CDS 47980..48351

/codon_start=1

/product="hypothetical protein"

/translation="MGKKVHIICGKCGSDEMNFVINGHCPDDPQNVASMSCSNCCELT

GIAEWSEFNGRELKGEAVALSTPANVNALLGLLRQAMDSVEYRLYGGGMSLSGNDAAE

LIDLQERAEKVLSVLRSDQHE"

CDS 48344..48814

/codon_start=1

/product="conserved hypothetical protein"

/translation="MSDLYEPLEFVFCGFRKGDAGLFISVATLRDGVLGREMYFSKGK

SKRRWVVGGIYSGASFSDNGAKGLDDAHYVKAWEVQGDKIEWQAKSEQAEALARSEKL

EADDRKRNELEELMLPIRKQYGALTKRRDRAGAAALEEAVLRALRAPIRKAEEK"

CDS complement(48829..49164)

/codon_start=1

/product="hypothetical protein"

/translation="MKHVLNIALMAVVVLGLAMLEATNWGYAVLAIPAVIWAKPVLNL

LHKLPVLAAAFWILVAVFAWQAALVGILFYGVLATPKAPANTNGKKSRKANLMGTYSY

DFKTGECFS"

CDS complement(49243..49530)

/codon_start=1

/product="hypothetical protein"

/translation="MDFIFRPDRINRRSQLSNVGRPKSKSITDYESPPSVDLGKPYPL

SDRVIVLGGRGRRAVYTKITEHKAFGLHIPFQTVSVHAALADNCTQSVTSF"

CDS 49752..50249

/codon_start=1

/product="conserved hypothetical protein"

/translation="MSMLEARYFVAKISDAQAVLCDEELATLERLIRKVDDGRRANGK

SSLTCVVVEEDWPNWQQTVDSVLSLADGKDNDWTNATPEQIKAFWVDDSVWKTLDGRD

KWIEDLDLLVDGSPVASEWEPFGLESGQSVVIRGGWIEGNALSDDGVPLVQAFVAWKQ

GQDNH"

CDS 50446..50616

/codon_start=1

/product="hypothetical protein"

/translation="MEGIGVCSSCPDALNQFASTSQEQATGQDDYRNSPEWKDFSSRI

GNALCGGRSKAK"

CDS 50811..51743

/codon_start=1

/product="hypothetical protein"

/translation="MEQAIQSYLADDRQYQDRITAALSQVEEKGAEYEALCQQRAQLG

IWQKIITFWQFRRDIAVIRSALKGHNSDLRYLRRGRDQLKEGLVSRAVKQAIDGSQIL

ERITQAQDRLDAASRLHESNKRLVDMGQKALREISEASSSISSAQTMEVLDLVTDNKG

ISVMSSMSNSSASSEIDDAKRAVKAFANALGDHRDIVGSLHHSMATEFIDLGMDFAGL

NDGFDFGSVFSLFSLSSASSSLDKVESRVESLMPDLRRAASNSAAEYARVNEEFFGLK

QQACCQVHELLVTNGIDVSVKRVESAVNSYRVGR"

CDS 51817..53280

/codon_start=1

/product="Cell division protein FtsH"

/translation="MSDITQQMDKLEIPIKLSFPVINVSTFELGRAESVFSDIAKKVG

KHFIVMPFKKLPDPGTMKAMVDESKKSSKNGVVVFDTFFFDRQRANPETLPALKSSLT

YLENEGINYIIAGKDVFNEEFVYHIDLPAMSNQEILKLLQTCEDNVKDGGVFESNERA

VIANHALGLSHTQMKNVFTYSAYLKFKGEEYLGEIRKEKAHILRDVGLDVLEAIDIGN

VGGLENLKEFLQIRKAGWDKDLPVKGVLLAGVPGGGKSLTAKAAAGVLGTTLVRLDMG

RFYSKYLGETERQFNRALQTIEQIAPVVVLIDEMEKFFGNADGEHEVSKRLLGSFLYW

LQERKEKIFIVATANRVQSLPPELMRAGRWDRAFFIDLPSVAERQKIFEIHLAKQKAN

IAAFDMPTLLRTTEGYTGAEIEQAVIDAMYLANAQDKELNNEALVDAVTRITPTSETR

REDINQIRSLRDQGFYPANNFDVQEQNGSGRKLAIED"

CDS 53342..53734

/codon_start=1

/product="hypothetical protein"

/translation="MSHIVKGKVQVAYKDKELLLKALEGVGVVVENEKLYRVGAGYTF

EKYPIVLIDQNNKEHRIGYKEKNGVWEQYQENYGSYGRWTQQASSKVQDRYIAFHYEQ

QLKEEGFSVTVKQHHDGTLELEAEEAVW"

CDS 53783..53977

/codon_start=1

/product="hypothetical protein"

/translation="MKKVNIKIKGGKIAADFTGFQGKTCEALEQRIRPEELEVEEKEL

KPEYHFNAGQTQHETEQNEW"

CDS 54022..55155

/codon_start=1

/product="hypothetical protein"

/translation="MVAAPAAFASDFVTGVMVGQMLSDDSGSKKVEDTGPESKTVIDY

HDGQPVVTKVEAFKSNKWRKLDGPQGGYFVCPGEYRSYSGRLRCRVHDDGLNGFMGGM

TDAQELPLQEALTKLEGKPISLQTIEVHGNNLAVKYQLAPPAQTHVKTQDLGVVKQDG

EMQKVASQPPALSIETAPKSEPPKQIGAAQDTDRNTSAFQSPFEEVSDSMIGLMDSSF

VKVIAGLMLVFGVASGIMRQSPSGIVMGIMPAIMIMTAPTVIRTMFDTGAASTKPVED

SGSSFPFFLVAIVPVLIFFAYRAFMNNRSDSEIDELLREARRAERAERPSNEPPSVDE

LRERQQQNPEREPVVVSSSAPAPKVQKEEPIEVQPGKRKIILD"

CDS 55167..55514

/codon_start=1

/product="hypothetical protein"

/translation="MSKKRIVIKNGEVCGFADEVSFKGLEVQEYSKTRVSRIVPTSGI

LMIAFYVIRGLCSDESKIAAWTRVWRCQWKVLIDGKSYGPFSSRADAISFEKDEIYKQ

GKFFADATHEAAV"

CDS 55511..55939

/codon_start=1

/product="hypothetical protein"

/translation="MMTRAAMAALLSALVIGLVSDLSAQELVVSQVAIDKSTEVEKEA

TFHNRKWVLRTGQVSGFFICQGDNKDIYYRHDRVGAHCQKTSTGWRNVLGLRDDVPEV

ELSVYLGTVEGVPVKVLHQEVDGYDLKVQYKVARKGSANE"

CDS 55962..56495

/codon_start=1

/product="hypothetical protein"

/translation="MVKIKILPSFVGVPFPSVLLIVAGLLNGPLWGFAAFVFHIVIKR

YIYREYRRLPYPMPTGSRPMDELSELLVKGIPEEFFRRLDAACSSDASVQITCPKKYR

LLKMSLNRYANRAGCYPQFREGDEIAIEVTEQRPRKAGAGKGFPSIQVLKASEGVLSE

PEQPIEIQKDKRKIILD"

CDS 56717..57076

/codon_start=1

/product="hypothetical protein"

/translation="MKMFEPRLFVSASEKGWFPLSLFVSIPFYFYLSYLLEIEEKRRT

FRANVIGFLRGVSEEIRRSTREHRDQGEIRHGGTELGSIDELQRGEAKEPEVISSTSD

QKEQGTPKRDKRKIILD"

CDS 57451..57624

/codon_start=1

/product="hypothetical protein"

/translation="MVGIRRNLELRKSLAGIERTIAHKEGQRPPGKEYQQRLEAYLQW

AMLALRLLKVKAS"

CDS 57579..57716

/codon_start=1

/product="hypothetical protein"

/translation="MGHAGAEVAQSQSKLVVRRKREPGTVKRRVFYAALFTVSKSDAI

K"

CDS 57741..58679

/codon_start=1

/product="Phage protein"

/translation="MAVINSLRALKGVTAPEELKKGDGFTISPQLLLEEEGFNTRGAF

CEDYYERPDIKAGIRVLADAYKRGDYVPPIIVKVIDGKVYVREGHRRRRAILLAIEEG

ADIQFVQVVEHKGDEAEQSLLIATSNDGLPLSPLERAVIYARLANWGWSDQMIAQRVG

RSAEHVRIARALLEMPLELKRMIQEGSVAATYAQELYNEHGTNAVEILKKAQEEQASG

NDGKKAPKKLTKKSVEKGPRLGKKVVEAMHRGVSSITSRLDNIKPNDDGETFTLTLSR

EDVDAFQELKAKLAELEPKTDESNEDQQELDLAGNQ"

CDS 58679..58876

/codon_start=1

/product="hypothetical protein"

/translation="MGRCTVEDAIQLLEGGTIVAVVPASSGPDTLVVSSIRLESGLTV

QFSPPAAASVSVQLAELDDEQ"

CDS 59114..59356

/codon_start=1

/product="hypothetical protein"

/translation="MAKLKVYGGITYGAEGQFRTVVAATSKSKAASILNITIYQMNSW

WTETFNKYEVEAAMSEPGAIFSKPLDGRDPFVKQEG"

CDS 59359..59721

/codon_start=1

/product="hypothetical protein"

/translation="MRSVFVWLKAGAILAFLFFGIAGAVNRVYWVNSQGLSQGSMSST

LVKEKFGLDLGAMCRYDKPQHVEFKVLPDQTIQFRCSWFEGGGLTWWPFYTEHDVKSA

EATSVLNDIFQGTEGHDE"

CDS 59711..60958

/codon_start=1

/product="putative DNA methyltransferase"

/translation="MMSSQLELFHVQEAYAKADKPLSNEELYDSVAELAGIPKSALNE

QSEIGKAKVKRSKLKRQIRWYQQTLKSMNLLQKVDGERGVWELSSKTKKGLHEALGGV

RLVAYSTNLGLAVWSNNKSFFSDLDEPVHLCVTSPPFPLRIQRGYGNVDEAKWVDFIT

QALEPIVKNLVPGGSVVLNVSNDIFEAKSPSRSLYVERMVLALHDRLGLSLMDRWPWI

NLSKPPSPTHWACVNRYQLCAGWEPVYWFTNDPDRVRSDNRRVLIPHTEKHQKLMAQG

GDNRVVSYGDGAYRLRGNAFSNVTEGRIPKNVIQRGHRCADTLELRRIARELGLPPHP

AMFPTDIPEMAIRFLTEEGDLVVDPFSGSNKSGLAAERNNRRWIACDIILEYIRTQAE

MFTGFDGFWMNPAIAAVGGGALN"

CDS 61008..61421

/codon_start=1

/product="hypothetical protein"

/translation="MAIIFVLSVLLVAGYIYYAGKQHQQAAINFWGEQYQPDAISTQI

DWGFIGNWVIPRGGPIISPGIAGVCPNTPLPVVPLKTGPDGRGYVLCGIGSEAVATSF

DVNDIQDEEIRNTLKTMFEEEFEKTVKGDKWTLKN"

CDS 61403..61714

/codon_start=1

/product="hypothetical protein"

/translation="MDTQELNHMIAEAYSRDLQKPELVSFKEVSRWGRKYGFPVVCTL

ADESEEKQIHWAASLLIQVAGTWPREDMPELLTPERGSALFNDAMQLLANGLGAANQL

R"

CDS complement(61729..61860)

/codon_start=1

/product="hypothetical protein"

/translation="MIKTTPKLSHVKDCSDHKMILQYLIMVLLKQTLNKKTAMKAAY"

CDS 61888..62673

/codon_start=1

/product="Chromosome (plasmid) partitioning protein ParA"

/translation="MAKVISFANQKGGVGKSTLCIQQAFYLALQKKKKVLVLDMDGQG

NTSSRLAPRRELEDGDYEPILTGTKTAELFAYELDGIEVMHCPCGADLIHTPKNDPDL

FEMEAVPLDQAMNPARHLAELFENYDYVLIDCPPSLGRKLVAALVMSTHVACPVKLSG

FAVDGVEGLLNTIIGVREAYNQNLEILGIVINDMDRSVNHDKALKSLENTVPDLLFEN

KIMHRPPLDTATTDGIPVWELRYGHVAAKEVEAVLEELLEKVG"

CDS 62677..63858

/codon_start=1

/product="Chromosome (plasmid) partitioning protein ParB"

/translation="MALNNLKGLSELAKAAKGKKGKEVLTVPVDDVVSKVQVRKRFRN

IEELAATLLTEGQQSPIIVFPKNEEGKFVIQKGERRWRACKHAGIETIDLVVNDKVQN

NLDETAGELIENIQRDDLTPVEIAEALNLFIEEGWKQKDIADRLGKNITFVSTHLSLL

KLPDCVRELYDNEVCSDTETLNNLRLLFDLNEERCRAVCAVAMSDGITRKQSRELLND

AKRIKDEMEKGPLTGSHQNDELGAGNTDEQSLNSGGDGTSEQTGNDDLNLAQEELEGG

KNSNGQDDDDEDPLRDEEGEHKDPVKQPDNSGKDKDEEGGDALPPLPKDKEWKNVRAD

SLIFAVNVNLDGETKRGVIMTDRVALVPSTVWVKTLDGEGKEKHVHVPVSDIELLSVE

G"

CDS 63907..64179

/codon_start=1

/product="hypothetical protein"

/translation="MKISQDMKRKFALVNALSKTEKPSLQDLHKATNIPESTIKRQLS

ALRDEFGMNILFVRESTGERGATGYYMLTDWGILDRSSFLNRYGKL"

CDS complement(64232..64867)

/codon_start=1

/product="hypothetical protein"

/translation="MSPETQQLTSKALSLIEQSRYRMGTSRFVEAFIDQWAYLQTGLY

PAKEEIPEELQPVAFELSHVLSAAIKRDPTSDVLGYVLSMSGFHKKGTNYFPTPPEIG

RLMSLIVGSQSSADFYEPCCGSGINAIHWMENLIENHGPEALREASIYLEDIDPLMVK

CCMIQLFHYFESRNTTPKTLSIVGIDTLSRRTKNIAYYAEKPPATAATVAA"

CDS complement(64959..65102)

/codon_start=1

/product="hypothetical protein"

/translation="MLQGNTNTNKRIPLAAFMKWPLAKRQDFIDREMSGCPDFIKTSL

LRH"

CDS 65558..65806

/codon_start=1

/product="hypothetical protein"

/translation="MTTFHCHKTVHSKSGGEWDEEGNYAPSGQESMCAGAAAYLMKIG

RPTVAMRIAFAFGDAKVSDWDEAQELVVEPLVQGDRNE"

CDS complement(65949..66068)

/codon_start=1

/product="hypothetical protein"

/translation="MFSSIFFLSNRFDKFNGVHAYMTDNGFQAAAVAWFKGQF"

CDS 66055..66729

/codon_start=1

/product="hypothetical protein"

/translation="MDENKLNIETVDGHNELVVSFLSRMVSLSDEEKQTVLSCLPDTG

KQTITQLYEALRSQGHQDLAEKVEPYLQQGVFGPIFDNAKSKVFVRDEAPFFLMDENP

LNWDDAKAFNRLRMSTTCVLGRGGWTIGERFDDRFDTEVGGTQLIVTQSLNEKGEIEG

GLPTSMSLNDFAEFPKQPRPPQIVDYQEDKRYTLEEAEAIPELAPVVQRLKERIEEYE

ERRAHD"

CDS 66722..67228

/codon_start=1

/product="hypothetical protein"

/translation="MIDLTCRLCAGSGVYDFSPSKETCGMCQGHGKFVDAKAMLVAAI

ELAKKQREPVIEVVMGLVLQGVDVRGKEPGERFKAAQAFNAPGHNLLRYGWDAWELYA

LHEGVSPELASLGRSVMREWHSHSWGRFSGEVGFNAAEIMIKQAKDNPEKAEERWAFL

LSEEWMVE"

CDS 67234..67545

/codon_start=1

/product="hypothetical protein"

/translation="MTEQTNKKLMLDESPDMPLQFKWRKFGGAIFTATQTMEHVKEGR

KLGPTPAGMLPQEVWLNRLIAMEETLPRGKFFDRFRRRDKNEQYDLAADHLRLVGQKK

R"

CDS 67554..68054

/codon_start=1

/product="hypothetical protein"

/translation="MKAQAYPPSVIRKGAVLYAALYYISDDDKAKVEVTEWIVRSIQK

RRNSTSDQRYVNLAQKLDGITWGKRSRKNGDFGWLPSIPSWCLKQFREGGELPFGVYT

TRLAALKFAKVSLQEEVQYCEAELKKAQTEEDTQELQEELAENQRLLKAAGAMVKREQ

NKKKRG"

CDS 68058..69485

/codon_start=1

/product="hypothetical protein"

/translation="MLPIVSPSVVTKQLAFNRVGDKRKVRVSSNFLDVMGFKPGMGIA

VEPGEGMGGFSVIPATDELQTHQVYQRRYQPKSRSNNPLETVIEFSGQGLIDKCFPRY

TERFHVEMRKGRVVFTPVANRAFAIADRFRKTSPFRAFVALTGGVDIHVMESLGWKAE

IVLEHRPVEARDRASGRNLSEVHALNTLVNSSPRILLNEDIHHLELDRLGALLAECPP

IGLAHYSLGCDDHSNAKSPRDKERSLEDLSTMLDMVYPALKQIEVVNPAVVLVENVPN

FKASGAGAMMGTTLRRMGYFLTEMVLNGLDFGAYQGRERYYMVASVFPGFVPPKPEQR

AGGRLWPVIEKHLGDCADVTALKSIQARESTSRRMPAFLTRESTSCPTILKSQDRGVK

DAVYIQDGGRIYKPSVDLVQELMSIPDSFDISWMAKEQATETLGQSVDYRLHSAVMAA

VRDHLNVNCGRHTVVQHGIRSKEGK"

CDS 69485..70141

/codon_start=1

/product="hypothetical protein"

/translation="MAVIYYGEGTHDAGFVGFRVARTVGVADDYRQEYFSLREYSYAT

AHRLAYSLDRKWEAEAEEVKRQNKTCKRRRNSGPNIIAEGLRAYISIENRSRMGVKRT

YFAPCFLVTKPGYGNGDIVFRISTHGYAEAYEKAVEKYCEIHDLTDEQYVELLDRMPS

TEVFTGYLLNALLIRGHRATKAEILSKLGAAKNEDDITNSKGKSGHNRVRCPEYRWAQ

"

CDS 70197..70814

/codon_start=1

/product="hypothetical protein"

/translation="MFFDNKVESHSLVMGASGKGKSVLSEQVRKNARLRGDLLVDTEM

YREGRGLKPYEHEYARRLVLGLSGPLPRELRGKPVTVISDVSRPKKVKRQPKQFVKTV

NGVTLERQLVADARDQLEMQTGVWLKQPQLIELMEESGIDETLADFGEAETQIREMLA

DALAMKLVGRSWPKCGALYNAAEKSDVNFSSELDAAAKEAGYMVR"

CDS 70815..71021

/codon_start=1

/product="hypothetical protein"

/translation="MGFGVDKIDRQSWLVKFRRAKCQDTLDTMRDAAIRNYEGNIRVI

ADIVLAHEARETEIEKGMFCLIVR"

CDS 71047..71325

/codon_start=1

/product="Phage protein"

/translation="MKKRILHLPVKKIYFDQIKSGEKPDEYRLVTDYWIKRLEGREYD

EVHVKCGYPKAGDMSRIEIRPWRGFSRNVITHPHFGDYPVEVFAIHVN"

CDS complement(71416..71904)

/codon_start=1

/product="hypothetical protein"

/translation="MFGKIILTAMLTTSSATAEDAGKNIASGLATASTRQIGQAIMPT

LAIGSAIAKSAGKGMVLGFAETSASYISHDDLSATRSYQNPEAVDMAKGLGTLKDVPD

FLYVITDVNANMADKCKRVWEPQSLALSQLIVELVALRKTNHKGSYEQALSHLDCSIF

TN"

CDS complement(71919..74111)

/codon_start=1

/product="DNA topoisomerase III"

/translation="MDLYICEKPSQAKDLAGVMKASQRGDGFLHDGGNRVITWAFGHL

LELYMPDDYDERYKSWSLETLPIAPESWRYNVRKSAFKQYKIVEGLVKKASTIYISTD

YDQEGEAIARSLLDRFRYSGPIRRVCLTALDESSIKKALNNVKDGKDTVSLYYAALAR

QRADWLVGMNVSRLYTVLARDVGFNHTLHVGRVITPTVALVCQRDREIAGFTPSPYWT

LGVNVSVQNGQFAAQWIPPEECSDEQGRCVNKAYAEQVASQVNGANAVISKAETKPGK

ESAPLPFDLTSLQQYASKRWGYTAQQVLDAAQALYETHKATTYPRTDSRYLPESQKED

IPDILQALILSDQNVSGLVAGADPHRKARVFNDAKVTAHHAIIPTPARTDISAMSEIE

FNLYDAIRRFYIAQFYSEFEFTKTSIEVQCGRHLFASAGKTPTKQGWKVLFASDSESS

PKDEGEDTDAPVEQEKLPRVSQGEPALLNGAELANKMTRPAPHFTEATLLAAMENIAR

FVTEEKFKQILKDTAGLGTPATRASIIQGAVDKGYFKRQKKVLLATDKAHALIAVLPP

AIKSPGMTAAWEQELEKVASGSGNMSVFMKQISTWICQMVEQLKVAAPVLTKEGGAMA

KAFEGAKPPSHECFNCGGEMHRIKGKNGFFWGCQNEACKKTFPDNRGKPEKRIAAEDC

PDCPDCGSPMRLRKGKAPGKKRASKFWGCTAYPDCKGTMPFKKSDFMD"

CDS complement(74111..74344)

/codon_start=1

/product="hypothetical protein"

/translation="MQSSEIRNQTELGRKAELFDALLIMLQEAGSRGNSSEAAYVISG

VLENLSRDYPEVKGLAQSWTELANLESKMRGAA"

CDS complement(74326..74943)

/codon_start=1

/product="hypothetical protein"

/translation="MRQLIAILGVAILAGCASNAPEQKPQPQTAPAENDKPARVSREL

SMAWDNMGRGGAALRQPGYIHVLGDGNVSATMNKVKDDSAGSDKTPAGIKQHGDVNEA

INTFKSMNKGKGYSLYELSRWERYCDGGKGMDEHDWRFVEAEGTTNIPKDVVTGCIPP

THTYKDYLNAWTHFCTSQAVTDADRRIVRESVRPYSVVNPCKALK"

CDS 75111..78083

/codon_start=1

/product="Conjugative transfer protein TraI, relaxase"

/translation="MLKALNKLFGGRSGVIETAPSVRVLPLKDVEDEEIPRYPPFAKG

LPVAPLDKILATQAELIEKVRNSLGFTVDDFNRLVLPVIQRYAAFVHLLPASESHHHR

GAGGLFRHGLEVAFWAAQASESVIFSIEGTPRERRDNEPRWRLASCFSGLLHDVGKPL

SDVSITDKDGSITWNPYSESLHDWAHRHEIDRYFIRWRDKRHKRHEQFSLLAVDRIIP

AETREFLSKSGPSIMEAMLEAISGTSVNQPVTKLMLRADQESVSRDLRQSRLDVDEFS

YGVPVERYVFDAIRRLVKTGKWKVNEPGAKVWHLNQGVFIAWKQLGDLYDLISHDKIP

GIPRDPDTLADILIERGFAVPNTVQEKGERAYYRYWEVLPEMLQEAAGSVKILMLRLE

SNDLVFTTEPPAAVAAEVVGDVEDAEIEFVDPEEVDDDQEEDVSALNDDMLAAEQEAE

KALAGLGFGDAMEMLKSTSDAVEEKPEQKDAGSTESSKPDAGKKGKPQSKPGKAKPKS

DTEKQPHKPEAKEDLSPQDIAKNAPPLANDNPLQALKDVGGGLGDIDFPFDAFSASAE

TASTDATNSEIPDVAMPGKQEKQPKQDFVPQEQNSLQGDDFPMFGSSDEPPSWAIEPL

PMLTDAPEQTTPAPAMPPTDKPNLHEKDAKTLLVEMLAGYGEASALLEQAIMPVLEGK

TTLGEVLCLMKGQAVILYPDGARSLGAPSEVLSKLSHANAIVPDPIMPGRKVRDFSGV

KAIVLAEQLSDAVVAAIKDAEASMGGYQDAFELVSPPGLDASKNKSAPKQQSRKKAQQ

QKPEVNAGKPSPEQKAKGKDSQPQQKEKKVDVTSPVEEPQRQPVQEKQNVARLPKREV

QPVAPEPKVEREKELGHVEVREREEPEVREFEPPKAKTNPKDINAEDFLPSGVTPQKA

LQMLKDMIQKRSGRWLVTPVLEEDGCLVTSDKAFDMIAGENIGISKHILCGMLSRAQR

RPLLKKRQGKLYLEVNET"

CDS 78080..79945

/codon_start=1

/product="IncF plasmid conjugative transfer protein TraD"

/translation="MTMSYDPLAYEMPWRPNYEKNAVAGWLAASGAALAVEQVSTMPP

EPFYWMTGICGVMAMARLPKAIKLHLLQKHLKGRDLEFISIAELQKYIKDTPDDMWLG

SGFLWENRHAQRVFEILKRDWTSIVGRESTVKKVVRKIQGKKKELPIGQPWIHGVEPK

EEKLMQPLKHTEGHSLIVGTTGSGKTRMFDILISQAILRGEAVIIIDPKGDKEMRDNA

RRACEAMGQPERFVSFHPAFPEESVRIDPLRNFTRVTEIASRLAALIPSEAGADPFKS

FGWQALNNIAQGLVITHDRPNLTKLRRFLEGGAAGLVIKAVQAYSERVMPDWEAEAAA

YLEKVKNGSREKIAFALMKFYYDIIQPEHPNSDLEGLLSMFQHDQTHFSKMVANLLPI

MNMLTSGELGPLLSPDSSDLSDERQITDSAKIINNAQVAYLGLDSLTDNMVGSAMGSI

FLSDLTAVAGDRYNYGVNNRPVNIFVDEAAEVINDPFIQLLNKGRGAKLRLFVATQTF

ADFAARLGSKDKALQVLGNINNTFALRIVDGETQEYIADNLPKTRLKYVMRTQGQNSD

GKEPIMHGGNQGERLMEEEADLFPAQLLGMLPNLEYIAKISGGTIVKGRLPILTQ"

CDS 79995..80540

/codon_start=1

/product="Conjugative transfer protein 234"

/translation="MVDVNPFDRVMNELKSRGRKNAHILSILQFDWPASEAIIEKLSC

YITDGIKANQEPVIYPIIEEALHRYSQLVFHEQREKYEDPARIGAFLETLITETCRAL

EVQIVDSGGDSWSVDSGESFSLWLSSHPGELSINPQPHEDETSLRGLLYELITCESVK

TVLRRTDYEEAVVAGRMAAGY"

CDS 80497..81126

/codon_start=1

/product="Conjugative transfer protein s043"

/translation="MKKPWLLVAWLLVIELLAILLLIPGDWTDRAIKRESELVEQSLG

VEARDWIQNKASTWFRSSVIDSGFYEGMYQTLIPSEEERQKSKGMQDMGKGWFVWVKG

RMEAFVNVIYQFYTRLALLAAWAPYMLILFVPAVYDGMMTWRIKRTNFDYASPVLHRY

SVRGTMYLMAGLFIAFFIPIALDPVVIPMTMMTCCVLVGLTFGNLQKRV"

CDS 81174..81350

/codon_start=1

/product="hypothetical protein"

/translation="MRHPIERLPGDTSPILVCVVALLGAELEFGEGAHCLRRKDDLCD

AGNVVLGAGGVPLG"

CDS 81592..81969

/codon_start=1

/product="hypothetical protein"

/translation="MKVTNRLQLLALGLCIAGTAIAAPDVLNDDAKLKNLEKVCPDCQ

MVAKDVLNLRVENCQLKDTSSAMMIGTMQNDPMFSFMLAVHTAAGSEAYKTVVGAAGN

HVDCENPLNWIKLTQQAIKGGKV"

CDS 81969..82631

/codon_start=1

/product="hypothetical protein"

/translation="MRKRDFFFGEVYEGSGGATLRLSDMEPLARKVSAEFFTAQLNRI

LKEHDGQLTLSDGTSYPSFWSFIDKVDPEQVGFVEIYARQDVNDNVEATLACDIVLVN

GVITVKPHWCAYKDIRADEVISTLLVPLHLKALQGKAYIRWDDGETEPLLQNDDYQAE

LENVFSVSKYPSAMSWGDTADQKVKQYKMDLECATDVGRRGVSSEQAWDAYRELRYNR

TV"

CDS 82812..82955

/codon_start=1

/product="hypothetical protein"

/translation="MSAQAHVRESESSSGSFISWQFLVWQVMFVIGVVAGMNLEHAFN

FLG"

CDS 82967..83332

/codon_start=1

/product="hypothetical protein"

/translation="MNLNKQFFWFLMNAVLMCIFIVPVAVAFWLSAFAAGFDWSQWVK

LAADTANRAASDPAKALGTVQTYWGILSFFLLAAYSLMFKFKANANKEVKTLEVARPA

NEVSVAASEKHSEAVPQNQ"

CDS 83477..83758

/codon_start=1

/product="IncF plasmid conjugative transfer pilus assembly

protein TraL"

/translation="MKPVKIPRRVDEPPHLLLWSADELAPMLLGLTIGVIIGKALICF

LGGLLVTNLYRRFRDNHPDGYLLHMIYWAGFIMTKAKSLKNPFVRRYLP"

CDS 83755..84381

/codon_start=1

/product="IncF plasmid conjugative transfer pilus assembly

protein TraE"

/translation="MNLKKYLKTWEGTQTENKWGRIFQGGLIAIVFLLVVQVFSKETI

VTIQPFTLTEEAWVTKSNASQSYKEAWGFAFAQLLGNVTPGTVDFVKERITPLLSPSI

YQDVIDAIEIQAQQIKNDRVTMRFEPRFVEYEPKSDKVFVYGYSYVKGASSNEERSER

SYEFAIKISNYAPVLDYIDTYVGKPRTKTVLEQLQRKEENRRKHEEQR"

CDS 84422..85282

/codon_start=1

/product="IncF plasmid conjugative transfer pilus assembly

protein TraK"

/translation="MAYASDDIPVVPASVMKKDVPAPVTSGQSSHEVVGSMNENPLLT

MKPGVNQIIPIAVGHPNRIVTPFSNPEIVSTSLTGATDNGQCGEVCIKENVVYVATDK

QYPVTMFITEKGSEAQALSLTMVPRRIPPREVFLKLDGGVGITGAFANTKAETWEQSQ

PYVETIRSVFRKIALGEVPQGYTLNRIPAGAAVPSCAHPGVKVDFSKGQYMMGHHLNV

FIGVALNVSDQPIEFKEALCGSWDVAAVTTWPLNVLEPGQKTEIYVAKKQKRGLAPTS

KRPSLLGGAQ"

CDS 85279..86595

/codon_start=1

/product="IncF plasmid conjugative transfer pilus assembly

protein TraB"

/translation="MIKRFWTQLDPNKKRWVSIAGGVFVLFAVVTMFSGEPKKEEKRG

RQETIKHVLTDKNTREIGIDSLSADVKMVSRENSDLKKELERVKKELEETKTTAGKSS

DVGREMTRLRQDLDRLTQKNMELAKKVETGAAGGKTSSSSEDARADVNGASGGDGQFM

EKKLDYKDPASFFRDAPLPDSKGGAPATGKGDGRDATKPGIQIVSYSQKAPEVEEKDN

KDDESIYLPSGSILTGVLINGMDAPTSQGARRDPFPSTLRIQKEAILPNRFRADVREC

FLIVSGYGDLSSERAYLRGETFSCVRDDGGVIEAKLDSYAVGEDGKAGVRGRVVSKQG

QIIAKSLMAGFLGGVSEAFDVNPVPVVSTNPGSNTQYQSVFSDQMLQGAAVKGASKAL

DRIAQFYIDMAEGIFPVIEVDAGRQVDIIVTKGTKLQIRSTGGTKK"

CDS 86592..87170

/codon_start=1

/product="Conjugative transfer protein TraV"

/translation="MKNLNILTRKGSSRGEAQKEQAVRSAKMLGVGAALLILSGCSTF

NIGKDEYSCPGMPNGVQCMSARDVYAATNDGNVPRPMKPEEVEAKAEADGEGSSNVSA

NSSSSGDPVIDNYVAPRLPDRPIPIRTPAQVMRIWVAPWEDTNGDLIVTGYVYTEIEP

RRWVIGDGTPQSEPVLRPLQTVQHEPKSETTK"

CDS 87183..87566

/codon_start=1

/product="Conjugative transfer protein TraA"

/translation="MNANQLANASSKNNALFLFLGLMVVAFLLVPDQAHAGTGGTAFD

DVWVTLKDWTQGTLGRIVAGAMILVGVVGGIARQSLMAFAMGIGGGMGLYNSPTVVES

IMSATLEHAEKVIPAVVQLSNGLGV"

CDS 87851..89113

/codon_start=1

/product="Mobile element protein"

/translation="MINKIDFKAKNLTSNAGLFLLLENAKSNGIFDFIENDLVFDNDS

TNKIKMNHIKTMLCGHFIGIDKLERLKLLQNDPLVNEFDISVKEPETVSRFLGNFNFK

TTQMFRDINFKVFKKLLTKSKLTSITIDIDSSVINVEGHQEGASKGYNPKKLGNRCYN

IQFAFCDELKAYVTGFVRSGNTYTANGAAEMIKEIVANIKSDDLEILFRMDSGYFDEK

IIETIESLGCKYLIKAKSYSTLTSQATNSSIVFVKGEEGRETTELYTKLVKWEKDRRF

VVSRVLKPEKERAQLSLLEGSEYDYFFFVTNTTLLSEKVVIYYEKRGNAENYIKEAKY

DMAVGHLLLKSFWANEAVFQMMMLSYNLFLLFKFDSLDSSEYRQQIKTFRLKYVFLAA

KIIKTARYVIMKLSENYPYKGVYEKCLV"

CDS 87851..89113

/codon_start=1

/translation="MINKIDFKAKNLTSNAGLFLLLENAKSNGIFDFIENDLVFDNDS

TNKIKMNHIKTMLCGHFIGIDKLERLKLLQNDPLVNEFDISVKEPETVSRFLGNFNFK

TTQMFRDINFKVFKKLLTKSKLTSITIDIDSSVINVEGHQEGASKGYNPKKLGNRCYN

IQFAFCDELKAYVTGFVRSGNTYTANGAAEMIKEIVANIKSDDLEILFRMDSGYFDEK

IIETIESLGCKYLIKAKSYSTLTSQATNSSIVFVKGEEGRETTELYTKLVKWEKDRRF

VVSRVLKPEKERAQLSLLEGSEYDYFFFVTNTTLLSEKVVIYYEKRGNAENYIKEAKY

DMAVGHLLLKSFWANEAVFQMMMLSYNLFLLFKFDSLDSSEYRQQIKTFRLKYVFLAA

KIIKTARYVIMKLSENYPYKGVYEKCLV"

CDS 89094..89216

/codon_start=1

/product="hypothetical protein"

/translation="MKNVWYNKNIINKIECCSVDNLQSLLSIIAAKMKSMIYQK"

CDS 89094..89216

/codon_start=1

/translation="MKNVWYNKNIINKIECCSVDNLQSLLSIIAAKMKSMIYQK"

CDS 89437..90582

/codon_start=1

/product="Beta-lactamase"

/translation="MMKKSLCCALLLTASFSTFAAAKTEQQIADIVNRTITPLMQEQA

IPGMAVAVIYQGKPYYFTWGKADIANNHPVTQQTLFELGSVSKTFNGVLGGDAIARGE

IKLSDPVTKYWPELTGKQWQGIRLLHLATYTAGGLPLQIPDDVRDKAALLHFYQNWQP

QWTPGAKRLYANSSIGLFGALAVKPSGMSYEEAMTRRVLQPLKLAHTWITVPQNEQKD

YALGYREGKPVHVSPGQLDAEAYGVKSSVIDMARWVQANMDASHVQEKTLQQGIALAQ

SRYWRIGDMYQGLGWEMLNWPLKADSIINGSDSKVALAALPAVEVNPPAPAVKASWVH

KTGSTGGFGSYVAFVPEKNLGIVMLANKSYPNPVRVEAAWRILEKLQ"

CDS 90676..91209

/codon_start=1

/product="Outer membrane lipoprotein Blc"

/translation="MRILPVVAAVTAAFLVVACSSPTPPKGVTVVNNFDAKRYLGTWY

EIARFDHRFERGLDKVTATYSLRDDGGINVINKGYNPDREMWQKTEGKAYFTGDPSRA

ALKVSFFGPFYGGYNVIALDREYRHALVCGPDRDYLWILSRTPTISDEMKQQMLAIAT

REGFEVNKLIWVKQPGA"

CDS complement(91206..91646)

/codon_start=1

/product="Quaternary ammonium compound-resistance protein

SugE"

/translation="MNKKVEIHFLPGKNAISHISFAGRPANASFTGDGPIVLEPDMSW

IVLLIAGLLEVVWAIGLKYTHGFTRLTPSIITIAAMIVSIAMLSWAMRTLPVGTAYAV

WTGIGAVGAAITGILLLGESASPARLLSLGLIVAGIIGLKLSTH"

CDS 91780..92205

/codon_start=1

/product="Putative luxR family bacterial regulatory

protein"

/translation="MLKILVIDRCHFTRTGIEALLNHSGRFSSSFLVSGINNLLLAKE

HILQWKPHLVIADLYSFISETHSSPPIKPFFMSCGVIPLILLQSADRQHAPIVPSQSV

AHSVLTKHTTLNTLSHTIQDFLICSKRALPQPMFMHSLP"

CDS 92251..97737

/codon_start=1

/product="Conjugative transfer protein 123"

/translation="MINFKPKIPAMLGALAVLTAGAAHAELLEYTFKAPDGTQRSLTP

NANYANPTGNISFALSAGIDRKVKISVLRSDGTVVSTATSHLLGATDRITVGGKSYYG

AELQLPAPVGGAYTIRAEILASDGSTVQTDEYPLTVDVTPPTYSSLAPVYSNYGQVTS

GDVWKLGLGGSEDNAFLLSGISDESPIKGVKAKLYRQDGSLYKDVSVNYDDANGQARQ

SFQSGFFPASDLDEVFTLQFEISDSAGNSYLSPRQKVMFDSITNAPSAPFGVYDPSST

NNLGPGLTGFVAYTEGMTVKTNPIKLAWRVPRDNWHEYREGGINMTNALGEMSKVGED

ASYVYLVTTAPYGNTDGNYWRWVNFGQWGGGGIAYNLTLSPSAPKSPRLLGVDYNYSD

IGWSSFYRYWVNNSVLPVTVSSIRVKVEPRPYVQTAVHRGSCEIPVGQDSCVIANSFT

MAKGTTGYVHDNATVFNPDKSLRSNPLWAEVNWNDQHYPQLSQQFDQNSKVFTLFVNQ

PGRGAYFDRLRLRSAWIEDSKGNKLSPTGGLIANNWENYTYQWDLKTLPEGQYSLVAA

AEEMHGPLTRQPMFQITSDRTPPTMTLSVADGAAIQTLDDVVITLADAIDPSPKLTSI

ALVGGPANDKVQLSWREESKGRFRLEYPVMFPSLKEGESYTLTVSGEDAQGNAVQKAV

GFEYKPRQVMLADGMDGKVMVPAVTHEFVHADGKRIIETKPLTLSDGAVVTGSYDVFA

TLRSDAKVPLVVNGVRIEPGQTMGIMSQHDFGASGGRLSIPVKPAVPDVVGSSSLLVM

TSAPNSPILVVDINTWKGTAKLSAESWTIRQVIDPVKIYALPESGVPCRFTTKEDVAM

AADPIRDPVCLLQWDRTPDEAEQTTQDNNGMKVAGLVGQAVSIGEQPVEYSLYLFSGD

GSKVKVGSGSQNLTVTTAYGSVGYTPIDDIAQVNRVIEDFDVNFKQSKGPDCSITLSA

DRAKKEAANKAVGSASRACLFEWQQIPDGLVQDPLSESPSLSGSLASNGVHPLGWRVS

IFTRNGTRVTLNDETFNVEAVDPPAPTVELASDYNFKDNIYLVPMTGNYLGDAIINSE

RADLDIAISRNSDVLESETFTPGWGATNKVYRRINTDERALWEETTYKVNAAYNKVPD

VKTEVVYRAISAPSDSIRPIVEVKGDTAIDTQALPVRVLIRDQYKPDGDYDANTMGVW

KVRLIQQKAYNETVALTDYAEASNGEAQFSVDLSGVDTSSVRIAAEAVLESPVEGYNR

TELSIRPAFLTVLRGGAIGAGVEARKLSGEAPFTAVFKLSLDDRQDLRATGQVVWETS

KDDGKTWEQFIPEDRYKYQLVKTFDKGEYQVRAKVVNVNSGAEKYTEAVSVVAYDKPD

IAVIGPTTLFVGSEGKYTANLTLNDEPISGGNAIVEWSTDGGKTYAQTGDSITLSSDE

ETRYRLWARVRSATAPADDGYAYEVAKTAVDFRAVKAPRPYVTGPRVIETGKKYVFKA

ETSLPYRGMDVKLNGFFTLPDGSIVQGDTAEYEPSDTDLNQATVETKYTTWIEGYRDQ

GAEASHSLRSRVWQYVWPSFGMQVRKNADVAPATITASVRPIAFNGKLEEPTYEWELP

EGAVIQDQRQDIVRSFVINEPGDYNIKVTVRDARGHETVIEQPLKIGQAAPYAIDLQY

SGSNKYEREPLDVLLRPYISGGHPRDRISTRVYSVDGTPLESSGYYGRATLGAGEHSI

KLKITSEMGHEAEGEVNINVAENKLPACSLSSRETVGSWIVYANCEDTDGRMKSYEWT

IAGELQSISSDRVTISKGTYETMPTISLVGVDDSGGKSEAVTMN"

CDS 97886..98593

/codon_start=1

/product="Thiol:disulfide involved in conjugative

transfer"

/translation="MRTKLLGALMVFGIITGTAHASSKLEITDPRAAKIEDIVELPIK

GVRAVQSDGQIMFLSENGRFVISGQIYDLWSKKPLNTMSQMRDVAERIHFKSMGMDVD

TLNTVSMGRGDKEVVVFVDPRCAVCHQLMGDAKSLVDDYTFKFIVIPALGAESNRLAK

NLYCAKDKTHALDALMNNTLGSLPSKETCDPGQYDQTLLTAHFIGIEGVPFVVAPDGR

VSKGRPKNLKSWLESAE"

CDS 98590..101037

/codon_start=1

/product="IncF plasmid conjugative transfer pilus assembly

protein TraC"

/translation="MIVTIKKKLEETLIPEHLRAAGIIPVLAYDEDDHVFLMDDHSAG

FGFMCEPLCGADEKVQERMNGFLNQEFPSKTTLQFVLFRSPDINQEMYRMMGLRDGFR

HELLTSVIKERINFLQHHTTDRIFAKTNKGIYDNGLIQDLKLFVTCKVPIKNNNPTES

ELQQLAQLRTKVESSLQTVGLRPRTMTAVNYIRIMSTILNWGPDASWRHDSVDWEMDK

PICEQIFDYGTDVEVSKNGIRLGDYHAKVMSAKKLPDVFYFGDALTYAGDLSGGNSSI

KENYMVVTNVFFPEAESTKNTLERKRQFTVNQAYGPMLKFVPVLADKKESFDTLYESM

KEGAKPVKITYSVVLFAPTKERVEAAAMAARNIWRESRFELMEDKFVALPMFLNCLPF

CTDRDAVRDLFRYKTMTTEQAAVVLPVFGEWKGTGTYHAALISRNGQLMSLSLHDSNT

NKNLVIAAESGSGKSFLTNELIFSYLSEGAQVWVIDAGKSYQKLSEMLNGDFVHFEEG

THVCLNPFELIQNYEDEEDAIVSLVCAMASAKGLLDEWQISALKQVLSRLWEEKGKEM

KVDDIAERCLEEENDQRLKDIGQQLYAFTSQGSYGKYFSRKNNVSFQNQFTVLELDEL

QGRKHLRQVVLLQLIYQIQQEVFLGERNRKKVVIVDEAWDLLKEGEVSVFMEHAYRKF

RKYGGSVVIATQSINDLYENAVGRAIAENSASMYLLGQTEETVESVKRSGRLTLSEGG

FHTLKTVHTIQGVYSEIFIKSKSGMGVGRLIVGDFQKLLYSTDPVDVNAIDQFVKQGM

SIPEAIKAVMRSRRQAA"

CDS 101052..101369

/codon_start=1

/product="Conjugative transfer protein 345"

/translation="MDIKSIAIAAILGAAGGFGGSYYVMSEQTASIHQRLNQTPPVVV

VDFAKVASAYPAGASQEEVERLMVKTNDAILKLKDAGYLVLDASAVVGAPSDVYLPDE

VLK"

CDS 101366..101896

/codon_start=1

/product="Conjugative signal peptidase TrhF"

/translation="MNFPLKKYFVKKESWKRFGVKAGVTLVVLWAAGAAFASRYRIGI

DPQQEKCLPGYTFFLIDLNDQTLERGAVYAFQAKNMQPFYKDGTRMVKILTGMPGDKV

EINDKWKITVNGDVVGEGLQLAGKLHLPESHFYGKTTLKENNYWFMGKSPFSFDSRYW

GTVKNDQIIGRAYPLF"

CDS 101916..103124

/codon_start=1

/product="IncF plasmid conjugative transfer pilus assembly

protein TraW"

/translation="MFSVAGGAYAQESPLTEQDKALIEQGKQIAQKAQKMEMPSLLQN

QHMDEAQAEAKAFFKQLQTTNPTLKEMHRKQAEKGIYSDHRILVFASLSLGEQGLDDV

LTAVSGQPDSVIVFRGIPEGMNLGQGVKAIQALAAKKDPVPNIIINPTLFKTYNITAV

PTIVMLEDEPLPGEQPNVVAQVSGLSDPVWLAREVDNGEKGDLGVKGPVEKISEPDLI

DVAKKRLANIDWEEKKKQAIERFWTKQNFNELPRAPKSRTREIDPSVMITSDISTPDG

TVFAHAGDVINPLCDPKEVCKPGTRPFTQAVVVFDPLDKKQMELLAKKLPEIKLEPGV

QRITYIATEFDKDKGWDSYKSVTDNFDAPVYLLTPDLITRFELEHTPSVITARGKKFV

VRELAEEGGE"

CDS 103121..103792

/codon_start=1

/product="hypothetical protein"

/translation="MIFAPAFQPIKDVGTGSFVAAEVLARWYDEGRVLTPSSLSSPPY

WGLVDMEMARFIQDNLHYCLDLYPALFLNVSEQTLQSDVIFKAWCRVVRDIAKNHSSR

LVIEITEGIQDASLASRWEALTEIGVELALDDYGDKNSSLDRLSRYDWHYCKFDARRL

RSLEDYPAILHCRRKGIQLIAEQVESFPLGESAKLLGLSWQQGFYHGKPAVMEKNLNY

VKALP"

CDS 103789..104796

/codon_start=1

/product="IncF plasmid conjugative transfer pilus assembly

protein TraU"

/translation="MMQKILRIMAVSAVFWVGSVSADPGCQNAEVIGGKLITDICWSC

IFPIKVAGVPISGGGGSFPSEAVSNPLCMCEDNLGVPRPGVTTSMWEPARLVEFQRVP

GCSSVLNGVRFPFDRTNQGHHGMGDMDGGDGSFMHYHYYAFPLLVMLDLFIKQTCNAD

GYMDLDIMYMSELDPTWNNDELAFFTNPEAAAVANPIAAAACTADAVSSTAGKPLKQL

FWCAGSWGTLYPFSGNQNGGKGVIRDSSLLSTRVLAALHRRGLAWKTMGSEAMCRGVI

SPTLPKTQYKFTLLHPVPETNSSHVIGESTLTWGLARTIPAIGQDPIYTIWRWNDCCN

N"

CDS 104912..107707

/codon_start=1

/product="IncF plasmid conjugative transfer protein TraN"

/translation="MRSHNYFMKAVASLLTVTMSALPIHSYANGSQDQDITAVGKEAQ

AFGQNLSNSFKSSSGTVQDGTISMPTLKDGQFQMNGGSQINVNDLFPGTSGTNNKPDS

YYFPDANKPDVGGLQGIYDSGDDMDSVGNNAKGSLWSDANSANPSISGAAYKVLLDAS

NRSRPDFSNDPVLNLSKKTYEDMDLIAGGFGDCSAETTINQNTINAHIPEYERCQRVV

DQSADCEVVHDYDASVVKHYDGPYNLKSCGEGCTELWIGRVGDNYWSGNCKIYEEYTR

VQVSNPDAIVSATLEYAKWDDYMQVWVGKSGQETKVWSGPDGNFPPETAGRCELSTSW

ERNPNVDVTPYFKNVKDGDVVTFKIRVSVSGEGEGFGRIKLRYDPSKAITKDEWAPQS

CMDSAKGVVDGFAEGEITCIDDPTDATGCTVINGIKVCESQLKPSPLPGIPKLCKKVR

VKADYDFYKGQMDCWTDPQGETHCPVNTGGNLDSCQKYEENPQCGFISSKCVDGAQGS

SGTCYVHEDTYDCGTDVSVPTLEKETEYQCGGPIRCMGDDCLDLTKTQSTDFARATAL

LNAAQFMTQDMSCTGQDGDDNPTGDENVICSAFAGEAGECKIAVGGVSDCCEKPTNIS

LADYLNLIMAVPKLDGAVMGLTDGNALKGAYQVLREPALQGWTEVTKPFTSYIENVSG

AVDSFFQPVEQFVDQLIDQLKEQVKEVMMDVMKSAGQDAATEQAAAAASEQAAEAMME

TATTWLSTAMTIYTVYVVAMVMIQMIYKCEEEEFTMNAKRALKNCTYVGSYCKSKVLG

ACIEKREAYCCFNSPLSRIIQEQVRPQLGQNFGDPKNPQCEGIPLDKIAEIDWSKINL

DEWLGILQQNGKFPDPASINLDSLTGAGNDFNIDGTRKNAQERALERLEGIDIDAKRK

EATNSIDPQTGAPTGGG"

CDS complement(107746..108606)

/codon_start=1

/product="hypothetical protein"

/translation="MKKALITTVALAVAALSGCASTSSYEPTAYKTNHSRAYNIAEAG

GLVTGIQDASVPRDKLERLTDTKTFGAAYVLSGYMSPQLGMTDWQGGLVNLANWAIGP

KQHGARNSLIAWMPANEAVSPADAQAKFLSHVKVSIESALTDLGANFTLLYDKDGRLT

YQFYKNEWGCPTWVNGQSKVADMCSVKVKIVEPRLGSAPNFVSGVQGTAYAFTSGHET

AYNFVNVGNGAASHAPQQAIYAAISKQLPAWAYLYLAPKSVELDNGEKVAFPYLLDQG

KAELFVYPEA"

CDS complement(108729..109370)

/codon_start=1

/product="hypothetical protein"

/translation="MAEAQVALDTNNDRSNHYSRPVFKQVLKVNSLQAQRVMERSFER

VSNSLFSIDVILRIIGEQDEIDQVETVILEHISKVSEDLDKATAQLNKLMEDNGIDMM

PGYTNPNEYTIEINSPQVAQFAHLIRKLDTLMGIVDTLWLNTVLTSKQRTDATYQWQQ

RLIKLAGRIIGIEKRARISAHSKGKEGEVAEAAPESATGDKEIADEAEKTKAA"

CDS 109665..109985

/codon_start=1

/product="hypothetical protein"

/translation="MHIITFIVVLLASLGALYVGAKAFAFSFIVLAKVFQRIGLFLWN

KFAVPAMRSVQAVIKERAAKAEQAKAVVEPEVTQVQEVAKQEPDWDYLEIPTYLRKGK

ELVW"

CDS 110289..110474

/codon_start=1

/product="hypothetical protein"

/translation="MQQNLHVYDDHAGIIYLADGREVKFDPKLYSSSYQAHSEAVKWA

KETGVIGQNDDVVMFVH"

CDS complement(110496..110612)

/codon_start=1

/product="hypothetical protein"

/translation="MLFVFGDQLSHFSLSEENKICLLMVFTMSKKTFKERGE"

CDS 110694..111662

/codon_start=1

/product="Aerobic cobaltochelatase CobS subunit"

/translation="MSQYSQFSVSKVFGMPSIPEKVTAIGYADGSNPFIPATDTNYVF

RKEFLREVLAYLKEPGGDALFVTGPTGSGKTSGITEIAGRLNWPVQQITAHGRMELTD

LIGHHALVAEKPGQPPVMKFMYGPLAVAMREGHLLLINEVDLADPAELAGLNDVLEGR

PLVIAQNGGEIIKPHPMFRVVVTGNSTGSGDASGLYQGVMMQNLAAMDRYRFTKVGYA

DEEAELSILGRATPKLPENVRKGMVRIANQVRKLFLGENGEDGQISVTMSTRTLVRWA

KLSLAFRGAPNALEYALDQALLIRAAKEEREAILRVAKDVFGDQWR"

CDS join(111673..112020,112026..>112580)

/codon_start=1

/product="hypothetical protein"

/translation="MKKNDCLCRRYTAKEWGNDETTIEVFIGYKLLREPSSSEPGQFT

MVELRRTVTDGKAENWSETKLEGPFEANGPDTIPMSYKDKESQYVSQFLSQGYTFLDE

VLVNAETQTVLEGGXXSAGQTASLGSLNWLLSPPSELPPGDINLFKGFVAGVFAKGAG

LIGFEVARSEGSNDLLPSVLMRTDSGYELGVSTGLGENTIHPATLEGAGELRPEHGHK

PLLMLVYLQQRFADDFSNVEKPLVAFCDEQGDTFDYERFDSLKPLIERFGFSYDEVRA

DAERLGLVSELIRLAEIDAEQEDHFF"

CDS 112644..113171

/codon_start=1

/product="Single-stranded DNA-binding protein"

/translation="MSKGVNKVILVGNLGSDPEIRYMPSGTAVANFNVATTDTWRDKQ

SGEQREHTEWHRIVLKGRLAEVAGEYLKKGSQVYLEGSNRTRKWTDSQQIERYTTEVH

CVEMQMLGGRGNAPQDNSQRAAPQKGQRTGAGTQSAPVQQSAPQGGMGGGYGPAPDGW

DDDIPFMRLHHLAGG"

CDS 113266..114255

/codon_start=1

/product="Recombination protein BET"

/translation="MSDNKSLVTRIASRFGVDTRKFYETLKATAFKQRDGSAPTDEQM

MTLLIVAEQYGLNPFTREIYAFPDKQNGIIPVVGVDGWSRIINEHPQYDGVEFVYSDK

MVRMQGAKVDCPEWIECVIYRKDRSRPIRIKEFIDEVYREPFQGQGRNGAYTVDGPWQ

THTKRQLRHKSLIQCSRVAFGFSGIYDQDEAERIREMEQASAINPAIANLPSPSQVQS

QEPLAIEHKELDPILTKLANRAIAENAWSAAHEYVKGRYEGSELQYATQFLRDKEMDQ

MEPPKPDYQEAHEQESAAGGSANAEPGAEEMPPLSDEDMIPVTEEEGAEGSYY"

CDS 114318..115328

/codon_start=1

/product="hypothetical protein"

/translation="MKIVNLSQREEDWLDWRRQGVTATDAAILLNRSPYKTRWRLWAE

KTGYAREVDLSLNPLVRRGIENEDAARRAFEEKYDDMLLPACVESVQYPLMRASLDGL

RDNGEPVELKSPSATVWEDVCAEKANSKAYQLYYPQVQHQLLVTGAKQGWLVFYFEGQ

IQEFPILRDEAMIQEILAEAKKFWQQVVDKKEPDKDPERDLYIPQGEEVNRWIAAAEE

YRLYDAEIQELKQRLSELQERQKPHLDTMKSLMGEYFHADYCGVMVTRYKAAGRVDYK

KLLADKASGVKPEDVDQYREKSSERCRVTVTGSVKPRYIVDEDVLAPLDDLPEEVETF

YW"

CDS 115528..116805

/codon_start=1

/product="Cobalamine biosynthesis protein"

/translation="MTKVNHLQSLCVIHVDFDIWSGQTRLSASDLKLGEGGEIPPEKV

AQLGSKKICDPAKLKGFHRLKTETRRLLLKFGMPFMNGFAVPVSKTDEICNKLNDINF

QFNQLKQDFIKGYNKAVDEWCQENPEYERAIRAGALPKETVEERIGFEYQVFMIQPVN

EDEANAKRLNRKVERLGDDLISEVVQEANKFYMERLAGRDQCAVTTRQTLRNIRDKVD

GLSFLNSAFNPLVKLLDQTLRGYEQHADGRNIVAPFFYQVVAAVLIMSERDRIEQYAN

GSITVEGMANDIGGSGAQMGDRSKDEKAEQKSDKAGELIPATEGGETKQQQVGGTESV

QSEQTNSGGNSVDLDEDIDNFFKSFAERGEGESEDESNAGDVVREERVDVEDELVLPE

ETPVEQEPVQEETTEEPLNQELPKTDDDGDYFF"

CDS 116890..118686

/codon_start=1

/product="hypothetical protein"

/translation="MSKKRTIYSALPIVAAAYGEKLGVKVAIGNDDAYTDGKTIVVPN

IPDDYPHMDAVWGYLAHEAAHVRFTDFGVERRRGLHAELSNVLEDCRIERAMMELFPG

TSQTLNEVARYMAQAGHYEHVTDKEAPASILTGFCLYWLQTKAVGQSVLQPYLDSATP

VFERVFPQGVVVRLNALLRKAVNTKSTAEVTSLADQIIKMIEEEKEKEEQKPQNGQDG

NNQQNAGGNQPQNSQGGSGNDQNQGPDANGGDQQGKDQKQDDANGKSDPKGQGDQGKS

DTDGGSKAGQSQAGGNSDAAKQDAAKMLQQVLNAGAGDLRGDAHDALKAELNRVAQDK

GDSSYMTVRSAVNTQDNPAVGKSLVGDVKSTTSKIRTQLYGLVQASQRVAHRNQRSGK

RVDARKLHRVVTGDTRVFLKPEAKKRPNTAVHILVDMSSSMAYKAANGKERQDIAREA

SLAISMALEAIPGVNPAVTFFGGNRNQPVFSVVKHGDTVQNRAGRFGFKATGGTPMAE

AMWYAAFELTKTREERKMLIVVTDGQPQSAPACRSVIDLCERSDVEVIGIGVETTAVS

GLFQKNIVIDDAAALQRTLFKLMERSLTAFAA"

CDS 118895..119083

/codon_start=1

/product="hypothetical protein"

/translation="MGYTGRRQFHRLGQAIDWAKSSVGDSWSNKRFHKPVGLDVLLAC

TASKVPEHLVEELKRRGS"

CDS 119348..119782

/codon_start=1

/product="hypothetical protein"

/translation="MSHLNNLKSVMISLAAEHKLPEIYQDDITTDVESLDRFDGLRLV

WLLRSCGSVLVPAEVGVNPIYITHWLWSNHGQQVVPFSVDTRTGLIEKIDFEQAEKLI

MQMPCNLSSLQNKEYLVDQVNRVLQRGCEMRIWGSWPKTAIT"

CDS 119837..120040

/codon_start=1

/product="hypothetical protein"

/translation="MSLDKLSRLVDQQRKIQDEIDNEIILAVKEVLATNSVGLARELV

GGVPQDHPFHPFLLAIAKQLDPR"

CDS 120112..120717

/codon_start=1

/product="Nucleotidase YfbR, HD superfamily"

/translation="MSNSNFGFLALALRQRLIKRWSLMHSVQPESVLEHSAVVTLLSY

LAGNIAIQQGKSVDLAVMLAHASLHDAAEVLCSDVVTPVKKANAVLQREFERLEKAAE

DKLIQTLPEELQDAVAIAFAPGGYEQSLVKACDTYSAYIKCKLEVAAGNGLEFQDALS

KMERVVAQVKSDFPEIDALDKWFGNGLGHSVDKLLAGGNDD"

CDS 120710..120979

/codon_start=1

/product="hypothetical protein"

/translation="MTNPIPGDIKIKDFGRDRKFRSVDELQSTLSEQYKGQHVSIVYP

AKPSGLLRTVFVSVDDAGGVNRTYGDQSPVDFSAIKDDLYVPSDL"

CDS 120993..121211

/codon_start=1

/product="hypothetical protein"

/translation="MVVIIVNTGHYEFIGLGETHGQATEGLLKRWDEHCERNPDAESG

YMQELIEEGSAQVVEMEPGSAVIYGLDG"

CDS 121285..121842

/codon_start=1

/product="conserved hypothetical protein"

/translation="MSNFEQALERTDGKTLILSNGSKWAGQDPDSIQTLLDVLGDNVL

DPMFEQYHCYRPYPFEPMVRTGRNGEMFQPWLGAACFFGNFLTVSHVFNIITKDDGVV

EALTEAIRKNMATEQYQQNAYERYAGWFYAETSEGLRLVSPSEAADIRAGAVSKLRYP

RNFEVMKTAVLKGPRFDTELSRKAS"

CDS 121917..122768

/codon_start=1

/product="hypothetical protein"

/translation="MIKQHFQNELVKCGYPDDLTIEYSLGYCQGDGVAFYGDLSVDDV

KALMNRLFSTEPGQVDAVSRVKNLMAQKDIENMLSVLREYGSCDLSITRNSHGHHYSH

WNCMNIDDNVDFTGIFPDDDSMIGTGIEGINQDMVERWQDLWERFVLELADDVKSLSK

KLEADGYSLIEASPCEDEVVWERATENYLVRVTELPERDFDMGHWDDEVRDQTICSIL

EGKERVLGLRVEVLSRENEIVLGEESLHGLTVASDDKSYAGYRRELLRGAIQQTRDFF

SRHLKAA"

CDS 123227..123613

/codon_start=1

/product="hypothetical protein"

/translation="MNQVAKLDLAQIRQQAINDGLLVDQSSIGKQAGFLTNVAVTPAI

VDGVFGADGKHSVEDFLFMFLQLCVAQTKVAFTDNKNWGKIRLYYPMPTVDGFFKPTE

VVIKSDPVTADVTIMMASEEGAHLCL"

CDS 123791..125518

/codon_start=1

/product="putative DNA primerase"

/translation="MSYYKADTVREAANGNWLFILAALAPHLEPALRKPGRHVSCPIH

GGKDGFRLFKDAHLTGGGVCNTCGANHDGFELLMWLNNWDFKQCLSEVGDYLGVEKEQ

PQYQQAAAPTRAPVQAKAPVQQEPMKVNNKVLDSKNRKKSIAGTLIAHGKAPYEHNED

NELSYFAFIRDKSGLERTIWGVDLERAIGESEAKYGDEIVMTNLGREPVTVVVEVKDE

QGNVVREQPMQTHRNTWLVERRGATVTQFRARSNGGVEPVSHHVESAPVVNRKVETPA

PQVQPAATQPEEQSSENKPKVVPMFREQPKPWLLELQEEMEKRMERERAYSARLREKI

EKVWNECLPFSSHVTEPMRLYFKNRELLFKVDEVEKTDCLRFNPAMAYYDEDGNEVGK

FPAIVCAIRDVEGNLVTLHRTYLTQNGKKAKVGNAKKMMPIPDGLDVNGAAIRLGEPT

EGILGVAEGLETALSAYRVTQIPVWSTVNATLMESFEVPEGVHTVLIWADKDKSVTGE

KSANVLKAKLEKRGIRVYVLLPKLPIPPRAKGIDWNDVLMSQGSLGFPNARYLRDFIA

RRRAEYGRH"

CDS 125640..125783

/codon_start=1

/product="hypothetical protein"

/translation="MHGQRMMVTASFARREPIQVTGPFADEATKIINSMKMNKAKPTA

LSA"

CDS 125856..126077

/codon_start=1

/product="hypothetical protein"

/translation="MKKFLRIKTWFVRLFSPDKKTLGAIGEDLRKVAVTAIGVGIVGV

VSENGIYGHSRFCNTDFDDKLACLNLSGV"

CDS 126118..126240

/codon_start=1

/product="hypothetical protein"

/translation="MNLQKIYGFNEPFRFTGSSTGRWAVSIINISIRKTRSVIL"

CDS complement(126259..127263)

/codon_start=1

/product="Mobile element protein"

/translation="MENIALIGIDLGKNSFHIHCQDRRGKAVYRKKFTRPKLIEFLAT

CPATTIAMEACGGSHFMARKLEELGHFPKLISPQFVRPFVKSNKNDFVDAEAICEAAS

RPSMRFVQPRTESQQAMRALHRVRESLVQDKVKTTNQMHAFLLEFGISVPRGAAVISR

LSTLLEDNSLPLYLSQLLLKLQQHYHYLVEQIKDLESQLKRKLDEDEIGQRLLSIPCV

GTLTASTISTEIGDGKQYASSRDFAAATGLVPRQYSTGGRTTLLGISKRGNKKIRTLL

VQCARVFIQKLEHQSGKLADWVRDLLCRKSNFVVTCALANKLARIAWALTARQQTYVA

"

CDS complement(127342..130314)

/codon_start=1

/product="Mobile element protein"

/translation="MPRRSILSATERESLLALPDAKDELIRHYTFNETDLSVIRQRRG

AANRLGFAVQLCYLRFPGTFLGVDEPPFPPLLRMVAAQLKMPVESWSEYGQREQTRRE

HLVELQTVFGFKPFTMSHYRQAVHTLTELALQTDKGIVLASALVENLRRQSIILPAMN

AIERASAEAITRANRRIYAALTDSLLSPHRQRLDELLKRKDGSKVTWLAWLRQSPAKP

NSRHMLEHIERLKSWQALDLPAGIERQVHQNRLLKIAREGGQMTPADLAKFEVQRRYA

TLVALAIEGMATVTDEIIDLHDRIIGKLFNAAKNKHQQQFQASGKAINDKVRMYGRIG

QALIEAKQSGSDPFAAIEAVMPWDTFAASVTEAQTLARPADFDFLHHIGESYATLRRY

APQFLGVLKLRAAPAAKGVLDAIDMLRGMNSDSARKVPADAPTAFIKPRWAKLVLTDD

GIDRRYYELCALSELKNALRSGDVWVQGSRQFKDFDEYLVPVEKFATLKLASELPLAV

ATDCDQYLHDRLELLEAQLATVNRMAAANDLPDAIITTASGLKITPLDAAVPDAAQAM

IDQTAMLLPHLKITELLMEVDEWTGFTRHFTHLKTSDTAKDKTLLLTTILADAINLGL

TKMAESCPGTTYAKLSWLQAWHIRDETYSTALAELVNAQFRQPFAGNWGDGTTSSSDG

QNFRTGSKAESTGHINPKYGSSPGRTFYTHISDQYAPFSAKVVNVGIRDSTYVLDGLL

YHESDLRIEEHYTDTAGFTDHVFGLMHLLGFRFAPRIRDLGETKLFIPKGDAAYDALK

PMISSDRLNIKQIRAHWDEILRLATSIKQGTVTASLMLRKLGSYPRQNGLAVALRELG

RIERTLFILDWLQSVELRRRVHAGLNKGEARNALARAVFFYRLGEIRDRSFEQQRYRA

SGLNLVTAAIVLWNTVYLERATSALRGNGTALDDTLLQYLSPLGWEHINLTGDYLWRS

SAKVGAGKFRPLRPLPPA"

CDS complement(130317..130874)

/codon_start=1

/product="Mobile element protein"

/translation="MQGQRIGYVRVSSFDQNPERQLEGVQVARVFTDKASGKDTQRPE

LERLLAFVREGDTVVVHSMDRLARNLDDLRRIVQGLTQRGVRMEFVKEGLKFTGEDSP

MANLMLSVMGAFAEFERALIRERQREGIVLAKQRGAYRGRKKSLNSEQIAELKRRVAA

GDQKTLVARDFGISRETLYQYLRED"

CDS 132355..132978

/codon_start=1

/product="6'-N-acetyltransferase"

/translation="MLAVKIKPFTKPILIMKNTIHSIVTNSNDSVTLRLMTEHDLAML

YEWLNRSHIVEWWGGEEARPTLADVQEQYLPSVLAQESVTPYIAMLNGEPIGYAQSYV

ALGSGDGWWEEETDPGVRGIDQLLANASQLGKGLGTKLVRALVELLFNDPEVTKIQTD

PSPSNLRAIRCYEKAGFERQGTVTTPDGPAVYMVQTRQAFERTRSDA"

CDS 133147..133494

/codon_start=1

/product="Ethidium bromide-methyl viologen resistance

protein EmrE"

/translation="MKGWLFLVIAIVGEVIATSALKSSEGFTKLAPSAVVIIGYGIAF

YFLSLVLKSIPVGVAYAVWSGLGVVIITAIAWLLHGQKLDAWGFVGMGLIIAAFLLAR

SPSWKSLRRPTPW"

CDS 133488..134327

/codon_start=1

/product="Dihydropteroate synthase"

/translation="MVTVFGILNLTEDSFFDESRRLDPAGAVTAAIEMLRVGSDVVDV

GPAASHPDARPVSPADEIRRIAPLLDALSDQMHRVSIDSFQPETQRYALKRGVGYLND

IQGFPDPALYPDIAEADCRLVVMHSAQRDGIATRTGHLRPEDALDEIVRFFEARVSAL

RRSGVAADRLILDPGMGFFLSPAPETSLHVLSNLQKLKSALGLPLLVSVSRKSFLGAT

VGLPVKDLGPASLAAELHAIGNGADYVRTHAPGDLRSAITFSETLAKFRSRDARDRGL

DHA"

CDS complement(134780..135079)

/codon_start=1

/product="hypothetical protein"

/translation="MQQGTLFTRRTVERHFRKHQARCPPEYREILIERILAKRWTEAS

LGKVVGIVASTFARHQLTDYDRLLAISGMARAEARLIVSREVSDILESWRSTALP"

CDS complement(135193..135354)

/codon_start=1

/product="hypothetical protein"

/translation="MNSQADNFDDDQEATAEGIADIEAGRTISHEAVKAWLLSWGTPN

ELPPPKVGD"

CDS 135453..136247

/codon_start=1

/product="Cytosine-specific DNA methyltransferase"

/translation="MRQREWLGGGPQDFSRRLVGFRRDHSVRRRCGGTPSVRPSQTLR

SAPLAFLLPIAIAGTSQLSKLRDAQCCGGTSTSAFLQLRAGGHRNSHANAGVVPAIAF

AQNNRGEVRFESGHGQVACTVLSNGKPGYGVPMVACVSLRGREQGLAAELGGSVAATL

RTSGGGADKPHVLAPDFEAHFRYDWNDPGPGDWSHWRVRRLMPTECERLQGMPDDYTL

IPYRGKPAADAPRYKAIGNSMAVPCMAWLGQRLVQCLHKTGSTASD"

CDS complement(136378..136551)

/codon_start=1

/product="hypothetical protein"

/translation="MKEFNVSSKGNSDKNDGKNFKILLKSTGWRHAPDGTWRPSRGLE

KRGIDAPVGSVRR"

CDS 136575..137225

/codon_start=1

/product="Endonuclease III"

/translation="MYNPTVNKKTRAKYVLEEMEKLFPDSSSELRNWETDFQFLLCII

LSAQTTDLQVNKVTHNLFAKYPDPASLSEAEVGEVEKILSSINYYRTKSKNIVNAAKV

VKTRFHGRVPRSVEKLIEIPGVGLKTANVYLNSMYQANQGVGADTHVMRVSRRLGFTD

SRDPRKVAIALQKLYPKKDWYRVTALFVLYGRYYCKARVKPENSKCIFKEFCTHCR"

CDS complement(137750..137941)

/codon_start=1

/product="Mobile element protein"

/translation="MLAWLPFPSDVLPAPLLKAWCVTAKALPDISAISALIAVKHGNY

SSLTPPLSPVRTRKSLIWP"

CDS complement(137750..137941)

/codon_start=1

/translation="MLAWLPFPSDVLPAPLLKAWCVTAKALPDISAISALIAVKHGNY

SSLTPPLSPVRTRKSLIWP"

CDS 139511..139906

/codon_start=1

/product="Mobile element protein"

/translation="MIVCAEMDEQWGYVGAKSRQRWLFYAYDRIRRTVVAHVFGERTL

ATLERLLSLLSAFEVVVWMTDGWPLYESRLKGKLHVNSKRYTQRIERHNLNLRQHLAR

LGRKSLSFSKSVELHDKVIGHYLNIKHYQ"

CDS 140060..140464

/codon_start=1

/product="Mobile element protein"

/translation="MAYDFKEAFFCIYDEPDKQSAQNAFEAWKNSLPPYGMEPFKKLV

KTVHNHYDDIFAYWDAPFSLTNGYTEGLNGLIKMSNRLGRGYSYEIIRAKTLYSKEAR

KVGSGIRAGRGKVEYGPHIPTLLKQAEGGELD"

CDS complement(140760..141308)

/codon_start=1

/product="Silver-binding protein"

/translation="MNIQPSSGEINAAELVSVMELKTPVVLPRTSLIQKWRVIMKNIV

LASLLGFGLISSAWATETVNIHDRVNNAQAPAHQMQSAEAPVGIQGTAPRMTGMDQHE

QAIIAHETMTNGSADAHQKMVESHQKMMGNNTVSTTVPSTSYAAMNEHERAAVAHEFM

NNGQSGPHQAMAEAHRRMINAG"

CDS complement(141442..142917)

/codon_start=1

/product="Osmosensitive K+ channel histidine kinase KdpD"

/translation="MHSKPSRRPFSLALRLTFFISLSTILAFIAFTWFMLHSVEKHFA

EQDVSDLQQISTTLSRILQSPADPDEKKVSKIKESIASYRNVALLLLNPRGEVLFSSA

QGAALRPAVNSADFSEHSRARDVFLWTVEDPAGPMDTGSEMKMETYRIIASSGQAIFQ

GKQQNYVMLTGLSINFHLHYLDALKKNLIAIAVVISLLIVLIIRIAVRQGHLPLRNVS

NAIKNITSENLDARLEPTRVPIELEQLVISFNHMIGKIEDVFTRQANFSADIAHEIRT

PITNLVTQTEIALSQDRTQRELEDVLYSSLEEYNRMTKMVSDMLFLAQADNNQLIPDR

VMFDLRAEVMKVFEFFEAWAEERNITLKFNGMPCLVEGDPQMFRRAINNLLSNALRYT

PEGQAITVSIREQESFFDLVIENPGKPIPEEHLSRLFDRFYRVDPSRQRKGEGSGIGL

AIVKSIVEAHHGRVQVESDVRSTRFILSVPRLEKMIPETQC"

CDS complement(142910..143590)

/codon_start=1

/product="Copper-sensing two-component system response

regulator CusR"

/translation="MKILIVEDEIKTGEYLSKGLTEAGFVVDHADNGLTGYHLAMTAE

YDLVILDIMLPDVNGWDIIRMLRSAGKGMPVLLLTALGTIEHRVKGLELGADDYLVKP

FAFAELLARVRTLLRRGNTMITESQLKVADLSVDLVSRKVSRAGNRIVLTSKEFSLLE

FFIRHQGEVLPRSLIASQVWDMNFDSDTNAIDVAVKRLRAKIDNDYGTKLIQTVRGVG

YMLEIPDA"

CDS 143701..143913

/codon_start=1

/product="Mobile element protein"

/translation="MYDGVFEVLQWLLFLSAVPPVQLLTGWCVTAKAPPDISAISALT

AVKHGNCSSLTPLLNPVRTRKSLIWP"

CDS 143701..143913

/codon_start=1

/translation="MYDGVFEVLQWLLFLSAVPPVQLLTGWCVTAKAPPDISAISALT

AVKHGNCSSLTPLLNPVRTRKSLIWP"

CDS 143924..144427

/codon_start=1

/product="Mobile element protein"

/translation="MPGNRPHYGRWPQHDFPPFKKLRPQSVTSRIQPGSDVIVCAEMD

EQWGYVGAKSRQRWLFYAYDRLRKTVVAHVFGERTMATLGRLMSLLSPFDVVIWMTDG

WPLYESRLKGKLHVISKRYTQRIERHNLNLRQHLARLGRKSLSFSKSVELHDKVIGHY

LNIKHYQ"

CDS complement(144438..144581)

/codon_start=1

/product="hypothetical protein"

/translation="MLSNFNLNITLLVQADLVNGSNEIQSIDRDTLPNALCLPGDRIM

TMW"

CDS 144556..145941

/codon_start=1

/product="Cation efflux system protein CusC precursor"

/translation="MFKLKLLSISTIFILAGCVSLAPEYQRPPAPVPQQFSLSKNSLT

PAVNSYQDTGWRNFFVDPQVSRLIGEALNNNRDLRMAALKVEEARAQFNVTDADRYPQ

LNASSGITYNGGLKGDKPTTQEYDAGLELSYELDFFGKLKNMSEADRQNYFASEEARR

AVHILLVSNVSQSYFSQQLAYEQLRIARETLKNYEQSYAFVEQQLVTGSTNVLALEQA

RGQIESTRAEIAKREGDLAQANNALQLVLGTYRALPSEKGMKGGEIAPVKLPPNLSSQ

ILLQRPDIMEAEYQLKAADANIGAARAAFFPSITLTSGLSASSTELSSLFTSGSGMWN

FIPKIEIPIFNAGRNKANLKLAEIRQQQSVVNYEQKIQSAFKDVSDTLALRDSLSQQL

ESQQRYLDSLQITLQRARGLYASGAVSYIEVLDAERSLFATQQTILDLTYSRQVNEIN

LFTALGGGWVE"

CDS 145970..146323

/codon_start=1

/product="Cation efflux system protein CusF precursor"

/translation="MRNSLKAVLFGAFSVMFSAGLHAETHQHGDMNAASDASVQQVIK

GTGVVKDIDMNSKKITISHEAIPAVGWPAMTMRFTFVNADDAINALKTGNHVDFSFIQ

QGNISLLKSINVTQS"

CDS 146437..147729

/codon_start=1

/product="Cobalt/zinc/cadmium efflux RND transporter,

membrane fusion protein, CzcB family"

/translation="MASLKIKYAAIIISSLIAGGLISVTAWQYVNSSQKTVQTEQKAP

ERKVLFWYDPMKPDTKFDKPGKSPFMDMDLVPKYADESGDKSSGGIRIDPTQVQNLGL

KTQKVTRGMLNYSQTIPANVSYNEYQFVIVQARSDGFVEKVYPLTIGDHVKKGTPLID

ITIPEWVEAQSEFLLLSGTGGTSTQIKGVLERLRLAGMPEEDIQRLRSTRTIQTRFTI

KAPIDGVITAFDLRTGMNISKDKVVAQIQGMDPVWISAAVPESIAYLLKDTSQFEISV

PAYPDKTFHVEKWNILPSVDQTTRTLQVRLQVSNKDEFLKPGMNAYLKLNTKSQEMLL

IPSQAVIDTGKEQRVITVDDEGKFVPKQIHVLHESQQQSGIGSGLNEGDTVVVSGLFL

IDSEANITGALERMRHPEKTESSMPAMSDQPVNMHSGH"

CDS 147740..150886

/codon_start=1

/product="Cobalt-zinc-cadmium resistance protein CzcA;

Cation efflux system protein CusA"

/translation="MIEWIIRRSVANRFLVMMGALFLSIWGTWTIINTPVDALPDLSD

VQVIIKTSYPGQAPQIVENQVTYPLTTTMLSVPGAKTVRGFSQFGDSYVYVIFEDGTD

LYWARSRVLEYLNQVQGKLPAGVSSEIGPDATGVGWIFEYALVDRNGKHDLSELRSLQ

DWFLKFELKTIPNVAEVASVGGVVKQYQIQVNPVKLSQYGISLPEVKQALESSNQEAG

GSSVEMAEAEYMVRASGYLQSIDDFNNIVLKTGENGVPVYLRDVARVQTGPEMRRGIA

ELNGQGEVAGGVVILRSGKNARDVITAVRDKLETLKASLPEGVEIVTTYDRSQLIDRA

IDNLSYKLLEEFIVVAVVCALFLWHVRSALVAIISLPLGLCIAFIVMHFQGLNANIMS

LGGIAIAVGAMVDAAIVMIENAHKRLEEWDHQHPGEQIDNATRWKVITDASVEVGPAL

FISLLIITLSFIPIFTLEGQEGRLFGPLAFTKTYSMAGAAALAIIVIPILMGFWIRGK

IPAETSNPLNRVLIKAYHPLLLRVLHWPKTTLLVAALSIFTVIWPLSQVGGEFLPKIN

EGDLLYMPSTLPGVSPAEAAALLQTTDKLIKSVPEVASVFGKTGKAETATDSAPLEMV

ETTIQLKPEDQWRPGMTIDKIIDELDRTVRLPGLANLWVPPIRNRIDMLSTGIKSPIG

IKVSGTVLSDIDATAQSIEAVAKTVPGVVSVLAERLEGGRYIDIDINREKASRYGMTV

GDVQLFVSSAIGGAMVGETVEGVARYPINIRYPQDYRNSPQALREMPILTPMKQQITL

GDVADIKVVSGPTMLKTENARPASWIYVDARGRDMVSVVNDIKTAISEKVKLRPGTSV

AFSGQFELLEHANKKLKLMVPMTVMIIFILLYLAFRRADEALLILMSLPFALVGGIWF

LYWQGFHMSVATGTGFIALAGVAAEFGVVMLMYLRHAIEAHPELSRKETFTPEGLDEA

LYHGAVLRVRPKAMTVAVIIAGLLPILWGTGAGSEVMSRIAAPMIGGMITAPLLSLFI

IPAAYKLIWLRRHKKSVS"

CDS 150973..151413

/codon_start=1

/product="CopG protein"

/translation="MKKVVLMALALGLSLPAMASEKVIDMYKSENCGCCSLWGKAMEK

DGFEVRTHVMNDQALSALKEKHAIPAGLRSCHTAVAGNLIIEGHVPATTIHKAMQSGS

GIYGLATPGMPAGSPGMEMGARKEAYDVIAFSPDGSKKVFQRIE"

CDS complement(151594..151842)

/codon_start=1

/product="3-dehydroquinate synthase"

/translation="MRLFYLIRADFVMIVVMVMGMFTGIFGKITIGMGFKFTLTAGGA

EIKLVVLVSNAAVRPVGQDGHPAHGISHLMQYVALVRG"

CDS 151939..153987

/codon_start=1

/product="Lead, cadmium, zinc and mercury transporting

ATPase"

/translation="MALEPLVATASTGPSDELHDMTRRFWLGLLLAFPVLVLEMGSHL

FPDLRNTVPPQYNTWLQLLLASPVVLWCGWPFFARAGMSLRNRSLNMFTLVAMGTGVA

WVYSVIATVFPSWFPASFRNMDGLVAVYFEAAAVITVLVLLGQVLELRAREQTSGAIT

ALLNLAPKTARRLDHDGHETDINAEDVLPGDKLRIRPGESIPVDGIVIEGKTTVDESM

VTGESMPVTKTKGDPVIGGTINQTGSLIIRAEKVGDETMLSRIVQMVADAQRSRAPIQ

RMADSVSGWFVPLVILIAVVAFLIWSVWGPEPRMAHGLIAAVSVLIIACPCALGLATP

MSIMVGVGKGAQAGVLIKNAEALERLEKVDTLVVDKTGTLTEGSPTVTGIISLSPGGE

ISLLRVTAAVEKGSQHPLGMAVVRAAHEKGIVIPAVSNFNAPSGKGVSGDVEGQRVVI

GNELAMQENSIVIDNQKAVADTLRMEGATVIYVATDGNLAGLIAISDPVKATTPDALK

ALRQAGIRIVMLTGDNQLTAEAVARKLGIDEVEAGILPDGKKAVITRLKASGHVVAMA

GDGVNDAPALAAADVGIAMGTGTDVAIESAGVTLLKGDLMILNRARHLSEITMKNIRQ

NLFFAFIYNALGVPVAAGLLYPVYGILLSPVIAAAAMALSSVSVIANALRLKSVRLGK

"

CDS 154028..154225

/codon_start=1

/product="hypothetical protein"

/translation="MKSTTYALIAVAAIAAFALLREHWSHVAGYWPYLLLLVCPLMHL

FHGHGGHGDHQHQGSENDKKN"

CDS complement(154259..154996)

/codon_start=1

/product="Cell wall endopeptidase, family M23/M37"

/translation="MYSTDVVKENAYLSATRSGLESNEIATLQRSLPSRFNLRHLKKN

ESLKLVLQKKAGKSRVVAYKFTSGSFNYTAYRISDKKFYNLSDTSGKGSLDYPLPATA

RLSSPFNPARLNPVSGKVSPHNGIDYSMPMNTKIVSVIDGKITRAEYNSTMGYFVEVT

GKAGVKTRYLHLNKILVTKGARVTRGDAIALSGNSGRSSGPHLHYELVINNNPVNSLA

FRAAAPADNKLEQHAFAHARDYERYLE"

CDS complement(155285..155734)

/codon_start=1

/product="Copper-binding protein PcoE"

/translation="MNILITTTAFTALFCGAAFAQSSDIAHEAHRFVNNASAVSHVNS

STHENLPDRVNKNNTPSFSEMNEHERAIVAHSFMNNSASYAHQKMIEEHKKMLSGSDA

NSKTSSSSFNELNAGEKAALVHEQVNNAGAEAHQTQARKLRGLYSTR"

CDS 155968..157785

/codon_start=1

/product="Multicopper oxidase"

/translation="MLLKTSRRTFLKGLTLSGVAGSLGVWSFNARSSLSLPVAASLQG

TQFDLTIGETAVNITGSERQAKTINGGLPGPVLRWKEGDTITLKVKNRLNEQTSIHWH

GIILPANMDGVPGLSFMGIEPDDTYVYTFKVKQNGTYWYHSHSGLQEQEGVYGAIIID

AREPEPFAYDREHVVMLSDWTDENPHSLLKKLKKQSDYYNFNKPTVGSFFRDVNTRGL

SATIADRKMWAEMKMNPTDLADVSGYTYTYLMNGQAPLKNWTGLFRPGEKIRLRFING

SAMTYFDIRIPGLKMTVVAADGQYVNPVTVDEFRIAVAETYDVIVEPQGEAYTIFAQS

MDRTGYARGTLATREGLSAAVPPLDPRPLLTMEDMGMGGMGHDMAGMDHSQMGGMDNS

GEMMSMDGADLPDSGTSSAPMDHSSMAGMDHSRMAGMPGMQSHPASETDNPLVDMQAM

SVSPKLNDPGIGLRNNGRKVLTYADLKSRFEDPDGREPGRTIELHLTGHMEKFAWSFN

GIKFSDAAPVLLKYGERLRITLINDTMMTHPIHLHGMWSDLEDENGNFMVRKHTIDVP

PGTKRSYRVTADALGRWAYHCHLLYHMEMGMFREVRVEE"

CDS 157785..158681

/codon_start=1

/product="Copper resistance protein B"

/translation="MRMKRNLKAIPVLVAGLFTSQLSIAAGSVSADPHAGHDMSAMQM

PADENFTEMTSMEPIVTESRTPIPPVTDADRKAAFGNLQGHAIHDSAINYLVLLDQLE

WQRSDNTNNFSWSVNSWIGGDTDRIWLKSEGERSNGETEAAEAQLLWGHAVGPWWDLV

AGVRQDFRPASARTWAAVGFQGLALYNFESEITGFVSNGGKAALRLGGEYDVLLTNRL

ILQPSYEVNFYSQDDESRGRGRGLTDTELGLRLRYEIRREFAPYIGVSWNQLYGKTSD

MAKREGEKDHQVVFLAGARIWF"

CDS 158721..159101

/codon_start=1

/product="Copper resistance protein CopC"

/translation="MSILNKAILTGGLVMGVAFSAMAHPELKSSVPQADSAVAAPEKI

QLNFSENLTVKFSGAKLTMTGMKGMSSHSPMPVAAKVAPGADPKSMVIIPREPLPAGT

YRVDWRAVSSDTHPITGNYTFTVK"

CDS 159106..160035

/codon_start=1

/product="Copper resistance protein D"

/translation="MNDLIMIVIRFLLYLDLMVIFGLPFFQIYGISGVRHETYNLTNF

RSFITFAVVTGIILTGINMLLVSNAMSGVTDLRELSIHVIEMVIEETDVGISWIVRLC

ALFTTLGALFLYTNKRVLSCLLMTMSGGVALATLAWGGHAVMHDGLHYYLHLLSDLTH

LGAAGAWTGALVAFAILLMRRNEHNAQSVIVISDSLAKFATAGTVIVVALILSALVNY

LYIAEGNLTPLFNSSWGRILLAKTALFVLMLLLAAANRFHLGPRLEVMVREGNYDRSV

ALMRNSILTEFVVAIIILGAVAWLGMLAPSQIS"

CDS 160090..160770

/codon_start=1

/product="DNA-binding heavy metal response regulator"

/translation="MQRILIVEDEQKTGRYLQQGLVEEGYQADLFNNGRDGLGAASKG

QYDLIILDVMLPFLDGWQIISALRESGHEEPVLFLTAKDNVRDKVKGLELGADDYLIK

PFDFTELVARVRTLLRRARSQAATVCTIADMTVDMVRRTVIRSGKKIHLTGKEYVLLE

LLLQRTGEVLPRSLISSLVWNMNFDSDTNVIDVAVRRLRSKIDDDFEPKLIHTVRGAG

YVLEIREE"

CDS 160767..162167

/codon_start=1

/product="Heavy metal sensor histidine kinase"

/translation="MRFKISLTTRLSLIFSAVMLTVWWLSSFILISTLNGYFDNQDRD

FLTGKLQLTEEFLKTETFRNKTDIKSLSEKINDAMVGHNGLFISIKNMENEKIVELYA

KNSVVPAVLLNKSGDILDYMIQTEENNTVYRSISRRVAVTPEQGKSKHVIITVATDTG

YHTLFMDKLSTWLFWFNIGLVFISVFLGWLTTRIGLKPLREMTSLASSMTVHSLDQRL

NPDLAPPEISETMQEFNNMFDRLEGAFRKLSDFSSDIAHELRTPVSNLMMQTQFALAK

ERDVSHYREILFANLEELKRLSRMTSDMLFLARSEHGLLRLDKHDVDLAAELNELREL

FEPLADETGKTITVEGEGVVAGDSDMLRRAFSNLLSNAIKYSPDNTCTAIHIERDSDC

VNVMITNTMSGQVPANLERLFDRFYRADSSRFYNTEGAGLGLSITRSIIHAHGGELSA

EQQGREIVFSVRLLMD"

CDS 162384..162818

/codon_start=1

/product="probable copper-binding protein"

/translation="MKKILVSFVAIMAVASSAMAAETMNMHDQVNNAQAPAHQMQSTS

EKSAVQGDSMTMMDMSGHDQAAMTHEMMQNGNASAHQDMAEMHKKMMKSKPAASNETA

KSFSEMNEHEKAAVVHEKANNGQSSVIHQQQAEKHRSQITQN"

CDS complement(163050..163229)

/codon_start=1

/product="hypothetical protein"

/translation="MFDVLAMHDIGTHRAELGDNICSLPVEQHMIYFVSSHSVVMIIR

ILSQSQDTARHEPWI"

CDS complement(163290..164213)

/codon_start=1

/product="Mobile element protein"

/translation="MLKNLQHQLGLIAGGEAGRRAAVASGIQISADTLLRRVVQAPEQ

TENRTRHVGIDEWAWHRGHRYGTLIVNSLHKKGCGIREISRITGLSRVTVRRWIQSKA

FPEISTKPPRPGLLEPWQEWLEEQRINGNHNAGQLWREMVNAGFTGSETTVRDAVAKW

RKQVNSPVIAPVRLPSASRVSRWLMPWRMIRGEENYASRFIESMCQKEPQLKMAQQLS

LDFYRMLKTKNKSQLNQWFSDVSQSGLIDLQRVAAGMEADATAIHEAISSRWSNGVVE

GHVNRLKMLKRQMYGRAGFELLRRRVMSPLA"

CDS 164213..164398

/codon_start=1

/product="hypothetical protein"

/translation="MQKTAAAGLQREQAIQQKFYVNRKGYDSTSAGWRRLTRGPGLSM

EDAVLASAGNYEQNDCA"

CDS 164653..165108

/codon_start=1

/product="Aquaporin Z"

/translation="MVSLIFYLIGACSFRATMNYIVAQLLGAGAAALCLKAVFGHSLL

AGVTRVHMGVSLYNAFFIEGVMTFILIMSILTTRNPAIISIAVFLDAFIGGPLTGASM

NPARSFGPALAMGYWDNQWLYWAAPLSGGLVAVACCQLFMPQLKSPSPE"

CDS complement(165158..165655)

/codon_start=1

/product="GCN5-related N-acetyltransferase"

/translation="MAEIIIRKMHEEDWCAVREIYQEGIATGNATFQTAAPEWLEWNE

GHLQDCRYVATVDNRVVGWAALSPFSRRHAYRGVAELSIYVSTHFQGKGAGRALLSGL

IKGSESAGFWTLLAGIFPENQASVALHRSQGFREVGCREKVGEMNGKWRDVLILERRS

RTVGC"

CDS 165987..166313

/codon_start=1

/product="Arsenical resistance operon repressor"

/translation="MELKIAAMVLKELGHTTRLDIYKTLVKAGRQGLPVGELQQHLAI

PASTLSHHLSSLISVSLVRQERQGRTLFCHACYDNLAALIAFLTEECCADEYAPPGFM

SPPEKK"

CDS 166310..167023

/codon_start=1

/product="Arsenic resistance protein ArsH"

/translation="MIAELKNVVPELFDTGLTGVRLGVKTVDHPPRILMLYGSVRERS

YSRLATEEAARLLTAMGADVRIFNPSGLPLPDDAADTHPRVMELREMVRWSEGMVWCS

PERHGAMTGIMKAQIDWIPLSEGAVRPSQGKTLAVMQVCGGSQSFNAVNQMRILGRWM

RMITIPNQSSVAKAWQEFDEDGRMKPSPYYDRIVDVMEELMKFTLLTREYAAYLVDRY

SERKESAEALSRRVNQSKI"

CDS 167032..167577

/codon_start=1

/product="hypothetical protein"

/translation="MSDVTAFNNCMSVFWQQHEAELSRFLASRTGDREQAADLLQEVF

LRARACADRFCEMENPRAWLYRTARNLLTDEYRAARDVVVLEDEIPLPDAFHEAVSTL

EICLPETLQALPDEERWLIEEADLNRRPQQRLADELGITLTAFKSRLLRARKHLKKTM

TELCQVEVDDASSVCCHKKMD"

CDS 167653..168015

/codon_start=1

/product="Arsenical resistance operon trans-acting

repressor ArsD"

/translation="MSKIEIFEAAGCCATSSVVVSDEAVKWNASAEWAKKHGVNIQRY

SLAKNPQQFLNTPVIRAFLNTSGMESLPATLLDGQLVMAGKLPSREDIARWAGISLTQ

DWNEDSTQPRCCSIPRMP"

CDS join(168040..169137,169143..>169790)

/codon_start=1

/product="Arsenical pump-driving ATPase"

/translation="MPFLQNIPPFIFFTGKGGVGKTSLACATAVWLADQGRRTLLVST

DPASNVGQVFSQTVGHRITGIRTVENLAAMEVDPMAAAQAYRDRVLDPVRELMPADVI

SNIEEQLSGSCTTEIAAFDEFTALLTSHELREKYDHIVFDTAPTGHTIRMLELPGAWS

GYLDAHPDAAANLGPLVGLEKQQDQYADAVKALSDTALTRLVLVARAQASTLKEVSHT

HEELSAIGLQHQHLAINGVLPPFAGEQDPLAHSILAREERALRAMPENLAHLPRSMLY

LKPFNLVGLEALRALFTESTLVLPDPGATLTTVDLPELASLVEDLSRAGKGLVMTMGK

GGVGKTTVAAAVAVSLARRGHKVHLTTSDPAAXXSYTLDGSLPGLQVSRIDPKAETER

YRRFVLENQGKGLDEEGLAVLEEDLRSPCTEEIAVFQAFSRIIKEANDHFVIIDTAPT

GHTLLLLDATGAYHREMVRQMGQAHDHVITPMMQLQDPEKTRVIIVTLAETTPVLEAA

GLQQDLRRAGIEPWAWVINNSLAAAKPSSPFLVTRARRELPLIDDVAGHYAQRIALTP

LLKDDPVGVDLLAEMAG"

CDS 169912..170448

/codon_start=1

/product="hypothetical protein"

/translation="MHIINAEEQHIPAIRRIYAHHVLHGTGSFETEPPDTQEMLARVK

NVQSRGFPWYIALQGETVIGYCYLSRYRERHAYRFTVENSVYIDPAYQRQGGGKALLD

HALTWARSQGYRQMIAVVGDSANVASVALHLRAGFTEIGTLKDIGFKHGRWLDTVLLQ

RQLGKGSCTLPDSPVPGR"

CDS complement(170481..170906)

/codon_start=1

/product="Arsenate reductase"

/translation="MSNITIYHNPACGTSRNTLEMIRNSGNEPTVIHYLENPPSRDEL

VKLIADMGISVRALLRKNVEPYEELGLEEDKFTDDQLIDFMLQHPILINRPIVVTPLG

TRLCRPSEVVLDILPDAQKGAFAKEDGEKVVDEAGKRLK"

CDS complement(170919..171755)

/codon_start=1

/product="Arsenic efflux pump protein"

/translation="MNKTKGCLIANFATVPFGLGFTEYASVMVPVDIAAIVATLVMLH

LFFRKDIPPTYDLALLKAPAKAIKDLATFRTGWIVLILLLVGFFVLEPLGIPVSAIAA

VGAVILFAVAKRGHAIKTGKVLRGAPWQIVIFSLGMYLVVYGLRNAGLTEYLSGVLNV

LADKGLWAATLGTGFLTAFLSSIMNNMPTVLVGALSIDGSTATGVIKEAMIYANVIGC

DLGPKITPIGSLATLLWLHVLSQKNMTITWGYYFRTGIVMTLPVLFVTLAALALRLSF

TL"

CDS 172456..175140

/codon_start=1

/product="Mobile element protein"

/translation="MGSCAAPSAKGDDKFITTDYLQQCPRSWISNERPGLLFDLATGW

LMQHRIILPGATTLTRLISEVREKATLRLWNKLALIPSAEQRSQLEMLLGPTDCSRLS

LLESLKKGPVTISGPAFNEAIERWKTLNDFGLHAENLSTLPAVRLKNLARYAGMTSVF

NIARMSPQKRMAVLVAFVLAWETLALDDALDVLDAMLAVIIRDARKIGQKKRLRSLKD

LDKSALALASACSYLLKEETPDESIRAEVFSYIPRQKLAEIITLVREIARPSDDNFHE

EMVEQYGRVRRFLPHLLNTVKFSSAPAGVTTLNACDYLSREFSSRRQFFDDAPTEIIS

RSWKRLVINKEKHITRRGYTLCFLSKLQDSLRRRDVYVTGSNRWGDPRARLLQGADWQ

ANRIKVYRSLGHPTDPQEAIKSLGHQLDSRYRQVAARLCENEAVELDVSGPKPRLTIS

PLASLDEPDSLKRLSKMISDLLPPVDLTELLLEINAHTGFADEFFHASEASARVDDLP

VSISAVLMAEACNIGLEPLIRSNVPALTRHRLNWTKANYLRAETITSANARLVDFQAT

LPLAQIWGGGEVASADGMRFVTPVRTINAGPNRKYFGNNRGITWYNFVSDQYSGFHGI

VIPGTLRDSIFVLEGLLEQETGLNPTEIMTDTAGASELVFGLFWLLGYQFSPRLADAG

ASVFWRMDHDADYGVLNDIARGQSDPRKIVLQWDEMIRTAGSLKLGKVQVSVLVRSLL

KSERPSGLTQAIIEVGRINKTLYLLNYIDDEDYRRRILTQLNRGESRHAVARAICHGQ

KGEIRKRYTDGQEDQLGTLGLVTNAVVLWNTIYMQAALDHLRAQGETLNDEDIARLSP

LCHGHINMLGHYSFTLAELVTKGHLRPLKEASEAENVA"

CDS join(175210..175408,199907..199926)

/codon_start=1

/translation="MNPFKGRHFQRDIILWAVRWYCKYGISYRELQEMLAERGVNVDH

STIYRWVQRYAPEMEKRLRWYWRALLQS"

CDS 175210..175914

/codon_start=1

/product="Mobile element protein"

/translation="MNPFKGRHFQRDIILWAVRWYCKYGISYRELQEMLAERGVNVDH

STIYRWVQRYAPEMEKRLRWYWRNPSDLCPWHMDETYVKVNGRWAYLYRAVDSRGRTV

DFYLSSRRNSKAAYRFLGKILNNVKKWQIPRFINTDKAPAYGRALALLKREGRCPSDV

KHRQIKYRNNVIECDHGKLKRIIGATLGFKSMKTAYATIKGIEVMRALRKGQASAFYY

GDPLGEMRLVSRVFEM"

CDS 175210..175914

/codon_start=1

/translation="MNPFKGRHFQRDIILWAVRWYCKYGISYRELQEMLAERGVNVDH

STIYRWVQRYAPEMEKRLRWYWRNPSDLCPWHMDETYVKVNGRWAYLYRAVDSRGRTV

DFYLSSRRNSKAAYRFLGKILNNVKKWQIPRFINTDKAPAYGRALALLKREGRCPSDV

EHRQIKYRNNVIECDHGKLKRIIGATLGFKSMKTAYATIKGIEVMRALRKGQASAFYY

GDPLGEMRLVSRVFEM"

CDS 175210..175914

/codon_start=1

/translation="MNPFKGRHFQRDIILWAVRWYCKYGISYRELQEMLAERGVNVDH

STIYRWVQRYAPEMEKRLRWYWRNPSDLCPWHMDETYVKVNGRWAYLYRAVDSRGRTV

DFYLSSRRNSKAAYRFLGKILNNVKKWQIPRFINTDKAPAYGRALALLKREGRCPSDV

EHRQIKYRNNVIECDHGKLKRIIGATLGFKSMKTAYATIKGIEVMRALRKGQASAFYY

GDPLGEMRLVSRVFEM"

CDS 175210..175914

/codon_start=1

/translation="MNPFKGRHFQRDIILWAVRWYCKYGISYRELQEMLAERGVNVDH

STIYRWVQRYAPEMEKRLRWYWRNPSDLCPWHMDETYVKVNGRWAYLYRAVDSRGRTV

DFYLSSRRNSKAAYRFLGKILNNVKKWQIPRFINTDKAPAYGRALALLKREGRCPSDV

EHRQIKYRNNVIECDHGKLKRIIGATLGFKSMKTAYATIKGIEVMRALRKGQASAFYY

GDPLGEMRLVSRVFEM"

CDS 175210..175914

/codon_start=1

/translation="MNPFKGRHFQRDIILWAVRWYCKYGISYRELQEMLAERGVNVDH

STIYRWVQRYAPEMEKRLRWYWRNPSDLCPWHMDETYVKVNGRWAYLYRAVDSRGRTV

DFYLSSRRNSKAAYRFLGKILNNVKKWQIPRFINTDKAPAYGRALALLKREGRCPSDV

EHRQIKYRNNVIECDHGKLKRIIGATLGFKSMKTAYATIKGIEVMRALRKGQASAFYY

GDPLGEMRLVSRVFEM"

CDS 175210..175914

/codon_start=1

/translation="MNPFKGRHFQRDIILWAVRWYCKYGISYRELQEMLAERGVNVDH

STIYRWVQRYAPEMEKRLRWYWRNPSDLCPWHMDETYVKVNGRWAYLYRAVDSRGRTV

DFYLSSRRNSKAAYRFLGKILNNVKKWQIPRFINTDKAPAYGRALALLKREGRCPSDV

EHRQIKYRNNVIECDHGKLKRIIGATLGFKSMKTAYATIKGIEVMRALRKGQASAFYY

GDPLGEMRLVSRVFEM"

CDS 175210..175914

/codon_start=1

/translation="MNPFKGRHFQRDIILWAVRWYCKYGISYRELQEMLAERGVNVDH

STIYRWVQRYAPEMEKRLRWYWRNPSDLCPWHMDETYVKVNGRWAYLYRAVDSRGRTV

DFYLSSRRNSKAAYRFLGKILNNVKKWQIPRFINTDKAPAYGRALALLKREGRCPSDV

EHRQIKYRNNVIECDHGKLKRIIGATLGFKSMKTAYATIKGIEVMRALRKGQASAFYY

GDPLGEMRLVSRVFEM"

CDS 175210..175914

/codon_start=1

/translation="MNPFKGRHFQRDIILWAVRWYCKYGISYRELQEMLAERGVNVDH

STIYRWVQRYAPEMEKRLRWYWRNPSDLCPWHMDETYVKVNGRWAYLYRAVDSRGRTV

DFYLSSRRNSKAAYRFLGKILNNVKKWQIPRFINTDKAPAYGRALALLKREGRCPSDV

KHRQIKYRNNVIECDHGKLKRIIGATLGFKSMKTAYATIKGIEVMRALRKGQASAFYY

GDPLGEMRLVSRVFEM"

CDS 175210..175914

/codon_start=1

/translation="MNPFKGRHFQRDIILWAVRWYCKYGISYRELQEMLAERGVNVDH

STIYRWVQRYAPEMEKRLRWYWRNPSDLCPWHMDETYVKVNGRWAYLYRAVDSRGRTV

DFYLSSRRNSKAAYRFLGKILNNVKKWQIPRFINTDKAPAYGRALALLKREGRCPSDV

EHRQIKYRNNVIECDHGKLKRIIGATLGFKSMKTAYATIKGIEVMRALRKGQASAFYY

GDPLGEMRLVSRVFEM"

CDS 175982..176158

/codon_start=1

/product="hypothetical protein"

/translation="MSLNALSVTLADNVQLSLQAGFIQRPAIRHPYHDLKGRQQAQKT

LQRGQRALPNNKPV"

CDS 176233..177666

/codon_start=1

/product="DNA-cytosine methyltransferase"

/translation="MSEFELLAQDLLEKAEAEEQLRQENDKKLLGQVLEIYDQKYVAE

LLRKVGKNEWSRETLNRWINGKCSPKTLTLAEEELLRKMLPEAPAHHPDYAFRFIDLF

AGIGGIRKGFETIGGQCVFTSEWNKEAVRTYKANWFNDAQEHTFNLDIREVTLSDKPE

VPENDAYAYINEHVPDHDVLLAGFPCQPFSLAGVSKKNSLGRAHGFECEAQGTLFFDV

ARIIRAKKPAIFVLENVKNLKSHDKGKTFKVIMDTLDELGYEVADAAEMGKNDPKVID

GKHFLPQHRERIVLVGFRRDLNIHQGFTLRDISRFYPEQRPSFGELLEPVVDSKYILT

PKLWEYLYNYAKKHAAKGNGFGFGLVNPENKESIARTLSARYHKDGSEILIDRGWDMA

TGETDFANEENQAHRPRRLTPRECARLMGFEKVDGRPFRIPVSDTQSYRQFGNSVVVP

VFEAVAKLLEPYILKAVNADSCKVERI"

CDS complement(177700..178908)

/codon_start=1

/product="hypothetical protein"

/translation="MSVFHNWLLEIACENYFVYIKRLSANDTGATGGHQVGLYIPSGI

VEKLFPSINHTRELNPSVFLTAHVSSHDCPDSEARAIYYNSRHFGKTRNEKRITRWGR

GSPLQDPENTGALTLLAFKLDEQGGDCKEVNIWVCASTDEEDVIETAIGEVIPGALIS

GPAGQILGGLSLQQAPVNHKYILPEDWHLRFPSGSEIIQYAASHYVKNSLDPDEQLLD

RRRVEYDIFLLVEELHVLDIIRKGFGSVDEFIALANSVSNRRKSRAGKSLELHLEHLF

IEHGLRHFATQAITEGNKKPDFLFPSAGAYHDTEFPVENLRMLAVKTTCKDRWRQILN

EADKIHQVHLFTLQEGVSLAQYREMRESGVRLVVPSSLHKKYPEAVRAELMTLGAFIA

ELTGLYADIP"

CDS complement(178921..179043)

/codon_start=1

/product="TniB NTP-binding protein"

/translation="MNCQELAAQQPLRPINWCSRLLKMTLFVYNHEKMVSRVSG"

CDS complement(179175..179969)

/codon_start=1

/product="Mobile element protein"

/translation="MLGGDWTDGTMTDFKWRHFQGDVILWAVRWYCRYPISYRDLEEM

LAERGISVDHTTIYRWVQCYAPEMEKRLRWFWRRGFDPSWRLDETYVKVRGKWTYLYR

AVDKRGDTIDFYLSPTRSAKAAKRFLGKALRGLKHWEKPATLNTDKAPSYGAAITELK

REGKLDRETAHRQVKYLNNVIEADHGKLKILIKPVRGFKSIPTAYATIKGFEVMRALR

KGQARPWCLQPGIRGEVRLVERAFGIGPSALTEAMGMLNHHFAAAA"

CDS complement(180082..180348)

/codon_start=1

/product="hypothetical protein"

/translation="MPVLTDEEVNTVRESCRQLGAIGRNLNQVARALNIEFRESDKLK

QEAIEKLAERIDQHLDHVSELFDKTWSRWHDCHFQKTTAPVDWA"

CDS complement(180569..181051)

/codon_start=1

/product="Dihydrofolate reductase"

/translation="MRTLKVSLMAAKAKNGVIGCGPDIPWSAKGEQLLFKALTYNQWL

LVGRKTFESMGALPNRKYAVVTRSGWTSNDDNVVVFQSIEEAMDRLAEFTGHVIVSGG

GEIYRETLPMASTLHLSTIDIEPEGDVFFPSIPNTFEVVFEQHFTSNINYCYQIWKKG

"

CDS 181198..182211

/codon_start=1

/product="Integron integrase IntIPac"

/translation="MKTATAPLPPLRSVKVLDQLRERIRYLHYSLRTEQAYVHWVRAF

IRFHGVRHPATLGSSEVEAFLSWLANERKVSVSTHRQALAALLFFYGKVLCTDLPWLQ

EIGRPRPSRRLPVVLTPDEVVRILGFLEGEHRLFAQLLYGTGMRISEGLQLRVKDLDF

DHGTIIVREGKGSKDRALMLPESLAPSLREQLSRARAWWLKDQAEGRSGVALPDALER

KYPRAGHSWPWFWVFAQHTHSTDPRSGVVRRHHMYDQTFQRAFKRAVEQAGITKPATP

HTLRHSFATALLRSGYDIRTVQDLLGHSDVSTTMIYTHVLKVGGAGVRSPLDALPPLT

SER"

CDS 181198..182211

/codon_start=1

/translation="MKTATAPLPPLRSVKVLDQLRERIRYLHYSLRTEQAYVHWVRAF

IRFHGVRHPATLGSSEVEAFLSWLANERKVSVSTHRQALAALLFFYGKVLCTDLPWLQ

EIGRPRPSRRLPVVLTPDEVVRILGFLEGEHRLFAQLLYGTGMRISEGLQLRVKDLDF

DHGTIIVREGKGSKDRALMLPESLAPSLREQLSRARAWWLKDQAEGRSGVALPDALER

KYPRAGHSWPWFWVFAQHTHSTDPRSGVVRRHHMYDQTFQRAFKRAVEQAGITKPATP

HTLRHSFATALLRSGYDIRTVQDLLGHSDVSTTMIYTHVLKVGGAGVRSPLDALPPLT

SER"

CDS 182333..182464

/codon_start=1

/product="BsuBI-PstI family restriction endonuclease"

/translation="MPDGSNPSAAIVQSASDVQCSRLLKTTNSQKGCYRRFDLQPIG"

CDS complement(182629..182766)

/codon_start=1

/product="Mobile element protein"

/translation="MGSCAAPSAKGDDKFITTDYLQQCRFAHIQLLLVRQPGAPHPED

A"

CDS 183467..184150

/codon_start=1

/product="Oxidoreductase"

/translation="MNKTKGCLIANFATVPEEDDAREVVDLIKKAGRNVLAIPGDIRD

EAFCGHLVTQAVKGLGGLDILVNNAGRQQFCESIEELTTEDFDATFKTNVYAMFWITK

AAIPHLSPDSVIINTSSVQAYEPSEILLDYAQTKAAIVAFTKSLAKQLAPKGIRVNAV

APGPYWTVLQCCGGQPQEKVEKFGANAPLGRPGQPVEIAPLYVTLAARENSYTSGQVW

CSDGGTGTL"

CDS complement(184242..184886)

/codon_start=1

/product="Qnr"

/translation="MALALVGEKIDRNRFTGEKIENSTFFNCDFSGADLSGTEFIGCQ

FYDRESQKGCNFSRAMLKDAIFKSCDLSMADFRNSSALGIEIRHCRAQGADFRGASFM

NMITTRTWFCSAYITNTNLSYANFSKVVLEKCELWENRWIGAQVLGATFSGSDLSGGE

FSTFDWRAANFTHCDLTNSELGDLDIRGVDLQGVKLDNYQASLLMERLGIAVIG"

CDS complement(184980..185366)

/codon_start=1

/product="Psp operon transcriptional activator"

/translation="MCREIGLPLFPGFSAEARETLLHYRWPGNIRELKNVVERSVYRH

GTSDYPLDEIIIDPFRRHTTQPQAPETKPMSVGLPLDLREFQQQQEKDFLQTSLQQAK

FNQKKAAELLGLTYHQLRALLKKHQI"

CDS complement(185457..187292)

/codon_start=1

/product="Mobile element protein"

/translation="MSFRPQPEPTPCLRFEGCDGTQHLAAALAKTQNELNGQDAQLQT

ADTRLLSKKSREFLLDGEGNILIDRYEWFLYQQIPDRLNGQLTLPDITKYRALDADLI

DGEHWRKNKYTLLQQSHFTKLAEEPEKLIKQMAMELDTRLYEVGEYLEQEDNRNIILR

NPQGKRFWRLPSASKHHLVNNPFFQQIPTTGIADVLRMVDRDTGFIDCFAHVLGSQSR

SRSHEYDLLAILVGNATNQGIYGMAQISDRTYDQLSTIQANYLRLETLNAANDNINNA

TAKLPIFRYYNIQEDVIHASADGQKFEARRETFKTRYSSKYFGTQKGVSAMTLIANHA

AINARVIGANEHESHYIFDLLMSNTSDIIPDVLSTDTHGVNHVNFALLDLFGYQFAPR

YAQVGKVINDMFDVKEDKEHRIQLCLKKPINTHRIAQHWDTIQRIAVSLKQRKTTQAT

LVRKLSEYKRNHPLLEALTEYNRLVKANYLLCYIDDASLRNYVQRALNRGEAYHQLRR

AVSSVNGDQFRGSSDEEIQLWNECARLVTNAIVYFNSRILSQLLTSFEYQGDTKRIDI

VKQASPVAWHNINLKGTYHFELSEKLPDLEELMRSIEGYLPVSEK"

CDS complement(187294..190191)

/codon_start=1

/product="Aldehyde dehydrogenase"

/translation="MSRRHIFTERQRAALFDLPTDELSLLKFYTLGDDDLENIRQRRR

PENRIGFALQLCALRYPGRALAPGEMIPREVLSFVGAQLGVPADALLTYATRRQTRQQ

HMDTLREIYGYKTFTGRGARDLREWTFGQAEDARSNEDLAHRFIVRCRETSTILPAVS

TIERLCADALVAAERRIETRIAENLTADVRDHLDKLLSEMLAGNISRFIWLRNFEVGN

NSAAANRLLDRLEFLRTLNINHSALASIPAHRIARLRRQGERYFTDGLRDITSDRRWA

ILAVCVVEWEAAIADAIVETHDRIVGKTWREAKRQHDETISGSKATLTDTIRTFTALG

ASLLEARSDGTPLEMAVASSVAWDRLAQLVATGTQLSNTLADEPLAYVGQGYHRFRRY

APRMLRCLKLEAAPVAGPLVAAALSIGEMKGVASPERRFLRPSSKWNRHLRAQEKGDT

RLWEVAVLFHLRDAFRSGDVWLAHSRRYGDLKQVLVPMIAAQENAKLAVPSNPQDWLA

DRKARLTIALKRLARAARNGTIPHGSIEDGTLRIDRLTADVPDGAEALILDLYRRMPS

VRITDMLLEVDAALGFTDAFTHLRTGAPCRDRIGLLNVLLAEGLNLGLRKMAEATNTH

DYWQLSRLARWHVESEAMNQALAIVVAAQGKLPMSRVWGMGTSASSDGQFFPTARHGE

AMNMVNAKYGSVPGLKAYTHVSDQFAPFACQSIPATVSEAPYILDGLLMNEVGRHVRE

QYADTAGFTDHLFGASSLLGYNLVLRIRDLPSKRLYVFNPDTTPRELRKLVGGKARED

LIVANWPDIFRCAATMTAGKIRPSQLLRKLASYPRQNNLAVALREVGRIERTLFIIEW

ILDTDMQRRAQIGLNKGEAHHALKNALRIGRQGEIRDRTTEGQHYRIAGLNLLTAVII

YWNTVHLGHAVTERRNEGLDVPPEFLPHISPLGWAHILLTGEYLWPKEPKA"

CDS 190286..190891

/codon_start=1

/product="DNA integration/recombination/invertion protein"

/translation="MGHRAAIYCRVSTADQSCERQEFDLRAFAGCAGYDVVGIFKETG

SGTKLDRAERKKVLALAQSRQIDAILVTELSRWGRSTLDLLNTLRELENWKVSVIAMN

GMAFDLSSPYGRMLATFLSGIAEFERDLISERVKSGLAVAKARGKRLGRQAGVRPKSD

RLLPKVVAMRAEGRSYRWIARELGISKNTVADIVQRHRANA"

CDS complement(190888..191649)

/codon_start=1

/product="Mobile element protein"

/translation="MLAAVQTLREMNADNLRKVPADAPTAFIKPRWKPLVITPEGLDR

KFYEICALSELKNALRSGDIWVKGSRQFRDFDDYLLPAEKFAALKREQALPLAINPNS

DQYLEERLQLLDEQLATVTRLAKDNELPDAILTESGLKITPLDAAVPDRAQALIDQTS

QLLPRIKITELLMDVDDWTGFSRHFTHLKDGAEAKDRTLLLSAILGDAINLGLTKMAE

SSPGLTYAKLSWLQAWHIRDETYSGSVPAEGEMTP"

CDS 191653..192060

/codon_start=1

/product="Amidases related to nicotinamidase"

/translation="MRGRPVLFPNFSLGEEYEHAPPATNRQISPYLPSGRFRTGLPVE

GLAIERGDLFYACPRASVFYGTALDADLRTRGVSTLVMAGISTTGVVLSSVAWASDAD

YDVRLVQDCCYDPDRDAHEALLRSGFGGRVQVV"

CDS complement(192128..192247)

/codon_start=1

/product="hypothetical protein"

/translation="MAGAIAVNNKSGVRNDMRLQDLRYGDGFLRVAGAAMTAP"

CDS 192198..193082

/codon_start=1

/product="Permease of the drug/metabolite transporter

(DMT) superfamily"

/translation="MSLRTPDLLFTAIAPAIWGSTYIVTTQYLPNFSPMTVAMLRALP

AGLLLVMIVRQIPTGIWWMRIFILGALNISLFWSLLFISVYRLPGGVAATVGAVQPLM

VVFISAALLGSPIRLMAVLGAICGTAGVALLVLTPNAALDPVGVAAGLAGAVSMAFGT

VLTRKWQPPVPLLTFTAWQLAAGGLLLVPVALVFDPPIPMPTGTNVLGLAWLGLIGAG

LTYFLWFRGISRLEPTVVSLLGFLSPGTAVLLGWLFLDQTLSALQIIGVLLVIGSIWL

GQRSNRTPRARIACRKSP"

CDS complement(193114..194313)

/codon_start=1

/product="Tetracycline efflux protein TetA"

/translation="MKPNRPLIVILSTVALDAVGIGLIMPVLPGLLRDLVHSNDVTAH

YGILLALYALMQFACAPVLGALSDRFGRRPVLLVSLAGAAVDYAIMATAPFLWVLYIG

RIVAGITGATGAVAGAYIADITDGDERARHFGFMSACFGFGMVAGPVLGGLMGGFSPH

APFFAAAALNGLNFLTGCFLLPESHKGERRPLRREALNPLASFRWARGMTVVAALMAV

FFIMQLVGQVPAALWVIFGEDRFHWDATTIGISLAAFGILHSLAQAMITGPVAARLGE

RRALMLGMIADGTGYILLAFATRGWMAFPIMVLLASGGIGMPALQAMLSRQVDEERQG

QLQGSLAALTSLTSIVGPLLFTAIYAASITTWNGWAWIAGAALYLLCLPALRRGLWSG

AGQRADR"

CDS 194392..195069

/codon_start=1

/product="Transcriptional regulator, TetR family"

/translation="MFISDKVSSMTKLQPNTVIRAALDLLNEVGVDGLTTRKLAERLG

VQQPALYWHFRNKRALLDALAEAMLAENHTHSVPRADDDWRSFLIGNARSFRQALLAY

RDGARIHAGTRPGAPQMETADAQLRFLCEAGFSAGDAVNALMTISYFTVGAVLEEQAG

DSDAGERGGTVEQAPLSPLLRAAIDAFDEAGPDAAFEQGLAVIVDGLAKRRLVVRNVE

GPRKGDD"

CDS complement(195385..196974)

/codon_start=1

/product="Mobile element protein"

/translation="MGSCAAPSAKGDDKFITTDYLQQCHFDDYLLPAEKFAALKREQA

LPLAINPNSDQYLEERLQLLDEQLATVTRLAKDNELPDAILTESGLKITPLDAAVPDR

AQALIDQTSQLLPRIKITELLMDVDDWTGFSRHFTHLKDGAEAKDRTLLLSAILGDAI

NLGLTKMAESSPGLTYAKLSWLQAWHIRDETYSAALAELVNHQYRHAFAAHWGDGTTS

SSDGQRFRAGGRGESTGHVNPKYGSEPGRLFYTHISDQYAPFSTRVVNVGVRDSTYVL

DGLLYHESDLRIEEHYTDTAGFTDHVFALMHLLGFRFAPRIRDLGETKLYVPQGVQAY

PTLRPLIGGTLNIKHVRAHWDDILRLASSIKQGTVTASLMLRKLGSYPRQNGLAVALR

ELGRIERTLFILDWLQSVELRRRVHAGLNKGEARNSLARAVFFNRLGEIRDRSFEQQR

YRASGLNLVTAAIVLWNTVYLERATQGLVEAGKPVDGELLQFLSPLGWEHINLTGDYV

WRQSRRLEDGKFRPLRMPGKP"

CDS 197768..198367

/codon_start=1

/product="6'-N-acetyltransferase"

/translation="MSNAKTKLGITKYSIVTNSNDSVTLRLMTEHDLAMLYEWLNRSH

IVEWWGGEEARPTLADVQEQYLPSVLAQESVTPYIAMLNGEPIGYAQSYVALGSGDGR

WEEETDPGVRGIDQLLANASQLGKGLGTKLVRALVELLFNDPEVTKIQTDPSPSNLRA

IRCYEKAGFERQGTVTTPYGPAVYMVQTRQAFERTRSDA"

CDS 198453..199328

/codon_start=1

/product="Beta-lactamase"

/translation="MLAVKIKPFTKPILIMKNTIHINFAIFLIIANIIYSSASASTDI

STVASPLFEGTEGCFLLYDASTNAEIAQFNKAKCATQMAPDSTFKIALSLMAFDAEII

DQKTIFKWDKTPKGMEIWNSNHTPKTWMQFSVVWVSQEITQKIGLNKIKNYLKDFDYG

NQDFSGDKERNNGLTEAWLESSLKISPEEQIQFLRKIINHNLPVKNSAIENTIENMYL

QDLDNSTKLYGKTGAGFTANRTLQNGWFEGFIISKSGHKYVFVSALTGNLGSNLTSSI

KAKKNAITILNTLNL"

CDS complement(199580..199702)

/codon_start=1

/product="hypothetical protein"

/translation="MPLIASHDKGSPTPDRAETTDDQLINVITVRKQIPCAVIE"

CDS 200771..201631

/codon_start=1

/product="Aminoglycoside N(3')-acetyltransferase III"

/translation="MHTRKAITEAIRKLGVQTGDLLMVHASLKAIGPVEGGAETVVAA

LRSAVGPTGTVMGYASWDRSPYEETLNGARLDDKARRTWPPFDPATAGTYRGFGLLNQ

FLVQAPGARRSAHPDASMVAVGPLAETLTEPHELGHALGEGSPVERFVRLGGKALLLG

APLNSVTALHYAEAVADIPNKRWVTYEMPMLGRNGEVAWKTASEYDSNGILDCFAIEG

KPDAVETIANAYVKLGRHREGVVGFAQCYLFDAQDIVTFGVTYLEKHFGATPIVPAHE

AAQRSCEPSG"

CDS 202326..202589

/codon_start=1

/product="Transposase and inactivated derivatives"

/translation="MALAAIRGEQTLVELSQQFDVHANQIKQWKDQLLEGATGVFGDE

TKAEPSGPTIDVKTLHAKIGELTLENDFLSGALGKAGLLGGKK"

CDS complement(204131..206146)

/codon_start=1

/product="Mobile element protein"

/translation="MQHRIILPGATTLTRLISEVREKATLRLWNKLALIPSAEQRSQL

EMLLGPTDCSRLSLLESLKKGPVTISGPAFNEAIERWKTLNDFGLHAENLSTLPAVRL

KNLARYAGMTSVFNIARMSPQKRMAVLVAFVLAWETLALDDALDVLDAMLAVIIRDAR

KIGQKKRLRSLKDLDKSALALASACSYLLKEETPDESIRAEVFSYIPRQKLAEIITLV

REIARPSDDNFHEEMVEQYGRVRRFLPHLLNTVKFSSAPAGVTTLNACDYLSREFSSR

RQFFDDAPTEIISRSWKRLVINKEKHITRRGYTLCFLSKLQDSLRRRDVYVTGSNRWG

DPRARLLQGADWQANRIKVYRSLGHPTDPQEAIKSLGHQLDSRYRQVAARLCENEAVE

LDVSGPKPRLTISPLASLDEPDSLKRLSKMISDLLPPVDLTELLLEINAHTGFADEFF

HASEASARVDDLPVSISAVLMAEACNIGLEPLIRSNVPALTRHRLNWTKANYLRAETI

TSANARLVDFQATLPLAQIWGGGEVASADGMRFVTPVRTINAGPNRKYFGNNRGITWY

NFVSDQYSGFHGIVIPGTLRDSIFVLEGLLEQETGLNPTEIMTDTAGASELVFGLFWL

LGYQFSPRLADAGASVFWRMDHDADYGVLNDIARGQSDPRKIGHCCK"

CDS 206469..206738

/codon_start=1

/product="Tryptophan synthase beta chain like"

/translation="MLNFDDRGTVTHRAILGETCTVLEMAAGTWHAVLSLDTGGIIFE

VKHGGYQPVAADDYAHWAPAEGEPGTTELMAWYAQAQVGDSTFAV"

CDS complement(206785..207660)

/codon_start=1

/product="Beta-lactamase"

/translation="MVKKSLRQFTLMATATVTLLLGSVPLYAQTADVQQKLAELERQS

GGRLGVALINTADNSQILYRADERFAMCSTSKVMAAAAVLKKSESEPNLLNQRVEIKK

SDLVNYNPIAEKHVNGTMSLAELSAAALQYSDNVAMNKLIAHVGGPASVTAFARQLGD

ETFRLDRTEPTLNTAIPGDPRDTTSPRAMAQTLRNLTLGKALGDSQRAQLVTWMKGNT

TGAASIQAGLPASWVVGDKTGSGGYGTTNDIAVIWPKDRAPLILVTYFTQPQPKAESR

RDVLASAAKIVTDGL"

CDS complement(209360..209578)

/codon_start=1

/product="transposase"

/translation="MPVDFLTTEQTESYGRFTGEPDELQLARYFHLDEADKEFIGKSR

GDHNRLGIALQIGCVRFLGTFLTDMNHT"

CDS 209742..210299

/codon_start=1

/product="Mobile element protein"

/translation="MRLFGYARVSTSQQSLDLQVRALKDAGVKANRIFTDKASGSSTD

REGLDLLRMKVEEGDVILVKKLDRLGRDTADMIQLIKEFDAQGVAVRFIDDGISTDGD

MGQMVVTILSAVAQAERRRILERTNEGRQEAKLKGIKFGRRRTVDRNVVLTLHQKGTG

ATEIAHQLSIARSTVYKILEDERAS"

CDS 210482..211342

/codon_start=1

/product="Beta-lactamase"

/translation="MSIQHFRVALIPFFAAFCLPVFAHPETLVKVKDAEDQLGARVGY

IELDLNSGKILESFRPEERFPMMSTFKVLLCGAVLSRVDAGQEQLGRRIHYSQNDLVE

YSPVTEKHLTDGMTVRELCSAAITMSDNTAANLLLTTIGGPKELTAFLHNMGDHVTRL

DRWEPELNEAIPNDERDTTMPAAMATTLRKLLTGELLTLASRQQLIDWMEADKVAGPL

LRSALPAGWFIADKSGAGERGSRGIIAALGPDGKPSRIVVIYTTGSQATMDERNRQIA

EIGASLIKHW"

CDS 211552..212091

/codon_start=1

/product="Mobile element protein"

/translation="MPHVAARTASRDRDTGRYQSHRPEQTLLYQIVDEYYPAFAALMA

EQGKELPGYVQREFEEFLQCGRLEHGFLRVRCESCHAEHLVAFSCKRRGFCPSCGARR

MAESAALLVDEVLPEQPMRQWVLSFPFQLRFLFGVVCGKGRNPTLRLWPAIFSGEIDV

FPGDRRSRLISSMTSVAPA"

CDS complement(212063..212899)

/codon_start=1

/product="Aminoglycoside 3'-phosphotransferase 2"

/translation="MFMPPVFPAHWHVSQPVLIADTFSSLVWKVSLPDGTPAIVKGLK

PIEDIADELRGADYLVWRNGRGAVRLLGRENNLMLLEYAGERMLSHIVAEHGDYQATE

IAAELMAKLYAASEEPLPSALLPIRDRFAALFQRARDDQNAGCQTDYVHAAIIADQMM

SNASELRGLHGDLHHENIMFSSRGWLVIDPVGLVGEVGFGAANMFYDPADRDDLCLDP

RRIAQMADAFSRALDVDPRRLLDQAYAYGCLSAAWNADGEEEQRDLAIAAAIKQVRQT

SY"

CDS complement(212899..213702)

/codon_start=1

/product="Aminoglycoside 3'-phosphotransferase"

/translation="MNRTNIFFGESHSDWLPVRGGESGDFVFRRGDGHAFAKIAPASR

RGELAGERDRLIWLKGRGVACPEVINWQEEQEGACLVITAIPGVPAADLSGADLLKAW

PSMGQQLGAVHSLSVDQCPFERRLSRMFGRAVDVVSRNAVNPDFLPDEDKSTPQLDLL

ARVERELPVRLDQERTDMVVCHGDPCMPNFMVDPKTLQCTGLIDLGRLGTADRYADLA

LMIANAEENWAAPDEAERAFAVLFNVLGIEAPDRERLAFYLRLDPLTWG"

CDS complement(213763..214578)

/codon_start=1

/product="Dihydropteroate synthase"

/translation="MNKSLIIFGIVNITSDSFSDGGRYLAPDAAIAQARKLMAEGADV

IDLGPASSNPDAAPVSSDTEIARIAPVLDALKADGIPVSLDSYQPATQAYALSRGVAY

LNDIRGFPDAAFYPQLAKSSAKLVVMHSVQDGQADRREAPAGDIMDHIAAFFDARIAA

LTGAGIKRNRLVLDPGMGFFLGAAPETSLSVLARFDELRLRFDLPVLLSVSRKSFLRA

LTGRGPGDVGAATLAAELAAAAGGADFIRTHEPRPLRDGLAVLAALKETARIR"

CDS complement(214766..214909)

/codon_start=1

/product="hypothetical protein"

/translation="MYLVLLRLRYPLYCKQDIFIFYIQWLIFLLIGNTMKNTMLRKGL

TIF"

CDS 214908..215084

/codon_start=1

/product="hypothetical protein"

/translation="MLTDTKLRNLKPRDKLYKVNDREGLYVGVASENGIYGHSRFCNT

DFDDKLACLNLSGV"

CDS complement(215266..216270)

/codon_start=1

/product="Mobile element protein"

/translation="MENIALIGIDLGKNSFHIHCQDHRGKAVYRKKFTRPKLIEFLAT

CPATTIAMEACGGSHFMARKLEELGHFPKLISPQFVRPFVKSNKNDFVDAEAICEAAS

RPSMRFVQPRTESQQAMRALHRVRESLVQDKVKTTNQMHAFLLEFGISVPRGAAVISR

LSTLLEDSSLPLYLSQLLLKLQQHYHYLVEQIKDLESQLKRKLDEDEVGQRLLSIPCV

GTLTASTISTEIGDGKQYASSRDFAAATGLVPRQYSTGGRTTLLGISKRGNKKIRTLL

VQCARVFIQKLEHQSGKLADWVRELLCRKSNFVVTCALANKLARIAWALTARQQTYEA

"

CDS complement(216349..218193)

/codon_start=1

/product="Mobile element protein"

/translation="MNPFKGRHFQRDIILWAVRWYCKYGISYRELQEMLAERGVNVDH

STIYRWVQRYAPEMEKRLRWYWRNPSDLCPWHMDETYVKVNGRWAYLYRAVDSRGRTV

DFYLSSRRNSKAAYRFLGKFATVPASELPLAVATDCNRYLNDRLTLLETQLATVNRMA

TANELPDAIITESGLKITPLDAAVPDTAQALIDQTAMILPHVKITELLLEVDEWTGFT

RHFAHLKSGDPAKDKNLLLTTILADAINLGLTKMAESCPGTTYAKLAWLQAWHIRDET

YGAALADLVNAQFRHPFAEHWGDGTTSSSDGQNFRTGSKAESTGHINPKYGSSPGRTF

YTHISDQYAPFHTKVVNVGVRDSTYVLDGLLYHESDLRIEEHYTDTAGFTDHVFALMH

LLGFRFAPRIRDLGDTKLYIPKGDAAYDALKPMIGGTLNIKHVRAHWDEILRLATSIK

QGTVTASLMLRKLGSYPRQNGLAVALRELGRIERTLFILDWLQSVELRRRVHAGLNKG

EARNALARAVFFNRLGEIRDRSFEQQRYRASGLNLVTAAVVLWNTVYLERAAHALRGN

GHAVDDALLQYLSPLGWEHINLTGDYLWRSSAKIGAGKFRPLRPLQPA"

CDS 218334..218489

/codon_start=1

/product="hypothetical protein"

/translation="MSPVQAKQKQHERYEAVAVQVLRGRAGYKPAVKSRFSKSASSKF

AHTIAFA"

CDS 218538..219530

/codon_start=1

/product="hypothetical protein"

/translation="MPVQDVIPPYEQMYLLNQQLICNADQFKHAVITVGGQAVQYWIS

YYHAQYGDRLPDERLTTSVDCDYSARKDDIAAIAKTLNVKTWENKDGQPPSLAQFMLI

DQDTHDIKRDDGRLFAVPDAPDEPNVVDIIDRPGGFDRSDFQGKKLYLYTAPFYVEAT

GPGMPEMNEKVRVLNPVACMRSRFSNLIALRRDAEIEIARINALKIPCYFFLIEQFDE

QPFKVARGIFMDLWRLANDESCLRHQAFWHSWQGPLLEGQQSNNITLIDVLEGVHVYL

EGHLDDFEIPEAFVTKEVPLKLAQLRERWERYVVLNAEWAARGRRGFERNPRDD"

CDS 219557..219718

/codon_start=1

/product="Transposase"

/translation="MCKPPEHTLIGRSEGERQATLKIARTMLQNGIDRNTVMKMTGLT

EDNLAQIRH"

CDS complement(219715..220326)

/codon_start=1

/product="hypothetical protein"

/translation="MTLTEKTGHLAWCALVALALARQEQGELSPAQENLFLTRWLAAA

LKQRRFSRDVAQDIGWLLNQGRLLGVRAKLADKLGYVWRSCSGELTEQNDMFRLTYAL

ETAKDMGWNYRVMSDREWAGRYALVLNPGVNGVYLLRTNLDAAFDDNGQQTNPLTVRL

TGNVTGIMKLLNRCGWQAEPESDASLPHQFSLMARQGVPGKGD"

CDS complement(220380..220661)

/codon_start=1

/product="putative cytoplasmic protein"

/translation="MRVAESIILDALTRGGCIKTFYRISSRQAAESATRIPEGYILES

PGEREDIVLSRADFHALEKLLEQKETWEQVVGVTCFGGATWQLRPTVQS"

CDS 220987..221169

/codon_start=1

/product="hypothetical protein"

/translation="MLIQERIARTGSAFKNESERNKYFERRRAENPIFDIDTPIRKAS

FIVNAGAFGLGKSRKN"

CDS complement(222062..222184)

/codon_start=1

/product="hypothetical protein"

/translation="MNKTKGCLIANFATVPFFLCPCPGLLAFVRIHEIAWFLSI"

CDS 222953..223348

/codon_start=1

/product="Resolvase-like"

/translation="MPIWRLDRLSRSLKDLIEMVKHLESKGIGLKSLQESIDTTSSSG

MLIFHLFGALAEFERNLTRERTQAGLQAARARGRKGGRPKTLSKDKQALAVQLYNEKK

HTVAQICVLMGISRPTLYKYIESARLFKK"

CDS 223395..224045

/codon_start=1

/product="Gifsy-2 prophage protein"

/translation="MTREEYLSLFANEADRNIAYDPEPIGRYNVAPGTRVLLLSERDE

QLQLDPVHWGYAPGWWDKPPLINARVETAASSRMFKPLWQHGRAICFADGWFEWKREG

DKKQPYFIHRKDGQPILMAAIGSTPFERGDEAEGFLIVTAAADKGLVDIHDRRPLVLV

PDAAREWMKQDVSGKEAEEIAADGAVSADHFLWHPVTRAVGNVKNQGPELIEAVGL"

CDS 224342..225337

/codon_start=1

/product="hypothetical protein"

/translation="MSWFNDVFGFEESDYAETQARFFQEGPFLHTRSQPSVSYRSGIL

TMPSLAELRKAVSNLVTEPYGRCRFDILEADAYDLHRKSEVKGALIQVASQFNLLEMP

CEYTTPEKGITDYQFDHTQGPACAMACAAATVFRNYLVPMGSQTGQSTTSQLNTLADM

EKAIGIDGIRMKNGYALMSANTVQAISRYIASLDEQMRDRLRQELRIGLHSDTEVTIP

GVPEDQFVSQALCSALPVAYNSSPREEWAPFASLVLEASYEATLLAGVLNYRLTGNPR

VYVTMVGGGAFGNEPGWIISALRRALYLVSHHNLEVMFVSYRHTPAALYSLIEEF"

CDS 226167..226361

/codon_start=1

/product="hypothetical protein"

/translation="MNRTAQEVQTCWLESRQPEDRTGNEAEKFSDECWKNGLRLDKSP

SVHYQLLMETIRRTLIPRPK"

CDS complement(226405..226635)

/codon_start=1

/product="hypothetical protein"

/translation="MSYGWLFENSDDDISGYNHINLEHLPPELKQVALQIVQNQFDRD

TAEKKLAELIQQLDFDITAAELSAYLFKPGHA"

CDS complement(226649..226852)

/codon_start=1

/product="Haemolysin expression modulating protein"

/translation="MTKQEWIFRLRRCTSKETLERIIEKNQYSLSDDELEHFNAAVDH

RLAEMTMGKLYDRVPPGVWKFVK"

CDS 226810..226926

/codon_start=1

/product="hypothetical protein"

/translation="MYSALVEKSIPVSSLVLFILRYPGNIILEDAFLSVGYE"

CDS complement(226886..227218)

/codon_start=1

/product="hypothetical protein"

/translation="MCFLLFTGWMFAFAQYAPYNGQEHAFSLTYLLNLFFLHVVMVPY

YEFFQTEPYSNALMSLADESGQMIRFHQVVPVLWIYSLSSLLTFVVYITYAKWFTHNR

LKEKHLPE"

CDS complement(227298..227792)

/codon_start=1

/product="hypothetical protein"

/translation="MKVTEPGLNKLIDNLNTLICEDSLLTRQERETLVLAVAAIGAMK

ARVGLKKGDAPTVARREKREKKDRQPDPRFPRAGHPWQEDEKTLLSDALDSVPDEEIG

KHLFWLSEKLGRTPFSVAFQIAAIRELQDGWEEQFREISDNIRLSGLSISDYLKQNGT

DLNA"

CDS complement(227823..228395)

/codon_start=1

/product="YdeA protein"

/translation="MMREKSRRPSPLQRRVLIVLAALDAKRPGPVATRDIERVLEQGG

DAPVYGPNLRASCRRMEAAGWLRTLRAPNLQLAVELTQDGRGIAEPLFQAEREAETAR

QRLTDVRRLPLRQTAAGDAVELQLGDGHYSLREAAYVIRLDGTTCLQLTDAGGIRRIK

EGDPLQVASWYQTCFDAGLPVTVQVNESRD"

CDS complement(228392..228640)

/codon_start=1

/product="hypothetical protein"

/translation="MDQKMTFSLSYEQMTQMAEEEIKQCDFRRDGTHYVWEVNKAHDI

LRFWYLLALRGHTGLSTTRVEADYKRLKTLISQRNEGQ"

CDS complement(228771..228908)

/codon_start=1

/product="hypothetical protein"

/translation="MTLSRWYTSITFRGKYMCVTEIRLSIFSDLRGGKHAKNMIIKPS

E"

CDS 228998..229348

/codon_start=1

/product="Arsenical resistance operon repressor"

/translation="MPLTALKLFKNLSDETRLGIVLLLRELGELCVCDFCTALDESQP

KISRHLAMLRESGLLLDRKQGKWVHYRLSPHIPSWAAQVIEQAWLSQQDDVQAIARKL

ASANCSGSGKAVCI"

CDS 229380..229760

/codon_start=1

/product="Arsenical resistance operon trans-acting

repressor ArsD"

/translation="MYEVFRMKTLTVFDPAMCCSTGVCGSDVDQVLVDFSADMQWLKG

RGVQVERYNLAQQPMSFVHNEKAKAFLDASGAEGLPLLLLDGETVMAGRYPKRAELAR

WFGIPLEKVGLAPTSCCGGNTSCC"

CDS 229778..231529

/codon_start=1

/product="Arsenical pump-driving ATPase"

/translation="MKLLQNIPPYLFFTGKGGVGKTSISCATAIHLAEQGKRVLLVST

DPASNVGQVFDLAIGNTIRPVTAVPGLSALEIDPQEAARQYRARIVDPIKGLLPDDVV

NSISEQLSGACTTEIAAFDEFTGLLTDASLLTRFDHIIFDTAPTGHTIRLLQLPGAWS

SFIESNPDGASCLGPMAGLEKQREQYAHAVEALSDPERTRLVLVARLQNSTLQEVART

HEELAEIGLKNQYLVINGVLPEAEAEHDALAAAIWQREQEALANLPAGLSELPTDTLL

LQPVNMVGVSALKGLLATRSEALPLPVTNILYTPENLSLSGLVDDIARSEHGLIMLMG

KGGVGKTTMAAAIAVRLADMGFDVHLTTSDPAAHLSTTLNGSLKNLQVSRINPHDETE

RYRQHVLETKGRDLDEAGKRLLEEDLRSPCTEEIAVFQAFSRVIREAGKRFVVMDTAP

TGHTLLLLDATGAYHREIAKKMGSKGHFTTPMMQLQDPDRTKVLLVTLPETTPVLEAA

NLQADLERAGIHPWGWIINNSLSIADTRSPLLCQRAQQELPQIEAVKNQHADRIALVP

VLASEPAGIEKLRELMS"

CDS 231577..232101

/codon_start=1

/product="Arsenic efflux pump protein"

/translation="MLLAGAIFVLTIVLVIWQPKGLGIGWSAMLGAGLALISGVVHVG

DIPVVWNIVWNATATFIAVIIISLLLDESGFFEWAALHVSRWGNGRGRLLFTYIVLLG

AAVAALFANDGAALILTPIVIAMLLALGFSKGTTLAFVMAAGFIADTASLPLIVSNLV

NIVSADFFGRHCCK"

CDS 232858..234003

/codon_start=1

/product="Mobile element protein"

/translation="MNKTKGCLIANFATVPTVERHIRGKWACDSCETLIQAPVPPQVI

DKGIPTAGLLAQVLVAKYADHLPLYRQERMFGRAGLEIPRSTLAEWVGACGVQLQPLV

DALRNTLLEHSVLHADETPVSMLAPGKKKTHKAYVWAYCTTPFADLKAAIYDFAPSRA

GEHARTFLGDWRGKLVCDDYAGYKAGFGNGITEIGCMAHARRKFYDLHEANKSELAAK

ALEYIGGLYEIERETKDLPPDMRREIRQTKAKPLADALHQWMLAHRQKVPDGSGTAKA

LDYSLKRWEALTRYLDDGAVPIDNNWVENQIRPWALGRSNWLFAGSLRSGQRAAAVMT

LIQSAKLNRHDPYAYLKDVLTRLPTQKNNAIDELLPHNWKPAIPNKV"

CDS complement(234254..234817)

/codon_start=1

/product="Gll1166 protein"

/translation="MNTRLFTFAGGETGVWRVVRMDAVAGAPLPGIPRLDVAAGSVSP

QPLGTKWLLRGITSNERYVVREEKDRLVAKQPSLGRAEATCAALIPIRKNPSWWGLAQ

DERRKIFEEQSRHIHIGLQYLPAVARRLHHCRDLGESEPFDFLTWFEYSPSDEPGFNR

LLAELRASVEWKYVDREIDIRLVHEPA"

CDS complement(234841..235203)

/codon_start=1

/product="Uncharacterized conserved protein, contains

double-stranded beta-helix domain"

/translation="MRAETESRIFSVDEYVRPSNGEPIRSVVLETNDSVVVVWHAHPG

QEIASHVHPHGQDTWTVISGEAEYHQGNGIVTHLKAGDIAIAKPGQVHGAMNSGPEPF

IFVSVVAPGNAGFALAEK"

CDS 235580..235801

/codon_start=1

/product="hypothetical protein"

/translation="MRLARLVSLGIARLFHVALDAVELVDKSQCHLRSADLALGLSFL

MNLRRACPATYTLDAFLPPGGAAAPPGYG"

CDS 236086..236421

/codon_start=1

/product="hypothetical protein"

/translation="MAQGGITGRLRYNGQRLRLLSIDTPETYKPRCDAELTKGREATA

RLRELVANAQRIEVVDSGQADKYDRRLVHLLIDGRDVGQTLMAEGLAVEWRPGPNAWR

ERRRHWCGY"

CDS complement(236691..236807)

/codon_start=1

/product="hypothetical protein"

/translation="MPSDSLKLFKTKKTYIKRVALGKTARGIMDNPASRIPA"

CDS complement(237037..237318)

/codon_start=1

/product="Aldehyde dehydrogenase"

/translation="MPAFEWVHVQLHQQKGMISLSPPTICNSATENYLNCVNSESGRV

RVEAKRASEEYGRVLRRYPLKPGETVERPQRGEVVSSSGVLPPLDPGEY"

CDS complement(238339..239352)

/codon_start=1

/product="replication initiation protein"

/translation="MSNAHNPQHWSQLDMDEQIRFWQGVEDGHVASFLVSPEKKSTRR

RRGEHSTKPKCENPTWFRPEHYKKLGGQLGHAYNRLVQKDRTTGEVRLRMHVSLHPLY

VRERRRAGRRYGFRPEKQRLLDAIWPVLISFCDAGKLTVGMCISRLAKELSQKDSHGK

VIPETEVTVSRLSRLIDEQVRFGVLAVSEENSWDRESRTWLPKYVYITALGFQMLGVD

LEKLDAEQQKKLRQSEERRRLIEEGILREDEEISPCAARERWYRQKTLDALRFRRQRG

AERKRANRLARYSRERQIHEMSLHILKTMPADEAYWCTTERLQQLAIQNLYQLELALA

PPS"

CDS complement(239345..239785)

/codon_start=1

/product="replication protein"

/translation="MVKANGFTESFSFDIASAMSRRDGIRKRKPPFLRRRAMNALLMA

MCFYYDPLSNKVLRSLREIALECGLATKSLSGEVSITRAIRALESLEKDFEFVACSSD

CYSTAEIFFTPKLFEFLGVFPLSLSEARLKCLAAKNSGRESADE"

CDS complement(240178..240447)

/codon_start=1

/product="replication protein"

/translation="MSQVVNGVTSSSKRPYRKGNPVSAAERQQKAVARKKATHKEVRV

FVRDKLKNQLQIMCENEGITQAAMIERLIERESAKLGIDVTTSHS"

CDS complement(240583..241143)

/codon_start=1

/product="Endonuclease"

/translation="MKKMNRTLRRCCLSGLISLSLFAPVPAVYAASIETGYSPEGTAL

QLVLKTINSAQQEIRLMGYSFTSPEVAGALVRAKQRGLDVKVVLDWKANTGRQNKASL

AAMNLLVNAGIPVRTVSQYKIMHDKVIIADGRNVEVGSFNYTRAADRFNSENVLVVWD

EPVVAQRYLQHWQTRWEMGKDWSSSY"

CDS complement(241238..241546)

/codon_start=1

/product="hypothetical protein"

/translation="MIIAAEVKRDEQGYWTHPALVRSGCETSAELSYWLRTHKLQCFV

ITMRDEAPEAFAAGFTDEAPNARGWIPEPPDDDGWFLGSVHDTGDGPVCYWFRHTTKS

"

CDS complement(241543..242193)

/codon_start=1

/product="YihA"

/translation="MAKILMMAGSTVVVLSLLGFLVLLLKGRAVTKTSPVLRSLKALG

IRPSEAEQRLCRQRVWVGNDTLMTPREQHFFRALLRHTSRTRWLLCPQVRVADIATLS

PHIRPRSRTWWQLFRMASQWHCDVVIVDIHTFAIIGAVELDDASHQKKHRIRRDILLE

EVLRQAGIPLLRDRDSESLVRKVGEFLKYRGADAAEKSITGMNSTTEHTERKENEK"

CDS complement(242249..242893)

/codon_start=1

/product="hypothetical protein"

/translation="MFRYFILRPEQQLFCYLYGCALALVQMVLFSPVSRASGFYLVAL

SVALFWAGLALYTRHIDRMRKPEVSPLVSIRDGIQVVAEVPRHEKARLEWEILRDDEM

FRQQRCELTGLTGRVISRGLLYTPAVMLVGIGILAWGSPQDAIRLINALRNMPAAELV

HQIGFVLCHFLQISVISVLIADVVAGRGLPNVFRRALLDRLPAEFCLIRRGTER"

CDS complement(242943..243545)

/codon_start=1

/product="hypothetical protein"

/translation="MTMTEKIIALVDYENIGTLESVRLSRYERLILFTGPQQEFIRFP

AVTYAGDISVSVFQVANVSKNNVDFHLVFELGRLSATAPEEMIFHIISNDKGYDGVIA

ELCRAGRRCCRISSPCSAEKPKAAPMVISNDEVLHLTDKVQSLSKKSAGNRPANTSSL

MNYIKSLSGNTKGMALYGRVKEELIRRGVIAVYEKTVVWR"

CDS complement(243706..244299)

/codon_start=1

/translation="MSEEKRPVLSLKRKPAENSTTSAEATPAPGVVRRKKVVVVSSPP

AWKAKKEKLEKVKQAAEAAARNAAPVPVKAVKTPPPVHYLRLLPPEQAIMTLKAFWPQ

LFDGNSPRLLATGMREQLFADIVNRDLPLSHKQVIKCLKSLTRSAGYLSRMKVGASRY

DLQGNAVATVTAEEAQYASERMMKELLRAERKMSQTL"

CDS complement(244455..245180)

/codon_start=1

/translation="MRAQYTFPGATDSWLQRLLTWTPGQTDMIKTVALVLMVTDHTGL

LLAGNNEVMRLLGRGCFPLFGLAWGMNLARHAEIRQSQLNSLWGWALVAQVSFMLIGY

PWYTGNILFAFAVTGQVLRWVSQPSWYYTLPAAGLLVTWIPLSTGSYGMAGVGMLTAS

WLLCRAQHAPERLGYGVLWALMVLLMNMHDVSESVAGLAIALLTLMVCSSAGERVKRF

WPRQFFVMFYAVHLAVLGIVVSM"

CDS complement(245260..250518)

/codon_start=1

/translation="MLSFSQVKSAGSAGNYYTEKDNYYVIGSMEERWQGKGAELLGLE

GKVDKQVFTELLQGKLPDGSDLTRIQDGVNKHRPGYDLTFSAPKSVSMLAMLGGDKRL

IDAHNRAVTVALNQVESLASTRVKKDGVSETVLTGNLIIARFNHDTSRAQDPQIHTHS

VVINATQNGDKWQTLASDTVGKTGFSETILANRIAFGKIYQNSLRADVESMGYKTVDA

GRNGMWEMEGVPVESFSTRSQELREAAGPDASLKSRDVAALDTRKSKEAIDPAEKMVE

WMNTLKETGFDIRGYREAADARAAELARAPAAPVNTDGPDITDVVTKAIAGLSDRKVQ

FTYADLLARTVGQLEAKDGVFELARKGIDAAIEREQLIPLDREKGLFTSNIHVLDELA

VKALSQEVQRQNHVSVTPDASVVRQVPFSDAVSVLAQDRPVMGIVSGQGGATGQRERV

AELTLMSREQGRDVHILAADNRSRDFLAGDVRLAGETVTGKSALQDGTAFIPGGTLIV

DQAEKLSLKETISLLDGAMRHNVQVLLSDSGKRSGTGSALAVLKDSGVNTYRWQGGHQ

TTADIISEPDKGARYSRLAQEFAVSVREGQESVAQISGTREQSVLNGLIRDSLRQEGV

LGEKDTTITALTPVWLDSKSRGVRDYYREGMVMERWDPENRTHDRFVIDRVTASSNML

TLKDREGGRLDLKVSAVDSQWTLFRADTLPVAEGERLAVLGKIPDTRLKGGESITVMK

VEEGQLTVQRPGQKTTQTLAAGAGVFDGIKVGHGWVESPGRSVSETATVFASVTQREL

DNATLNQLAQSGSHLRLYSAQDAARTTEKLSRHTAFSVVSEQLKSRSGETDLDTAIAQ

QKAGLHTPAEQAIHLAIPLLESQDLTFSRPQLLATAMETGGGKVSMADIDTTIQAQIR

SGQLLNVPVAHGYGNDLLISRQTWDAEKSILTHVLEGKGAVAPLMDRVPASLMTDLTA

GQRAATRMILESTDRFTVVQGYAGVGKTTQFRAVMSAISLLPEETRPRVIGLAPTHRA

VGEMQSAGVDARTTASFLHDTQLLQRNGQTPDFSNTLFLLDESSMVGLADMAKAHSLI

AAGGGRAVPSGDTDQLPPIASGQPFRLTQQRSAADVAIMKEIVRQMPELRPAVYSLIE

RDVHRALTTIEQVTPEQVPRKEGAWAPGSSVVEFTPKQEKAIEKALSEGKTLPEGQPA

TLYEALVKDYTGRTPEAQSQTLVITHLNKDRRALNSLIHDARRENGETGKEEITLPVL

VTSNIRDGELRKLSTWTAHKEAVALVDNVYHRISKVDKDNQLITLTDSEGKERFISPR

EASAEGVTLYRQEKITVSQGDRMRFSKSDPERGYVANSIWEVQSVSGDSVTLSDGKLT

RTLTPKAEQAQQHIDLAYAITAHGAQGASEPYAIALEGVAGGREQMASFESAYVALSR

MKQHVQVYTDSREGWIKAIKHSPEKATAHDILEPRNDRAVKTADLLFGRARPLDETAA

GRAALQQSGLAQGSSPGKFISPGKKYPQPHVALPAFDKNGKAAGIWLSPLTDRDGRLE

AIGGGGRIMGNEDARFVALQNSRNGESLLAGNMGEGVRMARDNPDTGVVVRLAGDDRP

WNPGAMTGGRVWAEPAPVAPVPQAGADIILPPEVLAQRAAEEQQRREMEKQAEQTARE

VAGEARKAGEPADRVKEVIGDVIRGLERDRPGTEKTTLPDDPQFRRQEAAIQQVASER

LQRERLQAVERDMVRDLNREKTLGGD"

CDS complement(250518..252830)

/codon_start=1

/translation="MSFNAKDMTQGGQIANMRFRMFGQIANIIFYVLFILFWVLCGLM

LMYRLSWQTFVNGCVYWWCTTLGPMRDIIRSQPVYTIQYYGQSLEYTSEQILADKYTI

WCGEQLWTSFVFAAVVSLVICIVTFFIASWVLGRQGKQQSEDENTGGRQLSDKPKEVA

RQMKRDGMASDIKIGDLPILKNSEIQNFCLHGTVGSGKSEVIRRLLNYVRARGDMAII

YDRSCEFVKSYYDPSLDKILNPLDSRCAAWDLWKECLTLPDFDNISNTLIPMGTKEDP

FWQGSGRTIFAEGAYLMREDDDRSYEKLVDTMLSIKIDKLRAYLQNTPAANLVEEKIE

KTAISIRAVLTNYVKAIRYLQGIEKNGEPFTIRDWMRGVREDRPNGWLFISSNADTHA

SLKPVISMWLSIAIRGLLAMGENRNRRVWIFADELPTLHKLPDLVEILPEARKFGGCY

VFGIQSYAQLEDIYGVKPAATLFDVMNTRAFFRSPSREIAEFAAGEIGEKEILKASEQ

YSYGADPVRDGVSTGKEKERETLVSYSDIQTLPDLSCYVTLPGPYPAVKLALKYKPRP

KIAEGFIPRTLDARVDARLSALLEAREAEGSLARALFTPDAPASGPADTNSHAGEQPE

PVSQPAPADMTVSPEPVKAPPTIKRPAAEPSVRTTEPSVLRVTTVPLIKPKAAAAAAA

ASTASSSGAPATAAGGTQQELAQQSAEQGQDMLPAGMNEDGVIEDMQAYDAWLADEQT

QRDMQRREEVNINHSHRHDEQDDVEIGGNF"

CDS complement(252959..253648)

/codon_start=1

/translation="MTISVPVRSLDTHELMPGDIYPHPAAGGIYSLSHREVCSLFSDH

IMYICSALVILLLVFIAGVFFRKIMTSSVERKRNTASVISDAKPEVQSKPEYFHLSIC

VEGPASMDDSALSSLASRVVREAENLYIPGVNRRDSADDVFAWSVQTQFPSDRLFGTR

IFPSTGWFERPDMIKHPVKCYCVCLQYTTETLPSTLYALVDEAGRQVAKSYIKGGHCP

GSEGYSFCITS"

CDS complement(253841..254572)

/codon_start=1

/translation="MQLNKLMTVMVVSSALVLSGCSAMGTAIKKRNLEVKTQMSETIW

LEPSNNKTVYLQIKNTSDKDMSGLQAKIASAVTSKGYQVVSNPDTAGYWIQANVLKAD

KMDLRESQGWLSRGYEGAVTGAALGAGITAYNSSSAGATLGVGLAAGLVGMAADAMVE

DVNYTMITDVQIAERTKTQVQTDNVAVLRQGTSGAKVQTSTETGNQHKYQTRVVSNAN

KVNLKFPEAQPVLEDQLAKSIANIL"

CDS complement(255448..258270)

/codon_start=1

/translation="MLEVYAIAGGDWLRGNLNAIAAFMGTGTWSTIEKMCIAISVLIV

AGSWVKKHNVMDLIGWVFSLTLVSMLVVIRTPVQIIDYSNVAQVYEVDNVPIGLAIPA

SLTTRVGNALIQSYEMVFALPDSVTYSKTGMLFGSNLVAKSTDFLSQNPQITTLFSDY

VQNCVMGDIFLNHKYSFEELLNSPDPYTLIFANPSPLRGVFDKNNQFQTCEEASRDLK

SALALDTQTGGKTWNYYVRQLFGGKPNPDVLFSQMIGDSYNYFYSSGQSAGQIIRQNV

TMNALRSGIQSYAARSGDTASLVNMANTSSLEKQRLAQATMGHQALRALPLMQTVIMG

LMIGMFPIMVMAAMFNMMTLQVLKGYVFALIWLQTWPLLFAILNSAMAYYAKQNGVPV

VLSELSQVQLKNSDIATTAGYIAVMIPPLSWGIVKSMGAAFSSAYSHFSSSGLSATSQ

AASGVVDGNYSFANMQMENVSGYSWGTNSTTSFGQMSRQLANGGMSTQTRDGSMVWDS

GGAMSKLPVDINVGRQIASAQQQMARESDVQAESALHGYNSSVTSAWNSLQQFGTNKG

NSASTTTGADTTESSQDSMARSKMMNAVDSYAKANHISRDEAFGQLMDKSVRGDVSGE

AYVGARGGVGFELFGNGGKAEFGGRMSARGTASSGSQDNTTESGRQSNADNHDTSSQA

VKDFRESSDYFINQKSSQSGNITDNNASSRVDQFSASLASAKNSYDQYTTSKTRSHEY

SEMASRTESMSGQMNENLTQQFANFVQKNVPQNAEAILTDTSSPEIAAQREQLAREFV

KQQVEPKVDEAYQEGRRNIGANMPSVSEGKGSGTVYADYNSHGDSIDEMTKNAGIKND

VHQSVEHMVSENQQAHKDRQDSIHKQEDDVQNEHTRLKNHHNLEGNKFEKEYNDKKAE

QRALPGADTRDELLAKAQEFERKHKP"

CDS complement(258270..259637)

/codon_start=1

/translation="MRIKLLSLFCGAVLGLSFSAAADVNGDMNNFFNKLGFASNTSQP

QVWQGQAAGYASGGSLYARTQVKTIQLVSMTLPDINAGCGGIDAYLGSFSFINSEQLQ

RFGKQIMSNAAGYFFDLALQTTVPEIKTAKDFLQKMASDINSMNLSSCQAAQGIVGGL

FPRTQVAQQKVCQDIAGESNIFADWAASRQGCSVGGQMDKVQDKASDKDKERVMKNIN

IMWNALSKNRLFDGNKELKEFVMTLTGTLIFGENSEITPLPARITDQDLIKAMMEGGT

AKIYHCNDSDKCLKVVADATVTITANKAFKNQIRALLSSIQNKAVADEKLTEQEKGFI

SSTTIPVFKYLVDPQMLGVSNSLIYQLTDYIGYDILLQYIQELIQQARAMISTGNYPQ

STMDMIMENLNQASVQIAAFQSRVQVQQDALLVVDRQMSYMRQQVSARMMTRYQNNYH

FGGNQ"

CDS complement(259627..260070)

/codon_start=1

/translation="MNKYIRSTGLYAFLFPASLKAPGQTAAEKIEQLKPEFIHRERRL

EIYLELFIVFLTAGALLLWIMRLLFNLCVDDWIASGDLRVKDLWNIMMYAIPYALIAV

GVGFFVAGVTLAIRNFFSYHLKSLFILRNDRVKKNAVRNGGQDAN"

CDS complement(260116..260673)

/codon_start=1

/translation="MKNSLMNLAKTPLAALALSLALAGMAHAGTLDEVKSLWDPRGIS

GTETASPQSDTPQAVSGASQPRWLRLSNGKQVDLRDWKVVLFMQGHCPYCHKFDPVLK

QLAGQYGFSVFSYTIDGQGDDAFPEALPAPPDVMQTFFPNIPVATPTTFLVNVNTLAA

YPILQGATDAQGFMARVDTVFQMMH"

CDS complement(260645..260884)

/codon_start=1

/translation="MRNIRFPDLDITGMWVLAVGVFFHLIARLVRKQPELAVQAGEIF

GLGMVVFGGYRILNALIAEAEKEEKACEKQPDEPR"

CDS complement(260895..261647)

/codon_start=1

/translation="MRTIHAAMLAGCLCCALMPAFAKDAGWQWYNEKLKPRDEDRKAV

PAAPPPQMDILEKLATLQAATKRSLYEAILYPSSENFVKYFRLQNYWTQQAGLFSMSA

KKAMLENPELDYNLQYSHYNGTVKNQLAADYAEQRQAISTLAQHYGVMFFYRGREAID

GQLVQVIKNFRETYGLSVIPVSVDGVINPMLPDSRTDQGQAEQLGVKFFPAMMLVNPK

SGQVKPLSYGFISQDDLAKQFLYVSSDFKPNF"

CDS complement(261668..261994)

/codon_start=1

/translation="MEEKKAYGLVMVFVGVFVFLLVSIMSYSLWRDRQVNAFMTTNRA

WGIQCDTVSQAAWVIRDGKRVDLQINHLPLYCSGYRFEARDDAGKVQRQLDKYSVYQH

LSRQSH"

CDS complement(262007..262273)

/codon_start=1

/translation="MQGKAKMNRYITIEKFIDILNEENLPQEHHVMVLAVLADISLHT

DRFLINSSELVQMAAQYSPAFQKLPADRQAFISSVLSMPLFLIM"

CDS complement(262233..262487)

/codon_start=1

/translation="MKINFHKQPAIDFVLRLTLTVIIISPVIYFSWDAVKGTSGSDYV

ESVVFILMAGFAMSVCYLFFSALEKVINARKSKDESIYHD"

CDS complement(262519..264474)

/codon_start=1

/translation="MKTVISVLTAHFLVLSAFIWLASPACADSGSDYKAGSDFAKQVQ

GNGLNSLKNFSGEQNLPGYTANPNQTKYYGGVTASGDSSLKSDSALEFSQGDTGKAVT

ESFTNRPPDQISQDAPFIQAAKDTESRADSIVGDTGQPCTAQVVNRSEFTNHTCERDL

QVENFCTREATLKDNATTQKVNRTYQQVVTLNYARSTRQWSGNLTIPTNGRLLNASVD

GEPLVIPWIEECDSEGKVRDSCKSAVSESLTLFERTFPIDVISWPRSESMCSGGQNTH

CTKYTYDGKGKIHQSFGVDKAVTAGQNFSVSKTSRTVSSASQKPVQVTVTLVMEETET

VYAPEVVWVESCPFSKDEGKKTGEECISPGGTRTITLGGRDYSFTEACWKYKDTWLTQ

PADNGSCESLMKNTACTLSSRQCAFSSEEGTCLHEYATYSCETRTSGKQMICGGDVFC

LDGECDKATSGKSNDFGQAVSELAALAAAGKDVAALNGVDVRAFTGKAKFCKKFAAGF

SNCCKDSGWGQDVGLARCSSEEKALAKAKKDKLTVSIGEFCSKKVLGICLEKKRSYCQ

FDSKLAQIVQQQGRNGQLHIGFGGASSPDCRGITVEELQRIDFNMLDFTNFMDDLMKN

QKIPENDVLTNKTKERIKEIMSQQSVQ"

CDS complement(264533..265180)

/codon_start=1

/translation="MNKGITGLAWGLSLLCTTASAADNINTPGDQQWLKQQENLSEQL

RQHPDKQLQQELEAQISRNPLPKSDRQFIDNLVSQQKAANQEKPAEGALYFVSFSIPE

EGLKRMLHETRQYGIPATLRGFVNNDMRTTTDAVLHLVKDGVTDGVQIDPTLYTQYGI

RSVPSLVVRCQAGYDVVRGNIHVKQALEKVAQTGDCAQLARQILDAHSNAAGSQP"

CDS complement(265259..265435)

/codon_start=1

/translation="MMEKEQNQCCQQPLLHQDVSPTTSRVTFSTDKKVLTAEQYRDWQ

YIGATLREKMQSNN"

CDS complement(265456..266445)

/codon_start=1

/translation="MKAMFFLICIAFSTFSGRAAADPSCEGRFVNPITDVCWRCIFPL

SLGSVQVGKGDLPDTSNPGSPLQLCPAPPPLFVRPGLAIGYWEPMAMTDVSRSPGCMV

NLGGFSINLGKTGMGTARKDDKQVNGAFYHVHWYKYPLTYWLNIITSAGCLEGGDMDI

AYLSEIDPTWVDSSLTTILNPEAILFANPIAQGACAADALASAFHMPLDVLFWCGGSH

GSLYPFNGQVSNESSPLQSSVLVSERMAFKLHRQGQIMESIGKDKAVCYEYPSPIIPK

ERWRYQMVNMYPDSGQCHPLGRSVMRWEAGKNPPNTRKNYGYLMWRKRNCVFL"

CDS complement(266442..266843)

/codon_start=1

/translation="MPMILALIILLTMLLLTLMWSTIRKQENVIRELSTRESWLEREV

ERLRTDNEKLYRWLRYNGEKVIDEMTCELDALSAVRQTNRDSYRRQLYLFDCLSLMVW

AGMEGAEDSTREKVRNKIDALRLVLREKTDE"

CDS complement(266878..267513)

/codon_start=1

/translation="MIRLKDCLLAACLLSPLTQAADLGTWGDLWPVREQDMLQLITQR

LQSLQSSGQWDQTMDAFKQRVIENSQRPAPVEGIKRAEKYEQRWFDPSIRLTEDLKDN

EGRVFARKGEVVNPLKTVPFVQTLYFINGDDADQLAWMKRQVPETLMSKIILVRGSIP

DTSAALDSRIYFDQNGVLSKRFGLTAVPVRITPAPSGERLNIETFPPVPHP"

CDS complement(267513..267902)

/codon_start=1

/translation="MKNNTEQPVSTTSHTFRRIPVIVALVLFALATSAFLSRVVLEYS

TPRVVAFDMKKTLDSFMDSVSQKQLTEAQSKALSDRFNDALEKSLAEYQQQHHVVILV

SPAVVQGAPDVTRNIQHDIARRMKGEQ"

CDS complement(267902..270541)

/codon_start=1

/translation="MSNNIIDAVTQTVNSLVSALKLPDESAKANDTLGSMNFPQFSRI

LPYKDYDSATGLFINNKTVGFMFEARPLPGADKSIVATLEHLLRSKLPRGVPVSFHLV

SSKLVGNDIDYGLREFRWSGKQAKKFNAITRAYYLRAAETQFPLPDNLDLPLTLRNYR

VYISCCVPRKKNSATQIVEMENQIKILRASLGGAYIPTRILDAAGLVELMRELINPDP

HEMYRVPYKLDPYQDLNYQCVDDSFDMKVTAGHLKIGRLGRDGKECVTRVTSYHLEKD

PEMAFLWSNADNYANLLNPELSISCPFVITLTLMVEDQVKTQNEANLKFMDVEKKSKT

SYAKYFPNVIKEMQEWGDIRQRLATNQTSLVSYFFNITTYTADNAEASLATEQQVLNS

YRKGGFQLIPARYHHLRNFLAMMPFKCGEGLFKELQAAGVVKRAETFQVANLLPIVAD

SPLAPAGLLAPTYRNQLAFIDLFYEGMNNTNFNMAVCGTSGAGKTGLIQPLIRSVLDS

GGFAWVFDMGDGYKSLCENMGGVYLDGDTLKFNPFANVLDDAHFDMSAERIRDQMSVM

ASPNGNLDEVHEGLLLQAVQAAWLSKRNHARVDDVVQFLQDAKDSDEYADSPTIRGRL

DEMIILLDQYTVNGIYGDYFNSDKPTLHDDARMVVLELGGLESRPSLLIAVMFSLIIY

IENRMYQSPRGLKKLNVIDEGWKLLDFKNEKVGQFIEKGYRTARRHTGAYITITQNIV

DFDSPTASSAARAAWGNSSYKAILKQSAKEFAKYNQLYPDQFSKLEKDMINGFGSAKE

QWFSSFMLQVEANCSWHRLFVDPLSRAMYSSKGPDFEYMQARRQEGVDIHDAVYGLAC

RNFKDEMAELESRIPVNDMEDKQ"

CDS complement(270613..271005)

/codon_start=1

/translation="MMLFLRKYGFLFTPPVYWLIYLGARLTYNGLAGDFSADFLQGIR

HEGVYIICSLCVFYFFCLEVEVGDGEFSANILFLIVFFHVVLLFITLLICLWQGEVLL

DSIFMGQIIASMCLLFFIPVLSIIRRSF"

CDS complement(271019..271165)

/codon_start=1

/translation="MALQTNNVTSSATLTFGDAKKMASECKDKKEIKAVREKQLNALN

DMSK"

CDS 271196..271465

/codon_start=1

/translation="MNNTLQLFWSGGLHIAKIFILNKKNIQLKQKKEEPKKYHIADQN

VSILAFLFHRPFLLNPESALQFLVVFPLFHPYRSIQFINQHFILS"

CDS complement(271452..271763)

/codon_start=1

/translation="MVAIPEKFNLRVNRGDTFIYCALIAVVGFSGMFVISIHSFSMDE

LEAGRHWKDDCRTLEVNMPTGAFTSPVNKLDCDGIIINVPGGQYYSYIHQWELYKANS

K"

CDS complement(271923..272234)

/codon_start=1

/translation="MAEYQLSFDGFVRLFFAMAIIICGYGYVIIGVFIRDTLAYILML

FGLTGLSALFSLMVFMDKNMWAGSPDLTLSVITGFFSATVIFYMFYVLIYYFDRMGAG

E"

CDS complement(272235..272453)

/codon_start=1

/translation="MHAFIFLVLMTPSSGGGIWDITPMPNTDVCIKMLRVLEARGGRG

YGSNTGECIEIKTREPLSSVIETLSEGG"

CDS complement(272533..272766)

/codon_start=1

/translation="MDELFKWTMALVFLLTGYSLLFSSDSNTVPEVLSHHWKNDCRLL

ETNIDKGFSLQRKIDFSVVMLLKMSARLIMTER"

CDS complement(272771..273181)

/codon_start=1

/translation="MTDSMKYLWLLLREDSSYIFMLILIVGSAVIVSSFFQRVITSWW

GKILSVILCMVSMITTATGVLDPESTYKQIINRKENIIYTLRNCRVSAFEAQQAGFLA

KAKDAWSCPDGVTRYMDVRYRDKAEINKLSTEGK"

CDS complement(273313..273897)

/codon_start=1

/translation="MKELMLLIPLGSALLLTGCAGTETEFQCNATTSDTCMTMEQANE

KAKLKEVRSDAKPAAAGLPQLAEGNFRTTSVNSFPLPPQPNLNRARVAAERERARAVY

LNNPTAKNEAIYFETEKRLQSLPVVKMSLPVVASTFTSPSTPPGNYPRPLRKGEETTS

LWIAPYVDSDDVYHQPATVLFVVKPSAWGQPRLN"

CDS complement(273920..274564)

/codon_start=1

/translation="MIRVSSLRAQRRRGKIMAKSLITAVLSALRWAHWLVKCAVVYPL

AMMMLLVVLVFLTGPYTFGQTLVKTVESVQQXGYVIQDCTGPKELNGDAVNAPLPPVL

QEDCTTVSTDAAGYAAYIDQSYTHNILWLWLLMAVVFTGIAVVFRRTPVRRVIIRKNG

VVVGEAGSGSSGLILARSDVSQIKRVYYEMSPDEGDKFVRAIKENTQIRMNLKK"

CDS complement(275933..276673)

/codon_start=1

/translation="MKKINPLFISGCLLLVAPAMSATLSGTLEPTVVPLTNGGQANIA

VSNTDPNLFTVPGDRITAINSLDGGLTNQEQTDSGGAILATVSKKPFTFIVETERGLN

FSIRAVPRAGSGRTIQLVSELAGTPGPAKAWEESNPYESLLVSLNRAVRQGSVPDEYQ

SVPVTSEVLQVPAGLRATADRVWVGHHLKVVRYSLDNVSLSARMVRESDFWQPGTRAV

MFSTPAGLLTAGGRMQIWVTTSDEGVKR"

CDS complement(276660..277226)

/codon_start=1

/translation="MEHGARQNTTRVIAVGFISLGTLLTLSLATNIIQGINNYRLQTE

QKVAVTPMLFRAPFAVSQNQADASYIEQLGLSFVALRLNVTPETVDAQHQQLLRYVLP

ASQNSLKVHLAEDAKRIKDNNVNSTFYMTSMRAWPAENRVDIRGELKTWIGDSKPYSE

IKSYVIQFSRVDGVSWLARFGEINNEKN"

CDS complement(277246..277623)

/codon_start=1

/translation="MASRLFPPLXPSVCRSPVTDRKHVMTGDELKRYRFPETLTNQSR

WFGLYLDELIPAVICLGWGFWTSKFIFGIASAVLVFWGIKKLKKGRGSSWLRDLIYWY

LPTSLLKGFFHDVPDSCFRQWIK"

CDS complement(277995..278165)

/codon_start=1

/translation="MLLKLDEETNRRLIKAKDRSRRSKTAEAYLRLKDHLERFPDFYN

SELAVPGGEKEE"

CDS complement(278334..279053)

/codon_start=1

/translation="MCAKDRSHIAVRSQTDSIHSMREMLNISSCPAFIRDSRGEVIHT

SPTFDKIFLTSGGTGAFDTPRAPGSWFLDLSLEIKLELMQSELKSFSEGSAVLVKNIW

LAGSLWTVFIETFSINEDTYSKWVFINEDEPMPHPSMEYNDFSIKMQRYIERIQRSAK

SDWAIFNLYAVGFSHASISKITGVGVQTSKNTVSKIKKELSFDNRDYIIMSSIYTLSY

GKFISNVVSILNDGVNFLLNQ"

CDS complement(279253..279669)

/codon_start=1

/translation="MLDAVFNKGTDMARVQAYASDEVAEKINAIVEKRRAEGAKEKDV

SFSSVASMLLELGLRVYEAQMERKDSGFNQTEFNKILLENVMKTQFTVSKLLAIGSLS

PHVTGDERFEFRSMVSNIREDAKDVISHFFPEQEEE"

CDS 280060..280545

/codon_start=1

/translation="MKGAAFGIILSLIPLSSMAADCFDMAGRDYKIDPDLLRAISWQE

SRFKIDAIGRNPVTGYGSGLMQIDSQHFNELSRYGIKPDHLLSDACLNIYTGAYYLAQ

AFKKWGVSWDAVGAYNAGFKKTPRQAARRYEYAKKIHYYYTAIKASKRTSSKDQKIAM

N"

CDS complement(280578..280739)

/codon_start=1

/translation="MYDFKRHLLALKRGGVSKFLRKNLILMHREGGYLLFHFDSDADY

YPGIDAVDF"

CDS complement(280940..281914)

/codon_start=1

/translation="MLVYALAANGSQTGEGYIQGQVQQVPAQSQNRGPGNSTGDGXKI

KQVKENNMRLASRFGLTHSIRQERPLTNDELVKVVPSVFSEEKHNSRSDRYTYIPTIT

LLDKLREEGFQPFFACQSRVRDEDKRGHTKHMVRLRREGANKGTEVPEIILLNSHDGS

SSYQMIPGMFRFVCTNGLVCGTSFGEIRVPHKGDIVGRVIEGAYEVLGIFDKITEGVD

VMKSITLTKEEQRLFGQAALTYRYEDENKSPVSIEQIIHPRRYEDKKDDIWTTYQRVQ

ENLIKGGLPGRTEKGKRTTTRPVKAIDGDVKLNKALWLIAEKFRTLKG"

CDS 282155..282274

/codon_start=1

/translation="MPAVLRRHGESGAAGTAAASAAVDFDLRHQTLAVRPESA"

CDS complement(282594..283007)

/codon_start=1

/translation="MISDANKAVNDLASIVPLLGGSSSRKDYEEARKLVEYLLEHDPD

SPLVDMLTARIDAWEDNAVEFEEFNTRFEAGKNGVSLLRVLMQQYGLSQSDFENEIGN

KSLVSRILSGERSLTFDHMRALANRFQIPVSMFVD"

CDS complement(283008..283286)

/codon_start=1

/translation="MKYKTLSQVHTEAMNDHEYSAAFEAEEASELLRETLATWRKEAG

LTSAQVAERMGIKAPTISRMEKNASRMSIQTLVRYARACGVNFKIQQV"

CDS complement(283276..283596)

/codon_start=1

/translation="MFTVIFHDEAEKEFTALPAAIRAKMARLLMKLEANPRQLREPDT

KPLGNGLFEIRTMGADIARGIWVYQSGERIFLLRIFIKKSPKTPPAEIDQALRRLEEM

QNEI"

CDS complement(283677..283892)

/codon_start=1

/translation="MKGKIQQGAGNAYFAGMQWRGITLGHADAAALNQFDIDISKAQA

PEARTWLLQNKAEFIALILGIEVVKVG"

CDS complement(283912..284124)

/codon_start=1

/translation="MKVYGHQHSSEAFFRENSPSCFEPKAEKVIAFHGKDASYAYNVL

ADLKAAGASYTRRNIIADMFRRLGVK"

CDS complement(284185..284541)

/codon_start=1

/translation="MSTQNVNEKPSTKESTTLRTDGFVRNIHSRNPFDVIRADVVLER

IEKKAGRSCGMHYELYQAHLLGGALDYLDALPLKDRPVLMGAAAKRGYLLTLAEEERA

LEARDVLMSELAANDC"

CDS 284943..285062

/codon_start=1

/translation="MPAVLRRHGESGAAGTAAASAAVDFDLRHQTLAVRPETA"

CDS complement(285177..285527)

/codon_start=1

/translation="MKKEISRNPSFTPSPKLRAHLNSHREGVTERLNNIFDRYAHLVR

VCALPLDDDETQVLLNVLSGSVVEPAFIEYLAQEIRDSDDYLEGIPAAKSLYEKCQSA

TYPQLLATVERLER"

CDS complement(285524..285658)

/codon_start=1

/translation="MGSTLSCGASEDDLASVARKWYRQFVRERRKFLMMSGQYSEDNP

"

CDS 285719..285847

/codon_start=1

/translation="MDITATLGCVKTENFAGFTGFYREVHAGSPLAHPRLRFASLS"

CDS 286725..287804

/codon_start=1

/translation="MLMQHIGVGYFGYYRATAYAMKHSLMPEIAKLRMKALNFWDKHG

IRAAADAFDVSTRTLYWWRRLLRTGGPEALIPRSKAPLVRRSRHWHPDVLKEIRRLRT

ELPNLGKEQIFVRLKPWCEARHFTCPSTSTIGRIIAGAHDKMRMIPVRLSARGKARLI

KKRSVKPRRPKQYRPVKTGELIGMDAIELRMGDLRRYIITMIDEHSDYALALAVPSLN

SDITSHFFSKATKLFPVAIRQVVTDNGKEFLGNFDKTLQEASIKHIWTYPYTPKMNAT

CERFNRTLREQFIEFNELLLFEDLNLFNQRMAEYLVLYNSKRPHKSLELMTPVDYILR

ESKNCNMWWTHTPPCKLHGKRPYWC"

CDS complement(288131..290413)

/codon_start=1

/translation="MQIEFIGQPDVELAFESLGNERGRNKAQHIEVLSIHKEGDITSA

NVFVPDGKLVHFENYIQDYLTEKRRADGVSADHKSLINTLSAIRLAEIKSLWTDDLSL

LPSDPDEAFWWEVWLPVRGNRNAVVTDFHRISHATGCQVSEHKVDFPERTITWMYGSQ

SQFSQARLVLNCVAELRRAKDTAEFFEGLPALEQQLWVDDALRRLQVPSPEDNVPYIC

LLDSGINRGHAMLAPVLHQQDMHTVNDAWGVNDTANHGTGLAGVAIYGDMIDALSSTD

AIEVGHRLESVKLTPNYGANVGDAKQHAYLFSSAVTRPEILNGQRKRIFSSAVTATAY

RDFGRPSAWSSMVDSLAVDALAETPFPRLFVLSAGNIVDRDHWGNYPASLSVNQIHDP

GQSWNALTVGAFTDKVELNEPEFIPVADQGALSPFTTTSMGWEPVWPFKPDVVFEGGN

AAANTEFVDNFASLELLTTSASSHRQFWTTNATSAASALCARMAARLMAQYPEYRPET

IRALITHSAQWTPAMLRMYPARNKSGFAQLIRHCGWGSPDVERALWSVKNSLTLVAED

SLYPYRKTRDGIKTRDLNLHALPWPLEQLQELQDTQVELRVTLSYFIEPNPSARGSSS

RYHYPSHRLRFAMKRQTESLDEFKTRINAAAESEESEHGTTGNDDNWSLGATQRHKGS

LHQDIWRGAAAELASCGYLAVYPAQGWWRTRGALQRFDSEAKYSLVVSIHAPEADVDL

YAAVETLVENMVENPVEIMG"

CDS complement(290619..291659)

/codon_start=1

/translation="MASAEQLKALIKSHISRDDGHFYSVAMQVAAHEAKQGHGRLAEE

LLTLLDKAKAKLANDKSGKLVPLSNAAKNRTDLGNLLIVSQPEYRLADVVLDHSAHSQ

LQRLIREQRMMSRIKEHGLSPRRKVLLVGPPGTGKTLTASALAGELGIPLFQVRFDAL

ITKFMGETASKLRQVFDAIADIRGVYFFDEFDAIGSQRSLTNDVGEIRRVLNSFLQMI

EQDNSSSIIIAATNHPEILDYALFRRFDDVIEYHLPTLEQALDLIKSRLGAFAPKPFR

KNGLEKQVAGLSYAEICRAVDESIKDALMSDRMQVDLVILKQALEERRLISSKLSLNE

KNKLKHDGTNQR"

CDS complement(291829..292638)

/codon_start=1

/translation="MQIAQIPRSTFYYHLKALKSSDKYEEIKSRIIEIYNENCGRYGY

RRVTLALRQEAGRINHKVVQRLMNLLSLKAAIKVKRYSSWRGALGETADNVLKRNFKA

TRPNEKWVTDVTEFAVNGRKLYLSPIIDLFNNEVISYSISERPAMAMIDEMLDKAFAK

LDKKSTPILHSDQGWQYRHRWYQYQLKASGVVQSMSRRGNCLDNACAECFFGTLKSEC

FYLNKFNNIEELKTALSDYIHYYNNQRISIKLKGLSPVEYRLKVCSHPVRT"

CDS complement(292695..293204)

/codon_start=1

/translation="MSRRKYTFEQRLEVVMHYLATDEGYRLTSARFNVPRTQVRLWVA

AYDVYGEEGLKPRDKGVSIDPEIRVEAVKAVLTGQISQTQAAAKFNVAGAASVGKWMK

VFSEHGEQGLRSLRVGKKRALHMIDDPVALEVALERSKDKHIQELERKVRSLELRVLY

LKKLKALVR"

CDS 293255..293407

/codon_start=1

/translation="MKINYGAFCFLPHNTVTNHLHVMRQKASSATSIIPGRCYFLPGI

STLYAA"

CDS complement(293404..293778)

/codon_start=1

/translation="MRYWLLVILMNVLFWLIVKHVAPEWYGSPGYYAGQIICTLIAAV

ITWRDPPDWLDGILLGVIKFGVLIAGIIFFVWGIGYKLAFWGQHDPNTIYASANDVIL

PNLALGTGVLIAGWVYIRYLKR"

CDS complement(293834..294082)

/codon_start=1

/translation="MEAGQAAPEEVMSRWVAGSGYAVCVDFLGQKQIQRWSDERKAAV

RRRNMQARINRVAPLFADELIERELAARPEYFNGKSAR"

CDS complement(294157..294885)

/codon_start=1

/translation="MIPSSSALVSLKPARQAALQAIMTVEEARQRGARLPSMPHVRTF

LRLLTGCSRINSDVARRIPGIHRDPKDRLSSLKQVEEALDMLISSHGEYCPLPLTMDV

QAENFPEVLHTRTVRRLKRQDFAFTRKMRREARQVEQSWLLRQNLLGQAVTELNFQSP

ETVCTWYTRWSDEFDAAELAAPFWRWQSRFASLKELDWLRISGEPLYAVMYEIPFIVR

ETPEHIRVAERWQVPNKLADRSGV"

CDS complement(294882..295313)

/codon_start=1

/translation="MKTLLTLDVLKTMSSDELEDYRAAGEDFRRELSHAVMRDLTSPS

SWSVNAEYRCEFGGFFPVQIRFTPPYGHFDVAVCSPGELNPRWIVVFVTRDGQPFSVV

RVMDAFNPELITHTLDLIECLDAGGYSFASIISTLSQEGAQ"

CDS complement(295358..297415)

/codon_start=1

/translation="MSATESKVKTAPKTSKKTLKSAEAEALKVALDAAQVEYVPVTAL

VKSPLNVRTIPYPAEKVCSMADSIEAIGLLQNLVVHNLPDGRCGVAAGGRRLKALQLL

QSENRIDAGYQVMVKKVPDELAVAASMAENEQQMAMHPSEQIAGFRTLAEQGKTPAQI

GDLLGFGTRHVQRMLKLTELAPEILAALAKDEITTEHCQALALESDQKRQVEVLESAR

KRSWNNEVSVSSIRSLITSEEVSTNGDKFRFVGEAAFSPDEIRVDLFSSENGGYVKSA

SLDTALLEKLQNIAEHLREAEGWSWCDGRLDPISHYGKDTKIWRLHAVPPVEYTEAES

ERLAELEALEAKYEDENPGVNDDVLAGALEAVWEEQQTIAHRAKHRAWTDEMKQSAGV

VVSWTGQEVKVQRGVVLCADEKMEEKDASTDQAPEKVDPLDAVSVPLLTRLSSERTLA

VQAALLQQPQKAVALMVWKMCNSVFHTTTSVKEPFCISVSVSHYALTREAPDGENSVA

FQAIQSEKERLEALLPENWRKDMTTFFTLDGATLMALMAFCTACSIDGVQGKDEFGRK

HQSSLDGVENAIQFDLRDWWKPTADNLFSHMKLPHIVQALSQTGLAGAAQDAAKMKKK

DAAEHAEHFLSKIRWVPEWMTSADNQKQLAAKSELSLATSQNDTDVDAGDVTDHNNPA

CAA"

CDS complement(297485..297733)

/codon_start=1

/translation="MDHNVLSPLESLPDGTFTREQAQVVAAQYQNVAIEDDQGTHFRL

VVRHQDDGSMIWRVWNFEPGGEDMMNRYIRDYGVRKTK"

CDS complement(join(<297782..298636,298642..298782))

/codon_start=1

/translation="MLRPFRPRRFHRAGEGLPALCGATPFRARALRRCGGCLLLSPGT

RITXXSPFAGAETSTTRLAVQGTAEPFAINFSRPFRCRFIRPKKISRSPPDICTGPCS

FTPLRRTALKRVKVISRGQVQQVPVQSQNLGPGNSTGDGFKRQNNGLRRFIMAARGVN

KVILVGHLGQDPEVRYMPNGGAVANLTLATSETWRDKQTGEMRENTEWHRVVMFGKLA

EVAGEYLRKGAQVYIEGQLRTRNWQDDAGVTRYVTEVLVGQNGTMQMLGGRRESGVPE

SAAQPQNPATPAQPAQAAAKSPKAKGGKKGRQDAAPSQQPPQPLPDDFPPMDDDAPF"

CDS complement(299159..299722)

/codon_start=1

/translation="MHHDLKHRIQAMRVKLEGRAPVAEIRGSSQLFVTPSPECRRLVE

LADVRETDRILEPSAGTGAILQAIRDAVPRAKCDAVELHAGLARHLQAHFPEVRIWCG

DFLEYHPERRYTRIIMNPPFNRGDDIRHIRRALTLLEPGGILTGICLDGPRQQKALES

LADVWEPLPRGTFTYTQVATAILRITV"

CDS complement(299770..301125)

/codon_start=1

/translation="MTPTTAQIMTENTVSQTYRATYSPDDNKLRLYASLRLDEETYSL

INKAGFRWAPKQKLFVAPAWTPGREDVLLSLAGDIEDEDSTLFDRQEQRAGRFSDYSD

RRAVESEQALAHVDSLASAVPLGQPILVGHHSERRARRHAQKIESGMKRAVMLFERAE

YWEQRAQASLRHAKYKERPDVRYRRIKKIEAELRKSQKHIARSEKYMTMWRAQTLDLK

MALLVSNYDHIHACFTLDKYPRPAEKSQYEGSMSLHSALSEEIITFEQARDIAIRCHE

RTINHQQRWVNHYQNRLAYERAMLNENGGVVTRTQEFEPGGQVLSRGEWLTILRVNRS

KGEVSSVETPGYRFLGYSGTMKLTPDRITDYKAPTAEEASDAKKAAKRPPIVNYPGEG

FREMTKAEWAKLPADYKGVRGAAETETHGAYRFRRCMTHGCTLVNVYITDMKTVEIPK

K"

CDS complement(301177..301407)

/codon_start=1

/translation="MIVSIFSPSAGAVKPRRHSRILRADITAPEIDPALRAFGRHIAR

SHRKGRGVHIPAMKNTALGQVLRTLELKRAFN"

CDS complement(301499..301726)

/codon_start=1

/translation="MQDPSLRTYRIAFLGSNASGNLPMFTRVQATTGKRAIKAFIERC

EPVKGWFLGAPEDITDQVQKEEEEAGSKPQV"

CDS complement(301745..301873)

/codon_start=1

/translation="MLVYALAANGSQTGEVNTRGQVQQVPAQSQNRGPGNSTGDVL"

CDS 301929..302087

/codon_start=1

/translation="MMATVLVSAPANGLKRVIRRQGEGAGTRRNDEERGRETALPCRV

PAALSPAR"

CDS complement(302295..302423)

/codon_start=1

/translation="MASFRRHEKHILSSLHLYVSGLLPAVCLLSLLLPAVMSLFNP"

CDS complement(302453..302821)

/codon_start=1

/translation="MISRIDFLTQCVQGGXSVRMSLMFAAVTLLTGINLFFIPFQPVP

ALWGAILAVAFVLINVVADKLRLWSGIAWIIVAGIIITLMLKGKEPGDEFRVLITVFA

FLASIWYSVAQFRKENKLRS"

CDS complement(302805..303059)

/codon_start=1

/translation="MSQWNIASFSKEEQDKVAVDKVAADVAWQERMNKPVMPELVERE

QPEHLREYFHERFRVHRLNSQQLPRANAPEYNKPGDDQQN"

CDS complement(303296..303721)

/codon_start=1

/translation="MYHARQTMLNVEESDRLSFLPDLFGNDFLAGEMQVYALAEKHIT

DYCGGFWHFIRIPEGGGYMMPDGDRFHLTNPDNWFDRSVSADAAGVIITALAINRRIW

LHHERGDAALTRHLMLRESQLWNFIDSHDECAAIYAALD"

CDS 303759..303974

/codon_start=1

/translation="MCRADTTARRKAEDARATPEAAAQIKHEAGLDGFRANLSEKTTG

RTASFLKKLRPGEDGGASEPLHLQRPG"

CDS complement(304241..304471)

/codon_start=1

/translation="MNTGLRQVLRYAGVPVDAAEPVRRQFGSRLILIVTVALLPLLIV

DVPQWLFVLSQIIWGSGMYIGISVASDKPDRR"

CDS complement(304705..306216)

/codon_start=1

/translation="MMYGEEKSDSLIVAANLANNPQGAESVERRSGAKGNAEQPHMRR

TQSRESMSQRLSRVREAAKQRKKERFTALFHLLTAEALENAFLSLSRKAAAGVDGVRW

KDYAENLKVNIADLHRRLHQGSYRAQPGRRHYIPKADGKQRPLGIASLEDKIVQYALV

KILNAVYENDFMGFSYGFRPGRSQHNALDALATGLVRTNVNWVLDADISQFFDKVSHE

WLIRFIEHRIGDQRVIRLIRKWLTAGTSEEGEWRASEEGEWRASEEGTPQGAVISPLL

ANIYLHYVFDLWAHQWRRRHATGNVVMVRYADDIVIGFDKRIDAQCFRIAMQRRLKEF

GLTVHPKKTRLTEFGRFAAENRASRGKGKPETFNFLGFTHISGKDRSGRFMLIRKTRR

DRMTATLKAIKDGLRKRWHYSIPEQGKWLRRVVQGYLNYHSVPGNYPMMRKFRIYVTD

LWRRALRRRSQQDDTTWTKANRLAAVWLPKVRVLHPWPVERFTARHPRQEPGA"

CDS 306618..307043

/codon_start=1

/translation="MKFIPAVSEASIVKIPLFTERCPAGFPSPAADYTESELDLNEYC

IHRRHSTYFVRAIGNSMTDIGLYSGDLLVVDKAEQPRHGDIVIAEIEGEFTVKRLLLT

PRPALQAMNPDFPSLYPDPETLQIFGVVTAFIHKTRRAD"

CDS join(307043..308077,308079..308315)

/codon_start=1

/translation="MFALADVNSFYASCEKVFRPDLRGKPVVVLSNNDGCVIARSAEA

KLLGIKMGTPWFQLKEAQFPEKLYVFSSNYELYASLSNRVVALLEELSPRVEQYSIDE

CFLDARGIGHCMDLEDFGRQLRGHVLSGTGLTIGVGFGATKTLAKSAQWASKEWPQFR

GVLALSPDNPRRTAKLLSLQPVEEIWGVGNRIAKKLHVMGITTALQLSLTNPTFIRKN

FNVVLERTVRELNGESCISLEEAPPPKQQIVCSRSFGQRITTYEEMRQAVCQYAERAA

EKLRGERQYCRHISTFIKTSPFAVNEPYYGNVATEKLHTPTRDTRDIIAAAVRSLDRD

LARWPPLRKSGIMLNDFSPNGVAQLNLFDDVQPRPHSDALMKVLDGINHSGLGKVWFA

GRGIAPDWQMKREMLSPAYTTRWKELPVARF"

CDS 308394..308645

/codon_start=1

/translation="MTNIILCDVTASISELKNDPVATASAGGGYPVAIIDRNRPVFYC

VPAALYEQMLDELDEKDLVQMITERQNQPLREVDLNQYL"

CDS 308698..308838

/codon_start=1

/translation="MINAVLKHYKINKIELYLHVDTIQNTMVRKLGDCTDRVARRKTL

LD"

CDS complement(308890..310842)

/codon_start=1

/translation="MSKKELKPELLTVGKLFTDNYLIPIYQRNYAWRAEQIEQLISDI

QDSVVGGQDNYFLGNLVVIKRGREDEFEVIDGQQRLTTLYLLLTFLEQDGEGEKPSVG

HAGHLQYESRARATEALLRVAQEAAKEHVRPQGSTSNADAGIHEGYSIINQFFKQNEN

LNRSREKFSDFLLTKVTVVRASLPPNTDLNRYFEIMNTRGQQLKQVDIVKARLMSKLP

NQYERECFAWVWDSCADMDSYVQMSLTRGDTSLRNKVFGDEWSWLEVTSFSSLMESRP

QSGINSSRQSSEGVSLSLDEALSKYAKELESNSTEDEGNERFRSTIEFPAFLLHVLRI

MKGDEVEDEGLLDDKRLIKSFDDAVNNVPDAKADWVRSFAFMLLKCRNLFDGFILKRQ

FTANIGDDGDWSLQRLKKGGGDKKPTPTYIHVFSASNGSLEEDGSADPHTRDVLLLQS

MLRITYTSPRTMHWITKVLRWLSVKAPRDVKHADLADLLKGYARSKVKETFSFEKDQQ

PQGFGISRIVFSYLDYLLLSDSSKRDFKFQFRTSIEHFYPQHPDKEQSGAVVSGSSLN

LLGNLALVSVSANSKFSNSLPRAKAENFKDTIELQSPKLKRMAEITRNTNWDDQQITA

HHEAMVTLLRDDVSLVEASGRSNPGR"

CDS complement(310839..312119)

/codon_start=1

/translation="MAINGENGVSAVTVDELLKQGLRIPNYQRPYSWDVSTALQLVDD

ISEALRDTERKDIPYVLGAIILHDDGEYLNVVDGQQRLLTLRMILAALDPINHQISMS

GNSDTPVSLVWIELQRRLSQLEDKKEFLDFICHKCQLVRIVTDDIDEAFRVFDSQNYR

GKPLAPHDLLKAHHLREMHDESAAMKVAVVEAWEAVNDEDLDRLFSTFLYRISKWSRG

ESSLEFTIRDIGMFKGISSRSHRSFSPNLRYHLAAQAAMPLLSAWSVSSTHDARNAGR

SRFQLDAPIIAGRSFFEMVTFMLDELKILEQEVIDRGFKNFGPSQSRYRYVYELFIAA

LLCYTNKFGDEDVDEVRNRLFAWAYALRVELLRVQFVSADNRARGKNDANKSPFVLLR

NAMTGSVVRKLPITSKPYSDNHEKELVAFIKGLQ"

CDS complement(312754..312867)

/codon_start=1

/translation="MTAATRARGATYCVVEPIACKSGRWSFSAPLKSYCRD"

CDS join(313281..313500,313502..313782)

/codon_start=1

/translation="MPGNRPHYGRWPQHDFTSLKKLRPQSVTSRIQPGSDVIVCAEMD

EQWGYVGAKSRQRWLFYAYDRLRKTVCARIGERTMPTLGRFMSLLSPFDVVIWMTDGW

PLYESRLKGKLHVISKRYTQRIERHNLNLRQHLARLGRKSLSFSKSVELHDKVIGHYL

NIKHYQ"

CDS complement(314730..315386)

/codon_start=1

/translation="MLMCDATGMSQRRACRLTGLSLSTCRYEAHRPAADAHLSGRITE

LALERRRFGYRRIWQLLRREGLHVNHKRVYRLYHLSGLGVKRRRRRKVLATERLPLLR

PAAPNLTWSMDSVMDALSTGRRIKCLTCVDDFTKECLTVTVAFGISGVQVSRILDSIA

LFRGYPATIRTDQGPEFTCRALDQWAFEHGVELRLIQPGSQRRTDLLRALTDDFAMNA

"

CDS complement(315413..315676)

/codon_start=1

/translation="MKKRFSDEQIISILREAEAGVPARELCRKHAISDATFYTWRKKY

GGMEVPEVKRLKSLEEENARLKKLLAEAMLDKEALQVALGRKY"

CDS complement(316719..317690)

/codon_start=1

/translation="MKRAPVIPRHTTHTQSTEDTSSPAPAAPMVDSLIARVGAMARGN

AISLPVCGREVKFTLEVLRGDSVESASRVWSGNERDQELLTEDALDDLIPSFLLTGQQ

TPAFGRRVSDVIEIADGSRRRKAAILTESDYRVLVGELDDEQMAALSRLGNDYRPTSA

YERGLRYTSRLQNEFAGNISALADAENISRKIITRCINTAKLPKSVVALFAHPGELSA

RSGEALQKAFADKEELLKQQAETLHDQKKAGLIFEAEEVISLLTSVLKQSPSPRVNLS

SRHQFAPGATALYKGDKMVLNLDRSRIPVECIEKIEAILKELEKPGV"

CDS complement(317690..318856)

/codon_start=1

/translation="MGLMDTLNQCISAGHEMTKAIAIAQFNDDSPEARKITRRWRIGE

AADLVGVSSQAIRDAEKAGRLPHPDMETRGRVEQRVGYTIEQINHMRDVFGTRLRRAE

DAFPPVIGVAAHKGGVYKTSVSVHLAQDLALKGLRVLLVEGNDPQGTASMYHGWVPDL

HIHAEDTLLPFYLGEKDDASYAIKPTCWPGLDIIPSCLALHRIETELMGKFDEGKLPA

DPHLMLRLAIETIAHDYDVIVIDSAPNLGIGTINVVCAADVLIVPTPAELFDYTSALQ

FFDMLRDLLKNVDLKGFEPDVRILLTKYSNNNGSQSPWMEEQIRDAWGSMVLKNVVRE

TDEVGKGQIRMRTVFEQAIDQRSSTGAWRNALSIWEPVCNEIFDRLIKPRWEIR"

CDS 319193..319378

/codon_start=1

/translation="MLTASVSRKRKDDSAYRSQCFIVVPVDLNLPERIQENQARVLDI

WYGASGLVSGLFMVSIQ"

CDS 319608..320618

/codon_start=1

/translation="MTSENNSLLLNLQEVDKTTGEVVKLDVNSTSTVQPVALMRLGLF

VPTLKSTGKSKANRKNVTDATEELVQLSIAKSEGYTDVKITGSRLDMDTDFKVWLGII

RSMSEYGVKSDTLELSFVEFVKMCGFDSRRSNKKMRDRISNSLFKLASVTLKFQSETK

GWTTHLVQSAYYDINEDIVEIKAEPKLFELYHMDRRVLLRLKAIDALQRKESAQALYT

YIESLPQNPAPISMKRMRERLNLTSNVYTQNHTVRKAMEQLRDIGYLDYTEFKRGRAT

YFSVHYRNPKLISGPVKVPRNEEEEKAPEQNYDEVIKALKAAGIDPLKLAEALSAMKP

EN"

CDS complement(321023..321136)

/codon_start=1

/translation="MHPTVRYFLKAQRIIPLTATVIDHLWPTFFCHPAPVS"

CDS complement(321109..321237)

/codon_start=1

/translation="MLMTEPPWSYPVTCNPAGINIPQTRQGTMQAFWPCIPRCVIS"

CDS complement(321336..322076)

/codon_start=1

/translation="MNSIIPLQNSPERVSLLPIAPGVDFATAVALRRMATSTGATPAY

LLAPEVSALLWYMPDQRHHMLFATMWNTGIRIGEARTLTPESFDLDGLRPFVRVLSEK

VRARRGRPPKDEVRLVPLTDASFVRQMESWMVTTRPRRREPLWPVTDETMRNWLKQAV

KRAEADGVHFSIPVTPHTFRHSYIMHMLYHRQPRKVIQALAGHKDPRSMEVYTRVFAL

DMAATLAVPFTGDGRDAAEILRSLPPAG"

CDS 322109..322276

/codon_start=1

/translation="MDTGILHLTGRSCHDNKSQRADPFVVLSRIIAGKTKYVKDPFSA

QILHEASHDAG"

CDS 322606..322755

/codon_start=1

/translation="MQFQGLILLSSAESLYKRAVCWFARRKKFSVAQIQAVLNFSVLY

QQFLS"

CDS complement(322799..322915)

/codon_start=1

/translation="MIFMVSYENQWLKKRMAVLNLNKTRRHERFKKIVQEGT"

CDS 323211..324167

/codon_start=1

/translation="MKIMTNHKEENRLLSLYSMGILDTKYEERFDRLTRIATKLFDVP

IALVSLIDRGRQWFKSCYGLKIKETDRSDSFCTIAVDLSEPLIVPDASLDPRFKENKL

VKNDPYIRFYAGHPVRLPDGEIAGTICIIDTEPRVLTRDDFLLLKDLAEIVEDEFRII

NEATTDPLTGICNRRSFALMTDESLRKAKKKKKTFCVLIIDLDNFKPINDNFGHSEGN

EVLCNFADMLEDLSSSKSVVARLGGDEFGVLLPESTYKEAEEFLHKLRAGITSYNLNS

QKKYNIDFSAGIIEYDEEIHTECSAIMQDADERMYEIKKGKR"

CDS complement(324194..324319)

/codon_start=1

/translation="MENNEQFELLKNMNCDEFQGYYYSKPLSGDQLIDFLRGQKK"

CDS 324525..325493

/codon_start=1

/translation="MKDQITYLPDNADRSVAKQKFKITNWPTYNKALINRGSITFWLD

DEAIQAWYESATPSSRGRPQRYSDLAITTVLVIKRVFRLTLRAAQGFIDSIFSLMNVP

LRCPDYSCVSRQAKSVNVSFKTPTRGEIAHLVIDSTGLKVFGEGEWKVKKHGQERRRI

WRKLHLAVDSKTHEIICADLSLNNVTDSEAFPGLIRQTHRKIRAASADGAYDTRLCHD

ELRRKKISALIPPRKGAGYWPGEYADRNRAVANQQMTGSNARWKWTTDYNRRSIAETA

MYRVKQLFGGSLTLRDYDGQVAEAMALVRALNKMTKAGMPESVRIA"

CDS complement(326166..326423)

/codon_start=1

/translation="MDKKYDFSLSYEALTRVCENAICEHIRRAGSLEGLGFALEYTKA

YAILEVWSLLAAAGDTFPALIEKDRIYLLQLISGKNNIELH"

CDS 327064..328479

/codon_start=1

/translation="MLVRSESLLVQTESVKTAAKSHLNIGDSPCTNENILHLRVVVWP

YPLIKDVGYIIKGELACSALWGSLRPTLSLEHFDKKWTSREGVWLFGIKLMDDISVNG

YISEGIMVTLSPFVFRRFETDMYSKNFSAVVGNSKHDRHYFNIGPDAPLLDRHDSVPL

RFRVFRACSLHYDLCVAGGGYFTGIFSEHWSIQMLLVTVSLLSGLMIYIIIMNRAELN

SSLSARFVKALKNEALSLVYQPIYRIEDGQICGFEALLRWKDERLGNISPEVFIPLSE

REGLQEDVTLFVINHAIREFIHTAIQNEIFLSVNINPSDLDSEKFRDKLLGLISEYNI

PYKTILLEITERQGGDFEGMKIHIDKYKNHGVRFAIDDFGTGYSNLNLVTALDVDEIK

IDKSLTSAIGTESLRYDLLPGLHEMFRSIADKIVFEGVETQEQVNYLKTFWPQSYAQG

WYYSRALPLEEARKLTIRELN"

CDS complement(328603..328749)

/codon_start=1

/translation="MLQTNNAVYRILAAPTPLYYETKLAHLSKYYIRCSVFCIKSHPG

FSVI"

CDS complement(329462..330739)

/codon_start=1

/translation="MISRWKWILKQTVKKLWFRATLFAIVAIITALLSILFKSMIPES

VSVKVGAEAVDNILNILASSMLAVTTFSLSIMVTAYGSATTNVTPRATRLVVEDVTTQ

NVLATFIGSFLFSLVGIIALSMGAYGERGRVILFIVTLVVIALILITLLRWIQHLTSL

GRVGETTAKVEQAAIETFIARARNPCLGGYPWLENNEQPKGTVAVYPKKIGYVEYINM

VKLSKLLTNDPRHVYLVAQPGSFIHPSMPVLYLSQGQESSISADLLETIIVSDVRSFA

QDPRFCLSVMAEIACRALSPAVNDPGTAIDVIGRGVRILSTYAQNKSDEIEVKYPSVH

VAPLQNNDLLEDFFSPVARDGAGMREIQIRVLKGLSMLSKGWPGIFSEAAHNLAFETL

EHAIRADHIDSDRCLIKSIYYNLFSGEDSNKKP"

CDS complement(330802..332805)

/codon_start=1

/translation="MIMSDNDTIPKKSTSQINKAVFFTSALLIFLLVAFAAVFPDVAD

KNFKLLQQQIFTNASWFYILAVALILLSVTFLGLSRYGDIKLGPDHAQPDFSYHSWFA

MLFSAGMGIGLMFFGVAEPVMHYLSPPVGTPETVAAAKEAMRLTFFHWGLHAWAIYAI

VALILAFFSYRHGLPLTLRSALYPIIGDRIYGPVGHAVDIFAVIGTVFGVATSLGYGV

LQVNAGLNHLFGVPINETVQVILIVVITGLATISVVSGLDKGIRILSELNLGLALLLL

ALVLCLGPTVLLLKSFVENTGGYLSELVSKTFNLYAYEPKSSKWLGGWTLLYWGWWLS

WSPFVGMFIARVSRGRTIREFVTGVLFVPAGFTLMWMTVFGNSAIYLIMNQGATDLAN

TVQQDVALALFNFLEHFPFSSVLSFIAMAMVIVFFVTSADSGAMVVDTLATGGVANTP

VWQRIFWASLMGIVAIALLLAGGLSALQTVTIASALPFSVILLISIYGLLKALRRDLT

KRESLSMATIAPTAARNPIPWQRRLRNIAYLPKRSLVKRFMDDVIQPAMTLVQEELNK

QGTISHISDAVEDRIRLEVDLGNELNFIYEVRLRGYSSPTFALAAMDNNEQQTEQHRY

YRAEVYLKEGGQNYDVMGWNQEQLINDILDQYEKHLHFLHLVR"

CDS complement(332951..333454)

/codon_start=1

/translation="MSRQCTHYGRWPQHGFTSLKKLRPQSVTSRIQPGSDVIVCAEMD

EQWGYVGAKSRQRWLFYAYDRIRRTVVAHVFGERTLATLERLLSLLSAFEVVVWMTDG

WPLYESRLKGKLHVISKRYTQRIERHNLNLRQHLARLGRKSLSFSKSVELYDKVIGHY

LNIKHYQ"

CDS complement(332951..333454)

/codon_start=1

/translation="MSRQCTHYGRWPQHGFTSLKKLRPQSVTSRIQPGSDVIVCAEMD

EQWGYVGAKSRQRWLFYAYDRIRRTVVAHVFGERTLATLERLLSLLSAFEVVVWMTDG

WPLYESRLKGKLHVISKRYTQRIERHNLNLRQHLARLGRKSLSFSKSVELYDKVIGHY

LNIKHYQ"

CDS complement(333839..334720)

/codon_start=1

/translation="MDSARALVAKGRGIALVSRTMGVSRAQLSLRINRSADWQDKRCN

RRNDEADEEILSAILDIISDMPSYGYRRVWGILRKQRRTEGQPPVNAKRLYRIMSEHN

LLLLHDKPERPKREHKGKIAVAESDMRWCSDGFEFGCDNGEKLRVTFALDCCDREAID

WAASTGGYDSSTVQDVMLRSVEKRFGDRLPDTAVQWLTDNGSAYTAYETWRFARELNL

EPCTTAVSSPQSNGMAERFVKTMKEDYIAFMPKPDVRTALRNLAVAFTHYNENHPHSA

LGYHSPREYRRQRTSLT"

CDS complement(333839..334720)

/codon_start=1

/translation="MDSARALVAKGRGIALVSRTMGVSRAQLSLRINRSADWQDKRCN

RRNDEADEEILSAILDIISDMPSYGYRRVWGILRKQRRTEGQPPVNAKRLYRIMSEHN

LLLLHDKPERPKREHKGKIAVAESDMRWCSDGFEFGCDNGEKLRVTFALDCCDREAID

WAASTGGYDSSTVQDVMLRSVEKRFGDRLPDTAVQWLTDNGSAYTAYETWRFARELNL

EPCTTAVSSPQSNGMAERFVKTMKEDYIAFMPKPDVRTALRNLAVAFTHYNENHPHSA

LGYHSPREYRRQRTSLT"

CDS complement(334678..335073)

/codon_start=1

/translation="MSNTNANFEMTGILLGQEARKRKTPQEKIAIIQQTMEPGMNVSH

VARLHGIQPSLLFKWKKQYQEGSLTAVAAGEEVVPASELTAALKQVRELQRLLGKKTM

EVEILKEAVEYGQSRKWIAHAPLLPKDGE"

CDS complement(334678..335073)

/codon_start=1

/translation="MSNTNANFEMTGILLGQEARKRKTPQEKIAIIQQTMEPGMNVSH

VARLHGIQPSLLFKWKKQYQEGSLTAVAAGEEVVPASELTAALKQVRELQRLLGKKTM

EVEILKEAVEYGQSRKWIAHAPLLPKDGE"

BASE COUNT 81895 a 85769 c 87979 g 79674 t

ORIGIN

1 cagggcggtg gtagcccacg ttttcggtga acgcactatg gccacactgg agcgtcttct

61 gagcctgctg tcagtttttg acgtggtggt atggatgacg gacggctggc cgatgtatga

121 gtcacgtctg aagggaaaac tgcacgtcat cagtaagcgt tacacgcagc gaattgagcg

181 acacaacctg aatctgagac agcatctggc aaggctgggc aggaagtccc tgtcgttctc

241 aaaatcggtg gagctgcatg acaaagtcat cgggcactat ctgaacataa aacactatca

301 gtaaattgga gtcattacca gattggctta caccattaga gaaatttgct cagcttgttg

361 attatcatat ggcttttgaa actgtcgcac ctcatgtttg aattcgcccc atatttttgc

421 tacagtgaac caaattaaga tcatctattt actaggcctc gcatttgcgg ggtttttaat

481 gctgaataaa aggaaaactt gatggaattg cccaatatta tgcacccggt cgcgaagctg

541 agcaccgcat tagccgctgc attgatgctg agcgctgatg aatcccctaa tgatttttat

601 caaaatcatt aagttaaggt agatacacat cttgtcatat gatcaaatgg tttcgccaaa

661 aatcaataat cagacaacaa aatgtgcgaa ctcgatattt tacacgactc tctttaccaa

721 ttctgccccg aattacactt aaaacgactc aacagcttaa cgttggcttg ccacgcctta

781 cttgactgta aaactctcac tcttaccgaa cttggccgta acctgccaac caaagcgaga

841 acaaaacata acatcaaacg aatcgaccga ttgttaggta atcgtcacct ccacaaagag

901 cgactcgctg tataccgttg gcatgctagc tttatctgtt cgggcaatac gatgcccatt

961 gtacttgttg actggtctga tatccgtgag caaaaacggc ttatggtatt gcgagcttca

1021 gtcgcactac acggtcgttc tgttactctt tatgagaaag cgttcccgct ttcagagcaa

1081 tgttcaaaga aagctcatga ccaatttcta gccgaccttg cgagcattct accgagtaac

1141 accacaccgc tcattgtcag tgatgctggc tttaaagtgc catggtataa atccgttgag

1201 aagctgggtt ggtactggtt aagtcgagta agaggaaaag tacaatatgc agacctagga

1261 gcggaaaact ggaaacctat cagcaactta catgatatgt catctagtca ctcaaagact

1321 ttaggctata agaggctgac taaaagcaat ccaatctcat gccaaattct attgtataaa

1381 tctcgctcta aaggccgaaa aaatcagcgc tcgacacgga ctcattgtca ccacccgtca

1441 cctaaaatct actcagcgtc ggcaaaggag ccatggattc tagcaactaa cttacctgtt

1501 gaaattcgaa cacccaaaca acttgttaat atctattcga agcgaatgca gattgaagaa

1561 accttccgag acttgaaaag tcctgcctac ggactaggcc tacgccatag ccgaacgagc

1621 agctcagagc gttttgatat catgctgcta atcgccctga tgcttcaact aacatgttgg

1681 cttgcgggcg ttcatgctca gaaacaaggt tgggacaagc acttccaggc taacacagtc

1741 agaaatcgaa acgtactctc aacagttcgc ttaggcatgg aagttttgcg gcattctggc

1801 tacacaataa caagggaaga cttactcgtg gctgcaaccc tactagctca aaatttattc

1861 acacatggtt acgctttggg gaaattatga ggggatctct cagtgctgag cgggtgcatg

1921 cccggtgaaa tccgcccgac gattggccag caaatggaaa ctggcgacca acggtttggc

1981 gatctggttt tccgccagct cgcaccgaat gtctggcagc acacttccta tctcgacatg

2041 ccgggtttcg gggcagtcgc ttccaacggt ttgatcgtca gggatggcgg ccgcgtgctg

2101 gtggtcgata ccgcctggac cgatgaccag accgcccaga tcctcaactg gatcaagcag

2161 gagatcaacc tgccggtcgc gctggcggtg gtgactcacg cgcatcagga caagatgggc

2221 ggtatggacg cgctgcatgc ggcggggatt gcgacttatg ccaatgcgtt gtcgaaccag

2281 cttgccccgc aagaggggat ggttgcggcg caacacagcc tgactttcgc cgccaatggc

2341 tgggtcgaac cagcaaccgc gcccaacttt ggcccgctca aggtatttta ccccggcccc

2401 ggccacacca gtgacaatat caccgttggg atcgacggca ccgacatcgc ttttggtggc

2461 tgcctgatca aggacagcaa ggccaagtcg ctcggcaatc tcggtgatgc cgacactgag

2521 cactacgccg cgtcagcgcg cgcgtttggt gcggcgttcc ccaaggccag catgatcgtg

2581 atgagccatt ccgcccccga tagccgcgcc gcaatcactc atacggcccg catggccgac

2641 aagctgcgct gagccatggc tgaccacgtc acccccaatc tgccatcgcg cgatttcgat

2701 gtgacagagg cgttttatgc gaagctgggc tttgcgacga gttggaagga tcgcggctgg

2761 atgatcctgc agcgcggcgg tttgcagctc gaattcttcc cctatcctga cctcgaccca

2821 gctacgagct cgttcggctg ttgcctgcgg ttggatgatc tcgatgccat ggtggcattg

2881 gtgaacgcgg cgggagccga ggaaaaaagc accggctggc cgcgcttcaa agctccgcaa

2941 ctggaggcga gcggcctgag gatcggctac ctgatcgatc ccgactgcac gctggtgcgg

3001 ctgatccaga accccgactg accgcatgcc cgcgaaaatc aagatttgcg ggatcagcac

3061 acccgaggcg ctcgatgcga ccatcgcggc gcgggcggac tatgccgggt tggtgttcta

3121 tccagcgtcg ccccgtgcgg ttacgtcgaa tgtcgcgggc gctttgacat cgcgcgcagc

3181 tggccagatc gccatggtcg gtttgttcgt cgatgcggat gatgctgtca tcgccgacgc

3241 actggtggca gccaagctga acgcgctgca gctgcacggt tcggaatcgc ccgaacgcgt

3301 ggcccagttg cgcgcgcggt ttggcaagcc ggtgtggaag gcgctgcccg tcgccagcgc

3361 cagcgatgtc gcacgcgccg cagcctatgc cggggcggcg gacttgatct tgttcgacgc

3421 caagaccccc aaaggcgcgc tgcccggcgg catggggttg gcgttcgact ggtcgctgct

3481 ggccggatat cgcggtgcct tgccgtgggg gctggctggc gggctaaatc cgacgaatgt

3541 tgccgaggcg attgcgcgca ccggagcgcc gctggtcgat acctccagcg gcgtcgaaag

3601 cgcgccgggc gtcaaggata ccgacaagat taccaatttc gcctttgcgg tgcgcttggc

3661 ctaaatcgcg tcgatcaata ggcgtcgttc agcgcaaaga tcggcttgcg ggtgcgccac

3721 tgccctcggg tgaagtcggg aaaatctaac gtgcgattgc cctcagcaat cgattgttcc

3781 gacagaggcg tgatcgcgct ccaggccagc gcgtcgtaaa tgtcgattgg catcggggcc

3841 ttggccttca gcgcctcgac aaaagcgtgg atcacgaacc agtccatccc gccatgcccg

3901 gcccctgccg ccagatcggc gtagcgtttc catagcgggt gatcgtattt cgcaaaccag

3961 ccctcggcag gctcccagcg gtgcggctgt gggctcttgc cctccagata gatcgacttg

4021 ttgacgtcca tccacagccc ctcggtgcct tgcacccgaa agccgagaga ataggggcgc

4081 ggcagcgagg tgtcgtggca cagcatgatc gtttcaccat tagtgcagcc gatcatggtg

4141 ttgaccacat cacccagtgc gaatttcacc tcggcgttgg gatgatcggc agagccgttc

4201 ttgacgacat aatcatgcag cccgcgcgcc ttacagccga agccgccagc gcccgcttcg

4261 cccggcaacg cgaccttcag ggtgcgggtc tgcggcgggt agcacacgcc ggcatcggcg

4321 cagccctggt acttcacggt cagggtggtc gcgctcgcgc cggccgcggg cgtgccggtg

4381 agggtgccga gcaattcctt gcggtaggtt tcgacgtcgc cgaagaattc gtcgcggtag

4441 gccttgccct tcggcagcgc catggtcgcg ccggtgaagg cggcatcggc cttgaccgag

4501 gtgcggtgcc ggtacaggta atagccgtcg gcgatccgcc agcgcacctc gatgcggtcc

4561 ggcgcggtgg cctgcgcgga caggacgaag acctcgtcga ccggcggcag ttcgaagtcc

4621 tgggcgacgg ccgaggtcgc gggcagcgca agcagcaggg cgagcccggc cagccagcgg

4681 cgcaggcgga tcgtggatgc ggtcattggc tcagtttacc ggtcggctct cggcggccag

4741 ccattgcagg tattcgggca ggccggacgc ggcttcgacc gcgagcagct ccgggagttc

4801 gtagggatgc agttggcgca ggcgttcctg cagggcgggg taggcctcgg cactggtctt

4861 gaccagcagc aggacctcgg ccgcggcctc gaccttgcgt tgccagcgat agaccgaacg

4921 caggccgggc aggaggttga cgcaggcggc caggcgctcg gccaccagcg cggtggcgat

4981 gcgctcggcg ctgtcggcgt cgggacaggt gcagaagcag atcagggcgc tcaccggcat

5041 agggtagcgg ctgccccgat ccggcgggcc tggcggacat ccgcgtgcgg cccttgaaag

5101 tcggcgggcc cgccccatct cggtggcatg ccgggttcgc ccggttctgt tgtccgcggt

5161 ttggcactcg cttcgcgcga ctgctaaaat cgccgggttt ttccacgtca atcaaccatt

5221 taccgaggtt gccatgtcca atatcaagcc gctgcacgac cgcgtggtca tcaagcgcat

5281 ggaagaagag aagctgtccg ccggcgggat cgtgatcccg gattcggcca ccgagaagcc

5341 gatcaagggc gaagtcgtcg ccgtcggcac cggcaaggtg ctggacaacg gccaggtccg

5401 cgcgccgcag gtcaaggtcg gcgacaaggt gctgttcggc aagtacagcg gcaccgaagt

5461 gaagctggac ggcgtcgagc tgctggtggt gaaggaagac gacctgttcg cgatcctcgg

5521 ctgatcgcgc gtcgctccca cacatttctc atccgaataa tttttcgagg taattcgcaa

5581 tggctgccaa ggacattcgt ttcggcgaag acgcgcgctc caagatggtg cgcggcgtca

5641 acgtgctcgc caacgccgtg aaggcgaccc tcggcccgaa gggccgcaac gtcgtgctgc

5701 agaagagcta cggcgcgccg accatcacca aggacggcgt ctccgtcgcc aaggaaatcg

5761 aactggctga cgcgttcgag aacatgggcg cgcagatggt gaaggaagtc gcttccaaga

5821 cctccgacaa cgccggcgac ggcaccacca ccgccaccgt gctggcgcag gcgttcatcc

5881 gcgagggcat gaaggcggtc gccgccggca tgaacccgat ggacctgaag cgcggcatcg

5941 accaggcggt gaaggccgcg gtcggcgaac tgaagtcgct gtccaagccg tcgtcgacca

6001 gcaaggaaat cgcccaggtc ggcgcgatct ccgcgaactc ggatgccaac atcggcgacc

6061 tgatcgcgca ggcgatggac aaggtcggca aggaaggcgt gatcacggtc gaggaaggca

6121 gcggcctgga caacgaactc gacgtggtcg agggcatgca gttcgaccgc ggctacctga

6181 gcccgtactt cgtcaacaac cagcagtcga tgtcggccga cctggatgat cccttcatcc

6241 tgctgtacga caagaagatc tccaacgtgc gcgacctgct gcccgtcctc gagggcgtgg

6301 ccaaggccgg caagccgctg ctgatcgtgg cggaggaagt cgaaggcgaa gcgctggcga

6361 ccctggtggt caacaccatc cgcggcatcg tcaaggtctg cgcggtgaag gccccgggct

6421 tcggcgaccg tcgcaaggcg atgctggaag acatggcgat cctgaccggc ggcgtggtga

6481 tttccgagga agtcggcctg tcgctggaga aggccaccat caaggacctc ggccgcgcca

6541 agaagatcca ggtgtcgaag gaaaacacca ccatcatcga tggcgccggc gaaggcgcgg

6601 gcatcgaggc gcgcatcaag cagatcaagg cgcagatcga ggagacctcc tccgactacg

6661 accgcgagaa gctgcaggag cgcgtggcca agctggccgg cggcgttgcg gtgatcaagg

6721 tcggtgccgc caccgaagtc gagatgaagg aaaagaaggc gcgcgtcgaa gacgccctgc

6781 acgcgacccg tgcggccgtc gaggaaggca tcgtcccggg cggcggcgtc gccctgatcc

6841 gtgccaaggc ggcgatcgcc ggcatcaagg gcgtgaacga agaccagaac cacggcatcc

6901 agatcgccct gcgcgcgatg gaagccccgc tgcgcgagat cgtgaccaat gccggcgatg

6961 agccgtcggt catcctcaac cgcgtggtcg aaggttcggg tgcgttcggc tacaacgccg

7021 ccaacggcga gttcggcgac atgatcgagt tcggcatcct ggacccgacc aaggtcaccc

7081 gcaccgcgct gcagaacgcc gcgtcgatcg cgggcctgat gatcaccacc gaagcgatgg

7141 tggccgaggc cccgaagaag gacgagccgg cgatgccggc cggcggcggc atgggcggca

7201 tgggcggcat ggatttctaa gccccgcgat ccatcaagca agaccacaaa gcccggcctc

7261 gtgccgggct ttgtgcgttc tggcgtccga ggcgggagac ttcctacccg ccccgcggca

7321 atgtctgacg cgaagatcag aaaacgccga tatgaacgcg tgctcgcggg cgcaaccctg

7381 agcagccgtc cctgcaacgg agcgctgcgt gccgcgcctg accgcacccc ggcggcaggc

7441 cgaggtgtgc gcgccactgc cggccgccca cgccgctgcg cgttacgcgc gccacctgcc

7501 cgagcgcacg ctgctgtacg cgctagtgca ggcgcactac ccggacttca tcgcgcgtct

7561 tgaggccgaa gaccgcccgc tgcccgagta tgtgcgcgag gagttcgaga cctacctgcg

7621 ctgcggcgtg ctcgagcacg gcttcctgcg cgtggtctgc gagcactgtc gtgccgagag

7681 gctggtggcg tattcctgca agaagcgcgg gctgtgcccg agctgcggcg cacggcgcat

7741 ggccgagtcg gcgcggcatc tggtggacga ggtgttcggc ccgcggccgg tgcggcaatg

7801 ggtgctgagt ttcccgtacc cgttgcgctt cctgttcgcc agcaagcctg aggcgatcgg

7861 cccggtgctg ggcatcgtgc atcgtgtgat cgccggttgg cttgccgatc aggccggcgt

7921 gccgcgggat acggcgcaat gcggcgtggt gaccctgatc cagcgcttcg gcagcgcgct

7981 gaatctcaag tgtaacccaa accctcacct atgacgccag aggaagactt gtcagctcca

8041 ctgggccaga aggaaccacg gtctacaact atgatgctgt gggcctcctg agttcgctga

8101 ccaagccaaa tggtgcaacc gtcagctatg agtacgacgc tgcacatcgg ctggtggcgg

8161 aaacagatgc acagggcaac cggcgcgagc ttgagctcaa tgacctcggg aacccagtag

8221 aagagcgact gctcgatgcg ctgggccaga cccgttggat agagcgccgg atcttcaacg

8281 aaatcggctg gctctccagt gtctccgacg cctatagcaa tcagtcatcg ttttcctacg

8341 atgtggtggc aaacctgata caggagacca gtccctctgg taacacacac tcctacaagt

8401 acgacggctt ccatcaccgg acacaaacga ccgatcccct cgggaaggtc acgcaggtgc

8461 tctacaagga taccggcgat gtttaccgtg tctccgaccc tcgttcgcgc ctgacctact

8521 acagctacaa cggctttggc gaagtgaccc aggtccggag cccggacacc ggcaccaccg

8581 acattaccta tgacgaagcc ggtaacgtgg caacgcgcaa aacggccaag gggcaaacca

8641 caagctacag ctatgacgcg ctgaaccgga tcatcgaaac ctccagcgat gtcgctggcg

8701 aatcgccaat tctgtacggg tacgacgaag caacctcacc atacggcata ggccgcttga

8761 cctcagtcga tgatggcaac ggtgtccgga gatttggcta cacccccgaa ggatggctgg

8821 cttacgaaac ctgggaaacc cacgggcaga gcctgactac ccagtaccaa tacgatggtg

8881 caggcctcgt cacgaagatc acgtatccca gtggccgtga ggtctcctac acccgtgact

8941 cagccggtga cgtcatcgag gtgacaacga cacaagcagg caccacaaca aacctggcaa

9001 gccagattga gcgagcgccc tttggccccg tcaccagtat ggtcagaggg aacggcattt

9061 cagaaagccg cactctggat ctcgattacc gtgtcaccgg catcgacgct gctagggtgc

9121 attcgctggt ctatcggtac acgccagact cgttgatttc agccatagac gacaatctca

9181 gctcatcagt caatcagtca ctcggttatg acgcggttgg ccgcatcacc tctgctgagg

9241 ggatctatgg cgttttgggc tatggctatg acgccaccgg caacaggacc tcgatcacga

9301 ccgatggcct gagccaaagc tacaccatca actacatgaa caactggttg gtgaaggccg

9361 ggcaaacctc cagaagctat gacgccaatg gcaacctgac gaagcagggg gcggatacct

9421 tcacctatga cagccagaac cggctggtgg ccgcaacggt cgcgggagtg actgtaagct

9481 acacatacaa ccatctggat cagcgtgtaa ccaagaccct aaacgggcat acccggctgc

9541 tggtttacga cctggcagga aacctcatcg aggagctgga cgcggccact ggagacgtgc

9601 tggcggagta catctggctc gatgggacac ccttgggctt tgttcagtca ggacagacct

9661 accaagtcca cgtcgatcac ctgggcaccc cgaaggcact gaccgacgtc agcggccaag

9721 tcgtttggaa ggcgagctac agcccgttcg gtaaggccag catcatcatc caggggccaa

9781 ccttcaacct gcgattccca ggacagtatt acgacgcgga gaccgggttc cactacaact

9841 ggcggcgtta ctacgaccca gcgaccgggc ggtacattac cagcgaccct cttggcctga

9901 tcgatggagt aaacacctac gggtatgtgc atggaaaccc tatgtccaat accgacccga

9961 cgggtgaatt tgcgtttgtt ggtgcaggta ttggagctgg gttggagcta cttagccaac

10021 taatcgaaaa taatgggagt tggaaatgtg ttagttggtc aaaagttgga atcgccggag

10081 cgattggggc tataggtggc ggctgggcgt caggagtttt cagacatgcc agctccggta

10141 aatcgtggtt caaattaagc caaaaatgga gcaatgtctc acccagagta aggaaagttc

10201 aaggggttcc acgaggtaat gagcttcacc attgggctat tcagagaaat ggcaagtttg

10261 gcaaatatgt tcctgactca ataaaaaacc atccttggaa cttgaagtcc attccaagag

10321 atattcacca aaacattcac ggcaatggac ctaccccata tagcgcattt ggtcgttggt

10381 ggcacgggac gcctgaatgg gcaaaagtag ctcaagcctc tcctgttagt ggtggtttag

10441 ctgattcaat aaatgatgag ggatgcggtt gtgcaaattg aatttccagc gctgcttgtg

10501 agcagcaaga aaaggtcgct cttcgtagtg gcatcagaat ccgagttcgg gaaatgtact

10561 attcagtctt tgaggaacgg ttattttgag ctgatggaca tttatgattc ggaaggtcgt

10621 cactacaaaa tagacgaggt tgcgagctac aagccgctaa gtccattctg gtactggcct

10681 gtagaaattg tgatgtatgg ttctcgactg tttaaggcta atttcaatgc tgttcttatc

10741 tctaatctgg attgcaagga gttaaaatct gaactttgtg atttggctaa aaagtacaga

10801 agtaatttgg attccggcgt cggaattgaa aagattatgg aggaaatgga gtctgccaga

10861 acaattaaag aattgataaa ggtttttggc tagttattca ttgaatacgc tggtgttagt

10921 ctagctaacc gacgccagcg gccaagtgtt tggaaggcga gccatcatag agtcagtaga

10981 gagagatact tttggaaatc aatagtatta tgattggtac aaatgagttg tagctccata

11041 ttttaaaggg ggatttcaga gaaatgaaga ataagaaggt aataactttg ataatggccg

11101 cagccgcatc atgctcgtca gtctacgccg cgacattacc gaccagtgag gtagacgcat

11161 acatacttgc gatgaacacc atgtcaccta tcactgcaaa gtacaccatc cagtacaagc

11221 aagcggttga gcagaaatgc aatacagccc tgagcgttga gcagctcaac tcaaaggcat

11281 tcaccaatgt tgtccaggca atggtgagta gtgagaccgt cgatagaatg gggcttgatg

11341 ctgctggtgg atcactccag gataccctgt ctgtgatcgg caagaacgtc acatgcagtg

11401 acctgaacgc gccatttaaa gcactcttgg atgataagga cttcaccaga aagcatcagc

11461 acctatcgaa ggtgctgcat acttggaatg aagttgtttc tcaaagcaaa ccctgagtaa

11521 caaaaaggag cctaactggc tcctttttta atggggatga tgttacgttc gtggacgtta

11581 ttttcactcg ggatatgtaa cgttgaaaaa ggcgtaccga caagagagtg gtcacatagc

11641 tctccataat agatgagcga caaaatctct tgctcgttga tttttcccac cttaaaaagg

11701 taattgatta ttgctgtatc cagtaggtcg tgacacccat taagcagatc cacgatattg

11761 gccgcagctt cattttcctc tccgtcatct cgaccggcta atactgattc aacccaagcc

11821 tcaaggctcg gatgtccctc agcgagctct atttgacggc actcgttctc gatcaaccct

11881 gtgattgctt cgttgagcgt tccgccataa gtcggcatgt tgagaacaac tccaaatccg

11941 gggatttctt tttggattgg cttcgcatcc ttgggagctg atttcagcca caggaaaaac

12001 cagatcccag agcttatgtc ttccggcttg acctggttgg ccgcaaggaa tttggaaacc

12061 tcgttacaga tgatgccatc tggcgctgcc atctcgtaaa tacccgccat cagatcttca

12121 aggctcttat acatattatc tctcgcacct gtacatgatg ttttcactat cgttgttaag

12181 cgttttttca gcaagtatgt tccagcgaga catcttatac cattccatct aaatctgtaa

12241 aattaccgta ttaccgtatt accgtaatta tgttaattac ggtaaataaa tttaagttct

12301 tcttgctgtt gttctactta ttaaaatgta cctttgaaaa agtagagttt ttggaatagg

12361 ataggtggtg ctgaatgccc actttttaca aggagcgcga ccagtgattt ttgatgtaag

12421 agcgaccttt gaagtggcac tccagacgga cacccacctg gtacttatcg atctggatca

12481 gggagcatca gttacgaacg atgctgatgc agtgatcgcc tggttggccg caaacctcga

12541 aggtggaatt ggaaaacgta aggtgtacta ccgggacacc gatggacgct ttgatgagct

12601 caaggttaat gccggtgctt ttgccggatt tgcaccttgt agcgaaggcc agcagaccac

12661 cttggctggg atgctcggcc agtaactttg tgtggagaac tcgatgttaa acaaaaacaa

12721 atttgagaaa gttttaaaga gaatcctcga taagaacttt gagcgctgct ctatttgcag

12781 gaaaccattc cctggaccgt gccatacttt tgctggtttg gattcggaca ataaagttca

12841 gaacgtaggt tcttgctgtc ggaccagtat tgtggacttg cggcatggag gtgtgtatac

12901 gaccgctcct gtagatactc aagaggggca aagtcaggcg catgagctac tcgcaacaca

12961 tccttgtaag gggatgatgg ggcatgctta gcaaaaaagt ctttttcatt agtcaagcgg

13021 aggccgaaag actggagccg gttcccggtg cggccatgat ctccataact gacccagaca

13081 agtcccctgc cgctctcgga cagtgggggc agttgtaccg cgatagcttc tatgacggcg

13141 gctattctga gaacaccatc cacacgatga aggcagcgtt ccggatgaat tacgcctctt

13201 acattgactc atcccaggct gaaaagctgt ccaccttcct ggatggattg gttggtagcg

13261 gtatcgatca gatcttcgtt cattgctatt acggagaatc cagaagtggc gcggtagcgc

13321 tgtacctcca gaacaaacat ggattcactc cgaataagcc gatcaccaag cccaaccgaa

13381 cagtttacga attgctgtgc aatccaacca agtttgaacc actgatgcaa agctacgaaa

13441 cgcaacatat ggaggaggaa ctacctcttc acctcaaaat atgggatttt cttctcgttg

13501 cggtcgggct taggaggtga ctttgcacga cacaaacaat gacaaggaag agctagtttc

13561 acatgcgaaa gtaaatgttc cggcagaaca aagcattcgc ggtgaactgt tgccgtcatc

13621 gagctcgctc cggaacatcc aggacaaccc cgccgtcgcc tatctggtga gcctcgggtc

13681 aaagcgaagc aggcagacca tgagctcatt cctcaacatt gtcgccaaga tgatcgggtt

13741 tcagaacctt cgtgactgtg catggagctc aatgaggcgg caccacatat tggcggtgct

13801 ggaaatgctg ggggatgcag ggaaggctcc ggcaacgatc aacacctacc tgtcagcgct

13861 caaaggggtg gctcttgaag cctggacgat gaagcaaatt gatacggata gcttccagca

13921 cattaagcaa gtccgttcag tacgtggatc tcgacttcct aaagggcggg cacttgaacg

13981 ccatgagatt cgcagcctct ttttcacatg tgaaagtgat tcaagcgcca aagggcttcg

14041 ggatgcggcc attctcgggg tgctcctcgg gtgtggcttg cgccgctcgg aaatcgttgc

14101 gctggacatg ggaagcatga tctacaagga ccgcgctctc aaggttcttg gcaagggcaa

14161 caaagagaga atggcgtatg tgcctggtgg cgcatggaaa agactggata agtgggttga

14221 agaggttcga ggaacgcatg aagggccttt attcccgagg atcaggcggt ttgatgatgt

14281 gactggagag cggatgtcgg atcaggccat ctaccacatc ttggagacca gaagagttga

14341 agccggtctg gagatgtttg cgccccatga cctgcgacgc acctttgcct cctcgatgct

14401 ggataacggt gaggatattg tgaccgtgaa ggacgcaatg ggccactcaa gcattgcaac

14461 tacccagaaa tatgacagac gcggcgatga gcgtttgaag agagcgagcc aacgcctcga

14521 tatagcggat taacagcatg aatgatgaag aactcgaact tgccagagcc gaagccatga

14581 aagccgatag gtgcttttca aaaggacggc tcagggacga atttcggatg aagcctaagc

14641 caggagtaga gccagtttcg ttctacaaaa acgggtatgg tggccaattt ggagtgtacc

14701 ggatagcaga ttgccaacca atgcggagaa ggggttgttc accggcatca caaaagcaaa

14761 taagggcgca atccatcctt tcggtcaagg caagaatgcg tagcaacctg gcaaaggcct

14821 ctgtcatggc acaaaggtgg gtcgctctgg aacctctggt tctcgatacc gagacgaccg

14881 gacttggtga aagagaccag gtgatcgagc tggctgttac tgacatcaga ggcgcggttc

14941 tcctctgcac cagattacgg ccaaccgtgg aaatagaccc tcaggcaatg ggtgttcatg

15001 gcatcaccga gactgagctg tcgaatgagc ctacgtggac tcaagttgcg ccagctctcg

15061 cccggctttt gtcgggccgt catctggtga tatttaactc cagtttcgac agtaggatgt

15121 tgaggcaaac agccagtgca tttggagacc aactctcttg gtggcaagag cagaactgtc

15181 tgtgcgcgat gaagctggcg gctgatgcct ttggctcaac caaccggcac ggcacaatct

15241 cacttgcgga tgccacctgc gaggcaggtg tgagctggaa aggtcgagcc cattcagcag

15301 cgaccgacgc tattgccacc gccgacttgg tgacagagat agccaaagtc caacgtgacc

15361 tcatggtcca gctccaggag cttcaaagca aaggtaattt ggaatgacag aacaatccta

15421 cggcgaaagc ctcaaatttt tctcagactg gcagaaggac cccgcgaaac gcaccggcct

15481 gaatgtgcaa cacacgctaa ccagaggtga atatcctacg gtcagcatcg agattgcgcc

15541 gatcagggca tcggggtcca gcccagactg gaaaagcaag atcacggtgc agctcactcg

15601 cggcgaactg acggcgtttt gcagcgttct ctttggtttg cgtagcaagg ctgagggttc

15661 ctatcacggc gacgcgaaga ataagagctt tgcggtttac aacaacggca aggccggtgt

15721 tgcgatcatc cttagtgagc gaggaaacca gctccagaat ttcatcaatg atgacgaccg

15781 aatggagctc gcagtcttcg cagttcgcca gctttccaac gcatggaaag ttaccccttc

15841 tgatgccatc gcgctgctac gccaatcagc gtggatggac cgaaatttgt cctaatcgag

15901 caaatctagg caccctaaaa gtcgtcctat aaccacaaag ttagaccatc atgcaaagtg

15961 gggtatagag ggtgccagcc ccaccccagc agtccccctc agccacattt ctggcttgcc

16021 ccgcgctttt cttctttctc tctttttctc tctctttctc aatctaatct cacacacgaa

16081 ggtcaaacag ggcacccgcc gacgcgaagc ggaggcgtat tcttgcctct ccccctcatt

16141 gcatgatggt ctaaatctgt ctactatgta accaatgaac tgttagaaca aacccacaca

16201 taggcggcag catggctaaa aacttcatat cagaacaatt ctcggccatg tgccgcgact

16261 taacgaacct gtcctctctt atcaaacgac taccgccacg gtacgcaaaa gttgcggcca

16321 tccctcctac cggaaaagga atggagaacg agcccgtcaa caagattgtc gtaactgaac

16381 aaactgggcg cgaagctctt gagctggcag cgcacagcta cagagatctt cacatcaacc

16441 cagactattc gcagaagtct gccaggcgca ccgtgggggt actctggttc tccccttcca

16501 ggattggtgt agctgacgaa attgcggcga cggttgaacg catcaacgcg gccaaagctg

16561 gtatcgagga gttcattatc tcgacgtacc ccaccaggca agagaggttt gaagcacttc

16621 gagcggactg ccctggtgtc atgactctcc acctttacag acaaattcgg tgctatacca

16681 atggcgacat cgactctatc cgcttcacct ggcaacggaa ggactctttg aagaagcccg

16741 taaaagagga gcttttgcaa cgcatcagag aggagctgga gcgatctggg ccagactatc

16801 aacttcccct ggagcagctt atccagaaga tcgccagcac cccggagcct tatctccggg

16861 agcgaaggga agtaaaggtt caaccggtgg caaacgtcat ggccgcaggg gtactcaaaa

16921 ccgttaccgc gccaatgccc ctgatcgtgc tccaggataa ggatgttcag ctcaaattgc

16981 ttcgtaactt tgacgcctca gaacaacgca aaactcgatc tgataaagcg gcatcggaaa

17041 tccttggcac cttcggtgga gtcactatcg aatccttccc tggatgaatc agcgtgaccg

17101 cgtaacaagg cttttccgca gccggaaggg ctttgtccca cacaaacaat tccaatcgac

17161 agcagcatga tatataatta tgataattac cgtattatcg taattatcgt aaacattaca

17221 aattaaaagg aacgttctca tggcaagaaa attggttgag tttgacgatg tagcggctgc

17281 ggcccaaaag ctcaaggacg ccggtaaacg cccaacggtc atcgctatca gagacatcat

17341 tggcaagggg agctttacca ccatttcaac gtatctcaaa cagtggtcag aggaacactc

17401 cctcgatgaa gagctggtag aggtagtcct tccagaatcg gttatgagcg atgctgagct

17461 cttcctacag aagatctata cggtagccaa agcaagcgcc gatgaacagc ttgagcgcga

17521 gcgtgaactg ctacgacaga aagaaattga gtaccaggaa gatatgcagc aagccgtgga

17581 catggcaaat gatgccaccg aacgggctga gctactggaa gaacaacttg aagctctcac

17641 caacaaaaaa tcagagctcg atgcggccct tggtaaggca gaaaactcgc tatccctcaa

17701 gtctgcggag ctagaacgct cactggctga cattgataag ctggaaaagc ggatcgttga

17761 gttggagggc aagctggagg cgaaagccgc agatctgacc cgagcacagg atcacctgga

17821 gcaagctaaa agcgaaaatc gctctttgag ccaaaaattg gcaactacag agggtgagtt

17881 ggaggagcaa aaaggcaaga gtatagagca gacagaaaag ctccgggccg ctcaggaaca

17941 aaaaacatcg ttgaaagagc agctcgaaaa tgtacaggcc aaactggccc aatcacagga

18001 ctcccttgcc acagccaaag ctcatggcga atccgctgaa cgagagtgcc agcgcctatc

18061 tggagaggta gaaaagctgg acgccaaatt atccagcgcc gaagcagaag ctcgcgctct

18121 tgttcaagac aaaggcatga tggctggtca gttgcaggaa aaagaccagc aggccaagag

18181 cctcgaacaa agactcaatg atgcactgac caaaatttct gggttggagc aagagctcgc

18241 taaagctggt aagggaggga aaaagaaaga ggaaaactga tggcgcgaaa ggctaaatat

18301 tcagaagagt ggcgacacag agctgcggct cttcaaacca agattgagga ggctatgaca

18361 ctagccacct catctattgg cgactatcgc tggttacacc gtctccatag ttgggttacg

18421 gaggtggctc aaggtaaagc cccagactgg tggacagatc tggattgtga agtatccctc

18481 ccccgagaag aaaagcggat cagtacgttc ctctcgacac aaaagaagcg catcactctc

18541 cagatgtgtt tgtcgtaggg ggcctcatga aacaacttcc tcctgacaca ccagaacaat

18601 cactgatcac tcagtacaaa gggcctcgcc ttgtcgttaa ggcctacgct ggaacgggta

18661 aaaccacgac actggtgaag tacgcccaca acaacctcga ttcacgtatc ctctacttgg

18721 catacaacag ggctatccgc gacgaggcaa gagaaaagtt tcctgcaaac gtagactgca

18781 aaacgtccca ccagcttgcc tacgccacta taggaagggg ctaccagcac aaactctccg

18841 gcaacctaag gctcaccgat attgcccaag cggtgaatac caagaactgg acgtttgcca

18901 aagatattct cgatacgctc aacgccttta tgtgtagtgc agacatgcgg attctttata

18961 cgcattttgc tcgcgccgat acgggtaaag tgcttacgtc caaacaggag agataccaaa

19021 tccaggtggt cgaaggtgct gagctcatat ggaaacggat gacaaacgtt caagatccgt

19081 tcccgaccgt acacgattgc tacctcaaac agtatcagct cgggatgccg aatctgtctc

19141 gccggtacac caccattctt tttgatgagg cacaagacgc taaccccgta acaagtagca

19201 tcgtcctaca gcagaactgc aaggtaatcc tggttggaga tcgccaccag cagatctata

19261 ggttcagagg cgcaaacaac gcccttgata gcaaagagct catgaacgcc gaccaactct

19321 atctcactca tagcttccgc tttggcccca acgtttcgct ggtggcaaac gcccttcttg

19381 aactcaaagg tgaaacacga cctgttgttg gccggggacc agcagatcag gtactcatgt

19441 ttttaccagg tgacgtgggc caccgcgcaa tacttcaccg aaccgtcatg ggggttatag

19501 agacggcgct ctctgcgacc gaatccggag cgcaggtatt ctgggtcggt ggaatcgacg

19561 cttaccagat caatgagctc caggatttgt actggttttc gatggcagag ccagaccggg

19621 taaagaataa gaaactgctt gatgagtatg aagactactt cgagtatcaa gaagtagcga

19681 aggcgaccaa agaccctgag atgatgaggg ctgtcaagat catcaacagc tacgatgaaa

19741 tccctgaacg actcaccact ctacgacgca atacagtcaa agaagagttt ggggctgaca

19801 ttacggtctc aacagctcat cggtgcaaag ggttggagtg ggactttgtt cagctctatg

19861 acgactttcc ggatgtcctg gacccagagc tcgacccaat ggcccgtgac gatgaaataa

19921 acctgctcta cgttgcatcc accagagcga tgcgaatcct tgcgttgaac agcgctgtcg

19981 agatggttat ccgctacatc acccaaaaac gcatggtcga gaagcagatg aagatggccg

20041 cagaagcgac agaagttgaa gaggacacga ccaaatagtt tggtcaattc tttcacatgt

20101 gaaagttgac aaataaaacg ctctaagcgc cctaaatggg cgcttacacc tgcctaattt

20161 cacgcctccc acctctacca tatcgagcat gggaaaatac gtgcgtgaga ctatgaaaaa

20221 atcacctttg aatttactgc tccttgcagc gctcacgctg ggggcatcac accaggcttg

20281 ggctcaagat ggcacaagac ctggttttta tgagcgaaag gaagagggtt ggttctggta

20341 caaggaagag cccaaagaac cagagaaaaa acccgaaaag cccaaaccaa agcctgtggc

20401 agaagcgaag cctacacagc ctaagcctgc tgctccgctt ccgagcggcc cagaaatgtt

20461 ctcagcggaa tggttccggg aaaacttacc caagtacaaa gaccttgctt ggaacaatcc

20521 taccgttgaa aacgtcagga cgtttctcta cttgcaacga tttgcgatag atcgctctga

20581 acaattttcc gatgctacag agctggcggt cgtaggtgat cctttcttgg atgaaattac

20641 tcgacgtcct gctgccacgt ttgcctcaca acaagttgat cgtgacgctg gtaacgccaa

20701 aaacatgcta ctcaaaagcg tagccgaacg cgtagggata ttcttcttct acaagtccga

20761 cgatgactac agtgacttgc aagcaccgct catcaagatg ttggaacaag gagaaggatt

20821 ctcgatcatt cctgtatcta tggacggcaa accactcccc agtgggcttt tcccccatta

20881 caaaaccgat gaaggccatg ccaaacaact tggtatcgta actttccctg ctgtttacct

20941 cgcatctcca gacggtcagt tcgctcctat aggacaaggg ccaatgtctc ttcctgagct

21001 gaatcacagg attttggtcg cagcaaaacg caatggttgg gtcacagacg aagagtttaa

21061 ccgtacacgt ccggtactca acctagaaaa caacatagcc gaacgcttgg cctcaccaga

21121 gctgggctct gacctcaaac aactatctca agcgagcggc gataaagaca actttgtgcc

21181 accggaacaa ctcatgaagt acatccggga caaattacag gagaactaag atggtcacgc

21241 acaagacatt aaaaaggagc ctgcttgccc tgagtgtggc ggccagtctc gtcatggcac

21301 cgacaggggc gatagccgct aacggcctcc aatcacagat ggacaaactc ttcaatgaaa

21361 tgagcaacac aacaccacct ggggtttatg aaagccaacg acgtggcgtt ttagctggtg

21421 gccggttcac tgcaaagaca cgaatcttcg acgagaacct ggtgagcttt gcccctccat

21481 catggaaagc tggttgcggt ggtgtagatc tgttcggcgg ttcgctttcc ttcattaacg

21541 cggatcaaat tgtccaactc cttcgggctg tagcagccaa cgccaagggc tatgccttcc

21601 agcttgcgct cgataacgtt ttcccggacg gagcgaagtg gatagagaac ttccagaaga

21661 aagtgcaagc gctcaaccaa catctgggca actcctgcca gctcgctcaa ggtttcgtga

21721 acgacctaac cagcggcatg gaccttaaac acaaaactga tgcttctata accgcgacaa

21781 cttccggcct gtatgaagac ttcttcgggt ccaagcagga aaccagcggc aagagtcctc

21841 tggaagaact gaaagccaac aaacctgacg aatacaacaa gatgattggc aacatcgtct

21901 ggaagcaact caagagcaac aacgccaaca cctggttcca gtacggggat aacacccttc

21961 ttgaagcgat catgtcttta accggcacag tcatcattgg tgatctggta aacgacccga

22021 actcaaccgg cactggtgcg aaaacaaccc ctctgacgac cctaccaggt aacaagatca

22081 ccttgtcaga cctgatttca ggcggttctg ttgagatcta ttcctgtgat tctgatacga

22141 ccaactgcct gagtgctggc tccagcaata aaactgtcgt gctcaaaggt atcaagaacc

22201 agatcaccga tatgctgtta ggaacaagct ctacacctgg tgtgatctac aaatacgcaa

22261 cgaactctgg aaccttaacc gacccagaaa aggcctttgt ttctaacctc cccggaggga

22321 ttggcaccat tgttcgtaac ttgtctgtcc tttcacagga cggcgctaac ctgttcgcaa

22381 cagagtcatc aggagcgata gccctgacca tgatgtatag cttctcggaa gagttcttcc

22441 gcgcagctcg cattgcgatg gctaacagca aatcacccta caagaaagag gcactagagc

22501 ttctcgcgca atcgcaacag caaatccgtg ctgaatacac aatcctgtcc tctcaatacg

22561 gcgatctggc aagccaaatt gagaaataca acaacctact ggacaacatc cgcaagcaaa

22621 aatacatgct ggcaactttg tccaatcctc ctagcacgaa ctaaggagct attggaatgg

22681 gatctttttc aatccactct atcggtgact ctgcttttct ggagcaaatc ctgattgcag

22741 tatcaatgat caccggcacc ggggatttcg agaagatggt cagtattggc ctgcttcttg

22801 gggtcttgat gatctgtatt cagtccgtct ttcagggcgc aaagcaaatc aacctccagc

22861 aagtgctggt aggttggatt ctatatgcct gtttcttcgg cccaaccaca actgtgacta

22921 tcgaggacgc ttatacaggg caggttcgag tcgtcgccaa tgtccctatt ggggtaggct

22981 ttgctggagg tgtcatatcc aacgtgggat acaccatcac caatttgttt gaaactggat

23041 atggggtaat cgtacccaat gtcacggaaa gccacttttc cgaaacactg aaactgttga

23101 atgacgtcag acggcgagcc tatgacacag gagtttttac tgcgcttaac tcagcaaatg

23161 gaggcggcta tgttgacgtg aggcgttcct ggaacaatta cattcgggaa tgcacgttaa

23221 ccaaagtcga tctcaaccta atgtcccttg atgagttgat gaaccgttca actgactcag

23281 ctttgcgatt caactcacag ctctacggaa ctaggttgta tttgtctaca gcaaaccctg

23341 acggcgctga ctacacatgt actgacggat gggtggctat tagcactgca accgccaacc

23401 taagcagccc ggttgttgtt gatgctctta acagcctact gggtattgac acgtcaactg

23461 gagacaacgc tctaacgaag ctgaccgatt cgcttcaagc gatgggtgcc acaactacgt

23521 catcaatcga ctatctgaaa gccgccgttc tggagcccct ctattatgaa gccgcagcag

23581 gacgttatca ggacctccag gattatggct ctgcattgat ggtcaaccag gctattcagc

23641 aacggaacac acagtgggcc gcagagcagt cgatgttcat gaccgtcgtc cgaccaatgc

23701 tgacgttctt tgaaggcttt atttatgcta taaccccgat cattgctttt attatcgtga

23761 tgggcagctt cggcctccag ttagccggga aatatgtaca aaccatcctc tggattcagc

23821 tatggatgcc agtcctctca attataaacc tgtttgttca taccgccgcg tcaaatgaga

23881 tgtctagcct cagtgctggt ggtctcaact ccatgtacgc tctttcctca actggagatg

23941 ttctgcaaca ctggattgca accggcggca tgttggctgc ggccactccg gtgatttccc

24001 tgtttatcgt cacaggtagc acctacgcct tcaccagctt ggcatcgaga ataagtggtt

24061 ctgaccacgt tgacgaaaag atgcaaacgc cagatctact caagcaaggt ccggttatgc

24121 aaagtcagcc agcgtacaat cacaaccagt tcagtggtgc gattgcaaac ggcgcagaaa

24181 gcatgatcag taccttctcg cttggctcca ccttagcatc aggcgtgagc tccgcacagg

24241 cattacaaag tcagaaatcg gaggctttcc aaagcactct tggtcgaggt ttttctgatg

24301 gagtaagtca ggatcaagcc tattcaagac tctccaatgt cgggcgcaac gtttcgtcgc

24361 aaaacacagc tcaaagccaa ttgatcaacc agcaagccaa gaacttcatg gataagttcc

24421 aggtggatga tagccactct gatgctgtca aaggtgcttt tgccatgcag gctatgggca

24481 cactcgatgt tgacgaagct gcgtccatgc ttatgcctat ggttggcaag gccagggcag

24541 caatgaaggc cgctgctggt gtgaaatcaa acagtacagc cctagttcct gctggtggta

24601 atggcgaatc aggcggcggc agtgatgtcc tggacatcaa agcgcaagcg aagggagcaa

24661 cagagtcatc aactcaagac tcttcaagct ggtcagcgag tgatgtgtcc cagttcatga

24721 agggtgtgag ctattcgcaa accgatagcc aggcgttgac aaatcaatta gcgcagggtt

24781 tcagccgttc tggaagcgag tcattcaagc aaacctgggg cgatagctta tcccagaacc

24841 tatccaagtc cgcttcggaa ctggtgtctg catcggacac cttcacaaca atgagtcagc

24901 tccaaaacca aatgggctcc atgactaata ccgactttaa aactctcggt ggtgcagtag

24961 cacaaacccc tgcggccatg aaccaactga atgactattt ccgaaatgcc gcgccgcaat

25021 cggttaaaga cgaggcggct tcactacagc aaagatacca agcctacgga atgtctcctc

25081 aagtggccca ggcagcagcg cgaatgacag caatgaccaa ctccaaaaat tacgaacagg

25141 gtaaggagct tggcgggtat caagccgcac tacaggcgat caataccgcg tccggtcgca

25201 acggagcatt tagtggtgat gcttacggaa ataacggcat tgaaggcccg aatgttcaag

25261 gcctaccagg tcaggttcaa ggggctgtag gcagtggacc taacatccca accggattcc

25321 gggagaatgt ggctgggatg gctggaacta atccggcatc agaagctggg cagttaccaa

25381 cgaatagccc ccttgttcaa aatgaacatg cagccggtac gtcagctctt cataaccaag

25441 cacagcaaac agagcgaaat gtatctgctc ctgaactgaa aaaagcccaa gataacctta

25501 tgaactcgct tccggaaatg tcttggagcg cttcggcgtg gggagcgtgg gacaactcca

25561 gtgattggat gggccgcaga gctgaacaag caggtggagc tctcattgct ggtggtcaag

25621 ctggcgctga tgcgttctca agagcgatgg atcaaatgag aacgatgaca cctgaacaac

25681 gcgaccaatt catcgcggcc actcaacgcg gcgaccaggc cgtgcaagag gagtttggct

25741 gggccggtga tgcgatggtc ggtatggcta aacttggccg caacgtcatg ggagctgctg

25801 caagcggcta tgatgcagcg aaggagtggt taactggtaa atctgatcta tcggaagccg

25861 ctaaagggat gagcattgag gaacgcggcg cgttctatgc agcagcgcta tcctctgccg

25921 cagaagctgg tggtggagcc gcgcagcagt ttatgaacca gtacggtgat gagttcaaag

25981 aaacgatgca gtctatcgct caaagccgtt atgggctgac tgaatcccaa gccgctgttt

26041 atgccgaatc gtttgacaca aacgaaggtc gcatgaacca ggctgttcag aatctgaaaa

26101 tggagtatgc agaacgtaac ccggatggct caccgatgat gcaaggaggt cagcctgttc

26161 tttctcagca aaacgaagag tttacggaca agctggtaaa cgtattgcag aactcgacgg

26221 aagctggaga ccgttcaggt agctatttga ctgccgtcag ggggtacaac atagcaaatc

26281 aaaggttcta aaagaccaac caaataataa gggggccagc aggccccctt attcgtctcc

26341 agctccaata tggaagcgct ggtcaaaatc ataggaatct tgaaacccac cagtttgttt

26401 ttttctgcgc cgggtaggtg gtgcctcttt atatgctgac cgcgcaacgc ggataaagca

26461 cgttagtcca aacagagccc cgacaaccaa agcaacttgc cactggaagt aagcgatccc

26521 tccccaaaag ataacagcag cccaaaggct tttgtgaacc agaaatagga aacgagcacc

26581 ccaaacgccg aagggagcca gcgccacacc aagtccccaa ggtgtgtcag ccatcatggc

26641 tattcccaga agcaccattc ccagcagaag aatattgacc acatgtttca tatcaacccc

26701 cttactcttt acctccagtt taccacggca tcaaaaaatg caactgtgaa aatttgacta

26761 ttttttgcta aacaaccatt ccattggcaa cttcttcgat tagaatttgc cctcaaactt

26821 tcacatgtga aagtttgccc ttacggaaca aggcgtttat tgacaaagca ttacatagtt

26881 gaacatgtca aacaaatcgc atacactatg gaataggcca ttgctaacgc tttggtcccc

26941 atctgcaaca ccgcagagtt ctctttctca aatatctctt tctgtaaaca ccatcatcct

27001 tgtgtggtcg ggatgcgcct ttgccttccc tgccctcctc agctcctgct gtattcgcaa

27061 tttcagccct cgctgtatcc ctaaaatcac ttatacctaa acgctattta atatcacaaa

27121 ataccgcact ccccgtgcgc tatttccgcc attcttttgg cgcactttcc attgctatcg

27181 caaaaatatc cgtatcccat tgattactaa aagtattgta actttgttcc gtcggtacaa

27241 agttaagtgt atgtttttaa aggtttttta ttgttgacag catcattatt tgggcttgta

27301 aaggcgcaaa atgtgtcagt ttgtgacaat agaagaactc taaaagcgtt catcgaataa

27361 cgcacttttt gtatggctaa caacgcagtt taaagggtgg caaacaatgg ataaatgcca

27421 attgatagac atcccaagcg acccagagaa gaaacgtgag tggatcaagt acaaactcaa

27481 gatccagggg ctttctctgg ccgcattggg cagaaaacac aaaacatctc ggcaggtggt

27541 gtctacggca ctctataagc ccagtccacg ctgggaacat gagatagcta cagctttggg

27601 tgtgaagccg tctgagattt ggccggagcg gtacgacgaa gaacacgaaa tacccctcag

27661 acataaggag gcaagctgat gaagaacaaa gccaaggcgc tagttctgtc tgcggctctc

27721 ctttcatcaa cagcgaatgc tattgacctg agcggaacca tcttcgacaa agcagcgaaa

27781 gcatataacc tcgaccctct tctagtgtat tcggtcgcat tggccgaatc tgcatcaggg

27841 agaggtaatg gctctataag tccttggcct tggacgcttc gcgttcctgg gcttcctttc

27901 tatgctaagt cggaagatca ggcaaaggct aagctcgctg agtttcagca gcagtacggt

27961 cgtgccattg atgtcgggtt tatgcaagtg agcatccggt ggaatggtca tagagtttct

28021 tctccagcag atcttctcga cccagagacc aacgtcatgg ttggggcaga ggtgctatca

28081 gaagccattc agtcatctcc aaatgacttg gagcttggcg ttggccgcta tcacgcctgg

28141 gaagacgaaa tccgagccag aaactatggt agccgagtct tggctatcta tcgcaacctt

28201 cgtgatttgt gaggggggcg gaatgttgga actggatatt attggtgcgt gggatgcaag

28261 agccgtcaac ctcgatcaag aagaagctga tagaaacgtc tacgagttcg atctgacatt

28321 gtggaacctg ctatccactc tggcaaaaga acgtccagat gatgcggcct cacaattttc

28381 tttgggcatg gacaccgttc aaaagctgtc actggcaaca ccttcccaat tggaagctct

28441 ggcctctggc gtgttgatct ctttcaaact cgaaacagca gagcagaaca tcatcacgcg

28501 actctctggc gactacgacc ctgtagtttt tatcaaccat agtgttgatg aatttgatgc

28561 tgcctactgg ttgctattta accgcgtcgc atcgagagac ccggagatgg caaaggaagt

28621 tttcggggtt tcgagagagc ttgcggagct ggtggctaag gcaacagaca gccagttgcg

28681 ccacatgtct ggaacaacgg ttacgcattt tacgcttcgt tttgctccga gcatcattga

28741 agaaattctc gatgacagcc gggaagagtt aacacacccg gtattgaaaa aactgcaaca

28801 gtctctacag ggacgtggga ggtggagatg aacattggca actctggtac attgggtcgc

28861 tgggttacag ctcgacacat ggcccttgct gggtacatca caaaaatcat catgatcgag

28921 actggcctga cctacaaaca ggtcagacgg ctttaccagg atctggagag ggacggatat

28981 actctggaac gaaaatccag aactttccgg ggtggtgcga cactgattca tagtcacaca

29041 tccaagatac aggcctctct tctaatgcag ctctacttca acattggtgg agaagccgtg

29101 ttgcggtctg tgaacatcaa agccttgaac aaggcattta gaatgtatca cgcaatccgc

29161 aaagaagtgc ccggaatgaa aggtgctcgg tgggctccgt ttgatattac tgatgcctgg

29221 tgtcttgctt cggagctgag aagtggggac gcaatgctgg aggtgtgcga caactgcaag

29281 tgtacgtact tcacctctgt taatcaaaga acctgcgttg aatgtccgtt ctgcaaagaa

29341 caaggaaggc atggtggtgg ggagaaagag tgtgcttgag tagactatga catttcggac

29401 cagaagatga gcgcccaaac tttgcggcgc tcattttttt acttcttctg tgggatacgg

29461 tagcgctcca gctcgacagc tcccaacccc ttgaaagcat ccgggcgacg tccttgccca

29521 ctccacgaaa ctccgtcttt tgaatattta gcattatcag gttctgatct gctcgtgaac

29581 atttcgttga gcaagccgat gtccacaccg caggattcca tgtcgctcat tattcgctca

29641 gcctgagctc gcttttcttt ttcttcttct tctcgctttt tgtactcttc ctccagttca

29701 ttgagaacgc ccttcattct gtcaatgatc tcccgaactt catctaacgg gagtcctcgc

29761 aacagggtgc gaatgcggct tttacgcttt aactctgcga ttattaactc tctacgttca

29821 gccgctgata gcgtagagaa ctcctcgtgg tctttcatgt catacggagc ccatcagttt

29881 tcctttaagt gaatcacgtt gagagttcag ttttgcgtca gtctgcactg gcgaattttg

29941 atgctgtgca ggttgactct ctgaactctg ttgctcaaaa gacccaatgt tgccagaaaa

30001 tcgaagagaa taaagaccca atagagcaag ggcgcggagc cgatcactac gcgccttatg

30061 ctgcatctgg gacagttcac gatagagctc cggaaaggct tgctccgata tgttcagatt

30121 agatattttt ccatcccatt tgctaccagc cacaacgacc tcctatctat cagccgcaga

30181 accagaagcc cctggcattg gatgccacgg attcgttggg cagtacgatc ctgctcttag

30241 ggaaaagctc cttggccgca tcttggtaag cctcggcacc gccgccagcc agcagaacta

30301 cgtcagcatc catcccgtcc tcacgcattg acttccgcat agggatcaag gcgttttgag

30361 cgactttggt tgaggctttc ttgaagtagt ctttgatcga taccttttca ccgtagagga

30421 agatttcggc cttaccggca cgaatagctt tctcgatctt ttcgatgcca ggggcaccgc

30481 cgtggtcttc ctgaattagc cggtccgttt cctgtagcaa caccgacatc gccttgaggc

30541 tggtgccaga tgagtgatag cggacctctc cctcttcaag agctacccag tctacagaaa

30601 agaacccagg gtcaattaca acggtttttc ctccctggat aatctccagg aggtcttcat

30661 ctttggttga acttacaaca tccatgtaag caccggcagg ttgaggtaca accacgacag

30721 acttaaccgc taccgatcgt tttggcgtga tctggtgttc gccctcaagc cgagctttca

30781 acgcctctct gcgctctacg tccatgtact gactaacagg caggccagtc accagcacat

30841 cgatctcctt ctgctcggac atcaggagtg cagcgtagaa aagagccttg tatggattgg

30901 tcgaaggata gtcgccgtga agctcacgct cccatccttg caatcggtca ggctcgacgc

30961 ctgcaaccca tttctctcca tcaatcacaa cctgaatgca ggtccctgca ccgccagtta

31021 actgttgtgg catcagttcc aatggacctg cccccaccgg catgacgact gtgcgagctt

31081 cctcaccttt ataccccatt gccattttca ggttggagta accaatatcc aaacccagaa

31141 caaattgact catgaaatcc tccaaagatt gcttttagat tgcttttcga ttgcttgccg

31201 cgtgttggat agagaataag gctaaaagcg gggacagatg gcctgtgtaa ttgcccaaaa

31261 ggggcatata ggttgctttt cggtggcttt ttgatcgttg agtaagggaa cctatgggaa

31321 cgctgcacgg ataggctcag ataaacagac cttaccctcg catcgagaac cgcttgccct

31381 ccagcatcga gagacggtgg taaagaggca tttggaatct ttgatgccat atccaatata

31441 tctggaatct ttacatatag attcatattt aaagaggctg tgaaagaata agagcatcaa

31501 gattccagat agatagaggg aaatttgaca aattccaaag atgggttagc ctagtgacag

31561 aactagattc caatattgga ataatcagct ttaaattcca gatagatagt tatgtggata

31621 ggaattggat aggaattggg agggtattga ggtgagtcta ccaacagagc gatggctaga

31681 tctgctagat ccgaagctcg aagaagaacg atccgagata acagaggatc tgctagaggc

31741 agagggacga gagtttgttg cagaggtacg gagcaaactg gatcacgcct tagcggttct

31801 tgctgtcgag gcgcagcagg aagcggacat gtactggaac gcgcacaaat cagcgcgtga

31861 agaagcgtca gaggacgaac aagggcgtgt cggtacacgg gttcgcattc taggcgtatc

31921 actcgttgca gagtggtatc gcaacagatt tgtcgaacaa gttcccggac aaaagaaaag

31981 ggttctatcg acacatatca agaagggtcg cggtcatgcc tacagcatgt cgcacttcaa

32041 gaaagagcct gtctgggcac aagagttgat ccagcaagtt gaaaccaggt atgccgtgtt

32101 aagacaacgc gccactgcct tagcaaaaat tcgccgggcg ctaaacgagt acgagcgcca

32161 gctaaacaag acacatagcg acgaggtgtg acaacatgac agcatctgta gcggccactg

32221 aattggcgaa actggggaaa tgtgaagcga tgatcaagaa agtcgccagc catcctcgcc

32281 ctgccctgtc aaagcgccca caatcgccac aggggacaga tagcaccctt cggggtgagt

32341 tcgctcattt ccgatatgaa gcggcagccc tgcgttttat gagcggcact gcgggggcta

32401 agcgtcgcat ctaccagcta gtgttttcag cgacggtagc agcgggagca ttgacaatgc

32461 tggcggcatg gaccaccagc tagaaagtat cgacggaaca atcatgagca agagaaccaa

32521 agacaaagac ctggagaaac tcgacgtaat caaagactca ccgcaaatga gcctgtttga

32581 gatcattgaa tctccggcca agaaagacga ctactccaac accatcgaga tctacgatgc

32641 gctgccgaag tacatttggg accaaaagcg tgagcatgaa gatttatcca acgctgtagt

32701 gacacgacaa tgcaccatca gaggccagca tttcacggtg aaggtgaagc cagccatcat

32761 cgagaaggat gacggaagaa ccgtgctgat ctacgcggga cagcgagagg aaatccttga

32821 ggatgctcta cgcaagctcg cagtgaacgg gaaaggccat atcatcgagg gcaaggctgg

32881 agtcatgttc actctgtacg aactccagaa agagctctcg aagatgggtc acggttacaa

32941 cctgaacgaa atcaaggaag caatccaggt ttgtcgtggc gcaacactcg aatgtatcag

33001 tgatgacggc gaagccttca tcagctccag cttcttcccg atggtgggac ttaccaccag

33061 aggtgagttt cgcaagaaag gcgggaacgc caggtgctat gtgcagttca acccgctggt

33121 aaacgaatcg atcatgaatc tgtcgtttcg tcagtacaac tacaaaatcg gaatgcaaat

33181 ccgctcccct cttgcacggt acatctacaa gcgaatgagc cactactgga ctcaagcatc

33241 gccagattcg ccgtacacgc catcgcttat cagcttcctg acacagagcc ctcgtgaatt

33301 gagcccacgg atgccggaga acgtcagagc tatgaagctc gctctggagg ccctcatcaa

33361 acaagaggtc ataagcgact acgacgcgaa ccagatcaag gatggccgca gagtcatcga

33421 cgtgcggtac gtcataaggc ctcatgagaa cttcgtgaag caggtgatgg cgtccaacaa

33481 gcgtaagcag cagacagagc tacgggccat caagcaaggc atgatcgacc acgacatcat

33541 tgatgaaccc cagcgtaagg ggaggtagcc accagaagct cccaataccc cagggaaaag

33601 aaagataggc gcgtcagcgc ctttttttat gccagaagaa agcgcacaag gtacgcccac

33661 aaggaaagac cggaaacgga atgcctccgc atatggggga gctgcacgga tagagcgaac

33721 gtgaccccag atgcttcgtg ggctgaggct cgatatatgg gaacgttgca cggtagttgc

33781 aagccgagtc tcagtatcgt gcaagcgtaa gccacgacga aggataagtg ccatgctgct

33841 ccagtggttc ccgtagagtg gttcccgtaa ggtattgata gttaaagggt gaggatgtgg

33901 gggacctgca cgttatgaga tttgatatgt gggtacgttg cacgaaagca ggttctgaac

33961 ctctcgatat atgggaactc tgcacgattg acgaagcaga acaaagattg acagatcagg

34021 acaaacctaa aaaggcagtc aaaatgactc caatagtcca taaagtgatc gtgctcacat

34081 aaaaagaggc caaaagttat ccacatttcc tgtgcataaa cggggttttg gtatgtggga

34141 gcgctgcacg atcacatgtg ggtacgctgc acggagcaaa aaacgaccaa aattgatata

34201 tgggaacgtt gcacgttaag aagagaatat atgggctgac tgcacgtata accttagaca

34261 tgtgggaaca ctgcacgaac accagttctg atatgtggga acgctgcacg aaagctctgt

34321 tttgtaagga ttttgtggaa aaaaaagggg ttccgtgtgg ataacagtgg ataagatcca

34381 agggtatgtg ggggcgttgc acggcaacct atgggttcgc tgcacggcaa cctatgggtt

34441 cgttgcacgg aataaatatg aaaatgccag caatatcaat gcgttgaatg acaactcaga

34501 gcgcctaacc tgtttttaac cgcttttaat cctttataca aagatctaaa gatctttata

34561 tttatccttt tgatcgctga gcaaaaaata atcaacactg ttttttagag aaaagtctcg

34621 tatgcaaggc cgaacaggac gagtatcact aacaagataa gcatcgacag cacaaagtaa

34681 ccagactcgt acaccacagc tttgatcaca gaaacgacgc tgatcgatct tccagagatc

34741 ctcccgatga tccagtgaaa cgcaacgaac gaaccaagcc cacagatcag caccaccgct

34801 acggagacaa gtgcatgttc cctcaggaca gactcactaa ggaaaccata ctgcatcccc

34861 atcaatacaa gcctgccaat gccaaacccc agacccaatg caacagctcc acgaaccagc

34921 acaaccttca acgactggag ccatgtcagt tcaagatcca tccccttacg ggaagaccga

34981 cgcaccagca ccatagatat gcccagcccc aaaaacacca aaaaaacaaa atccaacatt

35041 accacctctg cacaaatcaa tcagaaccac aggcctctcg gccaaatgcg ccaccaggga

35101 gaacccactt ctgcccctcc ggcacaaaaa tcgggagcgt gtaatcctct ctggatacct

35161 gctccaatgc ctcaacatca ctaatctgta tcggcgcaac agcagaacct aaatctatta

35221 cgtcgatttt ttttcttgat accgtcacac caaaggtttg ttcatactga gcaatcttcc

35281 cagctcgttc aggccagtag tgacggatag tggaccagat acgctgtgag ttgtagatgc

35341 aggtcataca cgaagaccgg ctccaaccaa ggcgataagg gactggggcc agaatgcggt

35401 gacgctcgat tacttcccat acctcttcct cagtccaatg aagaacgggc ctccaggcat

35461 caactaaccg tgcagtcttc ccatatcttc tgtcacaggc atgagcctca agctggttgt

35521 acttggatct gtttgcactt tcctcccggc gctcaccagt gatgaaaagg atcttcttcc

35581 ccttgaagcg ctcctggtta ttaagagctc tgcggccaac atcgatcttc aaggctgatg

35641 aacaccacct tgtttggagt gatggcgatt gctgagggaa acgaaggcgt gtaccaggct

35701 tagagcgctt atggtctcta ggcagcacca gaagcccctc aggcgtctct acacgatggg

35761 ggtggctata ggcattgtct ttgagcattt cgccctcaaa gccgccctca agccacgaga

35821 agtacattgg gacacccagt tcttccccga gctgacgaca gtaatcacgc atgaaggccc

35881 aatccatcaa cgaactacct tcctgaccat caacatcatg gtgccagaac tccacctttg

35941 acttatcaac acccatgtcc accagccgca agtacgcagc aatcgagtcc ttgcctccag

36001 acaggcaaac aatgatgtgg tcgtacaaac tcaagtccac atccggcgct gagaagtacg

36061 ttgttctatc gtcacaacgc tgactggaag atataccggt aagaaccgac tccagtgatg

36121 gcaacaccac ctggtcatcg aacaaatctc cttggttttc tcgtctcaac ataagtcgct

36181 cttttttctt tactgggttg tttttatgat ttacagtatt ataaaatgat gctgtgaaaa

36241 gtaaagtttt cagaacgacc agtcactcag gagaacttga taagcaccag aaagcaatct

36301 aaaggtggtt tttcggtgag gttagttcag gggcaacctg tcttgctgat tgaaaatcat

36361 caagaagtgg ttttttggtg tggttacggt ttgagggggt gagtatgtgg gaacgttgca

36421 cggttaaagg gggccgcaca tgaaaacgat aaagcggttc atcgtatggg tgaattacgg

36481 tcttgagggt tggagcattt tcggatcgag cgatgactgg gatgaagcgg tatccatccg

36541 ttcggaagcg atagacgaat gcaatattga tgaggaagac atcatcttgg cagaaaacaa

36601 aaacgagctg gtggtaaaac ctgcggccaa gcaaatgacg gagtggcacc gggaactgga

36661 agccgtcctt atgaccttgg atgactgcca gatggaatgt gacggcatga catgggcagt

36721 aagccaccta ctcaatgacg cgggtgtacc tcatgactgc atgtatggtt ttgtgcgaaa

36781 cgaacagacc aaggacatcg tgacaccgca tttctgggtt gtcctggatg atgggtggtt

36841 ggtggatctg aggctacgca tgtggcttgg cgatcacgac aacattcctc acggggtatt

36901 ccatcccgat aacgagccag gatttttcta caagggagac cccgttcaaa accataaagg

36961 gatgagactg ggcaaagccg tcctggacat catgaccgac ggaaaaattt cacatgtgaa

37021 agttcctgaa cgacaagacg gagagtaata gcgtggaatt tgattacgac aagtcagtat

37081 caaacgcaca tcttgaggcc gcaggctggg gtatggatgc ctttaatcac tccaacccat

37141 ttgagagcca tgtcatctat gtgcgtgact accgcaacga tcacatccgc ctgttcacca

37201 tcaagaaggc tgacttcgac acgataaagc tgccgcttca ccttacctcc gacatgctgg

37261 cttcggtgat cgctgagttc gtatcgaagg cagcaaaagg gaagttgaat acgaaggagt

37321 ccgatacgct ggctcctgcc ttggttggct atgccaaatc aacagaaacc taccggagct

37381 ggcggagagt gtctggaaca acagagcgat tgcatatggt catcaatatc tacgcgggtt

37441 ctggattgct gagacctttc atcgctcggg ctcctgagac cgtcctgacg acccaagagc

37501 tactggtctt ctcatcacag gttaagaaca tggatgtatc caatcatcca gagtggttta

37561 ggggcctgag gtagtcatga aggcagttct ctggatattc gtccttatca ttgcaccttt

37621 cgtcattgcg aaagtggacc agtggcgcaa acgcggcatt ggagacacct gggcgtggtg

37681 gaaatcagag aacatgcctt atgagctgcg ctcggccact ctgtttttat ccgaacagga

37741 catatcaacc acacagccgg tgcccatgca cggcagggtt gatcaggtgt acaaagccaa

37801 gaatggggtt ctcattccct tggacaccaa gctgcgccag gtaaaccaca tctatgagtc

37861 ggacattatc cagctctcgg tttaccgggt aattctgtca cacaagtaca aggcacccgt

37921 tgccaagtac ggctatgtcc gaaccgttgt tgagacagcg gacggggata gagtccgtta

37981 catcaagaca aaccttctca gcgaaaaaga agtggtcaag ttgtggcacc gctatcaatc

38041 catccgttct ggtcaggtaa aaacctcctg ttcctgtggc ggaaagtttc acatgtgaaa

38101 gttctggatt gaattagggt tgcccgaatt gggcagctag gcggggttgc atttttgaaa

38161 agtcctgtac gctggttggt ataggaggtg aatagtggcc cagcttaaac ataaccgctt

38221 cgtcatgttg gggtccttgg tagcgacggt cgtagctgct gcgaatggct tgagttttct

38281 cacccttatg gctggtggaa tgacccttat ccagttcatg gcttggaagc aacaagatgc

38341 tgtccgtagc cggtttaaag ctgcgaagga tgcgtttgaa gcgttgaacg ttattgcgtt

38401 cgacaagcac tgggtaggtt caaccgcgac ggtcgccaaa gtgtcgaaca tgatcactcc

38461 gcccgaacga ctggataagc cctgggcggt gcaagttctg gccgtaacca aaggtgggac

38521 gtggttcgca gtggatctgc aagtgaccgg cactgacaag gttcagatgc ttagcctcca

38581 ccagcttagt gaaaaggcgg caaagaccat gctggccttc gacctcgaag tgtatgaaaa

38641 atttttcgga aaacctgatg ttgcatgagg acaatgtgat ggaaaaagtc aaaagcgtag

38701 atgccgtgga gttcacggca gcagataaga aggcgatgac ccatgcggca tacgcccagt

38761 gtttcttcat tctggcccaa gtgatggcat tcccttccct aggggtggct ggcgctatgg

38821 ttgctgctct gtgcggcatg atgccgtggt tgaccaagtt cgccaaagag gctccatcga

38881 aagcctttgg catggtgatg gcttcactct gccttgctcc tgtgtacggg aaactctgtg

38941 agctggtggt ccacgcgatt cagggagctt gagcttatgg gggtagtacg agttccctac

39001 ctcttggctg agctgaaaga acgcggatgt gctgatgaat ctgctctggc gcaggttatg

39061 cagcctggat gccgaattgg agaggaagac ctacggaagt tggccgcaaa tttagggctt

39121 gaagtttctg agctggcacc agcgccagag aacgcggcca acacccggtt caaagcaaag

39181 ctgagaggag gtttggcctc ctttcttttt gagtacgacg gctgtttccg acatgccgaa

39241 ggctccagcc atgcagaaat gttgggtatc gagcaagaag acgatattgg tcttccgagt

39301 cgagctgctg acgccatgct cctggaaaag atgctttacc aggtaatcgc tcgggctaag

39361 tacatgctcg gcaaaatcga cagcaagttt gtcaggtcag aacaagcaat cgagttccgt

39421 gagcaacttg ctcctgggat cttcaggcca gggtacagag gtttccgatt caaggaggct

39481 gccgctggtg atttgcctac agtcatgatt gacggtcgga agttcaattg cgttgccagt

39541 atagccagag cgcatggact tgatccggta acggtgcgta ggcgcattgc ggacacaagg

39601 aaggccgcag acaaactctc caatgatgag tggaaactca tcctcgccaa gaaaaaaggt

39661 aaagggaaac ccttcaccta tctggacagg acctacagca acattgccca gttctgtcgt

39721 gagcatcagc tcaacaccaa ccttgtttac cagaaagtca aagaccgggc tgatagcgct

39781 gatgaagagt tctggggaca gataatcgaa acatgcaaaa ggaaagacta atggcagaaa

39841 acaaagagat cccctgggag aaagagctca ttgagaagta catgttcacc ctgcataaag

39901 agcaggtgaa agaccggcgc tggcggacca tgttgcgtgt gcttcgcgca tcaggtttcg

39961 tgcttctcat gatcggcttc atcattttgg catctaatcc gggtgggatg ccgtggcaaa

40021 gcgccaaggc tggagcccct cacaccgcgt atatcaacat ccgtggtgaa attgctgctg

40081 gcacactggc cgatgctgat caccttatcc cgtccatcca agcagcattc gacaacccga

40141 attcacaagc tgtcgtgctt cgcataaaca gccctggcgg tagcccggtt caagcaggac

40201 ggatttatga agaagtgaag gcgcagcgag cccttcatcc ggagaaaaag gtctacgcca

40261 tcattgatga catcggtgcc tctggcggtt attacatcgc ctctgctgcg gatgaaatct

40321 atgctgaccg cgccagtctt gtcggttcta tcggcgtcat tagctcaggg tttggattca

40381 ccggcttgat ggacaggctc ggcatcgagc gccgggcaat cacttccgga gagcacaaag

40441 cgcttctcga cccattctcc cctcttacct ctgatatgaa gaaattctgg gagggcgttc

40501 tatcgaaaac ccaccagcag ttcatcgaac gagtgaaggc tgggcggggt gatcgactga

40561 aagacgcccc agaggtgttt tctggattgc tctggaacgg ggagcaggcc aaagaaattg

40621 ggctgattga tggcctgggg agtttgaact ccgtggcgcg agacgtcatc caccagagca

40681 acttggtgga ctacacacca accgaagaca tcatccggcg actgacccaa cgagcgaagc

40741 tcgaagccag ttccttcgtg caagaactca gcgctgtgaa agtttactga taggagcagt

40801 ttgacatgac agaacaaagt accaaatcac tacgtctcgg ggtttttgcc gccattattt

40861 tggggctcgt aggaaccggg ttcggaatct accagctcgt gaaagagaag gatctggcgc

40921 aagagatcgc caacgtgaag ttcacggtca accaggtgaa agatgccgaa ggcgtcacct

40981 tcaaaagcaa ggcagagttt gaggctgctg tggccgaaag catcaataag tttgtcgcgc

41041 agaaacaaca agccgacatc gatcagaagt atgcccagtt cgaggcggca cctgaaaagg

41101 tcgaagacgg caaacacatc tatggggacc ttggtgcccg attcacgctg gtggagttct

41161 ctgatatgga gtgccccttc tgtaagcaat tccacgacac gcccaagcaa attgtggatg

41221 ccagtaaagg caatgtgaat tggcagtgga agcacatgcc tctcgacttc cataacccgg

41281 cagctcacaa ggaagcgttg gccgctgaat gtattgctga acagaagggc aaccgtggct

41341 tctgggtctt cgtgaatgag attttccatc acagcaaagg caacggtgcc ggtgtttctg

41401 acttggcctc tgttgtcacg ggtgttggtg ctgatctgga tgctttccgt gagtgtctca

41461 gctctggcaa gcacgaagat aaagttcaag ctgacatcca gaaagccaag agttacggcg

41521 ttaatggtac tccagcaacc tttgttgtag acaaccagac aggcaagagt cagctactcg

41581 gtggtgctca accggcacaa gccatcatgg cggtgatgcg aaaaatgatg attgagtcgc

41641 aacaagacga ctcagcgaac caataaaaac tttcacatgt gaaagtggag tgagaactga

41701 tgatcaagat tacaactaaa ttggggtgcc ttctggcggg gctgcttgtc ctgtcggctt

41761 gctcaagcgt ccctcagaca agcaacgaat acacgaaagc cctggatgat accaaacagg

41821 tatgtgctgc ctgtgctctg gttggtaatg acctgctggt tgccctcaac aagtcatgcg

41881 acaaacccat aaccccggag actctaacca gcgtcatgaa cagtaactcg atgtttgccg

41941 ccatgatggc aattaactcc attggtggaa ccgaccttta tcaggtttac cgtgatgcgg

42001 ctatcgacac cctgcgatgc aatgagatgg acacttggcc tgaacggacc aaggtgcgtt

42061 tccagcagcc cgacatgcaa aaggcgctgg ccttgagggt ttctgtcaga cagcagaatg

42121 caaattaact ttcacatgtg aaagtcaggt ccctcagggg cctgatttca ctatgaggtg

42181 taccaatgga gttacaagaa gcaaaaaatg ctctcgatag ccttcacccc cacaaggcct

42241 cagccccttt gaggcttgtc atccaccagc ctggagggat tggtggaacc cctaccgtag

42301 gggtgaaagc aattcatgct gggtttgatt gggacagcaa caccatcctg atctacccgg

42361 aagagcagct cacccggttg acgccggatg aggtcgccgc catcacgaag tcagtatcga

42421 agggacagtc ctggcattca tatcagcagt tcaagaagta tcgggagcag ttggccgaag

42481 ccacggagga aattaatagg ctcagggctg agctgggcag gtatcagaat aacgggaggg

42541 ggtaatgcta aaacgcggaa ttatcaatct cgctgctagt tacatcattg ttgatgccct

42601 actccggaat gcagcaattt ggatctttgg cttgtccttc tccattggtg gcacttacgt

42661 cgctggcgag gccagtacat ggggcgttta cctagccact tctggtgcaa tgacactctg

42721 ttctgtcgta acagcctacc tgctcgtgac ctatcaccgc tgggggctga tgacggccag

42781 ggtctggctg ctattgagtg cctgcctaaa cggctatgcc gtctatttga gcagccacaa

42841 catccagttg gtggtggcac tgctttccag cctgtttatt gctttgtgga tgctcaagac

42901 ccttgagcaa ccagcagtga aggggacgta caaggtaatc gctgatcttc accgtcagct

42961 atggggaatg ttgaagggac aaacacaatg acgacgaata ctcaaaacgc gaacgccaac

43021 caggttcggt ctttacgcga catgcttgtc cccgccctgt tgttctatgt ggtgatgacg

43081 gccatgtttg tgggtcttga cgcattcatg gataaaccca ccagcatgaa cctgccattt

43141 atgccgttcc ttgtctcgat ggtgagtttt accagtgacg cacgtcgagc gtgggactgg

43201 cggaacggga ccaaggttgt ggccgtattg actgcggtag ccatgctgtt ggcattcatc

43261 tatcaactcg cggtcggaga ggtaaatctc ttaggggttg gtatataccc ggccacagcc

43321 atccttctgt tggcaatcac ctgggtgatt cgcgctatcg gaaagacggc tcctttccag

43381 ttcctgggga gacacctggc gcggtttggt gcatcgaagt gggtccagcg taccgccgca

43441 gtcattgtgc tcgcgggtgg ccttgccatc actgtctatg cctactggct taaccacggg

43501 agctgatgat gatcattgca acgaagagcg gcttgctggt ggccgcagaa ctaatcaagg

43561 aagaggccgg gtactggcta ctacagcctc gtgaccaaaa gacgccggtc agagtgaata

43621 agcaagatga caataaacgc gctttcacgc atatgggaga cgcccttcgc tgggcaggtg

43681 atcctgagct tgcaaagcag ttcgatgccg agggggaaga acatgcaaat tcgtgactac

43741 atgacaaagt tgtttgaagc atttggtgat gtagaagaag tcacccgaga aatgcttctg

43801 gagcaggcgg agctcattca tacgatcagc gataagtgtc agagcacagg cctgtttctg

43861 gatagtcagg ttcgtttcaa ccagttcgtt caagagattg aggctgacga caatgtagag

43921 gatcggttgc ttcatgcttg gtgctgggta atggaccgaa tagtgaaggc accaacatcc

43981 tttcacatgg atggggctgt gattttgaca atgcctctgg tcgccagata cctgccacca

44041 gttgaacggg agccggaaac catcgtggtg aatctcgatg aggactacaa ggctcctgta

44101 ggcaaccaaa cactctgcga gctcattatg gaacggaggc attggccgca aggtgcaacg

44161 tgcgcgaccc aagaagcgga tggtgaaatc ctctactggg acgccccggt tcaggtagta

44221 gaggaaggca gaaaggccgc tggcaagcat ggcatgatgg ctgaaatagg attaaagcat

44281 caagtagact tttggttttc tgacatggcc gaaactcggc tcgcaaccga ttggaacacc

44341 gccgtcatca cacctcactg cttgctactt tcctatcttg atgtgctcca aaagaacaaa

44401 gtgccgtttg atgagggggt gcggctcgct gccgaatggg taacgcaact tggtggggag

44461 tctcgtaaag ataccgagga agagccggaa gctgatgcta cggtgctttc ccttgggcga

44521 gccacagctc attgctttaa accttatccg gacacacaaa atttctatta cgaggcctaa

44581 gcctcaccca aacaggagga tcaacatgcg ataccaggta tttaaaacga aggaaggggg

44641 cctgccggtg tttactgcac cgtggtactg gttggcctct gccattgctc actggtcatc

44701 tcttaactgg gatgcctgtc gaatcgtaga cagcaaggcg gataaaacaa tgctctgctg

44761 ggccaaggct ctaccggctg caaaaaagat ggagtgatta tgaggttcgg ttttgcaaaa

44821 aagacgggga tttcagcgtt cctatcagca atgctggtgc tggctcctca tgtgtgggca

44881 gagaccttca ctgcaaaggt tgtgggggtg tctgatggtg atacggtcaa agtgctaacg

44941 gaacaaagct gcgataccgg aaaagactgc cggagtggca agatccagta ccgagtaagg

45001 ctcgcggaga tcgatactcc agagaaaaag cagccatacg gctcaaaggc gaagcaggcc

45061 ttatcagatc tggtgtttgg tcgaatgatc aaagtggagc aaatcgacaa agaccgttat

45121 agccgcctgg ttgccaatct ctatgtcgat ggcaaatggg tcaatgccga aatggtccgt

45181 tctgggagtg cgtgggtgta ccggcagtac gccaaaacac cggagctgtt caagctggag

45241 accgaggcca aagccgataa gcgaggtctc tgggcattac cggaatcgga gagaactccc

45301 ccttgggagt ggcgaagaaa gcactaacca caccccagag tatgcaggtg taaacagtaa

45361 ccaagaaaac aaacaggaat aacgatgaac aaaagcgaac tgattatgaa agtggccgaa

45421 gacgctgata ttagcaaagc aaaggccgaa gctgcggtaa atgcgctgat caactcagtg

45481 aaagaggtgc ttaaagcggg tgggacagta gcgcttactg ggtttggtac tttccacgtt

45541 aaggaacgcg cagcgcgaac cggacggaat ccccagactg gagagaacat ccagatcgcg

45601 gcggccaaca ttcctgggtt caaagctggt aaggggttga aagactccgt gaactagatc

45661 ttaatcatgt gacacatgaa taaggtttac ataaggctcc tttcgtggga gccttattca

45721 tatcagacat gaataagggg gcggaatgag gttgtcgtca actcaaaagg acgttttgtt

45781 cattctatac gcaattgagg ccggtggaaa agccgagcct gtaccaggtg taaaaatact

45841 ggagatgatc aactcagctc gccagagcgg tattcatgga acgaacttcc gaacgtcatg

45901 ccacacgctg gtggagaatg gcctgttgaa caagtaccgg aatgcttctc taaagctggc

45961 ctttaggctg accgacgatg gcagggaacg tgcaggagag atataccgca aacggctgga

46021 ggaagtgcaa gataagtaat acgccccata acggggcgta tcgagttaaa cggcaaagcc

46081 gtagtgggtg ccgtccggaa gttcaacatc gaggcgtagc ttgccaccgg aggcctcaac

46141 gtaccgctta atggacgaca gcttgagatc tcgtccaggt ttttccatct cggagactgt

46201 cggctgtctc acacccagag atgcagcaat ttccccctgg gtaaggttca tgcgatcacg

46261 aagctctgcc aagtggatgt tcagtaacat ctcagtggcc gctttctgtg ctttggcaac

46321 aacctcaggc ttttctgttg ccagcatttg gtcaagagtt cttgccataa cgtcactcct

46381 tcttcaattt atccaagtgc gccgcaaact cgcggtctgc aattgggatc attacttcat

46441 aaaacctttt ctcgtccccg gtcttgttac cggcgcagag aagaatcccc ttacgctttg

46501 gatcgaacgc aaagaatgcc ctgataggat ctcctttgct ttggacccgc agctctttca

46561 tgttgctgta ggatgaaccg ttaacagtat ccgcgtatgg tctcgacagc atgggacctc

46621 tatctcgcag caccatcatc gaagccagca cgttggctct atcggtatca tccagtgcat

46681 cgaaccactc atcaaaggtg tcagttgtct cgatgaccca catggtttct ccttacttaa

46741 tataggtgca atcctatata gattcaaacc tataatcaag aaaaatcggc ggctatgggg

46801 gacgaaatgc cggtaagtag tcatgtgact aaaatagggg gtagaatagg cctcaatata

46861 gtcatgtgac taaaaggtgt actgaatgaa caacctgccc ctactgctcg acgcaagaga

46921 agccattgac tactaccacc agcatccaga catgactgat gcagaaaagg cctatgtggt

46981 cgcgttccta agcggagagg ggcgctcaaa cagtcagatc agagaggaac tggggattga

47041 gaaggtatac acggttacac acctgaaacg cgccggtacg ctgtcggagg aagagctcac

47101 actctggctc agaaatccac gcaagatcac cctgggacat gtgagagccg tggctaagtt

47161 acctatcagc aagagagaaa agctcctgag ggatctcctg cataccagga caccggttca

47221 tacatatgag gcgatagcga agggcaaaga agtggatagg gatgctgaca ttaagcgtct

47281 ggaaaccctg atgagcgatg cgacgggtag gcccatcaaa attcgctata accccgcgaa

47341 gcgctccggg gagctgacgc tgggattctt cacgctcgat gacctggatg acgtgtgcaa

47401 ggctctgggg tttgatccaa gcgagcagat gtaaaccaga aaactttcac atgtgaaagt

47461 cttgcaaggt gtctttttat tgtttacagt atcataaaat gaatctgtaa aggtgcgcga

47521 tgaatgtgct tccaacgaaa agctggcctt gggcgttata actcaaccag acctgagtgg

47581 ctggcggcgt accgaaacgc aagatgtatg agaagctcag gtaaagaacc tgatgcttct

47641 ctttccggga tagcttggaa ggcgcaactc attgttgccc atgaaagagg cagaattgac

47701 agcctggctg tccctttgga tgccagattg atagcaaaga acctcatcga cgagatcctt

47761 gctgaggaga actgatggac gaaaaaatta cctacgaaga aatgcttgaa caactcgacc

47821 agaaaggcat ccgcgtcacc aacggggcga gacggcttta tgtcgcgttg aacaacggag

47881 tcaaagctga ggtgctgggt aactgcggtc ccgccacaat cagcttggtt gacgggatga

47941 ttgttgtgga agagcagact ctccactaag ggcagcagca tgggcaaaaa agtacacatc

48001 atttgtggaa agtgcggcag tgacgagatg aactttgtca tcaatggaca ctgtccagac

48061 gatccccaga atgttgccag tatgtcctgc tcaaattgtt gcgaactgac aggcatagcc

48121 gagtggtcgg aatttaatgg acgtgagctg aaaggtgaag ccgtcgccct atcaacaccg

48181 gcaaacgtga atgctcttct aggattgctt cgccaggcga tggacagcgt tgagtatcgc

48241 ctctatggcg ggggcatgtc attgagcggt aatgatgctg ccgaactcat agacctccag

48301 gagcgagctg agaaggtctt gtctgttctt cggagtgatc agcatgagtg atttatacga

48361 accgcttgag tttgtgttct gcggctttag gaaaggggac gctgggctct ttatctcggt

48421 agccactttg cgcgatggtg ttttaggccg tgagatgtat ttctcaaagg gaaaaagcaa

48481 aaggcgctgg gttgtcggtg gcatttactc aggggcctcg ttttcggaca acggagcaaa

48541 aggtctggat gatgctcatt acgttaaagc atgggaagtg caaggcgaca agatagagtg

48601 gcaagcaaaa agcgagcaag ccgaagcctt ggcgcgaagc gagaaacttg aagccgacga

48661 taggaagcgc aatgaactcg aagagctgat gctacccatc aggaagcaat atggggcatt

48721 aacaaagcga cgtgataggg caggggccgc agctcttgaa gaagctgtcc tgagagcgct

48781 gagagcgccc atcaggaagg ctgaggagaa ataaccgtgg agccagtctt agctaaagca

48841 ttcgccggtt ttaaagtcgt agctgtaagt gcccatcaga ttggcttttc ggcttttttt

48901 gccattggtg tttgctggcg ctttaggggt cgcaagtacg ccatagaaca ggataccaac

48961 aagtgccgct tgccacgcga aaacagcaac cagaatccaa aaagcagcag ccagaaccgg

49021 cagtttgtgc agaaggttga gcaccggctt ggcccaaatc actgccggga tagccagcac

49081 tgcgtaaccc cagttggttg cctcaagcat agccaggcca agaacaacaa cagccatcag

49141 cgcgatattc agtacatgtt tcatggtcat ctctcctttt caacttacac ttttatccta

49201 tcacagcatt aaaaaatgca actgtgaaag ggcgtttttc attcaaaaag aggtgacact

49261 ttgagtgcaa ttatcagcaa gtgcggcatg tacagatacc gtctggaaag ggatgtgcaa

49321 cccgaaggcc ttgtgttcgg ttattttggt gtaaacggct ctacggccac ggccaccgag

49381 gacgatcaca cggtcagaaa gtggataggg tttaccaagg tcaacggagg gaggcgattc

49441 atagtcggta atgcttttgc ttttcgggcg accgacgttc gagagctggc tacggcggtt

49501 gatcctgtcg ggcctgaaaa tgaaatccac ttggaaagga ttattcggga tgctgatgtc

49561 ttggtgccct gctgggggag ccgaactaag ctgcctaagt ctttgcatgt tcatttggac

49621 aggcttttag agcagctcgt tgcatctggg aagccggtat tggcatttgg cgtgactggt

49681 tcgggggacc cgaagcaccc actaatgctt ggatattcga caaaacttgt gccctgggga

49741 ggaaaataat tatgtcgatg cttgaagcac gttacttcgt ggcaaagata agcgatgcac

49801 aggccgtgct ctgcgatgag gagctggcga cactggaaag gctgatccgg aaggttgatg

49861 atggccgcag agcaaatggc aagagctcgc ttacttgcgt tgttgtcgaa gaagattggc

49921 cgaactggca gcaaacggtt gactctgtac tttcacttgc tgacgggaaa gacaacgatt

49981 ggaccaatgc cacaccagaa caaatcaagg ccttttgggt cgatgactca gtttggaaaa

50041 ctctggatgg ccgggataaa tggattgaag atctcgacct gctggtggac ggttccccgg

50101 tggcttctga gtgggaacct tttggcttgg aatcagggca gtctgtcgtc atacgaggcg

50161 gatggataga agggaatgcc ttgagtgatg atggcgtacc gctagtgcag gcattcgtcg

50221 cttggaaaca gggccaagac aaccactaaa ggaaaattac ttcggaaggg cggctatggc

50281 cgctttcttt ttaatattta cagtatcaca taatgatact atgatgttgt taatttactt

50341 ggggaggcgc ttatgctcac tgctaaatgt atagggtgcg gatgcaccga tgatcacgct

50401 tgtgttgaaa acggtcaacc gtgtcactgg cttcgcgtga atagagtgga aggtatcggg

50461 gtctgctctt cgtgcccgga tgctctcaat caattcgcat cgaccagcca ggaacaagca

50521 actggacagg atgactaccg gaacagtcca gaatggaagg atttttcatc acgcattggt

50581 aatgcactat gcgggggccg gagcaaagca aagtaaggta aaaacggcta tgccgttttt

50641 tatttgtcta ttacggcatc acattataag cctgtaaatg ctgaatctgt aattgcccaa

50701 aatgggcgtg agcaggggcg gggtgtgagg tcgttctaag gcaaaatact ttgtactggc

50761 agaacaaaga aagaatgaac tttcacatgt gaaagttttt ggggtacggg atggaacaag

50821 caattcaaag ttacttggct gacgaccggc aatatcaaga caggattacc gcagctctca

50881 gccaggttga agagaaaggt gcagagtacg aagctctttg ccaacagcgt gctcagttgg

50941 ggatctggca aaaaattata acgttctggc agtttcgtcg agacatcgcc gttatacgtt

51001 cagcactaaa aggccataac agcgatttgc ggtatttgcg ccgtgggaga gaccagctca

51061 aagagggatt ggtttctcgg gccgtgaagc aagcgattga cggtagccag attctggaac

51121 gaatcaccca agctcaagat aggcttgacg ccgcatctcg actccacgag agcaacaagc

51181 gactggtgga catggggcaa aaagccttac gtgaaatcag tgaggcctct tccagtattt

51241 catcagcaca aacaatggag gttctcgacc ttgtgactga caacaagggc atttccgtca

51301 tgtcgtcgat gtcaaactcc tcggccagca gtgaaataga cgatgcgaag agagctgtga

51361 aagccttcgc aaatgcgctg ggggatcatc gtgacattgt gggctcactg caccactcaa

51421 tggcaactga gtttatcgat ctgggtatgg actttgccgg tttgaatgat ggtttcgact

51481 ttgggagcgt cttctcgctg ttcagcttgt cgtctgccag ttcctctctg gacaaagtag

51541 agtctcgcgt tgaatctctg atgccagatc taagacgagc cgcctccaat tcggcagcag

51601 agtatgcccg tgttaacgaa gagttctttg gcctaaaaca acaggcctgt tgccaagtgc

51661 atgagttgct ggtgacaaat ggtatagacg tgtcagtcaa gcgcgttgag tccgccgtga

51721 acagctacag agttggaagg tgatatttac agcatcataa aatgaactta tgatgtgagg

51781 tgatcaacaa gtcgaaattt atttaaagga taagcgatga gcgatattac acagcaaatg

51841 gacaaacttg agattccaat caagttgtcc tttccagtca tcaacgttag cacttttgag

51901 ctgggccgtg cagagagcgt tttctctgac attgcaaaga aagtcggcaa gcactttatt

51961 gtgatgccat tcaagaagct gcccgatcca ggcacgatga aggcaatggt ggacgagtcc

52021 aagaagtcct ccaaaaacgg tgttgtcgtg ttcgacactt tctttttcga ccgacaacgt

52081 gcaaacccgg aaacactccc agccctcaag tcatcactga cctatctgga gaatgagggg

52141 atcaactaca tcatcgctgg caaagacgtc ttcaatgaag agttcgttta tcacatcgat

52201 ctcccggcta tgagcaatca ggaaatcctg aaactgctcc agacctgtga agataacgtg

52261 aaagatggtg gagtcttcga gagcaacgaa cgtgctgtca tcgcaaacca cgccctgggc

52321 ttgtcacaca cccagatgaa gaacgtcttc acctattccg cttacttgaa attcaagggt

52381 gaagaatacc tgggcgagat ccgaaaagaa aaagctcaca tcttgcgtga tgtcggcctc

52441 gatgtgcttg aggccattga tattgggaat gtcggtgggc tcgaaaacct caaggagttt

52501 ctccagatac gtaaagccgg ttgggacaaa gaccttccgg taaaaggtgt ccttctggcc

52561 ggtgtacctg gtggtggtaa atcgctgacg gcaaaagccg ctgccggtgt acttggcact

52621 accttggttc gcctggatat gggccgtttc tatagtaagt atctcggtga aaccgagcgc

52681 cagttcaatc gtgcattgca gaccattgag cagatcgcac ccgttgttgt gttgattgac

52741 gagatggaga agttttttgg taatgccgat ggcgaacacg aagtatccaa gcgcctgctg

52801 ggctctttcc tctactggct tcaagagcgc aaggagaaga tcttcattgt ggcgacggcc

52861 aaccgggttc agtcgctgcc tcctgaattg atgcgagctg gccgctggga ccgagcattc

52921 ttcattgatc tgccaagtgt ggctgagcgc cagaagattt tcgagatcca cctcgccaag

52981 cagaaggcca acatcgccgc gttcgatatg cctacgctac tgcgtaccac cgagggatac

53041 accggggcag agattgaaca ggccgtcatt gacgcgatgt atctggcgaa cgctcaggac

53101 aaagagctca acaatgaagc gctggtggat gcggtcactc gcattacccc gaccagtgaa

53161 actcgccgag aagacatcaa ccagattcgc agtttgcggg atcaaggctt ctatccggcc

53221 aataacttcg atgttcaaga gcagaatggc tctggacgaa aactcgccat cgaggactaa

53281 gcgattaact ttgtcatgcc ggaacacggc atgacccccg aaacaactca ggagtgatga

53341 gatgagccat atcgtaaaag gcaaggtgca ggtcgcctac aaagacaaag agctgctgtt

53401 gaaggctctg gagggagttg gcgtcgttgt tgaaaacgaa aagctatacc gcgtgggcgc

53461 tggctacacc ttcgagaaat acccgattgt tctgatcgac cagaacaaca aggagcaccg

53521 gattggctac aaggaaaaaa acggtgtttg ggagcagtac caggaaaact acggctctta

53581 tggccgctgg acgcagcagg caagcagcaa ggtgcaggat cgctacattg ccttccacta

53641 cgagcagcag ttgaaagagg aaggtttcag cgtgacggtg aagcagcatc acgatggcac

53701 tttggaactg gaagctgagg aagctgtttg gtaatagcgc caaggcaatc agattagttg

53761 aacttaacga cgaggtgaca aagtgaaaaa ggtaaacatc aagatcaagg gcggcaagat

53821 tgctgccgat ttcacggggt ttcagggtaa gacctgtgaa gcgctggagc agcgtattcg

53881 cccggaagag ctcgaagtcg aagaaaaaga gctgaaaccc gagtaccact tcaatgctgg

53941 tcaaacccag cacgaaacgg aacagaacga atggtgatga gaaggacagg gcgcgtcccc

54001 cttcttttgc tggttggtct catggtcgca gctcctgcgg cctttgccag tgactttgtg

54061 actggcgtaa tggtggggca aatgctctca gatgactcag gcagcaagaa ggtagaggac

54121 actggcccgg agagcaaaac ggtcattgat taccatgacg gtcagccagt tgtgaccaaa

54181 gtggaggcct tcaaaagcaa taaatggcgc aaactggatg gccctcaggg cggctatttt

54241 gtttgcccag gtgaataccg tagctattcg ggaaggctgc gttgtcgcgt ccatgatgac

54301 gggctcaatg gctttatggg cggtatgact gatgctcagg agctaccgct ccaagaagct

54361 ctgaccaagt tggaaggtaa acccatctcc ctgcaaacca ttgaagtcca cgggaacaac

54421 ctggcagtca aataccagtt agcaccacca gcacaaacgc acgtcaaaac tcaggacctg

54481 ggggtggtta agcaagatgg tgaaatgcag aaggttgcca gccaaccacc agcgctatcc

54541 atagagactg caccaaagag tgaacctccc aagcagattg gtgctgcaca agacaccgac

54601 cgcaatacca gtgcgtttca aagtccgttc gaggaggtaa gtgactccat gatcggtcta

54661 atggatagct ctttcgtcaa agtcatagcg gggttgatgc tggtgtttgg ggttgctagt

54721 ggaattatga ggcagagccc atcaggaatt gtgatgggga taatgcctgc aattatgatc

54781 atgaccgcgc caacggttat tcgtaccatg ttcgatacag gcgcggccag taccaagcca

54841 gtggaagata gcggaagctc atttcctttc ttcttggtgg caatagtgcc ggtgctgatt

54901 ttctttgcct atagagcttt tatgaacaac cggagcgact ccgaaattga tgagttgtta

54961 agagaagccc gacgtgctga aagggctgaa cgaccaagca atgagcctcc ctcagtggat

55021 gaactccgtg agcgccagca acaaaaccca gaaagagaac cggtggttgt cagctcatca

55081 gctccggcac caaaggtgca aaaggaagag ccaatcgaag tacagccagg caagcgcaaa

55141 attattttgg attagtgggg taggttatga gcaaaaagcg catcgtaatc aaaaatggtg

55201 aggtctgcgg gtttgccgat gaggtttcct tcaaaggcct tgaagtgcag gaatacagta

55261 aaacaagggt ttcgcgcatc gtgccgacga gcggcattct aatgattgcg ttctatgtta

55321 ttcgcggact ttgttcagac gagtcaaaga ttgcggcatg gactcgtgtt tggcgttgcc

55381 agtggaaggt gctgatcgac ggtaaaagct atggaccatt cagcagtcgt gcggatgcta

55441 tctcgttcga gaaggacgag atctacaaac aaggcaaatt ctttgccgat gccactcacg

55501 aggcggcagt atgatgacac gggcggctat ggccgcgctg ttatccgcgt tggtaatagg

55561 ccttgtgtct gatttgtccg cacaagagct ggtggtaagc caggtggcta tagacaagtc

55621 cactgaggtg gagaaagaag ctaccttcca caaccgcaag tgggtgttaa ggacggggca

55681 ggttagtgga ttcttcatct gccaaggaga taacaaagac atttactacc ggcatgacag

55741 ggtgggcgct cactgccaga agacatcgac tgggtggcgc aatgtactgg gtctcaggga

55801 cgatgttccg gaagttgagc tgagcgtgta cctggggacc gttgaaggtg tccccgtgaa

55861 agtccttcat caggaagtgg atggttacga tctgaaagtg cagtacaagg ttgcacgtaa

55921 agggtcagca aatgagtgag agtgtgcgtc ggtcaacgcc gatggtaaag attaaaatcc

55981 tgccgagttt cgtaggggtt cccttcccgt cggtgctact tatcgttgcg gggctcctta

56041 atggcccttt gtggggcttt gccgcattcg tctttcatat cgtcatcaag cggtacatct

56101 acagagaata ccggaggttg ccatatccga tgccaaccgg ctcaaggcct atggatgagt

56161 tgagcgagct ccttgtaaag gggataccgg aggaattttt ccggcggctt gacgcagctt

56221 gcagctctga tgcgtcagta caaataacct gcccaaagaa ataccggctg ctcaaaatgt

56281 cgttgaatcg ctatgccaac cgcgctggtt gttacccgca gttccgggag ggggatgaaa

56341 tagcgattga ggtaacagag cagagaccaa gaaaagcagg agcagggaag ggctttccaa

56401 gtattcaagt tctgaaagcg tcagagggtg ttctgagtga accggagcag cctatcgaaa

56461 ttcagaaaga taagcgcaag attatcttgg attgaggtgg gttggaatgc ggtcattttt

56521 atacagaata ttgcttcttt gcttcgtcat tatcgtccag atagggctaa tcgccattga

56581 tgcgagcccg ttggttgttg ctatgttcct ctcgctaaca gctttagtcg ctggggtctg

56641 gatggcgtac cttcattccc gatttttgat ggcggtgact ccagtagtta tctctgtgct

56701 aatggcgacc ctcgccatga aaatgtttga gccgagactc tttgtttctg ccagcgaaaa

56761 aggctggttc cctttgtcat tgttcgtctc gataccgttt tacttctact tgagttactt

56821 gcttgagata gaggagaaga gacgtacatt ccgggccaat gtgattgggt ttttgcgtgg

56881 agtttcggaa gagatccgtc gttcaaccag agagcataga gatcaaggtg agataagaca

56941 cggtggaaca gagctgggga gtattgacga actacaaagg ggcgaggcca aagagccaga

57001 agtcatcagc tctacatcgg accagaaaga acaaggcact cccaagcgag acaagagaaa

57061 aatcattctg gactgaacat ggaacaaatc agaaaaggct taaccctcga atacgcaaaa

57121 gagaagcgtg agaagctctt ggcggagctc aaatcagatg aacactacag ccagactgaa

57181 acagtagcct atgggcacca cgacccgctc agcgttcctg tggcagcctg cgatagttgt

57241 catggccggg cgcaaatgca gaaggttata ggtcctcctg tgcgctggaa tatggtctgc

57301 ctgggttgtg ggaaggcgat ccaacaaatc cagaaacgac cgtggcaagc agcaatggcg

57361 tggaaccaaa ttaacttggg aacacaggac tacagacaac tgcccctctt tggacttggg

57421 agtctgtctc ctgaatcagc aagacagaga atggtgggaa tacgcaggaa cctggagctg

57481 agaaagagcc tcgccggtat cgagaggacg atagcccaca aggaaggtca acgcccacca

57541 gggaaagagt accagcaacg actggaagca tatctgcaat gggccatgct ggcgctgagg

57601 ttgctcaaag tcaaagcaag ttagtggtga gaaggaagag agaacccggt actgtaaagc

57661 gccgggtttt ttatgccgcc ctgtttacag tatcaaaaag tgatgctata aagtgagcaa

57721 gtcacaagga gaaccaacga atggctgtta tcaactcact ccgcgctctc aaaggcgtaa

57781 ccgcccccga agaactgaaa aaaggggatg gattcactat ctctcctcag cttctgctgg

57841 aggaagaagg gtttaacacc cgtggcgctt tctgcgagga ctactacgag cgcccggaca

57901 tcaaagctgg tattcgagtg ctggccgatg cttacaagcg tggcgactat gttccgccga

57961 tcatcgtcaa agttatcgac ggaaaggtgt atgtccgtga aggtcatcgc cgtcgccgcg

58021 ccatcctgct tgctatagag gaaggtgccg acatccagtt cgtgcaggtc gtagagcaca

58081 agggcgatga agccgaacag agccttctga tcgccaccag caacgatggg ctccctcttt

58141 ctccacttga gcgagccgtg atctacgctc ggcttgcaaa ctgggggtgg agcgaccaga

58201 tgattgccca acgtgttggc cgctcggctg agcacgttcg tatcgcccgt gcccttttgg

58261 agatgcctct ggaactgaaa cggatgattc aggaaggctc tgtggctgcc acctacgctc

58321 aggagctcta caacgagcac ggcaccaacg ctgttgagat cctgaaaaag gcgcaggaag

58381 aacaggccag tggcaatgac ggcaagaagg ccccgaaaaa actgaccaaa aagtctgtcg

58441 agaaaggtcc ccgcctgggc aaaaaggtgg ttgaagccat gcaccgaggc gtgagctcta

58501 ttaccagtcg tctggacaac atcaagccga acgatgacgg tgaaacgttc acccttaccc

58561 tgagccggga agatgttgat gcgtttcagg agctgaaagc caagctggcg gagctggagc

58621 ccaaaactga cgagtccaat gaagaccagc aagagctgga tttggcgggt aatcagtaat

58681 ggggcgctgt actgttgagg atgccatcca gttgctggaa gggggaacca ttgttgcggt

58741 cgtaccggcg tcatccggac cagatacgct ggtggtttcc tctatcaggc ttgaatccgg

58801 actgacagta cagttttccc ctccagcggc agccagtgtc agtgttcagc tcgcggagct

58861 cgatgatgaa cagtgacaag aaatcgtctg aactagccag actgaggcgt cagcatcagc

58921 tagtttgctc tgtcatcgca gccttgctca tctgtgcagg tatatatggg accgttgcac

58981 gtttgggctt catcctgatg gcgccattgt ttgttatcgc cttaggtgtg ttagtcgatg

59041 cgttcctgcc aagggtaagt tggttcttct gtgtgcggga cgtgagagac atatttgggt

59101 ggagaaaacg atagtggcga agttgaaggt ttatggcggg atcacttatg gggccgaggg

59161 gcagttcaga actgtcgttg cggccacaag taagagcaag gctgcttcaa ttctgaatat

59221 cacgatttac cagatgaata gctggtggac ggagactttt aacaagtacg aggtcgaggc

59281 ggcgatgtct gagccggggg ctattttttc aaagcctctc gatggcagag acccatttgt

59341 aaagcaggaa ggataattgt gagaagtgtt tttgtatggt tgaaggccgg ggcaatactg

59401 gctttcctat tcttcggtat tgctggtgct gtcaaccggg tctattgggt taacagccaa

59461 ggtttgagcc aggggtcgat gtcatccaca ttggttaaag aaaagtttgg cctagacctt

59521 ggtgcaatgt gccgttatga caaaccccag catgtcgagt tcaaagtcct tcctgatcag

59581 acaatccagt tccgctgctc atggtttgaa ggtggcggat taacctggtg gcctttctac

59641 accgagcatg atgtaaagag cgccgaggcg acctctgtgc tcaatgacat attccagggt

59701 acggaggggc atgatgagta gccagctaga actctttcat gtccaagagg cgtatgccaa

59761 ggccgataag ccgttatcga atgaggagct ctacgattcg gtggcagagc tcgctggaat

59821 cccgaaaagc gctctgaatg aacaaagtga aattggcaag gcaaaggtca agcggagcaa

59881 gctgaaaaga cagatacgct ggtatcagca aaccctcaag tcgatgaacc ttctccagaa

59941 ggtggacggc gagcggggtg tctgggagct ctcaagcaaa accaagaagg gcctgcatga

60001 agcgctaggt ggcgttcgac tggtcgctta ttcaacaaat cttgggctgg cggtgtggtc

60061 aaacaacaag agctttttct ctgacctcga tgagccggtg catctgtgtg tgacatctcc

60121 gccgttcccg ctccgtatac agcgtggcta tggaaacgtt gatgaagcca aatgggtgga

60181 tttcattact caggcactcg aaccgatagt caagaacctg gtgccaggag gaagcgttgt

60241 cctgaatgtc agcaatgaca tattcgaggc caagagcccc tccagatctc tctacgttga

60301 gcgaatggtg ctggcgctcc atgatcggct tgggctctct ctcatggata gatggccgtg

60361 gatcaacctg tcaaaaccac caagtccgac ccactgggct tgtgtgaacc gttaccagtt

60421 gtgtgccggg tgggagcctg tttattggtt caccaacgac cctgacaggg tgcgttctga

60481 caacagaagg gtcctgatcc ctcatacaga gaaacatcag aagctgatgg cccaaggcgg

60541 tgataacaga gtggtcagtt atggtgacgg ggcatatcga ctgagaggga acgcattctc

60601 taacgtcacg gaaggacgga taccgaaaaa cgttatccag cgcggccacc gttgtgcgga

60661 tactctggag cttcggagaa tcgctagaga actggggttg ccaccgcacc cggcaatgtt

60721 cccaacagac atacctgaaa tggctatccg ctttctgacg gaagagggcg accttgttgt

60781 tgatccgttc agtgggtcga ataagagcgg gttggccgca gaaaggaata accggcgctg

60841 gatcgcctgt gacatcattc ttgagtacat tcgcacccag gcggaaatgt tcaccggctt

60901 tgatggattt tggatgaacc cggccatagc agcagtgggc gggggagcgt tgaattaaga

60961 gaacaatgcc catgaattta agtattatcg caagagtctt gcgccaattg gcaattattt

61021 ttgtcctctc cgtgcttctt gtggccggat atatctacta tgccggtaaa caacatcagc

61081 aggcagcaat caacttttgg ggtgagcaat accagcctga tgccatctcg acgcaaattg

61141 attgggggtt tatcggtaat tgggttattc ctcgcggggg gccaattatt tctccgggca

61201 ttgctggagt gtgtcccaat acaccacttc ccgttgtgcc tcttaaaact ggtcctgatg

61261 gccggggtta tgttctctgt ggcattggaa gtgaggctgt cgctaccagc tttgatgtca

61321 atgacatcca ggatgaagag atacgaaaca cattaaaaac gatgtttgag gaagaatttg

61381 aaaaaactgt taagggggat aaatggacac tcaagaactg aaccacatga tagctgaggc

61441 ctacagccgg gatttgcaaa agcctgagct ggtatcgttc aaagaggtga gtcgctgggg

61501 gcgtaagtac ggtttccccg tcgtatgcac tctggccgat gaaagtgaag aaaagcagat

61561 tcactgggct gccagtttgc tcattcaagt agccggtact tggccgcgag aagatatgcc

61621 ggaattgctc acaccggaac ggggctccgc gctgttcaac gatgcgatgc agttattggc

61681 gaatgggctt ggagcagcaa atcaattgcg ctgacagcac aaaccttgtc aataagcggc

61741 cttcatggcc gtttttttgt ttaaagtttg ttttaacaga accataatta gatattgcag

61801 tatcatttta tgatcgctac aatctttcac atgtgaaagt ttgggggtgg tcttgatcac

61861 ctgcgagaaa ccaaaataga ggtgggtatg gcaaaagtaa tcagcttcgc caaccagaaa

61921 ggcggagtgg gtaagagtac cctctgtatc cagcaggcct tttatctggc gttacagaag

61981 aaaaagaaag tgctggtctt ggatatggat ggtcagggga acacgtcctc tcgactggcc

62041 cccagacgag agcttgagga tggtgactac gagcccatcc tcactggaac caaaaccgca

62101 gagctgttcg cttacgagct ggacggcatt gaggtcatgc actgcccttg cggtgcagac

62161 ctcattcata cgccgaagaa tgacccggat ctgtttgaga tggaggctgt gcctcttgac

62221 caagccatga atccggctcg ccatttggct gagctgtttg agaactacga ctacgtgctg

62281 attgattgtc cgcctagcct cggcagaaag ctggtggcag cgttggtgat gtctacccat

62341 gtggcatgtc cggtaaagct ctctggcttc gctgtggacg gcgtagaagg tctcctgaac

62401 acgattattg gtgtgcgcga ggcatacaac caaaatttgg agatcctggg catcgtgatc

62461 aacgacatgg accgctctgt caatcacgac aaagccctca agtcgctgga gaacacagtt

62521 ccggatctgc tgtttgagaa caaaattatg caccggcctc cgctcgatac ggcgacgact

62581 gatggcatcc ctgtctggga gcttcgctat ggacatgtcg cggccaaaga ggttgaggcg

62641 gtgttggaag aactattaga gaaggtgggc taagcgatgg cattgaacaa tttaaaaggc

62701 ctgtccgaac ttgctaaagc cgccaaaggc aagaaaggca aagaggttct taccgtacct

62761 gttgacgacg ttgtatccaa ggtccaggtt cgtaagcgct tccgtaacat tgaagagctg

62821 gcggcaacct tgctgaccga agggcagcaa tctccgatca tcgtgttccc gaagaatgaa

62881 gaaggcaagt tcgtcatcca aaaaggggag cggcgttgga gagcgtgtaa acacgctggt

62941 atcgagacca ttgacctggt ggtgaatgat aaggtccaga acaatttgga cgagactgct

63001 ggtgagctga tcgaaaacat ccagcgggat gacttgactc cggtagagat tgccgaggcg

63061 ttaaacctgt ttattgaaga aggttggaag caaaaggata ttgctgatcg gctcggtaag

63121 aatatcactt tcgtatctac gcatctgtcg ttgctcaagc tacctgactg tgtgcgtgag

63181 ttatacgata atgaagtatg ttctgataca gagaccttga ataaccttcg tctcttgttc

63241 gatctgaatg aagaaagatg tcgcgccgtc tgcgcggtgg ctatgtctga cgggattact

63301 cgtaaacaaa gccgtgagct gctgaatgat gccaaacgta tcaaagacga aatggaaaaa

63361 ggcccactga ctggctccca ccagaatgat gagcttggcg ctggcaacac cgacgagcaa

63421 tccctcaact cgggtgggga tggtacgtcg gaacaaaccg ggaatgacga cctgaacctt

63481 gcccaggagg aactggaagg gggtaaaaat tccaatggtc aggacgatga tgacgaagac

63541 cctttgcgtg atgaagaggg tgagcacaaa gatcctgtca agcagccaga taacagcggc

63601 aaggacaaag atgaagaagg cggtgatgct cttcctcctc tgccgaagga caaggaatgg

63661 aagaacgtcc gggctgacag tttgattttt gctgtcaacg ttaacctgga tggcgagacc

63721 aaacgtggag tcatcatgac cgaccgtgtt gctctggttc cgtctactgt ctgggttaaa

63781 acgctcgatg gcgaaggcaa ggaaaagcat gttcatgtgc ctgtgtcaga cattgaactc

63841 ctgagtgtcg aaggctaata aaggaggccc agccaaagag gttgggcctg tcaagaacag

63901 gtgaatatga agatctcaca ggacatgaaa agaaaatttg ctttggtgaa tgccctgtct

63961 aaaacggaga agccaagtct ccaagacctt cacaaggcaa caaatattcc tgaatcaacg

64021 atcaagagac agttgtctgc cctgcgtgat gagttcggaa tgaatatctt gttcgtcagg

64081 gagtctaccg gcgaacgagg tgccaccggc tactacatgc tgacagactg ggggatctta

64141 gacaggtctt cgttcttgaa ccggtacgga aaactgtaag gtgccataga gcagcgcatt

64201 gaatgcaaaa cccgccaatc ggcgggtttt tttatgctgc cacggtagcg gcggttgctg

64261 gaggtttttc ggcgtagtag gcgatgttct tggttcgtct gctaagggtg tctatgccga

64321 cgatactcag cgttttggga gtggtattgc gggactcgaa ataatggaat agctggatca

64381 tgcagcactt caccatcaac ggatcgatgt cttccaggta gattgaggct tctctcaatg

64441 cttcgggacc gtggttctcg atcaggtttt ccatccagtg aatggcgttg atgccagaac

64501 cacagcaagg ctcataaaag tctgccgaag actgtgagcc aacaatgagt gacatcaagc

64561 ggccaatctc tggaggagtg gggaagtaat tggtgccctt cttgtggaag ccagacatgc

64621 ttaggacgta acccaaaaca tcgctggtgg ggtcgcgctt aatggcggca gacaggacgt

64681 gagagagctc gaaggcgacc ggctgaagtt cttccgggat ctcctccttg gctgggtata

64741 ggccagtctg caagtatgcc cactggtcaa taaaggcctc aacaaaccga gatgtgccca

64801 ttctgtatcg ggactgctcg atgagagata aagccttgga tgtaagttgc tgtgtttctg

64861 gtgacataag cgctccagtt tgttctgacg aaacattcaa gaggtagaaa cgaagaaccc

64921 cgccgaagcg aggttctttg ctttcctgcc tgatcgcctt aatgccgaag caaagacgtc

64981 ttgataaagt cggggcaacc ggacatttcc cggtcgatga agtcctgacg tttcgccagc

65041 ggccatttca tgaacgccgc gagcgggatg cgcttattcg tgttagtatt gccttgcagc

65101 ataacaaagc tccttaattg cttgattgtg ttgtaagggc tgcaacgaag gtcgaagagc

65161 gatgcggccc ttatttatga ttattacagc atcataaaat aatatcaaga gacaataaaa

65221 cgataccaag aaacaaaact ttcacatgtg aaagtctccc cgctgtccac ctcgcacact

65281 ccaaacgccc aaacgcccaa ataacttgtc tatctgccca aaatgggcgc gtaagcgtcc

65341 gatagaaagt aattgttagt gagatattga ctacacggaa tatggctata tcatagaatc

65401 ataaaatgat gctgtaaaag tgatatgtat gccaaagcaa gcaaatcatc tccgtttgaa

65461 gaaaccttgc gccaactgtc cattccggaa ggagggcgct atcgagctgg cccctgggcg

65521 attagaaggc atcatcaacg acatcgttga aaacgacatg acgacgtttc attgccacaa

65581 gaccgtgcac tcaaagtcag gtggtgaatg ggatgaagag ggtaactatg caccttcggg

65641 acaagagtcg atgtgtgccg gtgctgcggc ttacttgatg aaaataggca ggccgacagt

65701 ggctatgcga attgcctttg cgtttggtga tgcaaaggtg tccgactggg acgaagctca

65761 agagctggtt gtcgagcctt tggtacaagg ggaccgaaat gagtaagcga tatgcggtag

65821 tgccgcatcc gaaactgaaa cgagagtaca aaggtcggct ggtcagaact actcgggtac

65881 taaaaaacgg ctgggggttg atccctctag gggctgtggc aacggtcacg catcagtctc

65941 ccaagggatc agaactgacc tttgaaccat gcgactgctg cggcctgaaa gccattatca

66001 gtcatgtaag catggactcc attgaattta tcgaaccgat tactgaggaa gaagatggac

66061 gagaacaagc tcaacattga aacggtcgat gggcacaacg agctggtagt cagctttttg

66121 tccagaatgg taagcctgag tgacgaggaa aagcagacag tattgtcctg ccttccggac

66181 accggcaaac aaacaatcac ccagctctat gaggcgctgc gttctcaagg gcaccaggat

66241 cttgcagaga aagtcgaacc atacctccag cagggggtgt ttgggccgat cttcgataac

66301 gccaagtcca aggtgttcgt tcgggacgaa gcgccttttt tcctcatgga tgaaaatcct

66361 ttgaactggg atgacgctaa agcattcaat cgcctacgta tgagtacgac ctgtgttctt

66421 ggccgtggcg gctggactat cggtgagcgc tttgatgacc gctttgatac cgaggtgggc

66481 ggaacacagc ttatcgtcac tcagtcactc aacgaaaaag gtgagattga aggaggactt

66541 cctacctcga tgtcgctgaa tgactttgct gagttcccta aacagccaag gcctcctcaa

66601 atagtcgatt accaggaaga caagcgatac acgcttgagg aagcggaagc tatccctgag

66661 ctcgcaccag tggttcagcg tctgaaagag cgcatcgaag agtatgagga gagaagagcc

66721 catgattgac ctgacatgcc gcctttgtgc tggttccggt gtttatgact tttccccttc

66781 caaagaaacc tgcggtatgt gccaagggca tgggaaattt gttgacgcca aagcaatgct

66841 ggtggctgcc atcgagctgg caaagaagca acgcgagcct gtcattgaag tggtaatggg

66901 gttggtgctc cagggtgtgg atgttagagg gaaggagcct ggagagcggt tcaaggctgc

66961 tcaggccttt aacgccccag ggcacaacct attacgatac gggtgggatg cttgggagct

67021 ctatgccctc catgagggtg taagtccaga acttgcatcg cttggccgaa gtgtgatgcg

67081 cgagtggcat agccattcat ggggccggtt cagtggagag gtaggcttta acgccgccga

67141 gatcatgatc aaacaggcca aagacaaccc agaaaaagca gaagagcgct gggcgttcct

67201 gcttagtgaa gaatggatgg tggagtaatt gtcgtgacag aacaaaccaa taagaaattg

67261 atgctggatg agtctccaga tatgcccctg caattcaagt ggagaaagtt tggtggtgcg

67321 atttttactg ccacgcaaac aatggagcac gtcaaggaag gacgtaagct cgggccgact

67381 ccagcaggga tgctacctca agaagtgtgg ctgaaccgcc tgatcgctat ggaagaaacc

67441 ctgccacgag ggaagttttt tgacagattc cgccgccgcg acaaaaacga gcagtatgac

67501 ttggccgccg atcatctccg tcttgtcgga cagaaaaagc gataggggga tagatgaaag

67561 cccaggcata cccaccatca gtcattcgta aaggcgctgt gttgtacgca gccctttatt

67621 acatctctga tgatgataaa gcaaaggtcg aggttacaga gtggatagtt cgctctattc

67681 agaaacgacg caactcaacc agcgatcaac gctatgtcaa tcttgcccaa aagttagatg

67741 ggattacgtg ggggaaaagg tcacggaaga atggggactt tggttggttg ccatctatac

67801 cgagctggtg cttgaagcag ttccgggaag gtggcgaatt gccttttggc gtttatacga

67861 cgcgactggc cgctctcaag tttgcgaaag tcagcttgca ggaagaggtc caatattgcg

67921 aggccgagct gaaaaaggct cagacagaag aggatactca ggaactccaa gaggagctgg

67981 cggagaacca gagacttctg aaagctgctg gagcaatggt gaagcgcgag caaaacaaga

68041 agaaaagagg ttgaccaatg ttaccgatcg tctccccctc cgtagtaacc aaacaacttg

68101 cgttcaatcg agtaggcgat aaacgcaagg ttagggtttc gtccaacttt ttggatgtta

68161 tggggttcaa gccaggtatg ggcatcgccg ttgagccagg ggaggggatg ggcggttttt

68221 cggtgatccc agcgaccgat gaactacaga cacaccaggt ttatcagcgc cggtatcaac

68281 caaagagtcg ctccaacaac ccgctggaaa ccgttattga gttttctgga caagggctca

68341 tagataagtg cttccctcgc tatacagagc gtttccacgt cgaaatgcga aaaggtcgag

68401 tagtcttcac tcccgtcgca aacagagcct ttgccattgc tgatcggttc agaaaaacca

68461 gccctttccg tgcctttgtg gcattgactg gcggggtaga cattcatgtt atggaatcgc

68521 ttggctggaa ggctgagatt gttttggaac atcgtccagt tgaagccaga gacagagcat

68581 ccgggcggaa cctgagtgaa gtacatgcgc tcaatacgct ggtgaacagc tccccgcgta

68641 tcctgctgaa tgaagacatt catcacctgg agctggatcg ccttggagcc ttgctggcgg

68701 agtgcccacc aattggtttg gcccattact cgttggggtg tgatgaccac tcaaacgcca

68761 aaagtccaag ggacaaagag cgttctcttg aagatctctc caccatgctc gacatggttt

68821 acccggcact aaaacagatc gaggtcgtga accctgccgt cgtgctggtg gaaaacgtcc

68881 cgaacttcaa agcatccgga gccggggcga tgatgggaac gacactgcgg cggatggggt

68941 acttcctcac tgagatggtc ttgaacggtc tggatttcgg cgcgtaccag gggcgagaac

69001 gctattacat ggtggcgtcg gtcttccctg ggttcgttcc accgaaacct gagcagagag

69061 ctggtggacg attgtggccg gtgatcgaga agcacctcgg ggattgtgca gacgtcacag

69121 cgttgaagtc aattcaggcc agagagtcaa cttctcgcag gatgcctgcg ttcttgacga

69181 gagaaagcac cagttgcccg accatcctca agtctcagga tcgtggggta aaggatgcag

69241 tgtacatcca agacggtggc cgcatttaca agccatcggt cgatctggtt caggagttga

69301 tgtcgatacc tgatagcttt gatatttcgt ggatggcaaa agaacaagcg acagaaacac

69361 ttgggcagag tgtggattac agattgcatt cagcggtcat ggccgcagtt cgggatcacc

69421 tgaatgtgaa ttgcggtcgc cataccgtgg tgcagcacgg tatcagaagt aaggaaggta

69481 aataatggca gtcatttact acggcgaggg aacccatgac gccggtttcg tcgggttccg

69541 tgtcgcacga acagttgggg tggcggatga ttaccggcag gaatacttct ccttgagaga

69601 gtattcctac gcaacagctc accggctggc ctacagcttg gaccgaaagt gggaagctga

69661 ggcagaagag gtgaagcgtc agaataagac ttgtaagcgg cgacgcaact ccgggccaaa

69721 tatcattgct gaggggctga gggcttatat cagtatcgag aaccggagcc ggatgggggt

69781 gaagagaacc tacttcgcac cttgttttct cgtcacaaag ccaggctacg gcaatgggga

69841 tattgttttc aggatttcca ctcatggcta cgcagaagcc tacgaaaagg cggtggaaaa

69901 atattgtgag atccatgatt tgacggatga gcagtatgtt gagttgcttg accgtatgcc

69961 gagtacagag gtgttcactg gatacctgct gaatgctctt ttaatacgcg gtcatcgcgc

70021 cacaaaagct gaaatactga gtaagctggg ggctgcgaag aatgaagatg acatcaccaa

70081 tagcaaaggg aaaagcggcc acaacagagt gcgttgccca gagtatcggt gggcgcaata

70141 acagcgccag atgttcaaac catgttttat agcaacacct ctttgggggc ggaattatgt

70201 tttttgataa caaagtagaa tctcactcgc tcgtaatggg agccagcggg aagggcaaat

70261 cggtcttgtc cgaacaagtg cggaaaaacg cgaggctgcg cggtgatctg ctggtggata

70321 ctgagatgta ccgtgaaggt cgagggttga aaccatacga gcatgagtac gctcgtcgct

70381 tagttctggg gctaagtgga ccgttgccgc gtgaactgcg tgggaagccg gtgacggtta

70441 tctctgatgt gtccagacca aaaaaggtaa agcgtcagcc aaagcaattt gttaagaccg

70501 taaatggcgt cacccttgaa cgccaattgg tggcagatgc aagagaccag ttggaaatgc

70561 aaacaggcgt ctggctcaaa caacctcagc tcatcgagct gatggaagag tcgggcatag

70621 acgaaactct agccgatttt ggtgaggctg aaacacagat ccgagaaatg ctggcggatg

70681 cgttggcgat gaaactggta gggcgctcat ggcccaagtg tggagccctc tataacgcgg

70741 cagaaaagtc cgacgttaac ttttcctccg agcttgatgc tgcggcaaaa gaagctggat

70801 acatggttcg ctgaatggga tttggtgtgg acaagataga tcggcaaagt tggctcgtta

70861 aattcaggcg agcgaagtgt caggacactt tagacacgat gcgggatgcg gccattcgca

70921 actatgaagg gaatattcgt gttatcgcgg atattgtatt ggcccatgag gcgagagaga

70981 cagaaattga aaaagggatg ttttgtctaa tagttagata gtctgatgag ctatcaggtg

71041 gtgattatga aaaagagaat acttcatctg cccgtaaaaa agatttactt cgatcagatc

71101 aaatctggag aaaaaccaga tgaatatcgc ctcgtcacag actactggat aaaaagacta

71161 gaggggcgcg agtatgatga agtccatgtt aagtgtggct acccaaaggc tggagatatg

71221 tccaggatag agattcgtcc ttggcgtggc ttctcaagga acgtcataac gcacccgcac

71281 tttggagatt atccggttga agtattcgcc atccatgtga attgattttg gtattctgat

71341 ttaaaactat tgtagctcag gtatcaaaag aacttgggta attaaagata aggggccgac

71401 ggccccttaa ttgttttagt ttgtgaatat tgaacaatcg aggtgggata gcgcttgttc

71461 gtagctacct ttgtggttcg tcttacgcag tgccaccagc tcaacaatca attggcttaa

71521 agccagtgat tgaggctccc agacgcgctt gcatttgtca gccatgttgg catttacgtc

71581 tgtaatgacg tagaggaaat cagggacgtc tttcaaggtc cccaggcctt ttgccatatc

71641 aacagcctca gggttttgat agcttcgagt ggcgctcagg tcatcgtggg aaatatagct

71701 ggcagatgtc tcagcgaaac ctaacaccat gccttttcct gcgctttttg caatggccga

71761 gccgatagct agagttggca tgatggcctg acctatttgg cgggtagatg cagttgccaa

71821 gcctgaggcg atgttcttcc cggcatcttc ggcagtggca gagctggttg tgagcatcgc

71881 agttaaaatg atttttccaa acatacttaa ttcctctatc aatccataaa gtccgacttt

71941 ttgaagggca tagtgccttt gcaatcggga tacgctgtgc agccccagaa cttagaggct

72001 cgtttcttgc caggggcttt tcctttcctg agacgcatcg ggctaccgca atctgggcag

72061 tctggacaat cctccgccgc gatacgtttc tcgggtttcc ctcggttgtc tgggaacgtc

72121 ttcttgcaag cctcgttctg gcagccccag aaaaaaccgt tcttcccttt gatccggtgc

72181 atctctccac cgcagttgaa gcattcgtgt gaaggaggct tcgcgccctc gaaggcttta

72241 gccatcgcgc caccttcttt ggtcagaacc ggtgcggcca ctttgagctg ctcaaccatt

72301 tggcagatcc aggtggaaat ctgcttcatg aagacagaca tgttcccgga gccggaggca

72361 actttctcaa gctcctgctc ccaagccgca gtcatgccgg gtgatttgat ggctggtgga

72421 agcactgcta taagcgcatg agccttatca gttgcaagca gtaccttttt ctgacgcttg

72481 aagtagcctt tatcaacagc gccctggatg atgcttgcgc gtgttgctgg agtgcctaaa

72541 ccggctgtgt ctttgaggat ttgtttgaac ttctcctcag tcacgaaccg ggcaatgttc

72601 tccatcgcgg caagcaatgt ggcttcggtg aagtgcggtg ctggccgcgt cattttgttt

72661 gccagctcgg ccccattcag caaggcaggt tcgccctggc taaccctggg gagcttctct

72721 tgttcaaccg gcgcgtcggt gtcttccccc tcgtccttgg gactgctttc actatcagaa

72781 gcaaacaaaa ccttccagcc ttgtttggta ggtgtcttac cagcggacgc aaagaggtga

72841 cgcccacact gcacctcgat ggaggttttg gtgaactcga actcgctgta gaactgcgcg

72901 atgtagaaac gacggatggc gtcgtaaaga ttgaactcga tctcagacat ggcactgatg

72961 tctgttctgg ccggtgttgg aatgatcgcg tggtgcgcgg tcactttggc gtcattgaat

73021 accctggcct tgcgatgagg atctgcgcca gccaccagcc cagagacgtt ctgatcggac

73081 aaaatgagcg cctggagaat gtccggaatg tcttcctttt ggctctcggg tagataacga

73141 ctgtcagtac gagggtaggt ggtcgctttg tgcgtctcgt acagagcttg agctgcatcc

73201 agtacctgct gagctgtgta tccccatcgt ttgctcgcgt attgttgcag cgacgttaga

73261 tcgaagggga ggggggctga ctctttacct ggtttggttt ctgctttgct gataacagca

73321 ttggcaccat tgacctggga ggctacctgc tcggcatagg ccttattgac gcaccggcct

73381 tgctcgtcac tgcactcttc tggtggtatc cattgggcgg caaactgtcc attctgtacg

73441 gacacgttca cacccagagt ccagtatggt gagggggtga aaccggcgat ctctcggtcc

73501 cgttgacaaa caagagcgac ggttggggtg ataaccctgc caacgtgaag agtgtggttg

73561 aagccgacat ctcgggccag cactgtgtag aggcggctca cgttcatgcc taccaaccag

73621 tcagcgcgtt gccgtgccaa tgcggcgtag tagagtgaga ccgtatcctt gccatccttt

73681 acgttgttca gcgctttctt gatgcttgac tcatcgagag ccgtcaaaca aactcggcga

73741 atagggccag agtagcggaa tcgatccaga agcgaacggg cgatagcctc accttcttgg

73801 tcgtagtccg ttgagatgta gatggtgctc gcctttttga ccagtccctc aacaattttg

73861 tactgtttga acgcactctt gcgaacgttg taccgccatg actctggtgc aatagggagg

73921 gtctccagtg accatgactt gtagcgttcg tcataatcgt ctggcatata caattccagc

73981 aggtggccga acgcccacgt aataacgcgg tttcctccat catggagaaa tccatcacct

74041 cgttgggagg ccttcattac gccagccaaa tcttttgcct gagaaggctt ctcgcagata

74101 taaaggtcca ttaagcagct cctctcatct ttgattccag attcgccagc tcagtccagc

74161 tttgagccag acctttgact tcggggtagt ctctggagag gttttcgagg actccagaaa

74221 tgacataggc cgcttcgctg gaattacccc tcgaaccggc ctcctggagc atgattaata

74281 aagcgtcgaa gagctcagcc ttacggccta gctctgtctg gtttcttatt tcagagcttt

74341 gcatgggttc accactgaat agggacgcac ggactcgcgc acaatgcggc gatctgcgtc

74401 agttacagcc tgactggtgc aaaagtgcgt ccaggcattc agataatctt tataggtgtg

74461 ggtaggtggt atgcagccgg ttacaacgtc cttggggatg tttgtggttc cctcggcttc

74521 cacaaagcgc caatcgtgct cgtccatacc tttgccacca tcgcaatagc gttcccagcg

74581 tgacaactcg tacagcgagt agcctttgcc tttattcatg gacttgaagg tattgatggc

74641 ttcgttgaca tccccgtgct gtttgatccc tgctggagtt ttatctgaac cagcggagtc

74701 atctttgacc ttgttcatcg ttgcgctgac attaccgtcg cccagcacat ggatataacc

74761 aggttgacgt aatgcagcac caccgcgccc catgttgtcc catgccatcg acagttcccg

74821 gctaactcgg gcaggtttgt cattttcggc aggggcagtc tggggctgag gcttttgctc

74881 gggggcgtta gaggcacacc cggccagaat tgccactccc agaattgcta tcagttgtct

74941 catcgtgtcg ttcctcataa aaaattgtcg atgagcccat tctaaaaggg gggggatgga

75001 aagctggttt ctcaaactgc ccaaattgga cagataggcg tgtgtgcgat ctaaaggtgt

75061 tttgagagca tgtagcgacg agaaatacga taggaatatg gtcaacctac atgctgaaag

75121 cccttaacaa gttatttggt gggcgaagtg gagtgatcga gaccgcgccg agcgtcagag

75181 tgttgccgct taaagacgtg gaagatgaag aaatccctcg atacccacct tttgccaagg

75241 gcctgccagt ggccccacta gacaagatac tggcaaccca agctgaactg attgagaaag

75301 tgcggaactc tctcggtttc actgtggacg acttcaaccg gcttgttttg ccggtgatcc

75361 agcggtatgc cgcgtttgtt cacctgttgc cagcttccga atcacaccac caccgtggcg

75421 ctggtggtct gttccgacat gggcttgaag tggccttctg ggcagctcag gcatctgagt

75481 cagttatctt ttccatcgag gggacgcctc gtgaacgccg tgacaatgag ccgcgttgga

75541 gactggcgag ctgtttctcc gggctgctgc atgatgtggg taaaccgctc tcggatgtgt

75601 ccattacgga caaagacggg tcaatcacat ggaacccgta ttcggagtca cttcatgact

75661 gggcacaccg tcacgaaatc gaccgttact ttatccggtg gcgcgacaag cgacacaaaa

75721 gacatgagca attctcgctg ctggcggtgg atcgaattat tccggctgag actcgggagt

75781 ttctgtccaa gtctggcccg tccatcatgg aagcgatgct ggaagctatc tcaggaacca

75841 gcgtcaatca gcctgtgacc aagctgatgc ttcgcgctga ccaagagagc gtctcacggg

75901 accttcgcca gagtcgtctc gatgtggacg agttctccta tggtgtgcct gtcgagcggt

75961 acgtgttcga tgccatccgc cgcctggtta aaaccggaaa atggaaggtc aatgagccag

76021 gcgcgaaagt ctggcacctc aaccaaggtg tattcattgc ctggaaacag cttggggacc

76081 tttatgactt gatcagccac gacaagatcc ccggtattcc acgagaccct gacacactgg

76141 ccgacattct catcgaacgt ggttttgctg taccaaacac ggtgcaggag aagggtgaac

76201 gtgcgtacta ccgctactgg gaagttttgc ctgagatgct ccaggaggcg gcgggatcgg

76261 tgaagatctt gatgctccga ctcgaatcaa acgacctggt gtttacgact gagcctcctg

76321 cggctgttgc tgcggaagtt gttggtgatg ttgaggacgc tgagattgag ttcgttgatc

76381 ctgaggaagt cgatgacgac caagaggaag atgtgtcagc tctgaacgat gacatgttgg

76441 ccgcagagca ggaagcagag aaagctctag ctggtcttgg ctttggtgat gcgatggaga

76501 tgctgaaaag cacctcagat gctgtcgagg agaaaccaga gcaaaaagat gctggatcaa

76561 cggaatcatc taagcctgac gctggcaaga agggtaagcc gcagagcaaa ccgggcaaag

76621 caaaaccgaa gagtgataca gagaaacaac cccacaaacc agaggcaaaa gaggatttgt

76681 cccctcagga cattgccaaa aacgcaccac ctttggcaaa cgacaatccg ttacaagcac

76741 tcaaggatgt tgggggtgga ctgggggaca tcgactttcc gtttgacgca ttcagcgcat

76801 cggcagagac agccagcact gacgcaacaa actcagaaat cccagatgtg gcaatgcccg

76861 gaaagcaaga gaagcagcca aaacaggact tcgttccaca agaacaaaac tccctgcagg

76921 gcgatgactt tccaatgttc ggtagttctg atgaaccgcc atcatgggcg attgagccgc

76981 tccctatgct gactgacgca ccagaacaaa caacaccagc gccagcaatg ccacctacgg

77041 acaaacctaa tctgcatgag aaagacgcaa agaccttact cgttgagatg ttagccgggt

77101 acggagaagc atcggcgttg cttgaacaag cgatcatgcc tgttttggaa ggtaaaacga

77161 cgctgggcga agtcctatgc ctgatgaagg gtcaagccgt cattttgtac ccggatggcg

77221 ctcggtcgct gggtgcgccg tcagaggttc tctcgaagct gtcccacgcc aacgcgattg

77281 ttccagaccc gattatgcca ggtcgcaaag ttcgtgattt cagcggagtg aaggcaattg

77341 tactggcgga gcagctttca gatgcagtcg tagcggccat taaggatgcc gaggcgtcaa

77401 tggggggata ccaggatgcc tttgagctcg tctctccccc tggcttggat gcaagcaaga

77461 ataagtctgc accgaaacaa caaagccgaa aaaaggcgca gcagcagaag cctgaggtta

77521 acgccggtaa accctcgcct gaacaaaagg cgaaaggtaa ggactcccag ccacagcaga

77581 aggagaagaa ggtcgatgtt acttctccgg ttgaagagcc gcagcgccag ccggtccaag

77641 aaaaacagaa cgtggctcgc cttcctaagc gggaggttca accagtggct cctgagccca

77701 aagttgagcg tgagaaggaa ttgggacacg tcgaggtgcg agaaagggaa gagccagagg

77761 ttagggagtt tgagccgcct aaggcgaaaa caaacccgaa agacatcaac gcggaagatt

77821 tcttgccgtc tggtgttacg cctcagaaag cactccagat gctcaaggac atgatccaaa

77881 aacgctcggg cagatggctc gtgacacctg tcctggaaga ggatggctgc ttagtaacca

77941 gtgacaaagc cttcgacatg attgccggtg aaaacatcgg catcagcaaa cacatcctct

78001 gcgggatgct gagccgggca cagagacggc ctttgctcaa gaaacgtcag ggaaaactgt

78061 atttagaggt aaatgaaaca tgacaatgag ttatgacccg ctcgcctacg agatgccgtg

78121 gcggcccaac tatgaaaaaa atgctgtagc aggctggctt gccgcctccg gcgcggcttt

78181 ggccgtagag caagtcagca cgatgcctcc ggagcccttc tattggatga cggggatctg

78241 tggcgtgatg gcgatggctc gtttgccaaa ggctatcaaa cttcacctgc tccaaaagca

78301 tttgaagggg cgtgatctgg agtttatttc tattgcggag ctccaaaagt acatcaagga

78361 cacgccggac gatatgtggc ttggtagtgg gttcctgtgg gaaaaccgcc atgcccagcg

78421 cgtgtttgag atcctgaaac gcgactggac ttccatcgta gggagagagt ccacggtcaa

78481 aaaggttgtc cggaagatac agggtaagaa aaaggagctg ccaatcggcc agccctggat

78541 tcacggggta gaacccaaag aagagaagct gatgcagcca ctcaagcaca ctgaggggca

78601 ttcgctgatc gttgggacca ccggctcggg caagacccgt atgttcgaca tcctgatttc

78661 acaggccatt ctgcgtgggg aagccgtgat catcatagac ccgaaagggg ataaggagat

78721 gcgggacaat gcacgacgtg cctgtgaagc tatggggcag ccggaaagat tcgtctcatt

78781 tcatccagca ttcccggaag agtcggtgcg tatcgaccct ctgcgtaact tcacccgcgt

78841 gactgaaatt gcaagtcgtt tggcagcgtt gatcccgtcc gaagcagggg ccgacccgtt

78901 caaatcattt ggatggcagg cactgaacaa catcgctcag ggcttggtca tcactcatga

78961 tcgtcccaac ctgacaaagc tccgccgatt ccttgaaggt ggcgctgctg gcttggtcat

79021 caaggccgtt caggcttact cagagcgagt tatgcccgac tgggaggcag aagcagcggc

79081 ttatttggag aaagtcaaaa acggttcgcg tgagaagatc gctttcgcgt tgatgaagtt

79141 ctactacgac atcatccaac ctgagcaccc aaactctgat ctggaaggct tgctgtcgat

79201 gttccagcac gaccaaaccc acttctccaa gatggtggcg aacctcctgc cgatcatgaa

79261 tatgctgacg tccggggagc taggccctct gctgtctcca gactcatctg atctgagcga

79321 cgaacgccag atcactgatt ccgcaaaaat catcaacaac gctcaagttg cttatctggg

79381 gctcgactcc ctgaccgaca acatggttgg tagtgctatg gggtccatct tcctgtcaga

79441 cctgacagcg gttgccggtg acagatacaa ctacggcgtc aacaacagac ccgtaaatat

79501 ctttgttgat gaggctgctg aggtgatcaa cgacccgttc atccagctcc tgaacaaagg

79561 tcgcggtgcg aaacttcgtc ttttcgttgc aactcagact tttgctgact tcgcagctcg

79621 actgggtagc aaagacaaag cgctccaagt gttggggaac atcaacaaca cgtttgctct

79681 gcgtatcgtc gatggtgaaa cacaggagta tatcgcagat aacttgccga agacccggct

79741 caagtacgtc atgcggactc aaggccagaa ctcggatggc aaggagccca ttatgcacgg

79801 aggcaaccaa ggcgagcgtt tgatggagga ggaagctgat ctgttcccag cccagttatt

79861 gggaatgctt ccgaacctgg aatacatagc caaaatttca ggcggaacaa tcgtaaaagg

79921 ccgtctgccc atattgaccc agtaagagca gagctatgtg tgacaagaag tatagaaatt

79981 acgaggtagc catcatggtc gatgtgaacc ctttcgacag ggttatgaat gaattgaaaa

80041 gtcgtggccg caagaacgct cacatcctga gcatcctcca attcgactgg cctgcatcgg

80101 aggccatcat cgagaagctg agctgctaca tcacagacgg gattaaggct aatcaggagc

80161 ctgtgattta cccgatcatt gaagaagctc tgcatcgcta cagccagctc gtgtttcatg

80221 agcagagaga gaaatatgaa gacccggcca gaattggggc atttctggaa accctgatca

80281 ccgaaacctg ccgggcgttg gaagtgcaaa ttgtcgatag tggcggtgat tcatggtctg

80341 tcgattcagg agagtcgttc tcactgtggc tttcttccca tccaggagaa ctatccatta

80401 acccgcagcc ccatgaggat gagacctctt tgcgtggctt gctgtatgag ctcatcacct

80461 gtgagagcgt gaaaactgtt ttaaggagaa ccgactatga agaagccgtg gttgctggtc

80521 gcatggctgc tggttattga gttgctggca atattgctgc tgatccctgg cgactggaca

80581 gacagagcca tcaaaaggga atccgagctg gtggaacaga gtcttggtgt cgaagcaaga

80641 gactggatac agaacaaagc atctacctgg ttcaggtcga gcgttattga ttcaggattc

80701 tatgagggga tgtaccaaac gctgatccca tcagaagagg agcgccagaa gtccaagggg

80761 atgcaggaca tgggcaaggg ctggtttgtg tgggtcaaag gccgcatgga agcctttgtc

80821 aacgtcattt accagttcta cacaaggttg gcactgttag ccgcgtgggc tccctatatg

80881 ctgatcctgt tcgtacctgc ggtatatgac gggatgatga catggcgaat taagcggacc

80941 aacttcgatt atgcgagtcc ggttctccat cgttacagcg ttcgcggaac gatgtacctg

81001 atggccggat tgttcatcgc gttcttcatc cccatagcgc tcgatccggt tgtcatcccg

81061 atgacaatga tgacgtgctg tgtcctggtt ggcctgacgt tcggcaacct ccagaaacgg

81121 gtatagggag ggaggatgag ctactccgtt ataaatcaag atggggtgca tctgtgcgac

81181 atcccattga acgtctacca ggtgatacgt cgccaatcct tgtctgcgtt gtggctttac

81241 tgggcgcaga gcttgaattt ggtgaaggtg ctcattgctt gcgtcggaaa gatgatcttt

81301 gtgatgccgg taatgtggtt ctgggtgctg gtggtgtgcc gcttggttga accagaacgt

81361 atctcagacg tgtcaggact tatctctccg gacgtgatcg ggtgggccgc tggtgcggcc

81421 atcaccctca ttatcttttc cgtatttaca cgcccagcgt tgtacggata ccacaacttt

81481 ttcaagcagc atatttgctc aagagtaaag caagtaacgc cggaactcag aacagtcact

81541 gggccactgt tcttttaccg aaacgaaatt ggaacgatat agagaggaac catgaaagta

81601 actaatagac tccaactgct ggcactgggg ctgtgtatag ccggtactgc catcgcggct

81661 cctgatgtgc tcaatgacga cgcaaagctc aagaaccttg agaaggtgtg tcccgactgt

81721 cagatggtgg caaaagatgt actcaatctg cgggttgaaa actgccagct caaagacact

81781 tccagtgcaa tgatgattgg caccatgcaa aatgacccta tgttttcgtt tatgctggct

81841 gtacacacag cggcaggctc ggaggcatac aaaacggtag ttggtgccgc tggcaaccat

81901 gttgactgtg aaaacccgtt gaactggatc aagctgaccc agcaagcgat taaaggtggc

81961 aaagtctaat gcgtaagaga gatttctttt ttggagaggt gtatgagggg agtggaggag

82021 ccactctacg actgagtgat atggaaccat tggcaagaaa agtgtcggca gagttcttca

82081 ctgcacaact gaaccgtata ctgaaagagc atgacggtca gttgacgctc agcgatggaa

82141 cgtcgtaccc cagcttttgg agcttcatcg acaaggttga tccagagcag gttggtttcg

82201 tggagatcta cgctcggcag gatgttaacg ataatgttga agcgacactg gcgtgtgaca

82261 tcgttttggt aaatggtgtg atcaccgtta aacctcactg gtgtgcttac aaagacatca

82321 gggctgacga agtgatttcc accttactgg tgcctttgca tttgaaggct ctccagggta

82381 aggcttacat tcgctgggat gatggtgaaa ccgaacctct gttacaaaac gacgactatc

82441 aggctgaact tgaaaatgtg tttagtgttt ccaagtaccc atcagccatg agctggggcg

82501 atacagcaga ccagaaggtt aagcagtaca agatggacct tgagtgcgcc acagatgttg

82561 gtcgtcgagg tgtctcatca gagcaagcat gggatgctta tcgagaactc cgttacaata

82621 gaacagtgtg aaacaagcgg ccatcggccg ctttgaactt tcacatgtga aagtttttcg

82681 cctcccgctc ttatcatctc ctcgaataaa cctccataaa gtgcccaaaa tgggccgtaa

82741 cagcactgtt cctagattga tcaattaaga gacgattact ccgtgatgac gacaagttat

82801 ttggagggtc tatgtccgca caagctcacg tcagagaaag cgaaagctca tctggttcat

82861 tcatctcctg gcagttcctt gtctggcagg tgatgttcgt tataggtgtt gttgcaggga

82921 tgaacctgga acacgcattc aattttctag gttaatggga ggaagtgtga accttaataa

82981 acaatttttc tggtttttga tgaacgcggt gctgatgtgc atcttcatcg ttccagtggc

83041 cgtcgcgttt tggctgtctg cttttgcggc aggttttgac tggagtcagt gggttaagtt

83101 ggctgctgat acggccaaca gagctgcaag tgacccggcc aaggctctgg gcacggttca

83161 gacctactgg ggcattctca gcttcttcct gctggcggcg tacagtctga tgttcaagtt

83221 taaagccaat gctaacaagg aagtgaaaac tctggaggtg gcccgaccag ccaatgaagt

83281 tagcgttgcg gcttcggaaa aacattcgga agcggttcct caaaatcaat aaccgatcca

83341 gtaaacgcaa aaaaggcggc ttaggccgcc tttttttatt tgtcgctgga agtgcccgaa

83401 aagggcgcga agccgtggca gaaaatcagt gaccctgaat gagaatacag gccactagga

83461 aaagaagagg gttgctatga agcctgtaaa gataccgcgc cgggtcgatg agcccccgca

83521 tctgctgttg tggagcgcag atgagttggc cccgatgctt ttggggctaa cgataggggt

83581 catcatcggt aaggctctga tctgctttct gggggggtta cttgtaacca acctttatcg

83641 ccgattcagg gataaccatc cggatggata cctgctccac atgatctact gggccgggtt

83701 catcatgacc aaggccaaat ctctcaagaa cccgtttgtc cggaggtatt tgccttgaac

83761 ctgaaaaagt atctcaagac ctgggaaggg acccaaacag aaaataagtg ggggcgaatc

83821 ttccagggtg gtcttattgc tatcgttttc ctgctggtgg tccaagtatt cagcaaggaa

83881 accatcgtca ctatccagcc tttcacgctc acggaagaag cctgggtgac gaaaagtaac

83941 gcctctcagt cctataaaga ggcttggggt ttcgcttttg ctcagctcct tggcaatgtg

84001 acgccaggaa ccgttgactt tgtgaaagaa cggatcaccc cgcttctctc cccgagcatc

84061 tatcaggacg tgattgatgc catcgaaatt caagctcaac agatcaagaa cgaccgcgta

84121 accatgcggt ttgagccgcg ttttgttgag tacgagccca agagcgacaa ggtgtttgtc

84181 tacggatatt cctacgtcaa aggggcttct tctaacgaag agcgtagcga acgctcctac

84241 gagttcgcca tcaagatttc aaactacgcg cccgtgctgg actacatcga cacctatgta

84301 ggaaagccac gcaccaaaac tgttttggag caactccagc gcaaagaaga aaaccggaga

84361 aagcatgaag aacaacgcta aactttcgct cctggcgctg tccttggcgc ttggaacttc

84421 aatggcctat gcctcggatg acattcctgt tgtcccggcc agtgttatga aaaaggatgt

84481 tcctgcccct gtgacgtcag gacaaagctc ccatgaggtt gtgggcagca tgaatgaaaa

84541 ccccttactg acgatgaagc cgggggttaa ccagatcatc ccgatagccg ttggtcatcc

84601 gaaccgaatc gtcacgcctt tcagcaatcc tgagatcgtt tcaacatctc tgaccggggc

84661 gacggataac ggccagtgtg gtgaggtctg catcaaagag aatgtggtct atgtcgccac

84721 ggataagcag tatccggtga ctatgttcat tactgaaaaa ggctcggaag cccaagctct

84781 cagcctgacg atggttcccc gtcgtattcc gcccagagaa gtctttctca agctcgatgg

84841 tggtgtaggt atcactggtg cttttgccaa taccaaggct gagacctggg aacagagcca

84901 gccttatgtc gaaaccatcc ggtcggtatt ccgcaagatt gctcttggtg aagtccctca

84961 aggttacacg ttgaaccgca tccctgctgg tgctgcggtg ccgagctgcg ctcatcctgg

85021 ggtaaaggtg gatttcagca aggggcaata catgatgggc caccacctta acgtgttcat

85081 cggtgtcgcc ctgaacgtct ctgatcagcc tattgagttc aaagaggcgt tgtgtgggag

85141 ctgggatgtg gctgcggtga ctacgtggcc gcttaacgtg cttgagcccg gccagaagac

85201 ggagatctat gtggcgaaga agcagaagcg tggtctcgca ccaacgtcta agcgtccatc

85261 gctgctggga ggtgcccaat gattaagcga ttttggacac agttagaccc caacaagaag

85321 cgttgggtgt ctatcgctgg cggcgtcttc gttctttttg cggtcgtgac aatgttctct

85381 ggtgaaccca agaaagaaga gaagcgcggt cgccaagaaa ccatcaagca cgttctcaca

85441 gacaaaaaca cccgtgagat cgggatagat tcgttgtctg ccgatgtgaa gatggtgtct

85501 cgtgaaaact ccgacctgaa aaaggagctg gaacgagtca agaaagagct ggaggaaacc

85561 aaaaccactg ccgggaaatc cagtgatgtt ggccgtgaga tgacccgcct tcgtcaagat

85621 ttggaccgcc tgacccagaa gaacatggaa ttggctaaga aagtcgaaac cggcgctgct

85681 ggtggaaaaa catcctcttc atcagaggat gccagagccg atgttaatgg tgcatctggt

85741 ggtgatggtc agttcatgga gaaaaagctc gactacaaag atcctgcatc cttttttcgg

85801 gacgcaccgc ttcctgactc gaagggtggg gctcctgcaa ctggcaaggg agacggtcgt

85861 gatgcaacta aaccaggcat ccaaatagtg agctactcgc agaaagcgcc agaagttgaa

85921 gagaaggaca acaaggatga tgagtctatc tacctacctt ctggctccat cctgacaggg

85981 gtgctcatca acggtatgga cgcaccaaca tctcaaggtg ctcgtcgaga tccgttccct

86041 tcgaccctca ggattcagaa agaggctatt ttgcctaacc gcttccgtgc ggatgttcgt

86101 gagtgcttcc tgattgtttc aggctatgga gatctcagtt cagagcgagc gtacctgcgt

86161 ggcgagacct tctcgtgcgt tcgggatgat gggggtgtca tagaagcgaa gctggattcc

86221 tatgcagtgg gtgaagacgg taaggccggt gtccgtggtc gcgtcgtatc gaagcagggg

86281 caaatcatcg ccaagagctt gatggcaggc ttccttggtg gcgtttccga agcctttgac

86341 gtcaatcctg tgccggtcgt tagcactaac cctggctcaa atacccagta ccagtctgtg

86401 ttctccgacc agatgttgca gggagcagca gtgaaggggg ccagtaaggc gctagatcgc

86461 atcgctcagt tctatatcga catggccgaa ggcatcttcc ctgttatcga ggtcgatgct

86521 ggccgtcagg tagacatcat cgtgaccaaa ggaaccaagc tacaaattcg ttccaccggg

86581 ggaaccaaga aatgaaaaat ttgaacattt tgaccagaaa gggcagttcc agaggcgagg

86641 ctcaaaagga acaggcagta agatccgcaa agatgttggg ggtgggtgca gcgctactta

86701 ttttgtcggg ctgttcgacg ttcaacatcg gcaaggatga gtatagctgt ccgggaatgc

86761 cgaatggtgt tcagtgtatg tcagcgcgag acgtttacgc cgcaaccaat gacggaaatg

86821 tcccgcgtcc aatgaaacca gaggaagtcg aggccaaagc ggaagcggat ggcgaaggtt

86881 cctcaaacgt ttcagcgaac tcatctagct ccggagaccc ggtgattgac aactatgtcg

86941 caccgcgtct tccggatcgc ccgattccaa ttcgtacacc agcacaggtt atgcggattt

87001 gggtagctcc ctgggaggac accaatggtg atctcatcgt gacagggtat gtctataccg

87061 aaatcgaacc gcgcaggtgg gtgattgggg atggcacacc gcaaagtgag ccagttttga

87121 gaccgctgca aacggtacaa cacgaaccga agtctgaaac aaccaaatag gagatgttct

87181 tgatgaacgc aaatcagttg gcgaatgcct caagtaaaaa caacgcactc ttcctcttcc

87241 tggggttgat ggtggtagcc ttcctgctcg tgccggatca ggcccacgct ggtactggtg

87301 gtacagcgtt tgacgacgta tgggtaactc tcaaggattg gacccaaggt actttgggtc

87361 gaatcgttgc gggtgcgatg atcctggtcg gtgttgttgg tggtatcgct cgccagagcc

87421 tcatggcttt cgctatgggt atcggtggcg gtatgggcct gtacaactcc ccgaccgtag

87481 tggaatccat catgtctgct actctggaac atgcagagaa ggtcatcccg gctgttgtgc

87541 aactcagcaa tggcctgggg gtgtaaaaga caccttgccg ttaatcaacg ggtgataggg

87601 gcagcctaaa agctgccctt attttttggc aaaaagtcca gtcataacaa attccaacta

87661 tttccctaga ttctacgtca gtacttcaaa aagcataatc aaagccttga taaatatgca

87721 ttccttcgaa attcagcttt cacccattgg gtgaaagaaa agtgctcaaa aatatgttaa

87781 attatcagct tttatgactc gatatatggt aaaataatag taagaaaagt agtaaaaagg

87841 ggttctaatt atgattaata aaattgattt caaagctaag aatctaacat caaatgcagg

87901 tctttttctg ctccttgaga atgcaaaaag caatgggatt tttgatttta ttgaaaatga

87961 cctcgtattt gataatgact caacaaataa aatcaagatg aatcatataa agaccatgct

88021 ctgcggtcac ttcattggca ttgataagtt agaacgtcta aagctacttc aaaatgatcc

88081 cctcgtcaac gagtttgata tttccgtaaa agaacctgaa acagtgtcac ggtttctagg

88141 aaacttcaac ttcaagacaa cccaaatgtt tagagacatt aattttaaag tctttaaaaa

88201 actgctcact aaaagtaaat tgacatccat tacgattgat attgatagta gtgtaattaa

88261 cgtagaaggt catcaagaag gtgcgtcaaa aggatataat cctaagaaac tgggaaaccg

88321 atgctacaat atccaatttg cattttgcga cgaattaaaa gcatatgtta ccggatttgt

88381 aagaagtggc aatacttaca ctgcaaacgg tgctgcggaa atgatcaaag aaattgttgc

88441 taacatcaaa tcagacgatt tagaaatttt atttcgaatg gatagtggct actttgatga

88501 aaaaattatc gaaacgatag aatctcttgg atgcaaatat ttaattaaag ccaaaagtta

88561 ttctacactc acctcacaag caacgaattc atcaattgta ttcgttaaag gagaagaagg

88621 tagagaaact acagaactgt atacaaaatt agttaaatgg gaaaaagaca gaagatttgt

88681 cgtatctcgc gtactgaaac cagaaaaaga aagagcacaa ttatcacttt tagaaggttc

88741 cgaatacgac tactttttct ttgtaacaaa tactaccttg ctttctgaaa aagtagttat

88801 atactatgaa aagcgtggta atgctgaaaa ctatatcaaa gaagccaaat acgacatggc

88861 ggtgggtcat ctcttgctaa agtcattttg ggcgaatgaa gccgtgtttc aaatgatgat

88921 gctttcatat aacctatttt tgttgttcaa gtttgattcc ttggactctt cagaatacag

88981 acagcaaata aagacctttc gtttgaagta tgtatttctt gcagcaaaaa taatcaaaac

89041 cgcaagatat gtaatcatga agttgtcgga aaactatccg tacaagggag tgtatgaaaa

89101 atgtctggta taataagaat atcatcaata aaattgagtg ttgctctgtg gataacttgc

89161 agagtttatt aagtatcatt gcagcaaaga tgaaatcaat gatttatcaa aaatgattga

89221 aaggtggttg taaataatgt tacaatgtgt gagaagcagt ctaaattctt cgtgaaatag

89281 tgatttttga agctaataaa aaacacacgt ggaatttagg aaaaacttat atctgctgct

89341 aaatttaacc gtttgtcaac acggtgcaaa tcaaacacac tgattgcgtc tgacgggccc

89401 ggacaccttt ttgcttttaa ttacggaact gatttcatga tgaaaaaatc gttatgctgc

89461 gctctgctgc tgacagcctc tttctccaca tttgctgccg caaaaacaga acaacagatt

89521 gccgatatcg ttaatcgcac catcaccccg ttgatgcagg agcaggctat tccgggtatg

89581 gccgttgccg ttatctacca gggaaaaccc tattatttca cctggggtaa agccgatatc

89641 gccaataacc acccagtcac gcagcaaacg ctgtttgagc taggatcggt tagtaagacg

89701 tttaacggcg tgttgggcgg cgatgctatc gcccgcggcg aaattaagct cagcgatccg

89761 gtcacgaaat actggccaga actgacaggc aaacagtggc agggtatccg cctgctgcac

89821 ttagccacct atacggcagg cggcctaccg ctgcagatcc ccgatgacgt tagggataaa

89881 gccgcattac tgcattttta tcaaaactgg cagccgcaat ggactccggg cgctaagcga

89941 ctttacgcta actccagcat tggtctgttt ggcgcgctgg cggtgaaacc ctcaggaatg

90001 agttacgaag aggcaatgac cagacgcgtc ctgcaaccat taaaactggc gcatacctgg

90061 attacggttc cgcagaacga acaaaaagat tatgccttgg gctatcgcga agggaagccc

90121 gtacacgttt ctccgggaca acttgacgcc gaagcctatg gcgtgaaatc cagcgttatt

90181 gatatggccc gctgggttca ggccaacatg gatgccagcc acgttcagga gaaaacgctc

90241 cagcagggca ttgcgcttgc gcagtctcgc tactggcgta ttggcgatat gtaccaggga

90301 ttaggctggg agatgctgaa ctggccgctg aaagctgatt cgatcatcaa cggcagcgac

90361 agcaaagtgg cattggcagc gcttcccgcc gttgaggtaa acccgcccgc ccccgcagtg

90421 aaagcctcat gggtgcataa aacgggctcc actggtggat ttggcagcta cgtagccttc

90481 gttccagaaa aaaaccttgg catcgtgatg ctggcaaaca aaagctatcc taaccctgtc

90541 cgtgtcgagg cggcctggcg cattcttgaa aagctgcaat aactgacgat gaggcccagg

90601 atattgggcc tcctttcttt ctcttttttt cctgttgtca tttacactta acaaaaatac

90661 agcaaggaaa atcccatgcg cattttgccc gtcgttgctg cagttacggc tgcattcctg

90721 gttgtcgcgt gtagctcccc gacaccgccg aaaggcgtta ccgtggtaaa taactttgat

90781 gccaaacgct atctgggaac ctggtatgaa attgcgcgct tcgaccatcg tttcgagcgc

90841 ggattggata aagtgaccgc aacatacagc ttgcgcgacg acggcggcat caacgttatt

90901 aacaagggct ataaccctga cagggagatg tggcagaaaa cggaagggaa agcctatttc

90961 accggcgacc caagcagagc cgcgcttaag gtttcttttt tcggcccctt ctatggcggg

91021 tataacgtaa ttgcactcga ccgggaatat cgtcacgcgc tggtttgtgg tccggatcgc

91081 gactacctgt ggatcctttc acggacccct actatttcag atgaaatgaa acagcaaatg

91141 ttagccatcg cgacccggga agggtttgaa gtgaataaac tgatttgggt gaaacagcct

91201 ggcgcttagt gagtgctcag cttcagacca ataatgccag caacgatcag cccaaggctc

91261 agcaaacgtg ccgggctggc agactcaccc agcagcaaaa tccctgtaat ggccgcccca

91321 acagcgccaa taccggtcca gaccgcataa gcggttccta caggcaacgt gcgcattgcc

91381 caagagagca tggcgatact gacgatcatc gccgcaatag tgataatgct tggcgtaaga

91441 cgcgtaaaac cgtgggtgta tttcaggcca atcgcccaga caacttcgag caaacctgca

91501 attaataaaa cgatccagga catatcaggc tccagaacaa tggggccgtc cccggtgaaa

91561 gaagcgtttg caggtcgtcc tgcaaagcta atgtgtgaaa tggcattttt gcccggaaga

91621 aaatgaattt caaccttttt attcaccgcc tgctaaaagc aagaattaag cataattagc

91681 ggcgtagttc ccgcatttgg cactcatcgg agttctctat gatgaagggc tttctgaaac

91741 cagaaagtcg attgcccctc ttctgaatgc gaaacgttta tgcttaaaat tttagtgatt

91801 gaccggtgcc actttacccg cacagggata gaggccttgc ttaatcattc tggcaggttc

91861 agctcctcat ttctggtatc aggaatcaat aatctcctgt tagcaaaaga gcatattttg

91921 caatggaaac cgcatctggt gatcgcggat ttatacagtt ttattagcga gacgcactcc

91981 agtccgccaa ttaaaccttt ttttatgagt tgtggcgtca tcccactaat tttactgcaa

92041 tcagcagaca gacagcatgc acccatcgtg ccctctcaat ccgtagccca ttcggtcttg

92101 accaagcaca ccacgctgaa cacactctcc cacacaattc aggatttcct tatttgctca

92161 aaaagggcac ttccacagcc catgtttatg cactccctcc cgtaacctcc agcattgggt

92221 tcttgtaact caaaacgaat ggagaatgcg atgatcaatt tcaagcctaa aattccggcg

92281 atgcttgggg ctctggcggt tcttaccgct ggtgctgctc atgcagagct gctggaatac

92341 acctttaagg ctccagatgg tacgcaacgg tctctgactc cgaatgccaa ctatgccaac

92401 ccaacgggca atatctcgtt tgccttgagt gccggtatcg accgaaaggt aaagatttcg

92461 gtgcttcggt cggatggaac agtggtttcg acagcgacca gccaccttct tggggctact

92521 gatcgcatca cagtgggtgg aaaatcctac tatggtgcgg aacttcaact accagctcca

92581 gttggtgggg catacactat cagggcggaa attcttgcct ctgatggttc tacagtacag

92641 acggatgaat accctcttac tgtcgatgtg acgccgccaa cctactcttc gcttgccccg

92701 gtttatagta actatgggca ggttactagc ggtgatgttt ggaaactggg tctaggtggc

92761 tctgaggata atgccttttt gctgtcagga atctctgatg agtcacccat caaaggcgtc

92821 aaagcaaagc tgtatcgtca agacggttca ctctacaaag atgtatccgt gaactacgat

92881 gacgctaacg gacaagctag acagtctttc caatctggct ttttcccagc ctctgacttg

92941 gatgaagtgt ttacacttca gtttgagata agcgatagcg ctggaaatag ctatctttca

93001 cctcgtcaaa aagtgatgtt tgatagcata accaatgctc cgtctgcacc ttttggtgtt

93061 tatgaccctt caagtacaaa caacttaggc cctggattaa ccggatttgt tgcttacact

93121 gagggcatga cagttaaaac taacccaatc aagttagctt ggcgtgttcc gcgtgataac

93181 tggcatgagt atagagaagg tgggataaat atgaccaatg ccttgggaga gatgtccaag

93241 gtaggagaag atgccagcta tgtttatctc gtcacaaccg caccttatgg caataccgac

93301 ggcaattatt ggcgttgggt gaacttcggc cagtgggggg gaggcggtat tgcttataac

93361 ctcacactct ctccatcagc gcctaaaagt cccagacttt tgggtgtgga ctacaattac

93421 agtgatatag gatggtctag tttttaccgc tactgggtga acaattctgt tcttcctgtc

93481 actgttagta gtatccgtgt caaagttgaa ccaaggcctt acgttcaaac tgccgttcac

93541 aggggaagct gtgaaattcc tgttggtcaa gatagctgcg tgattgcaaa tagcttcaca

93601 atggctaaag gcacaacagg gtacgtccac gataatgcga cagttttcaa tccggataaa

93661 tctcttcgct cgaatcctct ttgggcagag gttaactgga acgatcagca ctaccctcag

93721 ctaagtcagc agttcgatca aaatagcaag gtatttacct tattcgttaa ccagccagga

93781 cgaggcgctt actttgatag gctgcgtctg cgaagtgcat ggattgaaga tagcaaaggc

93841 aacaagctgt ctccaactgg tgggttaata gccaacaact gggagaacta cacttaccag

93901 tgggatctta aaactctccc tgaggggcaa tacagtcttg ttgccgcagc agaggagatg

93961 cacgggcctt taacgcgaca accaatgttc cagataacgt ctgatagaac gccaccaacc

94021 atgacactca gtgtggcaga tggagcagct atccagactc tagatgatgt tgttattacc

94081 ttggctgatg ctatcgaccc ttcacctaaa ttgacctcta ttgcccttgt aggggggcct

94141 gccaatgaca aggtgcagtt gtcttggcgt gaggagtcga aaggtcgatt ccgccttgag

94201 tacccggtaa tgttcccttc tttgaaggaa ggggaatctt acacgctgac tgtttccggt

94261 gaggatgcac aaggcaatgc ggttcaaaag gctgttggct tcgagtacaa acctcgtcag

94321 gtaatgctgg cggatgggat ggatggcaag gtcatggtcc ccgctgtcac tcatgaattt

94381 gttcatgcag atggcaaacg gatcatcgaa accaagccgc tgacgctcag tgatggtgct

94441 gtcgtgacag gttcatacga cgtgtttgcg accctacgtt ctgatgcgaa agtaccgctg

94501 gtggttaatg gggtgcgtat cgagccaggc cagacaatgg ggatcatgag ccaacatgat

94561 ttcggtgcgt caggtggtcg tttgagcatt ccggttaaac ctgctgttcc tgatgtggtc

94621 ggctcttcca gccttcttgt catgacctcc gcgccgaact cacccatctt ggttgtggac

94681 atcaatacct ggaaagggac ggctaagctc tctgctgaat catggacaat tcgccaggtc

94741 attgacccag tgaaaatcta tgccctgcca gagtcgggtg tgccttgccg gttcaccacg

94801 aaagaggatg tggctatggc cgcagaccca attcgtgacc cggtgtgttt gctccagtgg

94861 gacagaactc cagatgaggc tgaacaaact acgcaggaca acaacgggat gaaagttgct

94921 gggctggtgg ggcaggctgt gagcattggt gaacaacctg tcgaatacag cctgtacctg

94981 ttcagtggtg acggttccaa ggtaaaagtt gggtctggct ctcagaacct gacggtaact

95041 actgcctatg gctctgttgg ctacaccccg attgatgaca ttgctcaggt gaatcgcgtc

95101 attgaagatt ttgatgtgaa cttcaagcag agcaaggggc cagactgttc tataaccctc

95161 agcgcagacc gtgcgaaaaa agaggccgcg aacaaggctg ttggtagtgc aagccgtgcc

95221 tgtctgttcg agtggcagca gatccccgat ggattggtcc aagacccgtt atcggaatca

95281 ccttcgctct ctggttcatt ggcctccaac ggcgttcatc cactggggtg gcgggtaagt

95341 attttcaccc gtaacggcac tagggtgacg ttgaacgacg agactttcaa tgtcgaagcg

95401 gttgacccac cagctccgac cgttgaacta gcctccgact acaacttcaa agacaacatc

95461 tacttggtgc cgatgacagg taactacctg ggtgatgcca ttatcaactc tgaacgagct

95521 gatctggata ttgccatatc gcgcaactcg gatgttcttg agtctgagac cttcactccg

95581 ggatggggtg ccaccaataa ggtgtatcgc cgcatcaata ccgatgagcg agcgttgtgg

95641 gaggagacca cctacaaggt gaacgcggcc tacaacaagg tgcctgatgt gaagactgag

95701 gttgtctacc gggctatctc tgcaccttct gacagtatcc gtcctatcgt tgaagttaaa

95761 ggtgataccg cgattgacac ccaggcattg ccggttaggg ttctcatccg tgatcagtac

95821 aaacctgatg gtgactatga cgctaacacg atgggggtgt ggaaagtacg cctgatccag

95881 caaaaggcct acaacgagac ggttgcgctc actgattatg cggaagcatc gaacggtgaa

95941 gctcagttct cagtagacct gtctggtgtg gatacttcct ccgtccgtat cgctgctgag

96001 gctgttctgg aaagccctgt tgagggttac aaccgcacag agttgtctat cagacctgcc

96061 ttcttgacag tgcttcgcgg tggtgccatc ggtgctggcg tggaggctcg caagttgtct

96121 ggtgaagctc cgttcactgc tgtgttcaag ctatctttgg acgaccgtca ggatctccgg

96181 gctaccggcc aggttgtgtg ggaaaccagt aaggacgacg gtaaaacctg ggagcagttc

96241 atcccagaag atcgatacaa gtatcagctt gtgaagacct tcgacaaggg ggagtaccag

96301 gttcgggcca aggtggtgaa cgtcaactca ggtgcggaaa agtacaccga agcggtcagt

96361 gttgtcgctt acgacaaacc tgatattgct gttatcggcc cgaccacgtt gtttgtcgga

96421 agtgaaggca agtacacagc gaacctgacg ttgaacgatg agccaatctc cggtggcaat

96481 gccattgttg agtggtctac tgacggtggc aaaacctacg cgcagacagg ggatagcatc

96541 acgctttcga gcgatgaaga aacccgatac cgcctgtggg ctcgtgtgcg ctctgccacg

96601 gcaccggctg atgacggcta tgcctatgaa gttgcgaaaa cggcggttga cttccgagca

96661 gtgaaggcac ctcgtcctta cgtgacaggg ccgcgagtta ttgagaccgg taagaagtat

96721 gtgttcaaag ccgaaaccag cctgccttac cgtgggatgg acgtgaagct gaacgggttc

96781 ttcacgctgc ctgatggctc gattgtgcag ggtgatactg ctgagtacga accctctgac

96841 actgacctca atcaggctac tgtagaaacg aagtacacca cctggatcga aggataccga

96901 gatcagggtg cagaggcttc gcatagccta cgctcccgcg tttggcagta tgtatggccg

96961 agcttcggaa tgcaggttag gaagaacgca gacgtggctc ctgcgacgat caccgcgtca

97021 gtgcggccaa ttgccttcaa cggcaagctg gaagagccga cctacgagtg ggagttgccg

97081 gaaggcgctg tgattcagga tcagcggcag gatattgtcc ggtcctttgt gatcaatgag

97141 ccgggtgatt acaacatcaa ggtcactgtc cgtgatgctc gcggccatga gaccgtgatc

97201 gagcaaccgc tcaagatcgg ccaggcagcg ccctatgcca ttgacctgca atactctggc

97261 tcgaacaaat acgagcgtga gcctctggat gtgctgttgc gaccgtacat ttctggcggc

97321 cacccacgcg accgtatttc gactcgtgtt tattcggtag atggtacacc gttggaaagc

97381 agtggttact acggcagagc aactctgggc gctggtgagc acagcatcaa gctgaaaata

97441 acctcagaaa tggggcatga agctgagggc gaggtgaaca tcaatgttgc agagaacaag

97501 ttgcctgcat gtagcctgag ctcacgagag accgttgggt cgtggatcgt ctatgcgaac

97561 tgcgaagata ccgatggccg catgaagtcc tacgaatgga ccattgccgg tgagttgcag

97621 agcatcagct ctgatcgagt gactatcagc aagggcacct atgaaacgat gccgaccatc

97681 tctctggttg gggtcgatga ctctgggggc aaatccgaag ctgttaccat gaactaaggt

97741 tcattcctct caaagcccgg ttcagaccgg gctttttttt gtatcgcgct ggaattgtcc

97801 aaattgggct attacagagg cgagtctatt tccatccccc ggatacactg cccagcatcc

97861 agtaatccaa tcttagggga tagacatgcg gacaaaatta cttggggcgc tgatggtgtt

97921 cgggattatt accggcacgg ctcatgcgtc atcgaaattg gaaatcaccg atcccagagc

97981 ggcgaagata gaggacattg tagagctacc catcaaaggg gttcgagccg tccaaagtga

98041 tgggcagatc atgttcctct ctgaaaacgg acgatttgtt atttcaggac aaatctacga

98101 cctgtggagc aagaagcccc tcaacacgat gtcccaaatg agggatgtag cggagcgtat

98161 ccacttcaag agcatgggca tggatgtgga cacgctgaac actgtgtcga tggggcgtgg

98221 tgacaaagag gtggtggtct ttgttgatcc taggtgcgcg gtttgccatc agctcatggg

98281 tgatgccaaa tcgctggtgg atgattacac ctttaaattt atcgtgattc cagctctggg

98341 tgctgagtcc aaccgcctgg caaaaaactt gtactgcgcg aaagacaaaa cccacgcgct

98401 tgatgcgctg atgaacaaca ccctgggttc ccttccttca aaagaaacct gcgaccccgg

98461 ccaatacgac caaacgctgc tgacagctca tttcattggg attgagggcg ttccgttcgt

98521 tgttgctccg gatggtcgtg tcagcaaagg acgtccgaag aacctgaaat catggttgga

98581 gagtgctgaa tgatcgtaac catcaaaaag aaactcgaag aaacgttgat cccggagcac

98641 ttgcgagctg ccgggattat tcctgtcctg gcctatgacg aagacgatca tgtcttcctt

98701 atggatgatc acagtgcagg ctttggtttc atgtgtgagc ccctgtgtgg tgccgatgaa

98761 aaagttcagg agcgaatgaa cggtttcctg aatcaggagt tcccgtcgaa gactacgctc

98821 cagtttgtcc tgttccgctc cccggacatc aatcaggaga tgtaccggat gatgggtttg

98881 cgtgatggct tccgtcacga gctgctgaca tctgttatca aggaacggat taacttcctc

98941 cagcaccaca cgacagatcg catatttgcc aagaccaaca aaggtatcta cgacaatggc

99001 ttgatccaag acctcaagct gttcgttacg tgcaaagtcc ccatcaagaa caataacccg

99061 actgaaagcg aactccagca gctcgcacag cttcgcacga aggtcgaatc atcgcttcaa

99121 accgttggtc tgcgtcctcg cacaatgacg gcggtgaact acatccggat catgagcacc

99181 atcctgaatt gggggccaga tgcttcatgg cgacatgact ctgtggattg ggagatggat

99241 aagcccatct gcgagcaaat cttcgattac ggcaccgacg tggaagtcag caagaatggc

99301 atcaggctgg gtgactacca cgcgaaagtc atgtcagcga aaaagctgcc tgacgttttc

99361 tactttggtg atgcgttgac ctatgccggg gatctcagcg gcggcaattc cagcatcaaa

99421 gaaaactaca tggtcgtgac caatgttttt ttccctgagg cagaaagcac gaaaaacact

99481 ctggagcgca aacgccagtt cactgtaaac caagcctacg ggccgatgct caaattcgtg

99541 ccggtgctgg cggacaagaa ggagagcttc gacactctct atgagtccat gaaagagggg

99601 gctaagccag tcaagatcac ctactcggtg gttttatttg ctccaaccaa agaacgtgtt

99661 gaagcggcgg cgatggccgc acgaaacatc tggcgtgaat ctcggttcga gctgatggag

99721 gacaagttcg ttgctctgcc gatgttcctc aactgcctgc cattctgtac cgaccgggat

99781 gcagtgcgag acctattccg ctacaagacc atgacaaccg agcaggctgc tgtggtcctg

99841 ccggtgtttg gggaatggaa ggggaccggg acctatcatg cagcgctgat ttcccgcaac

99901 ggccagctca tgagtctgtc tcttcacgac agtaatacca acaagaacct ggtgatcgca

99961 gccgaatccg gctcgggtaa atcgttcctt accaacgaac tgattttttc ctacttgtcc

100021 gagggggctc aggtctgggt tattgatgcc ggtaagtcct accagaagtt gtcggaaatg

100081 ctcaatggcg acttcgttca ctttgaagaa ggaacgcacg tctgcctcaa cccgtttgag

100141 ctcatacaga actacgagga cgaagaagac gcgatagtca gcctcgtttg tgcgatggct

100201 tcggctaagg gcttgctgga tgaatggcaa atctctgcgt tgaaacaggt cctttctcgc

100261 ctgtgggaag agaaaggtaa agagatgaag gttgacgaca tcgctgagcg ctgcctggaa

100321 gaagaaaatg accagcgcct caaggatatt ggtcagcagc tctacgcctt tacgtcgcag

100381 ggcagctacg ggaaatactt ctctcgcaag aacaacgtca gcttccagaa ccagttcact

100441 gtactggagc tcgatgaact gcaagggcgt aagcacttgc gtcaggttgt actactccag

100501 cttatttacc agatccagca agaagtattc ctgggtgaac gtaaccgcaa gaaagtcgtc

100561 atcgtggatg aggcctggga cctgctcaaa gagggcgagg tctcggtctt catggaacat

100621 gcctaccgca aattccgtaa gtacggtggc tccgttgtca ttgcaacgca gtccatcaac

100681 gacctctatg agaacgcagt gggccgcgcc atcgcggaga actcggccag catgtacttg

100741 ctcggccaaa ccgaagaaac cgtggaatct gttaaacgta gcggtcgtct gaccctttca

100801 gagggcgggt tccacaccct caaaacggta cacaccatcc aaggcgtgta ctcagaaatc

100861 tttatcaaat cgaagagcgg tatgggcgtc ggtcgcttga tagtgggcga cttccagaag

100921 ctactttatt cgaccgatcc ggtggacgtt aacgccatcg accagtttgt gaaacaaggc

100981 atgagcatac ctgaggcaat caaggccgta atgcgaagcc gtcggcaggc tgcataacca

101041 gggagacagt aatggacatt aaatcaatcg caatcgccgc cattctcggt gccgctggtg

101101 gcttcggcgg tagctactac gtgatgagcg aacaaacggc aagcatccat cagcgtttga

101161 atcaaacccc gccagtggtc gtggttgact tcgctaaagt ggcgtcggcg tatcccgctg

101221 gtgcctctca ggaggaagtt gaaaggctga tggtcaagac caatgacgca attttgaagt

101281 tgaaagacgc aggttatttg gtccttgacg caagtgctgt cgtcggggct ccaagtgacg

101341 tgtacctccc tgatgaggtg ctgaaatgaa ttttccactc aagaagtatt tcgtcaaaaa

101401 ggaatcctgg aagcgcttcg gggttaaggc aggtgtgaca ctagtggttc tttgggctgc

101461 tggtgcggcc tttgccagcc gctaccgtat tggcattgat ccacaacagg agaagtgcct

101521 gccgggttac accttcttcc tcattgatct gaacgaccaa actctggaga ggggagcggt

101581 ttacgccttc caagccaaga acatgcagcc tttctacaag gacgggactc gcatggtcaa

101641 aatcctcacc ggtatgccgg gggataaagt cgagatcaac gataagtgga agatcaccgt

101701 caatggtgat gtcgtcggag aggggctcca gctcgcaggg aaactacatc tgccagagag

101761 ccacttttac ggcaagacca cgctgaaaga gaacaactac tggtttatgg gcaaaagccc

101821 attcagcttc gactcacgtt actgggggac tgtgaaaaat gatcagatca ttggccgcgc

101881 atatcccctg ttctaagagc gttctggtgg cgttgatgtt ctctgtggct ggcggggcat

101941 acgctcaaga gtctccgctc acagagcagg ataaggcgct tattgagcaa ggaaagcaaa

102001 ttgcccaaaa ggcccagaag atggaaatgc catctctgtt gcaaaaccaa cacatggacg

102061 aggctcaggc cgaagccaag gcatttttca agcagctcca aactactaac ccaacgctca

102121 aggagatgca ccggaaacag gctgaaaagg gtatctactc tgaccatcgg atactggttt

102181 tcgcctcgtt gtctcttggc gaacaggggt tagatgacgt cctaacggcg gtgtcaggcc

102241 agcctgattc tgtaattgtg ttccgtggca tcccggaagg aatgaacttg gggcagggag

102301 ttaaagctat tcaggcgctc gcggccaaaa aagacccagt gccgaacatc atcatcaacc

102361 ctacgttgtt caaaacgtac aacatcacag ccgttcccac gattgtgatg ctggaggatg

102421 agccgctgcc tggcgaacaa ccaaacgtcg tcgcccaggt ctccgggttg tccgacccgg

102481 tatggttggc tcgggaagtg gataacggag aaaaaggcga tctcggcgtt aaggggccgg

102541 tggagaaaat cagtgagcca gaccttattg atgttgccaa gaaacgcctt gccaatatcg

102601 actgggaaga gaagaagaaa caggctatag agcgcttctg gaccaagcag aatttcaatg

102661 agctgccaag agcgccaaaa tctcgaacac gagaaattga ccctagcgtc atgatcacca

102721 gtgacatcag cactccggat ggcactgtgt tcgctcacgc gggtgacgtg atcaacccat

102781 tgtgcgatcc gaaggaagtt tgcaagcctg gaacgcggcc atttacccaa gcggtcgtag

102841 ttttcgaccc gctggacaaa aagcaaatgg aactactcgc caagaagctg cctgaaatca

102901 agctggagcc tggcgtacaa cggatcacct atatcgccac agagttcgac aaagacaaag

102961 gctgggattc ctacaagagt gtcaccgaca actttgacgc gccggtatat ctgctgacgc

103021 cagatctgat tacccggttc gagctggagc acacaccgag cgtcattact gccagaggca

103081 agaagtttgt tgtccgcgaa cttgctgagg agggcggtga atgatttttg ccccggcttt

103141 ccaaccaatt aaagacgtcg gcacaggttc gtttgtcgct gctgaggtac tggctcgttg

103201 gtacgacgaa ggccgggttc ttacaccatc ctctctgtca tctcctcctt attggggact

103261 ggtggatatg gagatggcac ggttcattca ggacaacctt cattattgct tggatctgta

103321 cccggctctc tttctgaatg tctccgaaca aactttgcaa tcagacgtca tcttcaaggc

103381 gtggtgtagg gttgtccgcg acattgctaa aaatcactca tcacggcttg tcatcgagat

103441 tacagagggc attcaggacg cctctctcgc atcgagatgg gaggctctga ccgaaatagg

103501 ggttgagctg gcgctggatg actatggaga caaaaactct tctctggatc gcctgagccg

103561 ttatgactgg cactattgca agtttgacgc gagaagactg cggtcgcttg aagactaccc

103621 ggccatcctc cactgccgcc gaaaaggcat acagctcatt gctgagcagg tggagagctt

103681 cccattgggg gagagcgcca aattacttgg actgtcatgg caacaaggtt tctatcacgg

103741 gaagcctgct gtcatggaga aaaatttgaa ttacgtaaag gccttaccat gatgcaaaaa

103801 attctacgga tcatggccgt tagcgcggtc ttttgggttg gttcggtatc ggctgaccct

103861 gggtgccaga atgcggaagt gatcggcgga aaactgatta ctgacatctg ctggagctgt

103921 attttcccta tcaaagtagc aggggttcct ataagtggtg gaggcggatc attcccgagt

103981 gaagccgtaa gcaaccctct gtgtatgtgc gaggataatc taggggtccc tcggcctgga

104041 gtcaccactt ctatgtggga gccagcacgg ctcgttgaat ttcagagagt gcctggctgc

104101 tcatcagtct tgaatggtgt caggttcccg tttgatagga ctaaccaagg gcatcatggc

104161 atgggagaca tggatggtgg tgatggttct tttatgcact atcactacta tgcgtttcct

104221 ctgttggtga tgctcgattt atttattaag cagacctgta atgctgatgg gtatatggat

104281 ctcgacatca tgtacatgtc ggagctcgac ccgacctgga acaatgatga gctcgctttt

104341 tttaccaatc cagaggcggc ggcagtagca aacccaattg cggcggctgc gtgtactgct

104401 gatgctgtct catcaaccgc tggaaaacct ttaaaacagc ttttctggtg tgctggttca

104461 tggggcaccc tgtatccatt tagtggcaac cagaatggtg gaaaaggtgt tatccgcgat

104521 agcagtcttc ttagcacaag ggttctggct gctttgcatc gccgggggtt agcgtggaag

104581 acaatgggtt ctgaggctat gtgtagaggc gttattagcc caacactacc caaaacgcag

104641 tacaaattca cgctattgca tccggttcca gagacaaact catctcacgt aattggcgaa

104701 tccactctta cgtggggtct ggcgcgaact ataccggcaa ttgggcaaga ccctatttac

104761 accatctggc gatggaatga ttgctgtaac aattgagcag accctaacga atttgcacgg

104821 gcattttaga ttatgcccca aaagggccac tacacgtaca ggtggccctt gtcttctcag

104881 atagcatggg aaggttgaaa tggagaacac aatgcgaagt cataattact ttatgaaagc

104941 tgtggcctcg ctgttgacag taaccatgtc tgcgctgccg atacattcct acgctaacgg

105001 tagccaagac caggacatca cagcggtggg taaagaggcc caggctttcg gacaaaacct

105061 ctcaaactca ttcaagtcga gctcggggac tgtgcaagat ggcacaatct ctatgccgac

105121 gttgaaagac ggtcaattcc aaatgaacgg ggggagtcag attaatgtca atgatctatt

105181 ccccggaacg agcgggacca acaataaacc tgatagttat tacttccctg atgccaataa

105241 acctgatgtg ggcggtctgc aaggcattta cgactctggc gatgacatgg acagcgtggg

105301 gaataatgca aaagggtcgc tgtggagtga tgccaatagt gctaatccat caatctctgg

105361 tgcggcatac aaggttcttc tcgatgcctc taatcgatca cgccctgatt tcagtaatga

105421 cccggtacta aatctaagca aaaagaccta tgaggatatg gacctcatcg caggtggctt

105481 tggggattgt tctgccgaaa caaccatcaa tcagaatact atcaacgccc acattccaga

105541 gtatgaacgg tgtcagcgtg ttgtagatca aagcgcggac tgtgaggttg tccatgacta

105601 cgacgcctct gtggtgaagc actatgatgg tccatataac ctcaaatctt gtggagaagg

105661 ctgtactgag ttgtggattg gtcgagtggg ggacaactac tggagtggta actgcaagat

105721 ttatgaggag tacacgcggg tccaagtcag caatccagat gccatagtgt ctgcaacgct

105781 tgaatacgcc aagtgggatg actacatgca agtgtgggtc ggtaagtccg gccaagagac

105841 taaagtgtgg tctggcccag atggcaactt ccctccagaa acagccggta gatgtgagct

105901 ctccacgagt tgggagcgaa accctaatgt tgatgtcact ccctatttta agaacgtgaa

105961 agatggtgat gttgttacgt ttaagatccg cgtttcagta agtggcgaag gtgagggttt

106021 tggccgcata aaactccgct atgacccatc gaaagccatt accaaggatg agtgggctcc

106081 acagagctgc atggattcag ccaaaggggt ggtagatggt tttgcggaag gcgagatcac

106141 atgtatagat gacccgactg atgctactgg ctgcacagtc atcaatggga tcaaagtttg

106201 cgaatctcaa ctcaagccgt cacctttgcc tggtattcca aaactatgca aaaaggttcg

106261 agttaaagct gactatgact tctataaggg gcaaatggac tgctggacag accctcaagg

106321 tgaaacgcac tgtccggtaa acacgggcgg gaatctcgat agctgtcaga aatatgaaga

106381 aaaccctcag tgcggcttca tcagttccaa atgtgttgat ggtgctcagg gaagttctgg

106441 tacgtgctac gtccacgagg atacttatga ctgtggtaca gatgtttctg ttccgacctt

106501 ggaaaaggaa actgagtacc agtgcggtgg gcctatacgc tgcatgggag atgactgcct

106561 tgatttgacc aaaacacaaa gcactgattt tgctcgcgct actgcgttgc tcaatgcagc

106621 ccaattcatg acgcaggata tgagctgcac aggccaagat ggggatgaca atcctaccgg

106681 ggatgaaaac gttatttgct ctgcttttgc aggggaagct ggcgaatgca agatagctgt

106741 tgggggagtt tctgattgct gtgaaaagcc aaccaatata tctcttgccg attatctgaa

106801 cctaataatg gccgttccaa agctcgatgg cgcagtgatg gggctgactg atggtaatgc

106861 gcttaaaggt gcttatcagg tacttaggga acctgccctt caagggtgga cagaagtcac

106921 aaaaccgttc acaagttata tagagaacgt ttcaggtgct gttgattcgt tcttccagcc

106981 tgtagagcag tttgttgatc aactcattga ccagctcaaa gagcaagtca aagaagtgat

107041 gatggatgtc atgaaatcag caggccaaga tgcagcaaca gagcaggcgg ctgccgcagc

107101 atctgaacaa gctgccgaag caatgatgga gactgcgaca acatggctta gcactgccat

107161 gacgatatat accgtctatg tcgttgcgat ggtgatgatc cagatgattt ataagtgcga

107221 ggaagaagag ttcactatga acgcaaaaag agcgctcaag aattgcacct atgtaggctc

107281 ttattgtaaa tctaaggtgt tgggcgcttg tattgaaaaa agagaagcgt attgctgctt

107341 caattctccg ctctctcgta ttatacaaga acaggttcgc cctcaattgg ggcagaactt

107401 tggagacccc aaaaatcctc agtgtgaagg gattccacta gataaaattg ccgaaattga

107461 ttggagcaaa attaatttgg atgagtggct tgggatatta cagcagaacg gtaaattccc

107521 tgatccggcc tcaataaatc tcgactcgct gactggagca gggaatgact tcaatattga

107581 cgggacgcgg aagaatgctc aggaaagggc gttggagcgg ttggaaggga ttgatattga

107641 cgcgaagcga aaagaggcga caaacagcat agaccctcaa acaggcgcgc caactggtgg

107701 tggataaaaa aaggggcctt tcggcccctt tctcatgtag attgattacg cttctgggta

107761 aacaaataac tctgcttttc cctgatcaag aaggtaaggg aaagcaactt tttcgccatt

107821 atccagctca accgattttg gagcaaggta gaggtaggcc caagctggta gctgtttgct

107881 aatagctgcg tagatcgctt gctgaggagc atggcttgct gccccattac cgacattcac

107941 aaagttgtat gcggtctcat ggcctgatgt gaaggcatac gctgtacctt gaacgccaga

108001 gacaaaatta ggagctgacc ctaggcgagg ctccacgatt ttcaccttaa cggaacacat

108061 gtcagctacc ttactttgtc cgttcaccca cgtcgggcaa ccccattcat tcttgtagaa

108121 ctgataggtt aggcgaccgt ctttgtcata cagaagcgtg aaattggcac ccaaatcagt

108181 cagcgctgac tcaatggaaa ctttcacatg tgaaagaaat ttggcctgag cgtctgccgg

108241 tgaaactgct tcgttagcag gcatccaagc gatcaagcta ttgcgagcgc catgctgttt

108301 ggggccgata gcccagttcg caaggttgac caagccacct tgccagtcgg tcattccgag

108361 ctgaggggac atgtagccag acagcacgta ggctgcccca aacgttttgg tgtcggtcag

108421 acgctccagc ttgtcacgcg gaacagaagc gtcttggatg cctgttacca ggccaccggc

108481 ctccgcgatg ttgtaagcgc gactgtgatt ggttttgtac gctgtgggtt cgtaactgct

108541 ggtggatgca cagccagata gagctgctac agccagagcc acggtggtga ttaaagcctt

108601 tttcatgttg ttctccgttc ttacctgagt tgaatctgat ttacttcgat aatatcacag

108661 catcaaaaaa tgcaaccata aaagacaaaa aaaagcccct cgggcgaggg gcaaaggttt

108721 gggctgggtt atgctgcttt tgttttctcg gcttcgtctg cgatttcctt gtcgccggtt

108781 gcagattccg gagcggcttc ggccacttca ccttctttgc ctttgctgtg ggcggaaatg

108841 cgagcccgtt tctcgatgcc gatgattcga ccggccagct tgatgaggcg ttgctgccat

108901 tggtaagtgg cgtcggtgcg ctgcttgctg gtcagtacgg tgttcagcca gagagtgtca

108961 acgatcccca tcagggtatc cagtttgcgg atcaggtgtg caaactgagc aacctgagga

109021 gagttgatct cgatggtgta ttcattcggg ttggtgtagc cgggcatcat gtcgatgccg

109081 ttgtcttcca tcagcttgtt gagctgggcg gtagctttgt ccaggtcttc ggagaccttg

109141 gaaatgtgtt ccaggatgac ggtttcaacc tggtcgattt cgtcctgctc accgatgatg

109201 cgaaggatca cgtcgatgga gaacagggag ttggaaactc gctcaaagct gcgttccatg

109261 acgcgctgag cttggaggct gtttactttc agaacttgct tgaaaacagg gcgagagtag

109321 tggttgctgc ggtcgttgtt tgtgtccagt gctacttgtg cttctgccat tagagatact

109381 cctaaatttg aggtacagat aaacgcactg gaaaggataa atgcggtcaa tctgtaacct

109441 gacctctcga agtgcccaaa atggacagtt acagcgtttt ctgtttagca aaggtgagct

109501 atatttgaat catcttagtc gctctgcggc tataggtgag atagctggtc tccgaaaaca

109561 aacagcatct agtcttatgc cgtatggcat tacttaaccc caagccgggg gcttctcccg

109621 gttttcgggt gttgcccctg ttttttttac ataggagcaa caccatgcac atcatcactt

109681 tcattgtagt tctgcttgca tctctgggcg cactgtacgt aggcgcgaag gcatttgctt

109741 tttccttcat cgttctcgct aaagtgttcc agcgtatcgg tttgttcctt tggaacaaat

109801 tcgcagttcc tgcaatgcgg tctgtacagg ccgtcatcaa agaacgtgct gccaaggctg

109861 aacaagccaa agcagtcgtt gaacctgagg taactcaagt tcaagaagtc gctaaacaag

109921 aacctgactg ggattatctc gaaatcccga cttaccttcg taagggcaag gaacttgttt

109981 ggtaataact gtcgctgagt aatcttcgac actttcaaac ccatcggggc aaccataccc

110041 cgacggggat gttgcccctt tttgcttagg agcaacatca tggaaatcgc tatcatcgct

110101 ttgtgtatcc ctgctggttg ggcaatctct cgtcctttcg tgagaatcgc tgaatcgttc

110161 ggttactgct gatagtcaac gcaaagggag ggggcaaccc ctctctttct ggtcaagcct

110221 ctgtggcttg tcctcaaagt cttctggtgt cagttggctt tgacgacaag ctacaggggg

110281 aatgtcgaat gcaacagaat cttcatgttt acgacgatca cgcaggcatc atctatctgg

110341 ctgatggacg cgaggtgaaa tttgatccga aactgtattc cagctcctac caggcgcaca

110401 gtgaagcagt gaagtgggcc aaagaaaccg gggtcattgg tcaaaacgac gatgtggtga

110461 tgttcgtcca ttgagggagt agcctccagg agaggctact ctcctcgctc cttaaaagtc

110521 ttcttactca tggtgaatac catcagtaag cagattttgt tctcttcgga gagcgaaaag

110581 tgcgatagct ggtcgccaaa aacaaacagc aaattaacgt taatttacta gcccaaccgg

110641 gcgcatccgc ccggttcggg acgtggtgcg cctgtcacaa taggagcgca cctatgtctc

110701 aatactctca attctcagta agcaaggttt tcggtatgcc cagcattccg gagaaggtaa

110761 cggccatcgg ctacgctgac ggttcaaacc ctttcatccc tgccactgat accaactacg

110821 ttttccgcaa agagtttctg cgagaggtct tggcctatct caaagagcct ggcggtgacg

110881 cattgttcgt aaccggccct accgggtctg gcaaaacctc aggtatcacc gagatcgctg

110941 gtcgtctcaa ctggcctgtt cagcaaatca ctgcccacgg gcggatggag ctgacagatc

111001 tgattggaca tcacgctctg gtcgcggaaa agcctggtca accacctgtc atgaagttca

111061 tgtatggacc tctggcagtc gctatgcgtg aaggtcatct gctcctcatc aacgaggtgg

111121 atttggccga ccctgccgag ctggctggtc tcaacgatgt ccttgaaggg cgtccccttg

111181 tgatcgctca gaatggcggg gaaatcatca agccgcaccc gatgtttcgt gtggtcgtta

111241 ctggtaactc tacggggtcc ggtgatgctt ctggtttgta ccagggggtg atgatgcaga

111301 acctcgcagc tatggatcgc tatcgtttca ccaaagtagg gtatgcggat gaggaagctg

111361 aactcagcat tcttggccgt gctactccga aacttccgga aaatgtgcgg aagggaatgg

111421 ttcggattgc taatcaggtc cgcaaactgt ttcttggcga aaacggtgaa gatggtcaga

111481 tcagcgtcac catgtccact cggacgttgg tgcgttgggc gaaactgtct ctagcgttcc

111541 gtggtgcccc aaatgctctt gaatacgcct tggatcaagc tctgcttatc cgtgcggcca

111601 aagaggagcg tgaagccatc ctgcgtgttg cgaaggatgt gttcggggac caatggcgct

111661 aagaggtgac gcatgaagaa aaatgactgt ttgtgccgtc gttacactgc taaagagtgg

111721 ggcaatgacg aaaccacaat agaagtcttc attggctaca agttgctccg ggagcccagc

111781 tcctcggagc caggccaatt cacgatggtc gagctacgcc gaaccgtcac tgatgggaaa

111841 gctgaaaatt ggtctgagac aaagctcgaa ggaccttttg aagctaacgg cccagacacg

111901 atcccaatgt cctacaagga caaagaaagc cagtatgtgt cacagtttct cagccagggg

111961 tacacctttc tggatgaggt gctggtaaac gcagagacac agacggtgct ggaaggggga

112021 atgtttcagc cggacaaaca gccagcttgg gatctctcaa ctggctgtta tctccgccct

112081 ctgagttacc tccaggtgac ataaacctct tcaaaggttt tgttgctggg gtttttgcca

112141 aaggagccgg tttaatcggc tttgaggttg ctcggagcga aggctcaaat gacttgctac

112201 ccagtgtgct gatgcgtact gacagcggtt atgagcttgg ggttagcact ggactaggcg

112261 aaaacactat ccatccagct acgctggaag gcgctggtga actccgcccg gaacatggtc

112321 acaaacccct gctgatgttg gtttacctgc aacagcgttt cgcggatgac ttttcaaacg

112381 tagagaagcc gctggtggca ttctgtgatg aacagggtga taccttcgac tacgagcgtt

112441 ttgattcact aaaacccctc attgaacggt ttggtttcag ctacgacgaa gtaagagcag

112501 atgcagaaag gcttggcctt gtatctgagc tgattcgcct ggcagagatc gacgccgaac

112561 aagaggatca ctttttttaa cccttacggg ggcttgctcc ccgaaagggg cgttggccct

112621 ctccaaataa cttggaggtc atgatgtcta aaggcgtcaa caaagtaatt ctggtcggta

112681 atctcggttc tgacccggaa attcgctaca tgccaagcgg cactgccgtt gccaacttca

112741 acgttgcaac aacggatacg tggcgcgata agcagtctgg cgagcaaaga gagcatactg

112801 agtggcaccg tattgtgctt aaaggtcgtt tggcggaagt cgctggtgag tacctgaaaa

112861 agggctccca agtctatctc gaagggagca accgcacccg gaagtggact gacagccaac

112921 aaatcgagcg ctacaccacc gaagtacact gcgttgaaat gcagatgctt ggtggtcgtg

112981 gaaatgcacc tcaggacaac tctcaacgtg cagcgcccca aaaagggcaa cgtacaggag

113041 ccggtacgca atctgctcct gtgcagcaat cagcaccgca aggtggtatg ggcggaggct

113101 atggtcccgc tcctgatggc tgggatgatg acatcccgtt catgcggctg caccacttgg

113161 ctggcgggta acaccgccaa ctcttactaa accccacggg ggcactcatg ccccgagggg

113221 gtcatggtgt cctcaaaccg ttcattcgtg aattggagaa acaccatgtc tgataacaaa

113281 tctcttgtaa cccgtatcgc aagccggttt ggcgtggaca cccgaaagtt ctatgaaact

113341 ttgaaggcga ccgcattcaa gcagcgagat ggaagtgccc cgaccgatga gcagatgatg

113401 acgctcctga tcgtggctga acagtacggt ttgaaccctt tcactcggga aatctatgcg

113461 tttcctgaca agcaaaatgg gatcattccg gtagtaggtg ttgatggttg gagccgcatc

113521 atcaacgagc atccccagta tgatggcgtc gagttcgtgt attcggacaa gatggtcaga

113581 atgcaggggg cgaaagttga ctgccctgag tggattgaat gcgtgattta ccgtaaggac

113641 agatctcgcc ctatccgcat caaggagttc attgatgagg tgtaccgtga accgtttcag

113701 ggtcaaggtc gcaatggtgc ttacactgtt gatggcccct ggcaaacgca caccaagcgt

113761 caactccggc acaagtcgct gatccagtgt tctcgtgtcg catttggttt ctctggtatt

113821 tatgaccagg atgaagctga acgcatccgt gaaatggagc aggcatcggc cattaacccg

113881 gctattgcca atctcccttc accatctcaa gttcaaagcc aagagccttt ggctattgag

113941 cacaaagagc ttgacccgat cctaaccaaa ctcgcaaatc gcgccattgc tgaaaacgca

114001 tggtctgcgg cgcatgagta tgtgaaggga cggtatgaag gttcggaact gcaatatgcg

114061 actcaattcc ttcgtgacaa ggagatggat caaatggagc ctccgaaacc tgactaccag

114121 gaagcgcacg agcaagagtc cgccgctggt ggttctgcaa atgctgaacc tggtgccgaa

114181 gaaatgccgc ctttgagtga cgaggacatg atccctgtta cagaagagga gggcgcggaa

114241 ggcagttact actaacccca acggggggga ctcccccgct gggggagatc tccctcctaa

114301 ccattggaga gaggtccatg aaaatagtca acctatcgca acgcgaggaa gattggcttg

114361 attggcggcg tcaaggtgta acagccactg acgccgctat cctgctcaat cggtctccgt

114421 acaaaacacg atggagactg tgggccgaga agactgggta tgcgcgtgaa gtcgatctga

114481 gtcttaatcc gctggttcgc cgggggatag aaaacgaaga tgctgcaaga cgcgctttcg

114541 aggagaagta tgatgacatg ctgctccccg cctgtgtcga atcggttcaa tacccgctca

114601 tgagggcctc cctggatggc ctgagagata acggggagcc cgtcgagctg aaaagcccga

114661 gtgcgactgt ctgggaagat gtttgtgctg agaaagcaaa cagcaaggca taccagcttt

114721 attacccgca ggtgcaacac cagctcctgg taacgggggc caagcaaggc tggttagtct

114781 tctactttga aggtcagatt caggagtttc caatactccg agacgaagcc atgattcaag

114841 aaatcttggc cgaggctaaa aagttctggc aacaggtagt agacaagaag gagcccgaca

114901 aagatccaga gagagacctg tacataccgc aaggtgaaga ggtcaaccgt tggattgctg

114961 ctgctgagga ataccgcctc tatgatgcag agattcagga gctgaaacag cgactgtctg

115021 agcttcaaga aaggcaaaag cctcatctcg acaccatgaa gtccctcatg ggggaatact

115081 tccatgccga ctactgcggt gtgatggtaa cgagatacaa agcggctggc cgggtagact

115141 acaaaaagct gttggctgat aaggcgtcag gcgtgaagcc tgaggatgtt gaccagtaca

115201 gagagaagtc atcagagcgg tgccgtgtaa cggttactgg ctctgtgaag ccacggtaca

115261 ttgttgatga ggacgtgctt gctcctcttg atgatttgcc ggaagaagta gagacgttct

115321 actggtgagt gggggatttt cccccgcttt cccttgggga ttctgggagt ctccaacgga

115381 aagcaagatt ttgttctctt cggagagcga aaagtgcgat agctggtcgc caaaaacaaa

115441 cagcaaatta acgttaattt actagcccaa ccgggcgcat ccgcccggtt cgggacgtgg

115501 tgcgccccca actttttgga gtgcatcatg actaaagtga accatctaca aagcctctgt

115561 gttatccacg tagactttga catctggagt ggacaaaccc gtttgtctgc atctgatctc

115621 aagctgggcg agggtggtga aattccacct gagaaagtag ctcaactggg aagtaagaag

115681 atctgtgatc cggctaagct gaaaggcttt catcgcctga aaacagaaac tcgtcgcctc

115741 ctgctgaaat tcggtatgcc gttcatgaac ggatttgccg tacccgtcag caagaccgat

115801 gaaatctgta acaagctgaa tgacatcaac tttcagttta accaactgaa acaggatttc

115861 atcaaaggtt acaacaaagc cgtggatgaa tggtgtcagg agaaccctga gtatgaacga

115921 gctatccgtg ccggagccct tccaaaggaa acggtcgagg agcggattgg ctttgagtac

115981 caggtgttca tgatccagcc tgtgaacgaa gatgaggcca acgccaaacg ccttaaccgc

116041 aaggttgagc gcctgggtga cgatctcatc tccgaagtgg ttcaggaagc gaataagttc

116101 tatatggaac gtttggccgg tcgagaccaa tgtgcggtca ctactcggca gacactccgt

116161 aacatccgcg acaaggtgga tgggcttagc ttcctgaaca gcgcttttaa ccctctggtc

116221 aagctgctcg accaaaccct ccggggatac gagcaacatg ccgatggccg aaacatcgtt

116281 gcgcctttct tttatcaggt cgtggccgca gtgctgatca tgagcgagag ggaccgcatc

116341 gagcagtatg ccaatggctc gattactgta gagggcatgg ctaatgacat tggcggttcg

116401 ggagcccaga tgggggaccg ttctaaagat gaaaaggccg aacagaaaag cgataaagcc

116461 ggtgagctta tccctgcaac agagggtggc gaaaccaagc agcaacaggt aggtggtact

116521 gaatctgttc aatcagaaca gactaacagc ggtggtaact ctgttgacct ggatgaagac

116581 atcgacaact tcttcaagag ttttgcagaa cgaggcgagg gtgaatcgga agatgaatcc

116641 aatgccggtg atgtggttcg agaagagcgt gttgatgttg aggatgagct ggttttgcct

116701 gaggaaactc cggtagagca agagcctgtc caagaggaga cgacagagga gcctctcaac

116761 caggagctgc ctaaaactga cgacgatggc gactatttct tctaatagtc acttcaacta

116821 aaccgacccg gaggggatag ccattccctc caggggtgga ctatctcctt ccctaccagg

116881 agaaaagata tgtccaaaaa acgcaccatt tacagcgctc taccaatcgt ggccgcagcc

116941 tatggtgaaa aactcggtgt caaagtcgcc atcggtaacg atgacgcata caccgatggt

117001 aagaccatcg tggttccgaa tatccccgac gactatcctc acatggatgc tgtctggggg

117061 tatttggccc atgaagcagc ccatgtccgg tttacggact ttggtgttga gcgccgcaga

117121 ggtcttcatg ctgagttgtc caacgttttg gaggactgcc gcatagaacg ggccatgatg

117181 gaactcttcc ccggtacgtc gcagaccctg aatgaggttg ctcgctatat ggctcaagct

117241 ggtcattacg agcacgtcac agacaaagag gcccctgcct ccattctgac agggttttgt

117301 ttgtactggt tgcaaaccaa ggctgtaggg caatccgtcc ttcaacccta tctcgattcg

117361 gctacccccg tattcgagcg cgtgttccct cagggtgttg ttgttcggct gaacgcttta

117421 ctgcgtaagg ctgtgaacac taagtcaacc gcagaggtga catccttggc cgaccaaatc

117481 atcaagatga tcgaggagga aaaggagaaa gaagagcaaa agccccagaa tggtcaggat

117541 ggtaacaacc agcagaatgc tggtggcaac caacctcaga acagtcaggg cggaagtggt

117601 aacgatcaaa accaagggcc tgatgccaat ggtggtgatc agcaaggtaa agaccagaag

117661 caagacgatg ctaacgggaa atctgatccg aaaggacagg gcgaccaagg caagtcggat

117721 actgatggtg gcagcaaggc aggacaaagc caggctggtg gcaattctga cgcggcgaaa

117781 caagacgcgg ccaaaatgct ccagcaggtt ctgaatgccg gtgccggtga tttgcgtggt

117841 gacgcgcatg atgcacttaa agccgagctc aaccgggtgg ctcaagataa gggggacagt

117901 agctatatga ctgttcgctc tgctgtgaac acccaggaca accctgctgt tggcaaaagc

117961 ctggtagggg atgtgaagag caccacttca aagataagaa cgcagctcta cggattggtc

118021 caggccagcc agcgagttgc tcaccgtaac caacgatcag ggaagcgtgt ggatgctcgg

118081 aaactacatc gtgtagtgac gggtgatacc cgcgtattcc tcaagccaga agccaagaaa

118141 cgccctaata cggcggttca catcctggtt gatatgagct cctcgatggc ctacaaggcc

118201 gccaatggaa aggagcgtca agacattgcg cgggaagcgt ccttggctat ttcgatggct

118261 ctggaagcaa tacccggcgt aaacccggca gtcacctttt ttggtggcaa ccggaaccag

118321 ccagtgttca gtgtcgtgaa gcatggagat acggttcaga atcgggccgg tcggtttggg

118381 ttcaaagcaa ctggcggtac gcctatggcg gaagctatgt ggtatgcagc ttttgaactc

118441 accaagaccc gtgaagagcg aaaaatgttg atcgtagtga ctgacgggca gcctcaaagc

118501 gccccggcat gtcgctcagt gattgacctc tgtgaacgaa gcgatgttga ggtgatcggc

118561 ataggggtag agactaccgc agtgtcagga ctgttccaaa agaacattgt cattgatgat

118621 gcggcagctc tgcaacgcac actgtttaag ttgatggagc ggtcattgac tgcttttgca

118681 gcttaacagg aagtgaaacg acaaacggct atcccttcgg gggtggccgt ttttatttgg

118741 agagtctatg aaccaattcc attcatcttt ggatttgtat catcggaaca aaggtagaag

118801 ggctacagtg ccggagacgc cttttttact gcttgctaag cgcattcctc cgatgtactg

118861 gagactgttc cagggtgtta ctttggatag tcgtatggga tacacaggca ggcggcagtt

118921 ccaccgtctt gggcaagcaa tcgactgggc aaagtcatca gttggcgatt cctggtcaaa

118981 taagcgcttt cacaagccgg taggcctcga tgtattgctg gcctgtactg cgagtaaggt

119041 gcctgaacat ctggtcgaag aactgaaaag acggggcagt tgatgcgtct ttgagctttg

119101 cacccgaaaa gggcagtttg agcgttcctg ttcacacagc tctcgtggaa caatcaaagt

119161 gaggagggaa taccctccca ctttctcaga ggccttcatt gagggttgct gagaaagtga

119221 attttgtcct ctctgagggc gaaacgtgcg atagctggtc gccaaaaaca aacagcattc

119281 aacctcagcc taagggcgca ctgcgcctga caggctctgt gtgcccaagt tactttaagg

119341 agcacacatg tctcatctga acaatctcaa atccgtaatg atctctctcg ccgccgaaca

119401 taagctgcct gaaatctacc aggatgacat cactaccgat gtggagtctc tggatcgatt

119461 cgatggttta cgtctggtct ggctgttgcg gtcttgcggc agcgtattgg tgccagcgga

119521 agttggcgtt aatccgatct atatcaccca ttggttgtgg tctaaccacg gtcaacaggt

119581 ggttccattc tctgtggata cccgcacggg gttgattgaa aaaatcgact tcgagcaagc

119641 tgaaaagctg atcatgcaga tgccttgcaa cctctcttca ttgcagaaca aggaatactt

119701 ggttgaccag gtaaaccgag tattgcaacg aggttgtgaa atgcgtatct ggggtagctg

119761 gcccaaaaca gcaataacct aacccctcgg gaggaaaccc ctcctgggtt ctctcccaca

119821 aacaaaggag agaagcatga gcctagacaa gttaagtcgg ctggtggacc aacaacgaaa

119881 gatccaggat gaaatcgaca atgaaatcat tctggccgtc aaagaggtgt tggctacaaa

119941 ctcagtcggt ttagcccgtg agcttgttgg gggtgtcccg caagatcatc cattccatcc

120001 gtttttactc gctatagcta agcagcttga cccgagataa ctaagtaacc caaccgggta

120061 caccaaggcc cggtcggctt tggtgtacct ccaaccaatt ttggaggcac tatgtctaat

120121 tcaaatttcg gctttctagc tctggccttg cgccaacgcc tgatcaaacg ctggtcactg

120181 atgcactctg ttcaaccgga gtctgtgttg gaacacagcg ctgttgttac cctgctttcc

120241 tacctggctg gcaatattgc cattcagcaa gggaagtcgg tggatctggc agtcatgctt

120301 gctcatgctt cgttgcatga tgcagccgag gttctctgtt cggatgttgt aacaccggtc

120361 aagaaagcaa acgctgtttt gcagcgtgag tttgaacgac tggaaaaggc cgcagaggac

120421 aaactcatcc agactcttcc cgaagaactc caggatgcag tcgccatagc cttcgctccc

120481 ggtggctatg aacaatcact ggttaaggcc tgtgatacct actccgctta catcaagtgc

120541 aaacttgagg tggccgcagg taatggcctt gagttccagg atgctctcag caaaatggaa

120601 cgtgttgttg ctcaggtgaa atcggacttc ccggaaatcg acgctctgga caaatggttt

120661 ggtaacggac tcggccattc agtagataaa ctgctggcag ggggtaacga tgactaatcc

120721 aatccctggc gacatcaaga ttaaggactt tggccgcgac cggaaattcc gttcggttga

120781 tgagcttcaa agcactttgt cagaacagta caaaggtcag catgtcagca tcgtttaccc

120841 tgcaaagccc agcggtttac tccgcacggt ttttgtcagt gtcgatgatg ctggtggcgt

120901 gaatcgaacc tatggggacc agtctcctgt cgatttttct gccatcaaag atgacctgta

120961 tgtaccaagt gacctgtagg agagcactag ctatggtggt gatcatcgta aacacaggac

121021 actatgagtt tatcggcctt ggtgagaccc acggacaagc cacagaaggg cttttgaaac

121081 gctgggacga acactgcgaa cgtaatccgg atgctgagtc tggttacatg caggagctga

121141 tagaagaagg aagcgcacaa gttgtcgaga tggagcccgg ctccgccgta atttacggac

121201 ttgacggcta agccttctca actaacccac tcgggcgcat catcccgaga gggtgtggtg

121261 cgtcctaacc aaaaggaatg cactatgtct aactttgaac aagctctgga gcgtactgac

121321 ggtaaaacac tgattctcag caatgggagt aagtgggcag gccaagaccc ggacagtatt

121381 cagactttac tggatgtcct gggtgacaac gttcttgacc cgatgtttga gcagtatcac

121441 tgttatcgcc catacccgtt tgaaccaatg gtgaggacgg ggcgaaatgg cgaaatgttc

121501 cagccttggc ttggggcagc ctgcttcttt gggaacttcc tgactgtctc tcatgttttc

121561 aacatcatca cgaaagatga cggtgttgtt gaggccttaa cagaggcgat ccggaagaat

121621 atggcgactg aacagtacca gcagaatgcc tatgagcgtt atgccggttg gttctacgct

121681 gaaaccagcg aagggttaag gctagtctct ccaagtgagg ctgccgacat ccgagctggc

121741 gcagtttcaa agctgcgtta cccaagaaac ttcgaggtta tgaaaactgc ggttctcaaa

121801 ggtcctcgat ttgatactga attaagccgc aaggcttctt aacccaaaca ttaaccccac

121861 gggggacatc ggtccctgag gggatggtgt ctccctctga aattaaggag ataccaatga

121921 ttaaacagca ttttcaaaac gaactggtta agtgtggata cccggatgat ctgacgattg

121981 agtacagtct cggatactgc cagggggatg gcgtagcctt ctacggggat ttgagcgttg

122041 atgacgtcaa agctctcatg aatcgcctat tcagcactga gcccggccaa gtggatgctg

122101 tcagccgcgt gaagaacctg atggcacaga aagacattga gaatatgctt tctgtcctcc

122161 gcgaatatgg ttcctgtgac ctgtccatta ctcggaatag tcacgggcat cactacagcc

122221 attggaactg catgaacatc gacgacaacg tggacttcac agggatcttc cctgatgatg

122281 attccatgat tggcaccggc attgaaggga ttaaccagga tatggtcgag cgctggcaag

122341 acctctggga acgctttgtg ctggagctgg cggatgatgt aaaaagtctt tccaagaagc

122401 tcgaagcgga cgggtactcg ctgatcgagg cctctccatg cgaagatgaa gtggtttggg

122461 aacgggccac tgaaaactac ctggtgcgtg ttactgaact ccctgagcgg gatttcgata

122521 tgggccactg ggatgacgaa gtaagagacc aaacaatttg ttctatcctg gaagggaaag

122581 agcgagtgct tggcctacgt gttgaggtac tctctcgtga aaacgagatt gtcctgggtg

122641 aagagagtct gcacggcttg accgttgcca gtgatgacaa gagctacgct ggctacagac

122701 gagaacttct ccggggagct atccagcaaa ccagggactt tttctctcgc cacctcaaag

122761 cggcataaca aggcaggggg aaacccctgc tttacttccc aagtcttctt actcatggtg

122821 aataccatca gtaagcagat tttgttctct tcggagagcg aaaagtgcga tagctggtcg

122881 ccaaaaacaa acagcaaatt aacgttaatt tattaatccc tgcgggagtc tatctcctgc

122941 tggagatacc tcccgttatt tctggaggta tccatgaaca aattccaaga ctttaagttg

123001 gtttatcgtc aaggtggtcg tcgcttagag cgtgtcttta cggacactct ttacgcgaat

123061 gtgaagcgtg tcgctgactc gtttcctccg accgttcttt ggcgcattca gccagcgtaa

123121 aactcggaac cttaaagctc ggctttggcc gtgacctttc aataacccat gcgggtacac

123181 ttccccgcac ggggtaggtg tacccattaa ctttggagta caccttatga accaagtagc

123241 taaattggac ctcgcccaaa tccgtcaaca agccatcaat gatggtctgc tggtggacca

123301 atcttccatc ggtaaacaag ctggtttcct gaccaatgtg gcagtgaccc cggcaattgt

123361 cgatggagta tttggtgcag atggtaagca ctcggtagaa gacttccttt tcatgttttt

123421 gcagctttgt gttgctcaaa caaaggtcgc ttttaccgac aacaagaatt ggggaaagat

123481 tcgtctttat taccccatgc ccactgtaga tggctttttc aaacccactg aggttgtgat

123541 taagtcagat cctgtaaccg cagatgtcac aatcatgatg gcctcggaag agggcgctca

123601 tttgtgcttg tgatattcac aaaattcgcc tccgtttcgg gggcgacctt tttaaacccc

123661 tcgccgggga gaactccccg gctggggagc ctccttggca cattctcccg gtgtcggggt

123721 ttccttaaaa ccctggcgac gggggtttaa ggaagcctcg atgctggggt tcacgctaag

123781 gagaaaacct atgtcttact acaaagctga caccgttcgt gaagctgcaa acggaaactg

123841 gctattcatc ctggcggccc tagcgcccca tcttgaacca gctctccgta aacctggtcg

123901 tcatgtttct tgtcctatcc acggcgggaa agatggcttc cgactgttca aggatgccca

123961 cctgactggt ggcggcgtat gcaatacatg cggtgccaat catgatggtt ttgagctgct

124021 gatgtggctc aataactggg actttaaaca gtgcctgagc gaggttggtg attatctggg

124081 cgttgagaaa gagcaacccc agtatcaaca agccgctgca ccgacacgag ctcctgtcca

124141 ggccaaagcg cctgttcagc aagagcctat gaaggtgaat aacaaggttc tcgattccaa

124201 gaatcgtaaa aaatccattg ccggtactct gattgcccac ggtaaagctc cttatgagca

124261 taacgaagac aacgagctca gctactttgc cttcatccgt gacaagagcg gtttggaacg

124321 caccatctgg ggcgtggatc ttgaaagagc cattggtgaa agtgaagcca agtatgggga

124381 tgagatcgtc atgacaaacc tcgggcgcga gcctgtaact gtcgtcgttg aagttaagga

124441 cgagcagggg aatgttgtga gagaacaacc tatgcaaacg catcgcaaca cctggctggt

124501 ggaacgtcgc ggcgctacgg taacgcagtt ccgcgctcgc tcgaacggtg gtgttgagcc

124561 ggtgagtcac catgttgagt cggcccctgt ggtcaaccgc aaggtagaaa ctccggcacc

124621 gcaagtacaa cctgcggcca cacaaccgga agagcaaagc agcgaaaata agccgaaagt

124681 tgttccgatg tttcgtgaac aacctaagcc ttggttgctt gagctccaag aagaaatgga

124741 gaagagaatg gagcgcgaac gcgcttacag tgctcgtctc cgtgagaaaa tcgagaaggt

124801 atggaacgag tgtttgccgt tctccagtca tgtgactgag ccaatgcgtc tgtacttcaa

124861 aaaccgcgag ctgctgttca aagttgatga agtagaaaaa acagactgtc tgcggttcaa

124921 tccggctatg gcctactacg acgaagatgg caatgaagtt gggaaattcc cggctatcgt

124981 ctgcgctatc cgagatgtgg aaggcaacct ggtaacgctc caccgcacct atctcaccca

125041 aaacggtaaa aaagccaagg tcggcaacgc caaaaagatg atgcccattc ctgacggttt

125101 ggatgtcaat ggcgcggcca tccgcctcgg tgaaccgact gagggtatcc tgggtgttgc

125161 agaagggctg gaaacagctc tgtcagctta tcgagtcact caaatcccgg tttggtcaac

125221 ggtcaatgcc accctaatgg agtccttcga ggttccagaa ggtgttcaca ccgtactgat

125281 ctgggctgac aaagataagt ctgtgactgg ggagaagtca gcgaacgtgc tgaaagccaa

125341 gctggagaag cgcggcattc gtgtgtacgt cctgctgcct aagctcccga tcccgcccag

125401 agcgaaaggg attgactgga acgatgtcct gatgagtcag ggaagcctcg gtttcccgaa

125461 tgctcgctac ctgcgcgatt tcattgcgag aaggagagct gagtatggcc gtcattgatg

125521 tctcgaaggt tgatacaacg cctggtaacg acgcggtgtg ccccttctct ccccctgagg

125581 ggtgggaggg ggactctgcg gcctacgttg agcttatgcg gtctcggtat cgtcatctga

125641 tgcacggcca gagaatgatg gtgacagcct ccttcgcaag aagggagcct atccaagtta

125701 ctggcccgtt tgctgatgaa gcgacgaaga tcattaactc aatgaagatg aacaaggcga

125761 agccaacagc tttgtctgcc taaaacttta accctgaggg ggtgttcatg ccctctcagg

125821 catgattcgc ctcctcttca actatggagg taatcatgaa aaagtttttg cgtattaaga

125881 cgtggtttgt gcgtcttttc tctcctgaca agaagactct gggagctatc ggtgaagacc

125941 tgcgtaaggt cgccgtaaca gccatcggtg tcggtattgt aggggtcgtc tcagaaaacg

126001 gaatctatgg tcactcccgt ttttgcaaca ccgattttga cgataaattg gcttgcttga

126061 atctatccgg cgtctgaatg ggattttatt cccgcgcctc gatgagttcc gcgcctgatg

126121 aacctccaga aaatatacgg cttcaatgag cctttccgtt ttacaggttc ctcaacaggc

126181 cggtgggccg ttagtatcat caatatcagt attcgcaaaa ccagatcagt gattctttaa

126241 accggtgtat ttctgccgtt atgctacata agtttgctgt cgtgccgtta gggcccaggc

126301 tattctggcc agcttgtttg ccagagcaca agtgacgaca aagttgcttt tcctacacag

126361 tagatccctg acccaatcgg ccaatttgcc agactggtgt tccagttttt gtatgaatac

126421 cctggcacat tgaaccaaca aagttcggat ctttttgtta cctcgcttac taattcccag

126481 caatgtcgtc cgacctcccg tgctgtactg tcgaggcact agccctgttg ccgccgcaaa

126541 gtcacgactg ctggcgtact gcttcccgtc gccaatctca gttgaaatag tactcgctgt

126601 cagtgttccg acgcagggaa tgctcagcaa gcgctgtcca atctcatctt cgtccaactt

126661 tcgtttcaac tgggattcca aatctttaat ctgctcaaca agatagtgat aatgctgttg

126721 taatttcagc aataactggc tgaggtatag aggcaaacta ttgtcctcaa gaagggtact

126781 cagtcggcta ataacggcag ctcctcgtgg aacgctgatg ccaaattcca gcagaaaagc

126841 atgcatctga ttggttgttt ttaccttatc ctgaaccagg gattcacgga cacgatgcag

126901 cgcacgcatt gcctgctgag attcagttct gggctgtaca aaacgcatag acggacgcga

126961 tgcagcttca caaatagctt cggcgtcgac aaagtcgttt ttgttacttt taacgaatgg

127021 acggacaaat tgtggtgata tcagcttagg aaaatgcccc aactcttcca acttgcgtgc

127081 cataaagtga gagccaccac aggcttccat tgcgatggtt gtagcggggc atgtcgccaa

127141 aaattcgatt aactttggcc gtgtaaattt tttacggtaa acagccttgc cgcgacgatc

127201 ttggcaatga atatggaaag agtttttacc cagatcgata ccaatgagcg caatgttttc

127261 catgatagtt ctccgaatga aagcctatcc tcagcatagt accgggaagg agggagtgac

127321 catctcatta aataaagcac gctaagccgg tggcagcggt cgcaatggcc taaacttccc

127381 cgcaccgacc ttggcgctgc tgcgccatag gtaatcgccg gtcaggttga tgtgctccca

127441 ccccagcggc gacagatatt gcaacaatgt gtcgtccagc gccgtgccgt tgccacgcaa

127501 agcactggtg gcacgctcca gatataccgt gttccacaac acgatggccg ccgtcaccag

127561 attgaggccg ctggcccggt agcgctgctg ctcaaaactg cggtcgcgga tttcacccaa

127621 tcggtagaag aagaccgccc tggccagcgc gttgcgcgcc tcgcccttat tcagccccgc

127681 atggacgcgg cggcgcagct ccacgctttg cagccaatcc aaaatgaaca gcgtgcgctc

127741 gatgcgcccc agctcgcgca acgccacggc caagccgttc tggcgcgggt agctgccgag

127801 tttgcgcagc atcagcgaag ccgttaccgt gccttgcttg atggaggtgg ccagccgcag

127861 aatttcatcc caatgggcgc gtatttgctt gatgttcagc ctgtcgctgc taatcatcgg

127921 cttgagcgcg tcataggcgg catcgccctt ggggatgaat agcttggttt cgcccaagtc

127981 acggatacgc ggcgcgaagc gaaatcccag caaatgcatc aagccaaaca cgtgatcggt

128041 gaagcctgcc gtgtcggtgt agtgttcctc gatgcgcaag tccgactcgt ggtacagcag

128101 gccatcaagc acgtaagttg aatcacgaat gcccacgttg accaccttgg cactgaaggg

128161 cgcgtactgg tcggagatat gggtgtagaa agtccgtcct ggactgcttc catacttcgg

128221 gttgatatga ccagtgcttt ctgctttgct gccggttctg aagttctggc cgtccgacga

128281 tgacgtggtg ccgtcacccc agttgccggc gaagggttgc cgaaactgcg cattcaccag

128341 ctcggccagc gccgtcgaat aggtttcatc gcggatgtgc caggcttgca gccaagacag

128401 cttggcgtag gtggtgccag ggcaggactc ggccattttg gtcagaccca ggttgatcgc

128461 gtcggccagg atcgtcgtca acagcaaggt tttgtccttg gccgtgtcgc tggtcttcag

128521 gtgtgtgaag tggcgggtga agcccgtcca ttcatcgacc tccatcagca actcggtgat

128581 tttgaggtgc ggcagcagca tagctgtctg gtcgatcatg gcttgcgcgg cgtctggtac

128641 tgccgcgtcc agcggcgtga tcttcaggcc tgacgcggtg gtgatgatgg catccggtaa

128701 gtcgttggcc gcagccatgc ggttgactgt ggcgagttgc gcctccaaca attccaaccg

128761 gtcatgcagg tattggtcgc agtcggtggc cactgccagc ggcaattcgc tggccagctt

128821 caaagtggcg aacttctcga ccggcaccag gtattcgtcg aagtccttga actggcgaga

128881 accctgcacc cagacatcac cggagcgcag cgcgttcttc agctccgaca gggcgcataa

128941 ctcgtagtaa cgccggtcga tgccgtcgtc ggtcagaacc agctttgccc agcgcggctt

129001 gatgaatgcg gttggcgcat cggcgggcac cttgcgcgcg ctgtcgctgt tcatgccgcg

129061 cagcatgtcg atggcatcga gcacaccctt ggcggcgggc gcagcccgca atttgagcac

129121 gcccaggaac tgcggcgcgt agcggcgtag cgtggcatag ctttcaccga tgtggtgcag

129181 gaaatcaaag tcggcaggcc gcgccaatgt ttgcgcttcg gtgacgctgg cggcgaaggt

129241 gtcccagggc ataacggcct cgatggcggc gaacggatcg ctgccgcttt gcttggcctc

129301 aatcaacgct tgaccgatgc gcccatacat ccgcaccttg tcgttgatcg ccttgccgga

129361 agcctggaac tgctgctgat gcttgttctt ggccgcgttg aacagcttgc cgatgatgcg

129421 atcgtgaagg tcgatgattt catcggtgac ggtggccatg ccttcgatgg ccagcgctac

129481 cagcgtggca tagcgtcgtt gcacctcgaa ctttgccaga tcagcaggcg tcatctggcc

129541 accttcacga gcgattttga gcaggcggtt ctggtgaacc tgccgctcga tgcctgcggg

129601 cagatcaagt gcttgccagg atttcaggcg ctcaatatgt tcgagcatgt ggcgagagtt

129661 cggtttggca ggcgactggc gcagccatgc cagccacgtc actttactgc cgtccttgcg

129721 cttgagaagt tcgtccaggc gctgacggtg gggtgataac aaagaatcgg tcagcgccgc

129781 gtaaatgcgt cggttggcac gggtgatggc ctcggcgctt gcgcgctcga tggcattcat

129841 ggcgggcagg ataatgctct gccgccgcag attctcgaca agtgcgctcg ccagcacgat

129901 gcctttgtcg gtctgcaagg ccagctcggt caatgtatgc acggcttgcc gatagtggct

129961 catggtgaag ggcttgaacc caaaaaccgt ttgcagctcg accaagtgct cccgccgtgt

130021 ctgttcgcgc tggccgtact cgctccaact ttccactggc atcttgagtt gcgcggccac

130081 catgcgcaac aggggcggaa acggaggctc atcgacgccc aaaaaggtgc cagggaatcg

130141 caagtagcaa agctgcacag cgaagcccaa tcgattcgcg gcgccgcgac gctgacggat

130201 caccgacagg tcggtttcgt tgaacgtgta gtgccgtatc agttcgtctt tggcatctgg

130261 cagtgccagc aggctttcgc gctcggtggc ggacaggatt gagcggcgtg gcatggtcag

130321 tcttcccgca ggtactggta caaggtttcg cggctgatgc cgaagtcacg ggccaccaag

130381 gttttttggt cgcctgccgc aactcgccgt ttcaactcgg caatttgttc gctgttcagc

130441 gatttctttc gtccccggta ggcaccgcgc tgcttggcca gcacgattcc ctcgcgctga

130501 cgttcgcgga tcagggcgcg ctcgaactca gcgaaggctc ccatgaccga cagcatcaga

130561 ttggccatcg gtgagtcctc gccggtgaac ttcagccctt ctttgacgaa ctccatgcgc

130621 acgccccgtt gtgtcagccc ttggacgatg cggcgcaggt catcaaggtt gcgtgccagc

130681 ctgtccatgc tatgcaccac cacggtgtcg ccctcgcgga cgaaggccag cagcctttcc

130741 agctcgggac gctgggtgtc cttgccagaa gccttgtcgg tgaacacccg cgccacctga

130801 acaccctcca attgccgttc cgggttctgg tcgaagctgc tgacgcggac atagccgatg

130861 cgttgacctt gcaagatgcc tccaaaggca aaagtgtcag gatgaaatct attacctttg

130921 acggaatatg tcaatcaata ggaaatttaa ctctattctg acatcgtttg cacatggtgt

130981 cgttttcaga agacggctgc actgaacgtc agaagccgac tgcactatag cagcggaggg

131041 gttggatcca tcaggcaacg acgggctgct gccggccatc agcggacgca gggaggactt

131101 tccgcaaccg gccgttcgat gcggcaccga tggccttcgc gcaggggtag tgaatccgcc

131161 aggattgact tgcgctgccc tacctctcac tagtgagggg cggcagcgca tcaagcggtg

131221 agcgcactcc ggcaccgcca actttcagca catgcgtgta aatcatcgtc gtagagacgt

131281 cggaatggcc gagcagatcc tgcacggttc gaatgtcgta accgctgcgg agcaaggccg

131341 tcgcgaacga gtggcggagg gtgtgcggtg tggcgggctt cgtgatgcct gcttgttcta

131401 cggcacgttt gaaggcgcgc tgaaaggtct ggtcatacat gtgatggcga cgcacgacac

131461 cgctccgtgg atcggtcgaa tgcgtgtgct gcgcaaaaac ccagaaccac ggccaggaat

131521 gcccggcgcg cggatacttc cgctcaaggg cgtcgggaag cgcaacgccg ctgcggccct

131581 cggcctggtc cttcagccac catgcccgtg cacgcgacag ctgctcgcgc aggctgggtg

131641 ccaagctctc gggtaacatc aaggcccgat ccttggagcc cttgccctcc cgcacgatga

131701 tcgtgccgtg atcgaaatcc agatccttga cccgcagttg caaaccctca ctgatccgca

131761 tgcccgttcc atacagaagc tgggcgaaca aacgatgctc gccttccaga aaaccgagga

131821 tgcgaaccac ttcatccggg gtcagcacca ccggcaagcg ccgcgacggc cgaggtcttc

131881 cgatctcctg aagccagggc agatccgtgc acagcacctt gccgtagaag aacagcaagg

131941 ccgccaatgc ctgacgatgc gtggagaccg aaaccttgcg ctcgttcgcc agccaggaca

132001 gaaatgcctc gacttcgctg ctgcccaagg ttgccgggtg acgcacaccg tggaaacgga

132061 tgaaggcacg aacccagtgg acataagcct gttcggttcg taaactgtaa tgcaagtagc

132121 gtatgcgctc acgcaactgg tccagaacct tgaccgaacg cagcggtggt aacggcgcag

132181 tggcggtttt catggcttgt tatgactgtt tttttgtaca gtctatgcct cgggcatcca

132241 agcagcaagc gcgttacgcc gtgggtcgat gtttgatgtt atggagcagc aacgatgtta

132301 cgcagcaggg cagtcgccct aaaacaaagt tgggcgaacc cggagcctca ttaattgtta

132361 gccgttaaaa ttaagccctt taccaaacca atacttatta tgaaaaacac aatacacagc

132421 atcgtgacca acagcaacga ttccgtcaca ctgcgcctca tgactgagca tgaccttgcg

132481 atgctctatg agtggctaaa tcgatctcat atcgtcgagt ggtggggcgg agaagaagca

132541 cgcccgacac ttgctgacgt acaggaacag tacttgccaa gcgttttagc gcaagagtcc

132601 gtcactccat acattgcaat gctgaatgga gagccgattg ggtatgccca gtcgtacgtt

132661 gctcttggaa gcggggacgg atggtgggaa gaagaaaccg atccaggagt acgcggaata

132721 gaccagttac tggcgaatgc atcacaactg ggcaaaggct tgggaaccaa gctggttcga

132781 gctctggttg agttgctgtt caatgatccc gaggtcacca agatccaaac ggacccgtcg

132841 ccgagcaact tgcgagcgat ccgatgctac gagaaagcgg ggtttgagag gcaaggtacc

132901 gtaaccaccc cagatggtcc agccgtgtac atggttcaaa cacgccaggc attcgagcga

132961 acacgcagtg atgcctaacc cttccatcga gggggacgtc caagggctgg cgcccttggc

133021 cgcccctcat gtcaaacgtt agatgcacta agcacataat tgctcacagc caaactatca

133081 ggtcaagtct gcttttatta tttttaagcg tgcataataa gccctacaca aattgggaga

133141 tatatcatga aaggctggct ttttcttgtt atcgcaatag ttggcgaagt aatcgcaaca

133201 tccgcattaa aatctagcga gggctttact aagcttgccc cttccgccgt tgtcataatc

133261 ggttatggca tcgcatttta ttttctttct ctggttctga aatccatccc tgtcggtgtt

133321 gcttatgcag tctggtcggg actcggcgtc gtcataatta cagccattgc ctggttgctt

133381 catgggcaaa agcttgatgc gtggggcttt gtaggtatgg ggctcataat tgctgccttt

133441 ttgctcgccc gatccccatc gtggaagtcg ctgcggaggc cgacgccatg gtgacggtgt

133501 tcggcattct gaatctcacc gaggactcct tcttcgatga gagccggcgg ctagaccccg

133561 ccggcgctgt caccgcggcg atcgaaatgc tgcgagtcgg atcagacgtc gtggatgtcg

133621 gaccggccgc cagccatccg gacgcgaggc ctgtatcgcc ggccgatgag atcagacgta

133681 ttgcgccgct cttagacgcc ctgtccgatc agatgcaccg tgtttcaatc gacagcttcc

133741 aaccggaaac ccagcgctat gcgctcaagc gcggcgtggg ctacctgaac gatatccaag

133801 gatttcctga ccctgcgctc tatcccgata ttgctgaggc ggactgcagg ctggtggtta

133861 tgcactcagc gcagcgggat ggcatcgcca cccgcaccgg tcaccttcga cccgaagacg

133921 cgctcgacga gattgtgcgg ttcttcgagg cgcgggtttc cgccttgcga cggagcgggg

133981 tcgctgccga ccggctcatc ctcgatccgg ggatgggatt tttcttgagc cccgcaccgg

134041 aaacatcgct gcacgtgctg tcgaaccttc aaaagctgaa gtcggcgttg gggcttccgc

134101 tattggtctc ggtgtcgcgg aaatccttct tgggcgccac cgttggcctt cctgtaaagg

134161 atctgggtcc agcgagcctt gcggcggaac ttcacgcgat cggcaatggc gctgactacg

134221 tccgcaccca cgcgcctgga gatctgcgaa gcgcaatcac cttctcggaa accctcgcga

134281 aatttcgcag tcgcgacgcc agagaccgag ggttagatca tgcctagcat tcaccttccg

134341 gccgcccgct agcggacctg gtcaggttcc gcgaaggtgg gcgcagacat gctgggctcg

134401 tcaggatcaa actgcactat gaggcggcgg ttcataccgc gccaggggag cgaatggaca

134461 gcgaggagcc tccgaacgtt cgggtcgcct gctcgggtga tatcgacgag gttgtgcggc

134521 tgatgcacga cgctgcggcg tggatgtccg ccaagggaac gcccgcctgg gacgtcgcgc

134581 ggatcgaccg gacattcgcg gagaccttcg tcctgagatc cgagctccta gtcgcgagtt

134641 gcagcgacgg catccatgtc tcgacgatgc cagcggagtg cttaagctac tcctagcgca

134701 ttgggcgctg ggacggggcg ggggtgcata gccatgcacc ggcggataag cgtgcgcgcg

134761 gctcgctcca ccgatttggc taggggagcg cggtgctgcg ccagctttct agaatgtcgc

134821 ttacttcgcg gctgacaatt aaccgcgcct cggcgcgagc cataccggag atcgccaaaa

134881 gtcgatcgta gtcggtaagt tggtggcggg cgaaggttga ggccacaatg ccgacgacct

134941 tgcctagcga ggcttctgtc caccgtttgg ccagaatccg ttcgatgagg atctcgcggt

135001 attccggcgg gcagcgagct tgatgtttgc gaaaatgtcg ctcgaccgtc cgccgggtga

135061 ataatgtgcc ttgctgcatg cgatgcctcc aaggagacgc tcgcatcggc ctacgccaaa

135121 aggggtcgca tgaggtgctt gggggccgcc aaaagggtag ctcagcgaca tgccttccga

135181 tgttgccgtc agctaatcgc caaccttggg gggcggcagt tcgttcggcg ttccccacga

135241 caatagccag gctttcacag cctcgtggct gatagtccgg cctgcctcaa tgtcggcaat

135301 gccctcggcg gtcgcttcct ggtcgtcgtc aaagttgtcg gcttgggagt tcacgcagtc

135361 caactaccac aacacccgac cgcccggcca caccggtccg acgcctacgc ctttacggcc

135421 acacggtgta atggaggaga gagagccaac acatgcgcca gcgcgaatgg cttggcggcg

135481 gtccgcagga tttcagtcgc cgcctcgtag gcttcagacg ggaccactcg gtccggaggc

135541 gttgcggcgg gacccctagt gtgcgccctt cacagaccct gaggagcgca ccgctcgcct

135601 tcttgctgcc catcgcgata gcggggacat cacaactgag caaactcagg gacgcccagt

135661 gttgcggcgg gacatccact tctgcgtttt tacagctgcg cgccggcggg catcgcaaca

135721 gccatgcgaa cgccggtgtc gtacccgcca tcgctttcgc gcagaacaac cgtggtgaag

135781 tgcgcttcga gtcgggccat ggccaggtgg cttgcaccgt gctgtccaac ggcaagccgg

135841 gctacggcgt gccgatggtg gcctgcgtgt ctctgcgcgg ccgcgagcag ggccttgcag

135901 cggagctggg cggcagcgta gcggccacac tgcgtaccag cggcggcggc gcggacaagc

135961 cccatgtgct ggcccccgac ttcgaggcgc atttccgcta cgactggaac gaccccggcc

136021 ccggcgactg gtcgcactgg cgggtgcggc ggctgatgcc gacggagtgc gagcggctgc

136081 aaggcatgcc cgatgactac acgctgatcc cctaccgcgg caagcccgcc gcggatgcgc

136141 cgcgctacaa agcaatcggc aactccatgg ccgtcccgtg catggcatgg ctgggccagc

136201 gcctggtgca gtgcctgcac aagacgggct cgaccgcttc ggattgatcg gcgacgcagc

136261 ctgcgggccg cgccattctc cctcctgcat tgccgacgta tcggcagcag cccgcagcca

136321 gggtgtcaag gctgccgtgg gttccccccg cgccacccgc cagttcgcct cggcatctca

136381 ccggcgaacg ctgcccaccg gggcatcgat gcctcttttc tccaacccac gggatggccg

136441 ccacgtcccg tcaggggcat gtcgccaccc ggtcgatttc aggagaattt taaagttttt

136501 accatcattc ttgtcactgt tacctttcga actcacatta aattctttca tcttctaaaa

136561 attaatatta actaatgtat aatcctactg tgaacaagaa aacgcgtgca aaatatgtat

136621 tagaagagat ggaaaagctt tttccggata gtagctcaga gttgaggaac tgggaaacag

136681 atttccaatt tcttctttgc atcattttat cagctcagac gactgatttg caggtgaata

136741 aagtgactca taatctattc gctaaatatc ctgatccggc ttcactttcg gaggcggaag

136801 tcggagaggt agagaagata ttaagctcta taaattacta tcgcactaaa tctaagaata

136861 ttgtaaatgc ggctaaggtt gtgaaaacta ggtttcacgg ccgagttcct aggagtgtag

136921 aaaaattgat cgaaatccca ggcgtcggat tgaagactgc taatgtatat ttgaatagta

136981 tgtatcaagc gaatcaggga gtgggagcag acactcatgt gatgcgagtt tcgaggagac

137041 ttggtttcac ggatagtcgt gaccctagga aggtcgcaat cgcgctacaa aagctgtatc

137101 cgaaaaaaga ttggtaccga gttacggcgt tatttgtctt gtatgggagg tactattgca

137161 aggctagagt caagccagaa aatagcaaat gtatttttaa ggaattttgt actcactgta

137221 ggtaatgact ccaacttact gatagtgttt tatgttcaga taatgcccga tgaccttgtc

137281 atacagctcc accgattttg agaacgacag tgacttccgt cccagccttg ccagatgttg

137341 tcgcagattc aggttatgtc gctcaatgcg ctgagtgtaa cgcttgctga taacgtgcag

137401 ctttcccttc aggcgggatt catacagcgg ccagccatcc gtcatccata ccacgacctc

137461 aaaggccgac agcaggctca gaagacgctc cagtgtggcc agagtgcgtt caccgaagac

137521 gtgcgccaca accgtcctcc gtatcctgtc atacgcgtaa aacagccagc gctgacgtga

137581 tttagcaccg acgtagcccc actgttcgtc catttcagcg cagacaatca catcactgcc

137641 cggttgtatg cgcgaggtta ccgactgcgg cctgagtttt ttaagtgacg taaaaccgtg

137701 ttgaggccaa cgcccataat gcgtgcactg gcgcgacatc cgacgccatt catggccata

137761 tcaatgattt tctggtgcgt accgggctga gaggcggtgt aagtgaactg tagttgccat

137821 gttttacggc aatgagagca gagatagcgc tgatgtccgg cagtgctttt gccgttacgc

137881 accacgcctt cagtagcgga gcaggaagga catctgatgg aaatggaagc cacgcaagca

137941 ccttaaaatc accatcatac actaaatcag taagttggca gcattaccaa tcatctgttc

138001 tccaatgact ggtctaaaaa ctagtattaa gactatcact tatttaagtg atattggttg

138061 tctggagatt cagggggcca gtctagtgat cttgaccctc catctccgaa cagcttttgt

138121 atcttaagtt aacgatgtcc gctgccgccg gtattccctc ggggagtgat atcccagcgc

138181 gctgtgcggg tggttttcat tgtaatgcgt gaacgctact gcaaggtttc gcagtgctgt

138241 tctcacatcc ggttttggca tgaacgcgat atagtcttct ttcatcgttt tcacgaaccg

138301 ttcggccatg ccattactct gcgggctgct caccgctgtt gtgcagggct ccagattcag

138361 ctctctcgcg aacctccacg tttcatacgc ggtatacgct gaaccgttgt ccgtcagcca

138421 ctgcaccgct gtatcgggca acctgtcgcc gaagcgcttt tccaccgacc tcagcatcac

138481 atcctgcacg gttgaactgt catagcctcc cgtgcttgct gcccagtcta tggcctcacg

138541 gtcgcagcag tccagcgcga acgttacccg cagtttttcg ccgttgtcgc agccgaactc

138601 gaagccatct gaacaccagc gcatatcgct ttctgccacc gctatcttgc ccttatgttc

138661 acgcttcggt cgctctggtt tgtcatgcaa caacaacagg ttatgctcgc tcattatcct

138721 gtaaagccgt ttggcgttca caggtggctg tccctctgtg cgacgttgct tgcgcaggat

138781 gccccacacg cgtcgataac cataactcgg catatcgctg atgatgtcga ggatagccga

138841 cagtatttct tcgtctgctt cgtcattacg ccggttacag cgcttgtcct gccagtcggc

138901 agaacggtta atccgcagtg acagctgcgc acgcgacacg cccatggtgc ggctgaccag

138961 ggctattccc cgtcctttgg caacaagggc gcgtgcgcta tccattttcg cgactggccg

139021 tactccacgg cttctttcag gatctcaact tccatcgtct tcttgcccag aaggcgctga

139081 agttcccgga cctgcttcag agcagcagta agctcagaag caggaacgac ttcctctccg

139141 gccgcaacgg cggtgaggct gccttcctga tattgcttct tccacttaaa cagcaggctg

139201 ggctggatac catgcaggcg ggcgacatgg gagacattca tgcccggctc catcgtctgc

139261 tggataatgg cgatcttctc ctgaggagtt ttacgtttac gggcttcttg ccctaacagg

139321 atcccggtca tctcaaaatt ggcgttagtg ttagacatat attcaagcct atctcttatc

139381 tggagataca gctactgtcc ggtgtttcag ggggctacat cattatgggc gttggcctca

139441 acacggtttt acgtcactta aaaaactcag gccgcagtcg gtaacctcgc gcatacaacc

139501 gggcagtgat gtgattgtct gcgctgaaat ggacgaacag tggggctacg tcggtgctaa

139561 atcacgtcag cgctggctgt tttacgcgta tgacaggata cggaggacgg ttgtggcgca

139621 cgtcttcggt gaacgcactc tggccacact ggagcgtctt ctgagcctgc tgtcggcctt

139681 tgaggtcgtg gtatggatga cggatggctg gccgctgtat gaatcacgcc tgaagggaaa

139741 gctgcacgtt aacagcaagc gttacactca gcgcattgag cgacataacc tgaatctgcg

139801 acaacatctg gcaaggctgg gacggaagtc actgtcgttc tcaaaatcgg tggagctgca

139861 tgacaaggtc atcgggcatt atctgaacat aaaacactat cagtaagttg gggtcattac

139921 ccaggcacag ttaagcaaag aagatcgcat ttacgtcaaa aagtccattc gctggcttac

139981 cctgaagcga cccggcaacc tgacgcccgc agagcaaaag gcgctggagg ttgtaaagca

140041 ggcgatccca gcacttgcca tggcctatga cttcaaggaa gccttcttct gcatctacga

140101 cgaacccgac aagcagagtg ctcagaacgc ctttgaggct tggaagaaca gcctgccgcc

140161 ttatggcatg gagcctttca aaaaactggt aaaaacggta cacaaccatt acgacgacat

140221 ctttgcctac tgggatgcgc cgttctcact caccaatggc tataccgaag ggctcaacgg

140281 tttgatcaag atgtccaacc ggttaggtcg gggttatagc tatgagatca tccgtgcaaa

140341 gacgttgtac tccaaagaag cccgcaaggt tggcagtggt attcgagcag gacgaggcaa

140401 ggtcgagtat ggaccgcaca ttccgacatt gttgaagcag gcagaaggtg gagagttgga

140461 ttaacgcgag agagggcgcg tctgagaatc caaccaatgt tagattttcg cagggtggtg

140521 aaaaacagaa aaatccaacc agaagcagct ttttgtccga ctaagaatca agggttagaa

140581 tggatgaatt ccaaccctag aatgcaaata gcccatataa taattccttt cagacaggta

140641 ccggttaagt gtagctgagt cagacaaaaa agccccctga cggggggcaa aaatgtagca

140701 acgttgtttc aggaaaatct atcagggaaa aggtaacagc acggatacta ccgtcgcatt

140761 cagcctgcat tgatcatgcg gcggtgcgct tcggccatgg cctgatgtgg gccggattgc

140821 ccgttattca tgaattcatg agcaaccgct gctctttcat gctcattcat cgccgcgtaa

140881 gacgttgacg ggacggtcgt ggataccgtg ttatttccca tcatcttctg atgactttcc

140941 accattttct ggtgagcatc cgccgagccg ttcgtcatgg tttcatgagc aataatggcc

141001 tgttcatgct ggtccatacc ggtcatacga ggagcagtcc cctggatccc gacaggagcc

141061 tcagcagact gcatctggtg agcaggagcc tgtgcattgt tgacgcgatc atggatattc

141121 acggtttcag tggcccaggc cgatgaaatt aaaccaaagc ccagcaaaga tgctaatacg

141181 atatttttca tgataactct ccatttctga attagtgatg tccggggaag tacaaccggt

141241 gttttcagtt ccataactga aacaagttcg gcagcgttta tttctccgga agagggctgg

141301 atattcattt cctggcgtac tctgacgccg ggtattgatg aagcaatcca gccttcctga

141361 taagttgcac taattatatc gaatggcttc tgtttgctgc atgacaggct aatgacatct

141421 ttgtcattta catctttatt ttcagcattg ggtttccggg atcattttct ccagtctggg

141481 cacggataag ataaaacgcg tggagcgtac gtctgattcc acctgcactc ttccgtgatg

141541 tgcttccacg attgacttca caatcgcaag gccgatgccg ctgccttctc cttttcgttg

141601 tctggatgga tccacccgat aaaaacggtc aaacagcctt gataaatgtt cttcagggat

141661 cggtttcccc ggattttcaa tcacaaggtc aaaaaagctc tcctgctctc ttattgagac

141721 ggtgattgcc tgtccctccg gggtataacg cagggcattg gataacagat tattgatcgc

141781 ccttctgaac atttgtggat ctccctcaac caggcagggc atcccgttaa atttgagcgt

141841 gatattgcgt tcttcggccc aggcttcgaa aaactcgaag actttcatga cttccgctct

141901 gaggtcaaac atgaccctgt caggtatcag ctgattatta tctgcctgtg ccaggaacag

141961 catatcgctg accattttgg tcatccggtt atactcttca agactggaat agaggacatc

142021 ctcaagttcc ctctgtgttc gatcctgact cagtgcgatt tcagtctgcg tcaccagatt

142081 ggtgatgggc gttctgatct catgcgcgat atcggcagag aaattggcct ggcgggtaaa

142141 gacatcctca atctttccaa tcatatgatt gaacgagata accagttgct ccagctcaat

142201 gggaacgcgt gtcggttcca gtcgcgcatc aagattctcg gaggtgatgt ttttaatggc

142261 attgctgaca ttacgaaggg gcaggtgccc ctgacggaca gcgattcgaa tgatcagaac

142321 aatcaacagg cttatcacga cggcaatcgc aatcaggttc tttttcagcg catcgaggta

142381 atggagatgg aaattaatgg ataggccagt cagcatgaca tagttctgct gtttgccctg

142441 aaatatcgcc tgaccagagg aggcgataat cctgtatgtt tccatcttca tttcggaccc

142501 ggtatccatc ggtcccgcag gatcctccac cgtccagaga aagacatccc gtgcgcggct

142561 gtgctcgcta aaatctgctg aattcactgc cgggcgtagt gccgccccct gagctgagct

142621 aaagagcact tcacccctgg gattgaggag caaaagggca acgttgcggt agctggcaat

142681 tgattccttt attttgctta cttttttttc atccggatct gccggggact ggagtatacg

142741 gctcagtgtg gtgctgattt gttgcagatc gctgacatcc tgctcggcaa aatgcttttc

142801 aacagaatgc agcataaacc aggtgaaggc gataaacgcc agtattgtgg acaggctgat

142861 aaaaaaggtc agccgcagag cgagtgagaa aggtcgtctg gacggtttgc tatgcatccg

142921 ggatctccag catgtagccc acgccccgca ctgtctggat cagttttgtc ccgtaatcgt

142981 tgtctatttt agcgcggagt cgctttactg cgacatcaat cgcgttagtg tcgctgtcaa

143041 aattcatgtc ccagacctga gaggcaatca gggagcgggg aagaacttct ccctgatggc

143101 gaatgaagaa ttccagcaag ctgaactctt tactggtgag cacaatgcgg ttcccggcgc

143161 ggctgacttt tctggatacg agatcaaccg agaggtcagc cactttaagc tggctttccg

143221 tgatcatcgt gtttccccgc ctgaggaggg ttcttacccg ggcgagcagt tcagcaaacg

143281 caaagggctt caccagataa tcgtccgcac ccagctcaag tcctttgacc ctgtgttcaa

143341 tcgtgccaag ggccgtcagc agtaataccg gcatcccctt tccggcactg cgcagcatac

143401 ggatgatatc ccagccgttt acatcaggca gcatgatatc caggatgact aaatcatact

143461 cggctgtcat ggcaagatga tatccggtca gaccattatc agcgtgatcc acgacgaacc

143521 ccgcctctgt cagccccttg ctgagatatt cacctgtttt aatttcgtct tcaacgatta

143581 atattttcat catgctcccc ggctggctgc taatgtcatt ttattgcgcc cacgatcgtt

143641 atcaacggat tacagcaaaa atgacaacat tgtcggtgat gctgccaact tactgattta

143701 gtgtatgatg gtgtttttga ggtgctccag tggcttctgt ttctatcagc tgtccctcct

143761 gttcagctac tgacggggtg gtgcgtaacg gcaaaagcac cgccggacat cagcgctatc

143821 tctgctctca ctgccgtaaa acatggcaac tgcagttcac ttacaccgct tctcaacccg

143881 gtacgcacca gaaaatcatt gatatggcca tgaatggcgt tggatgccgg gcaaccgccc

143941 gcattatggg cgttggcctc aacacgattt tccgccattt aaaaaactca ggccgcagtc

144001 ggtaacctcg cgcatacagc cgggcagtga cgtcatcgtc tgcgcggaaa tggacgaaca

144061 gtggggatac gtcggggcta aatcgcgcca gcgctggctg ttttacgcgt atgacaggct

144121 ccggaagacg gttgttgcgc acgtattcgg tgaacgcact atggcgacgc tggggcgtct

144181 tatgagcctg ctgtcaccct ttgacgtggt gatatggatg acggatggct ggccgctgta

144241 tgaatcccgc ctgaagggaa agctgcacgt aatcagcaag cgatatacgc agcgaattga

144301 gcggcataac ctgaatctga ggcagcacct ggcacggctg ggacggaagt cgctgtcgtt

144361 ctcaaaatcg gtggagctgc atgacaaagt catcgggcat tatctgaaca taaaacacta

144421 tcaataagtt ggagtcatta ccacattgtc attatcctgt cacccggcaa acagagagcg

144481 ttcggtaaag tatccctatc aatactctgg atttcgtttg aaccatttac caggtctgcc

144541 tgtacgagaa gcgttatgtt caaattaaaa ttactcagca tcagtaccat attcatcctg

144601 gctggttgtg tttctctggc acctgaatat cagcgtccgc cagctccggt tccccagcag

144661 ttttcattgt ctaaaaacag tctgacgcct gcggtaaaca gctatcagga tacgggctgg

144721 cgaaactttt ttgtcgatcc ccaggtcagc aggctgatcg gtgaagccct gaataataac

144781 cgtgatttga gaatggctgc cctgaaggtt gaagaggccc gggcccagtt caacgtcacg

144841 gatgcagatc gttatcccca actgaatgcc tcatccggga tcacatacaa cggtggtctg

144901 aaaggtgaca agccgaccac acaggagtac gacgcgggtc tggagctcag ctatgagctc

144961 gatttttttg gcaaacttaa gaacatgagt gaggctgatc gccagaacta ctttgccagc

145021 gaagaagccc gtcgtgccgt acacatcctg ctggtctcca acgtttcaca gagctatttc

145081 agccagcaac tggcgtacga acaactccgt attgcgcggg aaacgctgaa aaattatgaa

145141 cagtcctatg ctttcgttga gcaacagctc gtgaccggga gtacgaacgt tctggcactt

145201 gaacaggcga gaggacaaat cgaaagtacc cgcgccgaaa tagccaaacg agaaggcgat

145261 ctggctcagg caaacaatgc cctgcaactg gtgctgggaa cgtaccgcgc acttccgtca

145321 gaaaaaggga tgaaaggcgg ggagatcgca ccagtaaaat tgccaccaaa tctatcttca

145381 caaattttgc tgcagcgacc ggatattatg gaagcggaat atcagctgaa agcggctgat

145441 gccaatattg gcgcagcgcg agcggccttt ttcccctcca ttaccctgac cagtggtctt

145501 tccgcaagca gtacggagct gtcaagcctg tttacgtcag gaagtggaat gtggaatttt

145561 atccctaaaa ttgaaattcc tatttttaat gctggcagga ataaagccaa tctgaagctg

145621 gctgaaattc gccagcaaca atcggtggtt aattacgaac aaaaaattca gtcagccttt

145681 aaggatgttt ccgacacgct tgcgctgcgc gacagtctta gccagcaact tgagtcacag

145741 cagcgttatc ttgattcact tcagataact ctccagcgtg ccagaggatt atatgcaagt

145801 ggtgctgtca gttacatcga agtgctggat gcagaacgtt ccctcttcgc tacgcagcaa

145861 accattctcg atcttaccta ttcccgacag gttaacgaaa ttaatctgtt taccgcgctg

145921 ggtggcggtt gggtagagta aatttattta attaatcagg aaattaaaaa tgcgtaattc

145981 acttaaagcc gttttatttg gtgccttctc tgtcatgttt tctgccggtc ttcatgctga

146041 aacacatcag catggcgata tgaatgctgc cagtgatgct tcggtacagc aggttatcaa

146101 gggcaccggt gtcgttaaag acattgatat gaatagtaaa aagattacca tttcgcacga

146161 agcaatccct gctgtgggct ggcctgcaat gaccatgcgc ttcacttttg ttaatgcaga

146221 cgacgctatc aatgccctga aaaccggcaa ccatgtcgat ttctcgttta ttcagcaggg

146281 caatatctcc ttactcaaaa gcattaacgt tacgcaatcc tgattatcag tccggagcga

146341 atacatccag tgcgcctgaa cattcattaa gggattactg tgaatgaatg atcgggcgca

146401 tatgccaggt gttttgattt ttcagcgaga aattgtatgg cttctttaaa gataaaatat

146461 gctgcaataa ttatcagcag cctcatagca ggggggctga tatcggttac tgcctggcag

146521 tatgtaaact catcacaaaa aacagtacaa accgaacaaa aggcaccgga gcgaaaggta

146581 cttttctggt atgacccgat gaaaccggat accaaatttg ataaacccgg aaaatctccc

146641 tttatggata tggacctggt gccgaaatat gctgatgaaa gtggcgataa aagcagtggc

146701 gggatccgta tcgatccaac gcaggttcag aatctgggat taaaaacgca aaaagtcacg

146761 cgaggaatgc tgaattattc ccagacaatc ccggctaatg tcagttacaa cgagtatcag

146821 tttgtcattg tgcaggcgcg ctctgacggt ttcgtcgaaa aagtgtatcc cctgacgatt

146881 ggcgatcatg tgaagaaagg cactccgctt atcgatatca ccattcctga atgggttgag

146941 gcacaaagtg agttcctgct gttatccggt acaggcggta cgtcaaccca gataaaaggg

147001 gttctggagc gacttcgtct ggctggtatg ccggaagagg atattcaaag gctgcgttca

147061 acccgcacaa tccagacccg ttttaccatt aaagcaccta ttgatggtgt cattactgcg

147121 tttgacctgc gcaccggcat gaatatttcg aaagataaag tagtggctca gattcagggg

147181 atggacccgg tctggatcag cgctgcagtg ccagaatcta tcgcatatct gctgaaagat

147241 acgtcgcagt ttgaaatttc ggtaccggct tatccggata aaacattcca tgtcgaaaaa

147301 tggaacattc ttcccagcgt ggatcagaca acccgtacgc ttcaggtccg tctccaggtt

147361 tctaataagg atgagtttct caagccgggc atgaatgcct atctgaaact gaataccaag

147421 agccaggaga tgctgctgat accaagccag gccgttatcg ataccggcaa agaacagcgc

147481 gtgattactg ttgatgatga aggcaagttt gtgccgaaac agatccacgt tctgcatgaa

147541 tcacagcaac agtccggcat cggttccggc ctgaatgaag gcgataccgt ggtggtcagt

147601 ggcctgttcc tcattgactc cgaagccaat attacgggcg cgctggaacg tatgcgccac

147661 cctgaaaaaa cagaaagcag tatgccagca atgtctgacc agcctgtaaa tatgcattca

147721 gggcactgag gagacgacga tgattgaatg gattatccgg cgctctgtcg ccaaccgttt

147781 cctggtcatg atgggggccc tgtttctcag catctggggt acatggacga ttattaacac

147841 gccggtcgat gccttgcctg acctgtcaga tgtacaggtc attatcaaaa ccagctatcc

147901 cggccaggcc ccgcagattg tagaaaacca ggtcacctat ccacttacca ccaccatgct

147961 gtccgtacct ggcgcaaaaa ccgtgcgtgg tttttcacag ttcggggatt cgtatgtgta

148021 tgtcattttt gaagacggca ccgatctgta ctgggcccgt tcccgcgtgc tggaatatct

148081 gaatcaggtt cagggaaaac tgcctgccgg tgtgagttct gaaatcggtc ctgatgccac

148141 gggtgtgggc tggatttttg aatatgccct tgtcgatcgc aacggaaaac acgacctttc

148201 agaactgcgc tctctgcagg actggttcct gaaatttgag ctgaaaacca tcccgaacgt

148261 ggctgaggtc gcttcggttg gcggcgtggt gaaacagtac cagattcagg tcaatccggt

148321 aaaactgtcc cagtacggta tcagcctgcc cgaagtgaaa caggcacttg aatcgtctaa

148381 ccaggaggcc ggtggctcat ccgttgaaat ggccgaagcg gagtatatgg tccgtgccag

148441 cggttatctt cagagcattg atgattttaa taacatcgtc ctgaaaacag gtgagaacgg

148501 cgtgccggtt tatctgcggg atgttgcccg cgtgcagacc gggcccgaaa tgaggcgtgg

148561 tattgccgag ctgaacggcc agggagaagt cgctggcggc gtggtgatcc tgcggtcggg

148621 taaaaatgcg cgcgacgtta tcacggcagt gagggataaa ctggagacgc tgaaggccag

148681 cctgccggaa ggcgttgaaa tcgtgaccac ctacgatcgc agccagttaa tcgaccgggc

148741 gattgataac ctcagttaca agcttcttga agagtttatc gttgttgctg tcgtttgcgc

148801 actgttcctc tggcatgtcc gttctgccct ggtggcgatt atttctctgc cacttggcct

148861 gtgtatcgcc tttatcgtca tgcacttcca gggactgaac gccaatatca tgtcgctggg

148921 agggatagcg attgccgtcg gtgcgatggt ggatgccgcc attgtgatga ttgaaaatgc

148981 gcacaaacgg cttgaggagt gggatcatca gcatccgggt gagcagattg acaacgccac

149041 ccgctggaag gtgattaccg acgcctccgt ggaagtggga cccgcgttgt tcattagcct

149101 gctgatcatc accctgtcct ttattcctat ctttaccctg gaagggcagg aaggtcgtct

149161 gtttggcccg ctggcattca cgaaaacgta ctccatggcg ggagcggccg cactggccat

149221 catcgtcatt cctattctga tgggattctg gatccggggg aaaattcctg ccgagacaag

149281 taaccccctg aaccgggtgc tgatcaaagc gtatcatcct ttgctgctgc gggtcctcca

149341 ctggccaaaa acaaccctgc tggttgcggc cttgtccatt ttcacggtta tctggccact

149401 gagtcaggtg ggcggtgaat ttctgccgaa gattaacgag ggcgatctgc tgtatatgcc

149461 gtcgaccttg cctggcgtct ctccggcaga agctgcagcg ctcctgcaga caacagacaa

149521 gttaatcaaa agcgttcctg aagtggcttc tgtatttggc aagaccggta aagcagagac

149581 cgcaacggat tccgcgccgc tcgaaatggt ggaaaccacg atccagctca aacctgagga

149641 tcagtggcgt cccggcatga caattgacaa gattattgat gaactcgaca ggacagtccg

149701 tttaccgggt ctggcaaacc tctgggtgcc gcctatccgt aaccgtattg atatgctctc

149761 aaccgggatc aaaagcccga taggtatcaa agtgtccggg actgttctgt ccgatatcga

149821 cgcgacggcg cagagtatcg aggcggtagc caaaaccgtg cctggcgtgg tgtctgtcct

149881 ggctgagcga cttgagggcg ggcgctacat cgatatcgat atcaaccggg agaaagcttc

149941 ccggtacggg atgactgtgg gcgacgtcca gctgttcgtc tcttcagcaa tcggtggggc

150001 tatggtgggt gagacggttg aaggcgtggc ccggtaccct attaacattc gctatccgca

150061 ggattaccgt aacagtccgc aggcgttgag agagatgcca atcctgaccc caatgaagca

150121 gcaaattacg ctgggcgatg ttgccgatat taaggtcgtt tccggaccaa ccatgctgaa

150181 aaccgaaaat gcccggccag ccagctggat ttatgttgat gcccgcggca gggacatggt

150241 gtcggtggtg aacgatatta agacggccat cagtgagaaa gtgaaactga gaccggggac

150301 cagtgtggca ttctcaggac agtttgaatt actcgagcac gccaataaga aattaaaact

150361 gatggtaccg atgacggtga tgatcatctt catcctgtta tatctggcat tccgccgggc

150421 tgatgaggcc ttacttattc tgatgagcct gccgtttgcc ctggttggcg ggatatggtt

150481 cctgtactgg cagggcttcc atatgtcagt ggcgaccgga accgggttta tcgccctggc

150541 cggggtggca gcagagtttg gcgtggtcat gctgatgtat ctgcgtcatg ccattgaagc

150601 gcacccggaa ttgtcccgta aagagacgtt cacaccggaa ggccttgatg aagccctcta

150661 tcatggtgcc gtactgcgtg tccggccgaa agccatgacc gtggcggtga tcattgcggg

150721 tctgctgcca atactctggg gaaccggtgc aggttcagaa gtcatgagcc gtatcgcggc

150781 acccatgatt ggtgggatga tcacggctcc gctgctgtcc ctgttcatta ttcctgccgc

150841 ctacaaatta atctggctgc gcagacataa aaaaagcgtg tcctgaacct gaaagggcac

150901 cccctgtggg tgtccttctt tactgattca ccctgacgtc agggtttata tcgataatat

150961 acagaggtga gtatgaaaaa agtggttcta atggcgctgg ctctcggcct ttcactaccc

151021 gcgatggcga gtgaaaaagt gattgatatg tacaaatctg aaaactgtgg ctgttgttcc

151081 ctgtggggca aagcgatgga aaaagacggg tttgaagtac gaactcacgt catgaatgat

151141 caggcgctgt cagccctgaa agaaaagcat gctattcctg caggactacg aagttgtcat

151201 accgcggttg ccggtaattt gatcattgaa ggccatgtgc ctgcgacaac gatacataag

151261 gcaatgcagt ctggttcggg tatatacggt ctcgccaccc ccggtatgcc agcaggaagt

151321 cctggaatgg agatgggagc ccgaaaagag gcttacgatg ttatcgcatt ctcaccggat

151381 ggaagtaaaa aagtcttcca gcgaatcgaa tagtcagcgg aacggctgat aacgggacgc

151441 cggtagcagg cactcctgtg ccggcgatat tcgtggtaat cgcatccatg acataccctg

151501 aagacagaag atgcttcggt atgcataagg agagttactg tgaaaaatga caatgcagtg

151561 caacacaaca accagactgc ttctgagcag acattatccc cggacgaggg ccacgtattg

151621 cataaggtga gagatcccgt gtgcgggatg gccatcctgc ccgacagggc gcacagcagc

151681 attcgatacc aggaccacca actttatttc tgctccgcca gctgtgagag taaatttaaa

151741 gcccatcccg atcgttatct taccgaagat gccagtgaac attcccatca ccatcaccac

151801 gatcatcacg aagtcagccc tgatcagata aaacagcctc acaaccaggc ggaaaaagag

151861 aattctgaag gtgtgtggac atgtccgatg cacccggaga tacgccgcag tggtcccgga

151921 agctgtcctg tctgtggaat ggcactggag ccgctcgtag ctacggcatc cacggggccg

151981 agtgatgaac ttcacgacat gacaagacgc ttctggctgg ggttgttgct ggcgtttccg

152041 gttctggtac tcgaaatggg atctcatctg tttcccgact tgaggaatac agtaccgcca

152101 cagtacaaca catggctgca gttgcttctg gcctcccctg tcgtgttgtg gtgtggctgg

152161 ccattcttcg cccgggccgg aatgtcgtta cgtaaccgct ccctgaatat gtttaccctt

152221 gttgcaatgg ggaccggcgt agcctgggtt tacagcgtca ttgcaaccgt cttcccctcc

152281 tggtttcctg catcgttcag aaacatggat ggcctggtgg ccgtttattt tgaagccgca

152341 gcagttatta cggtgcttgt tctgctggga caggttcttg agctgcgggc acgggaacaa

152401 acctcaggcg ccattactgc gcttctgaac cttgccccca aaaccgccag acggctggat

152461 catgacggtc atgaaacgga tattaatgcg gaagatgtcc tgcctggcga taagctccgc

152521 atcagacctg gagagagtat tccggtcgac ggtatcgtga tcgaaggcaa aacaaccgtt

152581 gatgaatcga tggtgaccgg ggagtctatg ccggttacca aaacgaaggg tgaccctgtc

152641 attgggggga cgattaatca gacaggtagt cttatcatcc gtgcagagaa agtcggtgat

152701 gaaacgatgc tctcacgaat tgttcagatg gtcgctgatg cacagcgttc gcgtgccccc

152761 atccagagaa tggcagacag cgtttcaggc tggtttgttc ctctggtgat acttatcgcg

152821 gttgttgctt tcttgatctg gtctgtctgg gggcccgagc ccaggatggc gcacggtctc

152881 attgcggctg tgtcggtcct gattattgcc tgtccctgcg cgctgggact ggccacgccg

152941 atgtcgataa tggtgggggt gggcaaaggc gcccaggccg gggtgttaat caagaatgcc

153001 gaagcccttg agcgtcttga aaaagtggac acgctggttg tcgacaaaac aggcacgctc

153061 acggaaggtt cgcctacggt gacagggatt atcagtctca gtccgggtgg ggaaatatct

153121 cttttacgtg taacagctgc agtggaaaaa ggttcgcagc atccgttggg tatggctgtt

153181 gtcagagccg cgcatgaaaa ggggatcgtg atacctgccg tcagtaattt caatgccccg

153241 tcggggaaag gtgtctcagg cgatgtcgaa ggtcaacggg ttgttattgg taatgaactg

153301 gctatgcagg aaaacagtat cgttattgat aatcaaaagg ccgttgcgga tacgttgcgg

153361 atggaaggcg ctaccgttat ctatgtggcc acagacggga accttgcagg cctgatagct

153421 atctcggatc ccgtgaaagc aaccacgccg gatgcgctta aagctttgcg tcaggcgggg

153481 atccgcattg ttatgctcac cggggataac cagcttactg ctgaagcagt cgcacggaaa

153541 ctgggaatag atgaggttga agccgggatt ctgccggatg gcaaaaaagc agtgataacc

153601 cgactgaaag cgtctggcca tgtggttgcg atggccggag acggtgtgaa tgatgccccg

153661 gcgctggcag cggctgacgt gggtatagcc atgggaacgg gtacagatgt ggcaatagaa

153721 agtgctggcg ttacccttct caaaggcgat ttgatgatac tcaacagggc ccgtcatctg

153781 tcagaaatca ccatgaaaaa tatccggcag aatctgtttt ttgcgtttat ctacaatgct

153841 cttggggtgc ctgtagctgc aggtctgctt tatcctgtgt atggaatact gctgtcgcca

153901 gttattgcgg cggcggctat ggccctttcc tccgttagcg tcattgcaaa tgcgttgcgt

153961 ctgaaaagtg tcaggctcgg gaaataaccc tgaatgaagg gtctgttact aacagaagga

154021 gtccggtatg aaaagtacca cctatgcgct tattgctgtc gccgcgattg cggcatttgc

154081 cctcctgcgc gaacactggt cacatgtggc aggttactgg ccatatctgt tattgctggt

154141 ctgcccgcta atgcatcttt tccacggcca cggagggcat ggagatcatc aacatcaagg

154201 aagtgaaaac gataaaaaaa attaatccgg cagacggggc cgcgtcgcgg ccccgttatc

154261 attccaggta tcgttcgtag tctctggcat gcgcaaaggc atgctgttca agtttgttat

154321 cagcgggtgc cgctgcccgg aacgccagtg agttaacagg attgttattg atgaccagct

154381 cgtaatgcag atgaggaccg gatgaacgtc cgctgttacc ggataacgca atagcgtctc

154441 cccgggtaac cctggcccct ttagtaacga gtattttatt gaggtggaga tagcgagttt

154501 taacaccggc ttttcccgtt acttcaacaa aatatcccat ggtactgttg tattcggccc

154561 gggtgatttt tccgtcgatg acgctgacta ttttcgtgtt catgggcatg gaataatcaa

154621 tgccattatg gggactcact tttcccgata ccgggttaag tcttgcagga ttgaaaggcg

154681 aactgagtct tgctgtggcc ggtaacggat aatcgagact gcctttcccg gaagtatcgg

154741 aaaggttata gaacttttta tctgatatac gatacgccgt gtaattaaat gaaccggacg

154801 taaatttata ggccacgaca cgtgattttc ccgctttctt ttgcagtacg agttttaatg

154861 attcattttt tttcaaatgc cgcagattaa accgggaagg caaggagcgc tgaagagtag

154921 cgatctcgtt cgattccagc cccgagcggg tggctgaaag gtaggcattt tcttttacga

154981 catcggtaga atacatttac tggaattcgc cgttagcatg aacactgcgg cgtatgctag

155041 gggttttcag gaggcgtgtc atctgtcagt taatcgggag caccgttgat ggtcgtcatt

155101 tttgtaacat atcttgtttc ccgtttgctc ctgaagctct gggaactgta tgaccagccc

155161 gacggtgatg attaacggga ctgaaacagg ttacggcaga gcaatatggg gctcatgtcc

155221 tgctacgtaa cccgtcagta aagccctgct gcgcacctga cgctaagcac taacccgcct

155281 gcagttacct ggtcgaatac agcccgcgaa gctttcttgc ctgcgtctga tgtgcttccg

155341 caccggcatt attgacctgc tcatgcacga gagcggcttt ttctccggca ttcagttcgt

155401 taaaagaaga agacgaggtc tttgaatttg catcactgcc ggacagcatt tttttatgtt

155461 cctcaatcat tttctgatgc gcataggacg cgctgttgtt cataaatgaa tgagcaacaa

155521 tggccctttc atgttcattc atttcagaga atgagggcgt gttgttttta ttaaccctgt

155581 ccggtaagtt ttcatgcgtc gaggagttca catgactgac ggctgaggca ttattaacaa

155641 atcgatgtgc ttcatgggca atatcactgg actgagcaaa agctgcccca caaaataaag

155701 ctgtaaacgc agtggtcgtg attaatatat tcatgtgtaa ttaccttctg aggtacataa

155761 aagatgtcct tatgatcata tataaaaaaa tcaacctgtg gagaagatga cgtaaatgta

155821 atacagccac gtacattaca cgattgtaat gaatttgttt cttaaggtgt gctagattca

155881 tttcattgta agtggatgaa ccagtaattt aatttaaatc ggttctcgaa ttctgtcagt

155941 aaccatactt taaataaggg aatgcgcatg ctgttgaaaa cgtctcgacg aactttcctg

156001 aaggggttaa ccctctctgg cgtagccgga agtcttggcg tatggagttt caatgcgcgt

156061 tccagtctga gcctgccagt tgccgcatcc ctgcagggta ctcagtttga cctgaccatt

156121 ggtgaaacgg ccgtcaatat cacgggcagt gagcgtcagg ccaaaacaat caatggaggc

156181 ctgccggggc ccgttcttcg ctggaaagaa ggtgacacca ttaccctgaa ggtcaaaaac

156241 cgtcttaatg aacagacgtc cattcactgg cacggcatta ttcttccggc caatatggat

156301 ggtgttccgg ggctgagttt tatgggcata gagcctgatg atacctacgt ttacaccttt

156361 aaggttaagc agaacgggac ttactggtac cacagccatt ccggtctgca ggaacaggag

156421 ggggtatacg gtgccattat catcgatgcc agggagccag aaccgtttgc ttacgatcgt

156481 gagcatgtgg tcatgttgtc tgactggacc gatgaaaatc ctcacagcct gctgaaaaaa

156541 ttaaaaaaac agtcggatta ctacaatttc aataaaccaa ccgttggctc ttttttccgc

156601 gacgtgaata ccagggggct gtcagccacc attgccgatc ggaaaatgtg ggctgaaatg

156661 aaaatgaatc cgactgacct cgcggatgtc agtggctaca cctacaccta tctcatgaac

156721 gggcaggccc cgctgaaaaa ctggaccgga ctgttccgtc ccggtgaaaa gatacgctta

156781 cggtttatca acggctcggc aatgacctat ttcgatatcc gtatccccgg gctgaaaatg

156841 acggtcgtgg ctgcagatgg ccagtatgta aacccggtta ccgttgacga attcaggatt

156901 gccgttgccg aaacctatga tgtcattgtg gagcctcagg gtgaggccta taccatcttc

156961 gcacaatcca tggacaggac cggttacgct cgagggacac tggccacgag agaagggtta

157021 agtgctgccg ttccccccct cgatccccgt cctctgttga ccatggaaga tatgggtatg

157081 gggggaatgg gacatgatat ggcaggaatg gaccacagcc agatgggagg catggataac

157141 agcggagaga tgatgtctat ggacggtgct gaccttccgg atagcgggac atcctccgcg

157201 cccatggatc acagcagcat ggccggtatg gatcattccc ggatggccgg aatgccgggt

157261 atgcaaagtc atcctgcgtc agaaacggat aacccactgg ttgatatgca ggcgatgagc

157321 gtctctccga aattaaatga tccgggtatt ggtcttcgaa ataacggaag aaaggttctc

157381 acgtacgcgg atttgaaaag ccgctttgag gatcctgacg gacgtgaacc tggccgtacc

157441 atagaactgc atttaaccgg ccacatggaa aagtttgcct ggtcatttaa cggaatcaag

157501 ttttcagatg ccgcaccggt gctgctgaaa tacggtgagc ggctcaggat cacgctgatc

157561 aacgatacca tgatgactca ccccattcac ctgcatggta tgtggagcga tctggaagat

157621 gaaaacggta atttcatggt tcgtaaacac acaatagatg ttccccctgg tacaaaacgc

157681 agttacagag tgacagcaga tgcgcttggc cgctgggcgt atcactgcca tttgctctat

157741 cacatggaaa tgggaatgtt tcgtgaagtc cgggtggagg aatgatgcga atgaagagaa

157801 atttgaaggc catacctgtt ctggtcgccg gtttgtttac ctcacagctt tctattgcgg

157861 cgggctccgt ctctgcagat ccccacgccg ggcacgacat gtctgccatg cagatgccag

157921 cagatgagaa tttcactgag atgacgtcaa tggagcccat tgtaactgag agcagaacgc

157981 caattccgcc tgttaccgat gccgaccgga aggctgcatt cggcaattta caggggcatg

158041 cgattcacga cagtgcgatt aattatctgg ttctgctgga tcaactggaa tggcaacggt

158101 cggataacac caacaatttc agctggagtg ttaacagctg gattggaggc gacacagatc

158161 ggatttggct aaagagtgaa ggtgaacgaa gcaatgggga aacggaggcg gctgaagcgc

158221 agttactctg gggacatgcg gttggcccat ggtgggattt ggttgcgggt gtcaggcagg

158281 atttcagacc tgcttctgcc cggacctggg ctgctgtcgg ttttcagggg ctggcactct

158341 ataattttga gtctgaaatt acgggttttg tcagtaatgg cggaaaagca gcccttcgtc

158401 tgggaggaga atacgacgtt ttactgacta accggctcat actccagcca tcctatgagg

158461 tgaatttcta cagtcaggat gatgaatcgc ggggtcgcgg caggggactg actgacacag

158521 agctggggct ccggctgcgc tatgaaatac gccgtgagtt tgcaccctat ataggcgttt

158581 cctggaatca actttacggg aaaacatccg atatggcgaa aagagaaggt gagaaagacc

158641 atcaggtagt attcctggcg ggagccagaa tctggtttta acgcactgat ataaaacact

158701 caactaaaca ggtaaataaa atgtcgattt taaataaagc cattcttaca ggtggcctcg

158761 ttatgggcgt tgctttctct gctatggccc atccggaatt aaaaagctct gtgccacagg

158821 ctgattcagc cgtagcggcc ccggaaaaga ttcagcttaa tttctcggaa aatctgaccg

158881 tgaaattctc aggtgcaaaa ttaacgatga cgggtatgaa aggcatgtca tcacattctc

158941 cgatgccggt cgcggcaaaa gtggcgccag gcgctgaccc taaatcgatg gtcattattc

159001 cgcgagagcc tttacccgct ggcacttatc gtgttgactg gcgcgcggtt tcttcagata

159061 cgcaccctat taccggtaat tacaccttta cagtgaagta atattatgaa cgacctgatt

159121 atgattgtta ttcgttttct tctttatctg gatttgatgg taatatttgg attgccattt

159181 tttcagatat atggaataag cggtgtcaga catgaaacct ataacctgac taatttcagg

159241 tcgtttataa cctttgctgt tgttacaggc atcattctta ctggcattaa tatgctcctg

159301 gtatctaatg ccatgagtgg agtaactgac ctcagagaat tatccatcca tgttatcgag

159361 atggtgatag aagaaactga tgtgggtatt agctggattg tcagactctg tgccctgttt

159421 accacactcg gtgctttgtt cctttacact aataagagag tattgtcctg cctgctgatg

159481 acgatgagtg ggggcgtggc gctggctaca cttgcctggg gaggacacgc cgttatgcat

159541 gacggtctgc attactatct ccatttactg agcgatctga cccatctcgg cgctgcaggt

159601 gcctggacag gtgctctggt tgcatttgct atcctgctga tgcgcagaaa cgagcataat

159661 gcacagagcg tcattgtgat atctgactcc ctggcaaaat ttgccacggc aggaacggtg

159721 attgttgtag ccctgatcct gagtgcgctg gtcaactatc tgtatattgc tgagggtaac

159781 ttaactccct tattcaacag ttcctggggg aggatattgc ttgccaagac ggctctgttt

159841 gttctgatgc ttcttctggc tgcagcaaac cggtttcacc tgggtccccg gcttgaagtt

159901 atggtcaggg aagggaatta tgatcgcagc gttgccctga tgcgaaacag catcctgaca

159961 gaattcgttg ttgcgattat cattctgggc gccgtagcgt ggcttggaat gcttgctccg

160021 tctcagatca gctaggggac agccaaagct catgcgtgag atttttactt tcatatcagc

160081 gagttgacca tgcagcgtat tttaatcgtt gaagacgaac aaaaaacagg tcgttacctg

160141 cagcagggac tggttgagga aggctatcag gccgatctct ttaataatgg ccgcgatggt

160201 ctcggggccg cgtcgaaggg acagtatgat ttgataatac tggacgtgat gctgcctttc

160261 ctcgacgggt ggcaaatcat cagcgcactg agggagtccg ggcacgaaga accggtcctg

160321 tttttaaccg caaaggacaa cgtgcgggac aaagtgaaag gactggagct tggcgcagat

160381 gactacctga ttaagccctt tgattttacg gagctggttg cacgtgtaag aaccctactg

160441 cgccgggcac gctcgcaggc cgcaacagtc tgcaccatcg ccgatatgac cgttgatatg

160501 gtgcgccgga ccgtgatccg ttcggggaag aagatccatc tcaccggtaa agaatacgtt

160561 ctgcttgagt tgctgctgca acgcaccgga gaagtgttac ccaggagtct tatctcgtcc

160621 ctggtctgga acatgaattt tgacagtgat acgaatgtga ttgatgtcgc cgtgagacgt

160681 ctgagaagta aaattgatga tgactttgag ccaaaactga tccataccgt tcgcggtgcc

160741 ggatatgtcc tggagatcag agaagagtga ggttcaaaat ttccctgacc acacgcctga

160801 gcctgatttt ttctgcggtg atgcttacgg tatggtggtt atcaagtttt atcctgatta

160861 gcaccctgaa tggctatttc gataatcagg accgcgattt tctgacaggt aaacttcagc

160921 tcaccgaaga gtttcttaaa acagagacgt tcaggaacaa aacggatatt aagtcattat

160981 cagaaaaaat aaacgatgcg atggtggggc acaatggctt attcatttct ataaaaaaca

161041 tggaaaatga aaaaattgtt gaactctatg ccaaaaattc tgttgttcca gcggtcctgc

161101 ttaataagtc gggtgatatt ctcgactata tgatccagac ggaagaaaat aacaccgtgt

161161 accgcagtat ctcgcggcgg gttgccgtga cgccggaaca gggtaaaagc aaacatgtca

161221 tcattacggt tgccacggat actgggtatc acaccctgtt tatggacaaa ctcagtacct

161281 ggctgttctg gttcaatatc ggtctggtct ttatttctgt ttttctgggc tggctgacca

161341 cacgtattgg tctgaaaccg ctacgggaaa tgaccagtct ggcttcctcc atgaccgtac

161401 acagcctgga tcagcgtcta aatcccgatc tggctccgcc ggaaatctct gagaccatgc

161461 aggagttcaa taatatgttt gatcgcctgg agggggcatt ccggaaactg tcagatttct

161521 cgtctgacat cgcgcatgag ctgcgcacac cagtcagtaa tctgatgatg cagacgcagt

161581 ttgcactggc taaggaaagg gatgtttcgc attaccgcga aattttattc gctaacctgg

161641 aagaactgaa aaggttgtca cgaatgacca gtgacatgct ttttctggca cgttcagagc

161701 atggtctgct gcggctggat aaacatgatg tggatctggc agccgaactg aatgaattac

161761 gtgagttgtt cgagcccctg gcagacgaaa caggaaagac aatcacggtt gaaggagagg

161821 gcgttgttgc cggagacagc gatatgctcc gacgtgcttt cagtaacctg ctttccaatg

161881 caatcaagta ttctcccgat aacacctgta cagcgataca cattgagcgt gacagtgact

161941 gtgtgaacgt gatgattacg aatacgatgt ccggccaggt tcccgctaat ctggaacgtt

162001 tgtttgaccg gttctatcgc gcagactcat caaggttcta caacacggaa ggcgcggggc

162061 tgggattatc aattacaagg tcgatcattc atgctcacgg cggcgagctg tcagcagaac

162121 agcaggggcg ggaaattgtg ttcagtgtgc gtctgttaat ggattaatac cgttattcag

162181 gagaaacccg gaaggtgaca aaaatgtcat cgttcagtca cgcgataaac agaggcggtt

162241 ttttataatc agccataaat caggacagcg tgataattca atcgcccggt tcctggcgtg

162301 atgatcaacc agccctgaga tcaaatgctt tctctgttat aagccgttga ttgtttgggt

162361 atgaaaacac cggagaccca accatgaaaa agatccttgt atcatttgtt gccattatgg

162421 ctgtcgcttc atccgccatg gctgcagaga caatgaacat gcatgaccag gtaaataatg

162481 cccaggcacc cgcccatcag atgcagtcaa cctctgaaaa aagcgctgtt cagggagaca

162541 gtatgacaat gatggatatg agcggtcacg atcaggctgc aatgacccat gaaatgatgc

162601 aaaacggcaa cgcttctgcc catcaggaca tggctgaaat gcataaaaaa atgatgaaat

162661 ccaagccagc agcttctaac gaaacagcaa aatcattttc cgaaatgaac gagcatgaga

162721 aagccgctgt tgtacacgag aaggcgaata atggtcagtc ttcagtgatt catcagcagc

162781 aggctgaaaa gcatcgcagc cagatcaccc agaattaacc cgcagctcca cttgtcagac

162841 cctcatttga cgccgaagtc actggcttac gctcccgccg ggagcgtttt ttttcgtaat

162901 taataagtgt catcaaaagc atccatcacc attatcataa ttggccctga tcaaataaaa

162961 tttaacttat gttgcgcgag aattcagttc atgaaaaaaa tatcagtatc atattgtttt

163021 taattattat tttcgttccc ggttatatcc tatatccagg gttcatgccg cgccgtatcc

163081 tgagactggc tgagaatacg aatgatcata accaccgaat gtgatgacac aaaatagatc

163141 atgtgctgtt ccaccggcag cgagcagata ttgtctccca gctccgcgcg atgcgtgcct

163201 atgtcatgca tggccagaac gtcaaacacc gctgctatac tgttaccaac ggtgcaaaac

163261 tgggtcttcc gcaacttagg tggagttgtt taagccagag ggctcatgac tcgtcgtcgc

163321 agtaactcga agccagctcg accgtacatc tgccgtttca gcatcttcag gcggtttaca

163381 tggccttcca caacgccatt actccatctg ctgcttatcg cttcgtgtat tgctgttgca

163441 tccgcttcca taccggccgc aacacgctga aggtcaatga gcccactttg actgacgtca

163501 gagaaccatt gatttaactg cgatttattc ttcgttttca acattcgata gaagtcgagc

163561 gacagctgtt gcgccatttt cagttgcggt tctttctggc acatcgattc gatgaagcgg

163621 gatgcatagt tttcttctcc tctgatcatt cgccagggca ttaaccagcg gcttactctg

163681 gatgctgagg ggagtcgaac tggggcgata acgggactgt taacctgctt acgccactta

163741 gccacagcat ccctgaccgt cgtttcgctc ccggtgaagc cagcatttac catctcccgc

163801 catagctgcc cggcattatg gttaccgttt atccgttgtt cttccagcca ttcctgccat

163861 ggttcaagta acccgggtct gggtggctta gttgatattt cagggaacgc ttttgactgt

163921 atccaccggc gaaccgtcac ccgggacagt ccggtgatac gcgatatttc tcttatccca

163981 caaccctttt tatgaagaga gtttacgatc agtgtgccgt acctgtggcc ccgatgccat

164041 gcccactcgt caataccaac atgcctggtc cggttctctg tttgttccgg agcttgaacc

164101 actctgcgca gtaaagtatc cgcgctgatc tgtattcctg aagctactgc agctcgtctc

164161 cctgcttcac cacctgcgat aagccccagc tgatgctgca gatttttcag caatgcagaa

164221 gactgctgct gcgggcctgc aaagggagca agcgattcag caaaaatttt acgtgaacag

164281 gaagggttac gacagtacca gtgccggatg gcgaagacta accagagggc ctggcctgag

164341 catggaagat gctgtactcg cctccgccgg caactatgaa cagaatgact gtgcgtgagg

164401 cactcaggac atgatgccgt tttacgtgtg gcgcggagat ggagggttat tccatcagta

164461 ccagtaatct gtcggcaggt ttgccacccg cgcggtagct gtaaaagtct cttaagtgag

164521 gccattttgt tatacataac ttaaaacagc ttagatcctt gttaactaaa aagtttcatc

164581 aatttttgtc cacctaagtt gcggaagacc cactttttac ccgttgttga cagcggagca

164641 caccttaacc ccgtggtaag cctgattttt tatctgatcg gggcctgctc tttcagggca

164701 accatgaatt acatcgtggc tcagttgtta ggggccggcg ctgctgcact gtgcctgaag

164761 gctgtctttg gccactccct gctggccggg gtgacccggg tccatatggg ggtatcactg

164821 tacaatgcct ttttcatcga aggtgtcatg acttttattc tgattatgag catcctaacg

164881 accagaaacc ccgcgattat cagcattgct gtttttcttg atgcttttat tggcgggccg

164941 ctgacggggg caagcatgaa cccggcccgt agcttcgggc cggcattggc catgggatac

165001 tgggataatc agtggctgta ctgggccgct ccgctgtctg gcgggcttgt agctgttgcg

165061 tgctgccagc tgtttatgcc gcaactgaag tcaccctcac cggaataaag tcccttccag

165121 gacagaatgc ctctgatgaa cagaggcatt ctttttttca gcaacccact gtccggctac

165181 gcctttccag aatgagaaca tctctccatt ttccgttcat ttcacccact ttttcccggc

165241 agccgacttc cctgaatccc tggctgcgat gtaaagcaac actggcctgg ttttccggaa

165301 agatgccagc aagtagcgtc cagaaacccg cactttccga gccctttatc agcccggaga

165361 gcaaagccct gcccgcgcct tttccctgaa aatgagtact gacataaatg ctcagctctg

165421 cgacaccgcg gtatgcatgc cggcgggaaa aaggagatag cgcagcccat cccaccactc

165481 tgttatcaac agtggcgacg tagcggcagt cctgaagatg cccttcattc cattccagcc

165541 actcaggtgc tgcggtctga aaggtggcgt tccctgttgc aattccttcc tgataaattt

165601 ccctgactgc acaccagtcc tcttcgtgca tttttctaat gatgatctct gccatttctc

165661 tgtaccgtat gtttgctgta ttcataggca gtcagaaaac gagcctgcct gggcttcccg

165721 gcggcgggac ataatgcgcc cgtcagcaaa ggatgaaggt cattttattt ttagcgggaa

165781 tcggtttcta ctgtgacgga ttcatatgca tttttccata tatattgtga cgtacctcat

165841 ttctgagagg tataaacacc gatcatgttt tctccatgcc gcgtaagtgt aaggccccgg

165901 agatggagtc atattatgga cgtggttgcg ctcactatat atttctatta ttctggaaat

165961 atagaaataa aattacgagg aatgggatgg agctgaaaat cgccgcgatg gtgctgaaag

166021 aacttggcca caccactcgt ctcgatattt acaaaacact cgttaaggcc ggacgtcagg

166081 gactgcccgt gggggaactg cagcaacatc tcgccattcc ggcctcgacc ctgagtcatc

166141 atctttcatc gctgatatcc gtctcactgg ttcgccagga aaggcagggg agaacactgt

166201 tctgccacgc ctgttatgac aaccttgctg cattgattgc ctttctgaca gaagagtgct

166261 gtgcagacga atatgccccg cctggtttta tgtcaccacc ggagaaaaaa tgattgctga

166321 actgaaaaat gttgttcctg agctgtttga tacaggtctc accggggtcc gcctgggtgt

166381 aaagacagtt gaccatcctc cccggatcct gatgttgtac ggttccgttc gagagcggtc

166441 ctacagccgt ctggccaccg aagaggctgc ccggcttctt accgccatgg gtgcggatgt

166501 ccgcatcttt aatccttccg gtctccctct gccggatgat gcagcggata cgcatcccag

166561 ggttatggaa cttcgcgaaa tggtgcgctg gtctgaaggg atggtatggt gctctcctga

166621 acgtcatggt gcgatgaccg gcattatgaa agcccagatt gactggatac cactatcaga

166681 gggcgcagta cgcccttctc agggaaaaac cctggccgtc atgcaggtct gcggaggctc

166741 ccagtctttc aatgcggtaa accagatgcg tattcttggc cgctggatgc gaatgattac

166801 tatacctaat caatcctcgg tagcgaaagc ctggcaggag tttgatgaag acggacggat

166861 gaaaccatcc ccgtactacg accgtattgt ggatgtaatg gaagagctga tgaagtttac

166921 cctgctgacc cgggagtatg cagcgtatct cgtcgatcgc tacagtgaac gtaaagagtc

166981 ggcagaagca ctttcccggc gggtgaatca gagcaaaatc tgagggctgg tatgagtgat

167041 gtaacggcat tcaacaactg catgagcgtg ttctggcaac agcatgaagc agagctctcc

167101 cgttttctgg catcaaggac aggcgatcgg gagcaggcag ccgatctgct tcaggaagtc

167161 ttcctgcgcg cccgggcctg tgcagaccgc ttctgtgaaa tggagaaccc gcgggcctgg

167221 ctgtaccgga cggcgaggaa tctgctgacg gatgagtacc gtgccgccag agacgtcgtg

167281 gtgctggaag acgagatccc gctgcctgac gccttccatg aggcggtttc aacgctggag

167341 atttgcctgc ctgaaaccct gcaggcctta cctgacgagg aaagatggct gatagaagag

167401 gccgatctga accggcgtcc ccagcagcgc ctggccgacg agctggggat cacgctgacg

167461 gcgtttaagt cgcgcctgct tcgcgcccgg aaacatctga agaagacgat gacggaactc

167521 tgccaggtgg aagtggatga tgcgtcttcc gtctgctgcc ataaaaaaat ggattaaccg

167581 cgcatctttt tcccgcctca ctcgtttacc tcagtgtaaa gccccatcat tcaacactga

167641 ggtaaacagt ccatgtcaaa aattgagatc tttgaagccg caggctgctg tgcgaccagc

167701 agcgtcgtgg tcagcgatga agccgtcaaa tggaacgcca gcgccgaatg ggcgaaaaag

167761 catggtgtga atattcagcg ttacagcttg gcaaagaacc cgcagcagtt cctgaatacg

167821 cccgtcatca gggccttcct gaacacgtca ggaatggagt cccttcccgc taccctgctt

167881 gacggccagc tggtgatggc gggcaagctc ccgtcccggg aagatatcgc ccgctgggcc

167941 ggtatttccc tcacgcagga ctggaatgaa gacagcacgc agccgcgctg ctgcagcatt

168001 ccccgtatgc cttaatcccg gaggagctca acaatgacga tgccattttt acagaatatc

168061 cccccgttta tcttcttcac cggcaaaggc ggcgtgggga aaacctctct ggcctgcgcg

168121 acggcggtat ggctggctga ccagggccgg agaacgcttc tggtcagtac cgatccggcc

168181 tccaatgtcg gtcaggtctt cagccagact gtgggccacc ggatcactgg tatccgcacc

168241 gtggaaaacc ttgcggcaat ggaagttgac ccgatggcgg ccgcgcaggc ttaccgcgat

168301 cgcgtgctcg atcctgttcg tgaactgatg cctgccgatg tcatcagcaa cattgaagaa

168361 cagctttccg gttcatgtac cacggagatc gcggcgtttg atgaatttac cgcgctgctg

168421 accagtcatg aactgcgcga aaaatatgat catatcgtct ttgatactgc accaaccgga

168481 cacaccatcc gtatgcttga gctgccgggc gcctggagcg ggtacctcga tgcacatcct

168541 gatgctgcgg cgaacctcgg ccctctggtg gggctggaga aacaacagga ccagtacgct

168601 gacgcagtga aagccctctc tgatacggca ctcacgcgac tggttctggt tgcccgcgca

168661 caggcgtcga ccctgaaaga ggtctcccat acccacgagg agctgtcggc tatcggtctg

168721 cagcatcagc accttgccat caatggcgtg ctgcctccct ttgccggtga acaggatccg

168781 ctggcgcaca gtattctggc gcgggaggaa cgggccttac gggccatgcc tgaaaacctc

168841 gcgcatcttc cccggtcgat gctgtatctg aagcctttta atctggttgg tctggaagcc

168901 ctgagggcgc tgtttacaga gagcacactt gttctgccgg atccgggagc cacgctcacg

168961 acggtcgatt taccggagct tgcctccctg gtggaggacc tcagccgagc gggaaaaggg

169021 ctggtcatga cgatgggaaa agggggcgtg gggaaaacca cggtggcggc tgcggtcgcg

169081 gtctcgctgg ccagacgcgg tcataaagtc cacctgacca cctccgatcc ggcagccacc

169141 tgtcttacac cctggatggc tcgctgccag gccttcaggt cagccgtatc gatccaaaag

169201 cagagactga gcgctatcgt cgctttgtgc tggaaaatca gggaaaaggt ctggatgaag

169261 agggactggc ggtactggag gaagatctcc gttccccctg caccgaagag attgccgtgt

169321 ttcaggcatt ctcccggatc atcaaagagg ccaacgacca ttttgtcatt atagacactg

169381 cgcccaccgg gcatacgctg ctgttgctgg atgcaaccgg ggcctatcac cgggaaatgg

169441 tgcgccagat ggggcaggct cacgatcacg tgataacgcc aatgatgcag ctgcaggatc

169501 cggagaagac cagagtcatt atcgtgacgc ttgccgaaac cacgccggta ctggaagcgg

169561 ccgggctgca gcaggactta cgccgggccg ggattgaacc ctgggcgtgg gtcatcaata

169621 acagcctcgc ggccgcgaag ccgtcctccc cgttcctggt cacccgtgcc cgtcgcgaac

169681 tgccgctgat cgacgacgtt gccgggcatt atgcacagcg cattgccctg acgccactgc

169741 tgaaggacga cccggtaggc gtggacctgc tggctgaaat ggcgggctga caggtggggc

169801 gacgccctac ctccacttaa aacatttcct tatacaggtc ataagatgaa gcgcttaatc

169861 tgccgtcgcg aattccggta gtgtgtcagt caacgtcaca ggagtcatac aatgcacatt

169921 atcaatgctg aagagcagca cattcccgcc atacggcgca tttacgccca tcatgtcctg

169981 cacggcacag gcagcttcga gacggaaccc ccggacacgc aggaaatgct tgcccgggtg

170041 aaaaatgttc agtcacgtgg atttccctgg tatatcgccc tgcaggggga gacggtcatc

170101 ggctactgtt atctctcccg ctaccgcgaa cgccatgcct accggtttac ggttgaaaat

170161 tcggtgtaca tcgatccggc ttatcagcgg caggggggcg gaaaagcctt actcgatcat

170221 gccttaacat gggcccggtc tcagggatac cgccagatga tagcggttgt gggggacagt

170281 gcgaacgtcg cgtctgtagc gctgcatctt cgcgccggat ttactgaaat cggcacgctg

170341 aaggacatcg gtttcaagca tggccgctgg ctggacacgg tgttgctgca gcgtcagctg

170401 ggaaaaggga gctgtacgct gccggacagt ccggtacccg gacgctgagg caggagaaag

170461 ggggcatcgc cccctttcgg ttatttcagc cgtttccctg cctcatctac tactttctcg

170521 ccgtcttcct tagcaaatgc gcctttttgc gcatccggaa gaatatccag taccacttcg

170581 gaagggcggc acagacgcgt tcccaacggt gtcaccacaa tcggacggtt aatcaggatc

170641 ggatgctgaa gcataaaatc gattaactga tcgtcagtaa acttatcttc ctcaagcccc

170701 agttcttcat aaggctcgac gtttttgcgc agcaaagccc ggactgaaat gcccatatct

170761 gcaatgagtt tgaccagctc atcgcgtgac ggtggatttt caaggtaatg aataacggtc

170821 ggttcattac cactgttgcg gatcatctcc agcgtattac gcgacgtgcc gcaggccggg

170881 ttgtgataaa tggtgatgtt gctcatatca gtatctcatt acaaagtgaa agagagacgt

170941 agcgccagcg ctgccagcgt tacaaacagc acaggcagag tcatgacgat cccggtgcgg

171001 aaatagtatc cccaggtgat ggtcatattc ttctgtgaaa ggacatgcag ccagagcagc

171061 gttgccaggc taccaatagg tgtaatttta ggtcccagat cgcagccaat cacgttggcg

171121 tagatcatcg cttctttgat aacgcctgtt gcggtgcttc catcgataga aagagcgcca

171181 accagcacgg taggcatgtt gttcatgatg gaagacagga aagccgtcag gaagccagta

171241 cccagcgttg cagcccagag tcctttatcc gcaagtacat tcagcacacc tgaaaggtat

171301 tcggttagcc cggcgttgcg cagaccgtag accaccaggt acatccccaa tgagaagatg

171361 acgatctgcc atggcgcacc gcgcagcact ttgccagttt taatggcatg gcctcgttta

171421 gctactgcaa acaggatcac agctccaaca gccgcaatcg cgctaacggg aataccaagc

171481 ggctcaagga caaagaaccc aaccagcaga agtattaaaa ctatccagcc ggttctgaag

171541 gttgccagat ctttaatcgc ttttgccggt gctttcagaa gagccaggtc gtaagtcggt

171601 gggatatcct tgcggaagaa caaatgcagc ataaccaacg tggcaacaat ggctgcaata

171661 tccaccggca ccattaccga cgcatactcg gtgaagccca gtccaaaggg cactgttgca

171721 aagttagcga tgaggcagcc ttttgtctta ttcaaaggcc ttacatttca aaaactctgc

171781 ttaccaggcg catttcgccc aggggatcac cataataaaa tgctgaggcc tggcctttgc

171841 gtagtgcacg catcacctca atacctttga tggtggcgta agccgtcttc atggatttaa

171901 atcccagcgt ggcgccgatt atccgtttca gtttgccatg atcgcattca atcacgttgt

171961 tccggtactt aatctgtcgg tgtttaacgt cagacgggca ccggccttcg cgtttgagca

172021 gagcaagcgc gcgaccatag gcgggcgctt tatccgtgtt gatgaatcgc gggatctgcc

172081 acttcttcac gttgttgagg attttaccca gaaaccggta tgcagctttg ctgttacgac

172141 gggaggagag ataaaaatcg acagtgcggc cccggctgtc gacggcccgg tacagatacg

172201 cccagcggcc attgaccttc acgtaggttt catccatgtg ccacgggcaa agatcggaag

172261 ggttacgcca gtaccagcgc agccgttttt ccatttcagg cgcataacgc tgaacccagc

172321 ggtaaatcgt ggagtgatcg acattcactc cgcgttcagc cagcatctcc tgcagctcac

172381 ggtaactgat gccgtatttg cagtaccagc gtacggccca cagaatgatg tcacgctgaa

172441 aatgccggcc tttgaatggg ttcatgtgca gctccatcag caaaagggga tgataagttt

172501 atcaccaccg actatttgca acagtgcccc cggagctgga taagcaacga acgtcctggc

172561 ctgcttttcg atctggcgac agggtggctt atgcaacatc gtattattct ccccggagcc

172621 actacgctga cccggttgat ttcagaggta agggaaaagg cgacgttgcg cctgtggaac

172681 aaactggcac tgataccgtc agccgaacag cgttcacagc tggagatgct gctggggcca

172741 actgattgca gccgcctgtc tttactggaa tcactgaaaa agggccctgt gaccatcagt

172801 ggtccggcgt ttaatgaagc aattgaacgc tggaaaactc tgaacgattt tggcctgcat

172861 gctgaaaacc tgagtacact cccggctgtg cgcctgaaaa atctcgcacg ttatgctggt

172921 atgacttcgg tgttcaatat tgccaggatg tcaccgcaga aaaggatggc ggttctggtt

172981 gcctttgtcc ttgcatggga aacgctggcg ctggatgatg cattggacgt tctggacgcc

173041 atgctggccg ttatcatccg tgacgccaga aagattgggc agaaaaaacg gctccgctcg

173101 ctgaaggatc tggataaatc tgcattggcg ctcgccagcg catgttcgta cctgctgaaa

173161 gaagaaacac cggacgaatc gattcgtgct gaggtgttca gctacatccc aaggcaaaag

173221 ctggctgaaa tcatcacgct tgtccgtgaa attgcccggc cctcagacga taattttcat

173281 gaagaaatgg tggagcagta cgggcgcgtt cgtcgtttcc tgccccatct gctgaatacc

173341 gttaaatttt catccgcacc tgccggggtt accactctga atgcctgtga ctacctcagc

173401 cgggagttca gctcacggcg gcagtttttt gacgacgcac caacggaaat tatcagtcgg

173461 tcatggaaac ggctggtgat taacaaggaa aaacatatca cccgcagggg atacacgctc

173521 tgctttctca gtaaactgca ggatagtctg aggcggaggg atgtctacgt taccggcagt

173581 aaccggtggg gagatcctcg tgcaagatta ctacagggtg ctgactggca ggcaaaccgg

173641 attaaggttt atcgttcttt ggggcacccg acagacccgc aggaagcaat aaaatctctg

173701 ggtcatcagc ttgatagtcg ttacagacag gttgctgcac gtctttgcga aaatgaggct

173761 gtcgaactcg atgtttctgg cccgaagccc cggttgacaa tttctcccct cgccagtctt

173821 gatgagccgg acagtctgaa acgactgagc aaaatgatca gtgatctact ccctccggtg

173881 gatttaacgg agttgctgct cgaaattaac gcccataccg gatttgctga tgagtttttc

173941 catgctagtg aagccagtgc cagagttgat gatctgcccg tcagcatcag cgccgtgctg

174001 atggctgaag cctgcaatat cggtctggaa ccactgatca gatcaaatgt tcctgcactg

174061 acccgacacc ggctgaactg gacaaaagcg aactatctgc gggctgaaac tatcaccagc

174121 gctaatgcca gactggttga ttttcaggca acgctgccac tggcacagat atggggtgga

174181 ggagaagtgg catctgcaga tggaatgcgc tttgttacgc cagtcagaac aatcaatgcc

174241 ggaccgaacc gcaaatactt tggtaataac agagggatca cctggtacaa ctttgtgtcc

174301 gatcagtatt ccggctttca tggcatcgtt ataccgggga cgctgaggga ctctatcttt

174361 gtgctggaag gtcttctgga acaggagacc gggctgaatc caaccgaaat tatgaccgat

174421 acagcaggtg ccagcgaact tgtctttggc cttttctggc tgctgggata ccagttttct

174481 ccacgcctgg ctgatgccgg tgcttcggtt ttctggcgaa tggaccatga tgccgactat

174541 ggcgtgctga atgatattgc cagagggcaa tcagatcccc gaaaaatagt ccttcagtgg

174601 gacgaaatga tccggaccgc tggctccctg aagctgggca aagtacaggt ttcagtgctg

174661 gtccgttcat tgctgaaaag tgaacgtcct tccggactga ctcaggcaat cattgaagtg

174721 gggcgcatca acaaaacgct gtatctgctt aattatattg atgatgaaga ttaccgccgg

174781 cgcattctga cccagcttaa tcggggagaa agtcgccatg ccgttgccag agccatctgt

174841 cacggtcaaa aaggtgagat aagaaaacga tataccgacg gtcaggaaga tcaactgggc

174901 acactggggc tggtcactaa cgccgtcgtg ttatggaaca ctatttatat gcaggcagcc

174961 ctggatcatc tccgggcgca gggtgaaaca ctgaatgatg aagatatcgc acgcctctcc

175021 ccgctttgcc acggacatat caatatgctc ggccattatt ccttcacgct ggcagaactg

175081 gtgaccaaag gacatctgag accattaaaa gaggcgtcag aggcagaaaa cgttgcttaa

175141 cgtgagtttt cgttccactg agcgtcagac ccctaaactt atcatcccct tttgctgatg

175201 gagctgtaca tgaacccatt caaaggccgg cattttcagc gtgacatcat tctgtgggcc

175261 gtacgctggt actgcaaata cggcatcagt taccgtgagc tgcaggagat gctggctgaa

175321 cgcggagtga atgtcgatca ctccacgatt taccgctggg ttcagcgtta tgcgcctgaa

175381 atggaaaaac ggctgcgctg gtactggcgt aacccttccg atctttgccc gtggcacatg

175441 gatgaaacct acgtgaaggt caatggccgc tgggcgtatc tgtaccgggc cgtcgacagc

175501 cggggccgca ctgtcgattt ttatctctcc tcccgtcgta acagcaaagc tgcataccgg

175561 tttctgggta aaatcctcaa caacgtgaag aagtggcaga tcccgcgatt catcaacacg

175621 gataaagcgc ccgcctatgg tcgcgcgctt gctctgctca aacgcgaagg ccggtgcccg

175681 tctgacgttg aacaccgaca gattaagtac cggaacaacg tgattgaatg cgatcatggc

175741 aaactgaaac ggataatcgg cgccacgctg ggatttaaat ccatgaagac ggcttacgcc

175801 accatcaaag gtattgaggt gatgcgtgca ctacgcaaag gccaggcctc agcattttat

175861 tatggtgatc ccctgggcga aatgcgcctg gtaagcagag tttttgaaat gtaaggcctt

175921 tgaataagac aaaaggctgc ctcatcgcta actttgcaac agtgcctctc agattcaggt

175981 tatgtcgctc aatgcgctga gtgtaacgct tgctgataac gtgcagcttt cccttcaggc

176041 ggggttcata cagcggccag ccatccgtca tccataccac gacctcaaag gccgacagca

176101 ggcccagaag acgctccagc gtggccaacg tgcgttacct aacaataaac ctgtttaaat

176161 atccagataa aaacattcaa tctgggtcaa atgagtgata cagtttcacc cataagaccc

176221 aatggaggca atatgtctga atttgaatta ctggcgcagg atctgcttga gaaagcagaa

176281 gcggaagaac aactgcgaca ggaaaatgat aaaaagctgc tcgggcaggt gctggaaatc

176341 tatgaccaga agtacgtggc tgaactgctt agaaaagttg gtaaaaatga gtggagtcgc

176401 gagactctta atcgctggat taatggtaag tgctcaccta agacgctgac gttagccgaa

176461 gaggaacttc tacgaaaaat gcttccggaa gcgcctgcac atcaccctga ctatgccttc

176521 cggtttattg acctgtttgc tgggattgga ggtatacgga agggcttcga aaccatcggt

176581 ggccagtgcg tttttaccag tgaatggaat aaagaggctg tgcgcacata taaagctaac

176641 tggtttaacg atgctcagga acacactttc aatctcgata ttcgtgaagt cacgctcagt

176701 gataaacctg aagtacctga aaacgatgcc tatgcttaca ttaatgagca tgtgccggat

176761 catgatgtac ttctagcagg tttcccctgt caaccgttta gccttgcggg cgtaagcaag

176821 aaaaactcgc tcgggcgcgc gcatggtttc gaatgtgagg ctcagggaac gcttttcttc

176881 gatgtggcgc gtattatccg cgcaaaaaaa cctgccatct ttgttcttga aaacgttaaa

176941 aacctgaaga gccatgacaa gggtaaaacc tttaaagtca tcatggatac cctcgacgaa

177001 ctgggctatg aagttgcgga tgcagctgag atgggcaaaa acgatcctaa agttatcgac

177061 ggaaagcact ttttacctca gcaccgagaa cgtatcgttt tggtcggttt ccgccgtgat

177121 ctgaacattc accagggctt taccctgcgc gatattagtc gtttttatcc ggaacagcgt

177181 ccgtcatttg gcgaactgct ggaacccgtg gttgacagca aatatatact gacgccgaaa

177241 ctctgggagt atctctataa ctacgccaaa aagcacgcag ctaagggtaa cggattcggt

177301 tttggcctcg ttaatcctga aaataaagaa agcattgccc gtacgctttc tgctcgctat

177361 cacaaagacg ggtctgaaat tctgatagac cgtggctggg atatggccac aggtgaaaca

177421 gacttcgcga acgaagaaaa tcaggcgcat cggccccgca ggctgactcc gcgagagtgc

177481 gcgcgcctta tgggttttga aaaagtagat ggcaggcctt ttcgcattcc tgtgtcagac

177541 actcagtcgt acaggcagtt cggtaactcc gtagtggtgc ccgtgtttga agccgtagcc

177601 aaactgcttg aaccttatat cctgaaagcg gttaatgccg attcgtgcaa ggttgaacga

177661 atctgatcgc tcctcccggt atttatgccg ggagataatc tatggaatat ctgcgtaaag

177721 ccctgtcagc tcagcaataa acgcacctag cgtcattagc tcagctctca ccgcctccgg

177781 gtattttttg tgcagagatg atggcacgac caatctgaca cccgactccc gcatctcccg

177841 atattgagcc agagaaactc cctcctggag tgtaaacaga tgcacctgat gaattttatc

177901 ggcctcattc agtatctgac gccagcgatc cttacaggta gtcttgactg ccagcatgcg

177961 cagattttct acgggaaact cagtatcgtg gtaagcccct gcggaaggga aaaggaaatc

178021 gggtttttta ttaccttctg tgatggcctg cgtcgcaaag tgtcgcaggc cgtgctcaat

178081 gaatagatgc tccaggtgca gttccagcga cttcccggct ctggatttac ggcgattgct

178141 gacagaattg gccagcgcaa taaattcatc cacagagcca aatcctttcc ggatgatatc

178201 cagaacatgc agttcctcaa ccaatagaaa tatgtcgtac tccacgcgcc ggcggtcaag

178261 aagttgctca tccggatcaa gggaattttt cacataatgg ctggctgcat actgaataat

178321 ttcacttccc gacggaaagc gcaggtgcca gtcttcaggt agaatatatt tatgatttac

178381 tggcgcttgc tgtagagata gtccgcctag aatctgtcct gcggggccgg atataagcgc

178441 tccgggtata acttcaccaa tagcggtctc aatgacgtcc tcttcatcag tgctggcgca

178501 tacccaaata tttacttcct tacagtcccc cccttgctca tcaagcttga aagccaggag

178561 cgtcagagcc cctgtatttt caggatcctg aagtgggctg cctctacccc agcgggtaat

178621 ccttttttca ttccgggttt taccaaaatg acggctgtta taataaattg ccctggcttc

178681 gctgtcaggg caatcatgcg atgacacatg tgcggtgaga aaaaccgaag ggttcagttc

178741 acgggtatgg ttgatagacg gaaagagttt ttcaacgata cctgaaggga tataaagccc

178801 tacctggtga ccacctgttg cgccggtatc gttggcggaa aggcgtttga tgtagacgaa

178861 gtaattctca catgcgatct caagtagcca gttgtggaaa accgacataa gcatcccctg

178921 ttaccctgaa actctactca ccattttttc atgattatat acaaacagtg tcattttcag

178981 aagacgactg caccagttga ttgggcgtaa tggctgttgt gcagccagct cctgacagtt

179041 caatatcaga agtgatctgc accaatctcg actatgctca atactcgtgt gcaccaaagc

179101 gaggtgagca tggcgacgga ggctctgttg caaagattgg cggcagtcag aggtaggctg

179161 tcgctctgcg ccgatcaggc ggctgctgcg aaatggtggt tgagcatgcc catggcctcc

179221 gtcagcgccg agggcccaat gccaaaagct ctctccacaa ggcgcacctc gcccctgatg

179281 ccgggctgca ggcaccaggg gcgagcctgt cctttgcgca gggctcgcat gacttcgaat

179341 cccttgatcg tggcataggc cgtggggatc gatttgaaac cgcgcaccgg cttgatcagt

179401 atcttgagct ttccgtgatc ggcctcgatc acgttattga gatacttcac ctgccggtgg

179461 gccgtctccc ggtccagctt tccttcgcgc ttcaattcgg tgatcgctgc accatagctc

179521 ggcgctttgt cggtattgag cgtggcaggc ttttcccagt gcttcaggcc tcgcagggcc

179581 ttgcccagga accgcttcgc tgccttggcg ctgcgggtcg gcgacaggta gaaatcgatc

179641 gtgtcgcccc gcttgtcgac tgcccggtac aggtaggtcc acttgccccg caccttgacg

179701 taggtttcat ccaggcgcca gctcggatca aagccacgcc gccagaacca gcgcagccgc

179761 ttctccatct ccggggcgta gcactggacc cagcgataga tcgtcgtatg gtcgaccgaa

179821 atgccgcgtt ccgccagcat ttcctcaagg tcgcgatagc tgatcggata gcgacaatac

179881 cagcgcaccg cccacaggat cacatcaccc tggaaatggc gccacttgaa atccgtcatc

179941 gttccgtccg tccaatctcc gccaagcatg ctcaagcttc acgatttttg caacagagcc

180001 cacacgagta ttgagcatag tcgagattgg tgcagatcac ttctgatatt gaactgtcag

180061 gagctggctg cacaacagcc attacgccca atcaactggt gcagtcgtct tctgaaaatg

180121 acaatcgtgc caccggctcc aggtcttatc gaacagctca gacacatggt ccaaatgctg

180181 gtcgatccgt tcggccagtt tttcgatggc ctcttgcttg agcttgtcac tttccctgaa

180241 ctcgatgttc aaggccctgg ccacctggtt gaggttccgg ccgatagctc caagctgccg

180301 gcacgattcc cgtacggtgt tcacttcttc atcagtgagg accggcatcc ggttcaacga

180361 ccgcagtcat ttctgtacct aaacagtcct gcgaatatgt gagttttgac ctaatgagcc

180421 acaaaaactc attttcgccc acgataattt ttctacttcc tttctttgga atgctcattt

180481 caatattcac ataacgtcgc tatcaatcgc ggaaaaggcg tagtgcgcga agcgaacgaa

180541 gctttttgcc gtcgattgca tagctttgtt aacccttttt ccaaatttga tagcaatagt

180601 taatgtttga agtaaagtgt tgctcaaaaa caacttcgaa ggtatttgga atactcggga

180661 agaaaacatc cccctctggc tcgatgtcga tcgtcgataa gtggagcgta gaggccatgg

180721 gtaatgtttc tcggtaaatt tctccgccac cagacactat aacgtgaccg gtgaattcag

180781 ctagcctgtc catggcctct tcgattgact gaaatacaac tacattgtca tcatttgatg

180841 tccaacctga gcgggtaacg accgcgtatt tcctattggg gagtgcgccc atagattcaa

180901 acgtcttgcg acccaccaga agccactgat tgtaggtcaa tgctttaaaa agtagctgct

180961 cccctttcgc ggaccagggt atgtctggac cgcaaccaat cacgccgttt ttcgctttcg

181021 cagccatcaa tgatactttc aaggttctca tcctgggtta actttgtttt agggcgactg

181081 ccctgctgcg taacatcgtt gctgctccat aacatcaaac atcgacccac ggcgtaacgc

181141 gcttgctgct tggatgcccg aggcatagac tgtacaaaaa aacagtcata acaagccatg

181201 aaaaccgcca ctgcgccgtt accaccgctg cgttcggtca aggttctgga ccagttgcgt

181261 gagcgcatac gctacttgca ttacagttta cgaaccgaac aggcttatgt ccactgggtt

181321 cgtgccttca tccgtttcca cggtgtgcgt cacccggcaa ccttgggcag cagcgaagtc

181381 gaggcatttc tgtcctggct ggcgaacgag cgcaaggttt cggtctccac gcatcgtcag

181441 gcattggcgg ccttgctgtt cttctacggc aaggtgctgt gcacggatct gccctggctt

181501 caggagatcg gaagacctcg gccgtcgcgg cgcttgccgg tggtgctgac cccggatgaa

181561 gtggttcgca tcctcggttt tctggaaggc gagcatcgtt tgttcgccca gcttctgtat

181621 ggaacgggca tgcggatcag tgagggtttg caactgcggg tcaaggatct ggatttcgat

181681 cacggcacga tcatcgtgcg ggagggcaag ggctccaagg atcgggcctt gatgttaccc

181741 gagagcttgg cacccagcct gcgcgagcag ctgtcgcgtg cacgggcatg gtggctgaag

181801 gaccaggccg agggccgcag cggcgttgcg cttcccgacg cccttgagcg gaagtatccg

181861 cgcgccgggc attcctggcc gtggttctgg gtttttgcgc agcacacgca ttcgaccgat

181921 ccacggagcg gtgtcgtgcg tcgccatcac atgtatgacc agacctttca gcgcgccttc

181981 aaacgtgccg tagaacaagc aggcatcacg aagcccgcca caccgcacac cctccgccac

182041 tcgttcgcga cggccttgct ccgcagcggt tacgacattc gaaccgtgca ggatctgctc

182101 ggccattccg acgtctctac gacgatgatt tacacgcatg tgctgaaagt tggcggtgcc

182161 ggagtgcgct caccgcttga tgcgctgccg cccctcacta gtgagaggta gggcagcgca

182221 agtcaatcct ggcggattca ctacccctgc gcgaaggcca tcggtgccgc atcgaacggc

182281 cggttgcgga aagtcctccc tgcgtccgct gatggccggc agcagcccgt cgttgcctga

182341 tggatccaac ccctccgctg ctatagtgca gtcggcttct gacgttcagt gcagccgtct

182401 tctgaaaacg acaaacagcc agaaaggctg ttacaggcga tttgatctgc aacctattgg

182461 ttaaattaat gtatcaaaaa cgatggtttt tgtgacagtc ttgaaaagtc ctgacttctc

182521 ccgaaaaatg actcccctca tgtaacaaaa ctcgttactg tatcaacata acaataaccc

182581 cataactaat tagcgagaaa agaatgaaaa tcggctatgc acgtaaatct acgcatcttc

182641 aggatgtggc gcaccaggtt gacgaactaa caaaagctgg atgtgagcaa atcggcactg

182701 ttgcaaatag tcggtggtga taaacttatc atcccctttt gctgatggag ctgcacatga

182761 acccattcaa aggccggcat tttcagcgtg acatcattct gtgggccgta cgctggtact

182821 gcaaatacgg catcagttac cgtgagctgc aggagatgct ggctgaacgc ggagtgaatg

182881 tcgatcactc cacgatttac cgctgggttc agcgttatgc gcctgaaatg gaaaaacggc

182941 tgcgctggta ctggcgtaac ccttccgatc tttgcccgtg gcacatggat gaaacctacg

183001 tgaaggtcaa tggccgctgg gcgtatctgt accgggccgt cgacagccgg ggccgcactg

183061 tcgattttta tctctcctcc cgtcgtaaca gcaaagctgc ataccggttt ctgggtaaaa

183121 tcctcaacaa cgtgaagaag tggcagatcc cgcgattcat caacacggat aaagcgcccg

183181 cctatggtcg cgcgcttgct ctgctcaaac gcgaaggccg gtgcccgtct gacgttgaac

183241 accgacagat taagtaccgg aacaacgtga ttgaatgcga tcatggcaaa ctgaaacgga

183301 taatcggcgc cacgctggga tttaaatcca tgaagacggc ttacgccacc atcaaaggta

183361 ttgaggtgat gcgtgcacta cgcaaaggcc aggcctcagc attttattat ggtgatcccc

183421 tgggcgaaat gcgcctggta agcagagttt ttgaaatgta aggcctttga ataagacaaa

183481 aggctgcctc atcgctaact ttgcaacagt gccagaagaa gacgatgcgc gtgaagtggt

183541 cgatctgata aaaaaagcag gcagaaatgt tttggccatc cccggagata tccgtgatga

183601 ggctttttgc ggtcatctgg taacacaggc ggtaaaagga ctgggagggt tggatatcct

183661 cgtcaataat gcgggtcgtc agcaattttg tgagtcaatt gaggaactca ccacagaaga

183721 cttcgacgca acattcaaaa ccaatgttta cgctatgttt tggatcacca aagcggccat

183781 accccatctt tcaccagaca gcgtgataat taatacctcc tccgtacagg cttatgagcc

183841 aagtgaaatc ttgcttgatt atgctcagac taaagcagct atcgtggcat ttactaaatc

183901 gctggcgaaa cagctggccc cgaaagggat ccgtgtcaat gccgtcgcgc cgggtccata

183961 ctggactgta ctgcagtgct gtggtggtca accgcaggaa aaggtggaga aatttggggc

184021 aaatgcgccg ctgggacgcc ctggtcaacc ggtggaaatc gcgccgcttt atgtcaccct

184081 ggccgctcgg gagaacagct atacgtctgg tcaggtctgg tgttctgatg gggggaccgg

184141 aaccctctaa cgtttacgat ctgtgcgtcg aaagtgactt ttaaggcaag ggcggacact

184201 attgtataag ggcgggagcg gtacttaccg ctccgtgaat gctaaccaat caccgcgatg

184261 ccaagtcgct ccatgagcaa cgatgcctgg tagttgtcca acttaacgcc ttgtaaatca

184321 acgccccgaa tatctaagtc acccaactcc gaattggtca gatcgcaatg tgtgaagttt

184381 gctgctcgcc agtcgaaagt cgaaaactcg ccgccggaga gatctgaacc actgaacgtc

184441 gcgcccagta cctgggcacc tatccaacgg ttttcccaca gctcacactt ttccaacacg

184501 actttcgaaa aattggcgta gcttagattg gtattcgtga tatatgcgct acaaaaccag

184561 gtgcgcgtgg tgatcatatt cataaagctt gcgccgcgga aatctgcgcc ttgtgcgcgg

184621 cagtggcgaa tttcaatgcc cagcgcactg gaattgcgaa aatccgccat ggataaatca

184681 cagcttttaa aaatggcatc tttcagcatc gcacgactaa aattgcaccc tttctggctt

184741 tcacgatcat agaactgaca gccgataaat tcagtgccgc tcaggtcggc acctgaaaaa

184801 tcacagttaa aaaatgtact attttcaatt ttctcaccgg tgaaacggtt tctgtcaatt

184861 ttttcgccaa cgagtgccag agccatattt gtacctgttt tttatacagt aatggcgtca

184921 tggtaaaccc tgatgaggtt atgcgtcaaa tccgccaata tgacatctgc aaatgtgcgt

184981 taaatctggt gttttttcag caaagcgcga agctgatggt aagtcagacc cagtaattca

185041 gcggcttttt tctggttaaa ttttgcctgc tgtaagctgg tttgtagaaa gtctttctct

185101 tgctgctgct ggaattcacg cagatccagc ggtaacccta cagacatcgg tttagtttcc

185161 ggcgcctgcg gctgagtagt gtggcggcgg aaggggtcaa tgattatctc atccagcggg

185221 taatcactgg ttccatgccg gtaaacggat cgctccacga cgtttttcag ttcacgaata

185281 ttgcccggcc aacggtagtg gaggagagtt tctctggcct cggcactaaa acccggaaac

185341 agaggtaatc cgatctcgcg gcacatctga atggcaaaat ggggttctag ggattttccg

185401 tccaaaaacg acaaagtgct ctggaggccg cgccgtccgt ggcctccaga ggggtattac

185461 ttttcgctga cgggtaaata tccctcgatt gagcgcataa gctcctccag atctggcaat

185521 ttttcgctca attcgaagtg gtaagtcccc ttgaggttaa tgttgtgcca ggccacaggg

185581 gatgcctgtt tgacgatatc tattctcttg gtatctcctt ggtattcgaa gctggtcaac

185641 agctggctga gtatcctgga gttgaagtaa acgatggcat tggtgaccag gcgagcgcac

185701 tcattccata gctggatttc ttcgtctgaa ctgccccgga actgatcccc attgacgctg

185761 ctcacggccc gacgcagttg gtgataggcc tctccccggt tcagcgcgcg ctgaacatag

185821 tttcttaaac tggcatcatc gatgtagcac agtagataat tcgctttcac caggcgattg

185881 tattccgtca gggcttccag cagcgggtga ttgcgcttgt actccgagag ctttctcacc

185941 aaggtggctt gcgttgtttt ccgctgctta agtgatactg caatccgttg gatggtatcc

186001 cagtgctgcg caatacgatg ggtattgatt ggctttttta agcacagctg aattcggtgt

186061 tctttgtctt ccttgacatc aaacatgtca ttgatcactt tgccaacctg ggcatagcgt

186121 ggggcaaact ggtatccgaa cagatccagt aacgcgaagt tcacatggtt caccccatgg

186181 gtatcggttg agagcacatc cggaatgatg tctgacgtat tgctcatcaa caaatcaaag

186241 atgtagtgcg attcatgttc gttggcgccg atcactctgg cgttgatcgc agcgtgattg

186301 gcgatcaagg tcatggcaga aacacctttt tgagtgccaa agtacttcga cgaataacgg

186361 gttttgaagg tctcgcgccg ggcttcgaac ttttgaccat cggcactggc gtggatcaca

186421 tcttcctgga tgttgtagta ccggaagatg ggtagcttgg ctgtcgcgtt attgatgttg

186481 tcgttagcag cattcaatgt ttccaggcga agatagttcg cctggatagt gctgagctga

186541 tcataggtac gatcagagat ctgtgccatg ccgtaaatgc cttgattggt tgcattgccg

186601 accagaattg ccaacaggtc atattcatgg gaacggcttc tggattggga acccagcaca

186661 tgagcgaagc agtcaatgaa accggtgtca cgatcaacca tgcgcagtac atccgcaatc

186721 cccgttgtgg gaatttgctg gaagaaggga ttgttgacca gatgatgttt gctggccgaa

186781 ggcaggcgcc agaagcgttt accctgcgga ttacgcaaga tgatattccg gttgtcttcc

186841 tgttcaagat attcgccgac ttcgtacaaa cgggtatcca attccatggc catctgtttg

186901 atcagctttt caggctcctc cgctaatttt gtaaaatggc tctgttgaag cagcgtatat

186961 ttgttttttc gccaatgttc cccgtcgatc aggtcggcgt cgagtgcccg gtatttagtg

187021 atatcaggca gcgtcagctg gccattcagg cgatcaggaa tctgttgata gaggaaccac

187081 tcataacgat cgatcaggat attcccttca ccatccagca ggaattcacg tgattttttg

187141 gaaaggagtc tggtgtcggc agtttgcaac tgagcgtcct ggccgttgag ttcgttttgt

187201 gttttcgcca aggcggccgc taagtgctgg gtgccgtcgc agccttcgaa gcgcagacat

187261 ggggtcggtt ccggctgagg gcgaaatgac accctaagct ttcggttcct tgggccaaag

187321 atattcgcca gtcagtagaa tgtgcgccca gcccaatggg gatatgtggg gaagaaattc

187381 agggggaaca tccaaccctt cgttccgccg ctccgtgacg gcatgaccaa gatggacggt

187441 attccagtaa atgatcaccg cagtcaataa attgagccca gcgattcggt agtgctgccc

187501 ctctgtcgtg cgatcgcgaa tttccccctg cctcccgata cggagcgcat ttttgagcgc

187561 atggtgggcc tctcccttgt taagaccgat ctgagcacgc cgctgcatgt ccgtatccag

187621 gatccactca ataatgaaaa gggtccgttc aatacgacca acttcacgaa gcgcaactgc

187681 aaggttgttt tgtcgtgggt aagaagcgag cttgcgcagg agttggctgg gcctgatttt

187741 gccagcggtc atcgtcgcgg cacaacggaa aatatcaggc cagttcgcaa cgataagatc

187801 ctcccgggct tttccaccta ccaacttgcg taactccctg ggggtcgtat cgggattaaa

187861 tacgtacaac cgcttcgatg gcagatccct gattcgcaga acgagattgt agccgagcag

187921 gctactggct ccgaacaaat ggtcggtgaa tcctgctgta tcggcatact gttcgcgaac

187981 atggcgaccg acctcgttca tcagtagtcc atcgagaata tacggtgcct cgctcacggt

188041 cgccgggatc gactgacaag cgaatggcgc gaactggtcg cttacgtgag tatacgcttt

188101 gaggccggga acagaaccat atttggcatt gaccatgttc atggcttcgc catgccgcgc

188161 tgtcgggaaa aactgaccat cgctcgatgc tgacgtgccc atcccccaga cgcgtgacat

188221 cggcagttta ccctgcgcgg ccaccacaat tgccaatgcc tggttcatgg cttcgctttc

188281 aacatgccag cgggcaaggc gtgagagctg ccagtaatca tgcgtgtttg tagcttccgc

188341 catcttacgc aggcccagat tgagcccttc agcgagcagg acgttgagca gaccgatccg

188401 gtcgcgacat ggagccccgg ttctcagatg ggtaaacgca tctgtgaaac caagggctgc

188461 atcaacttca agcagcatgt cggtaatccg aacggacggc attcggcgat acagatccag

188521 tatgagtgcc tcggcaccat ccggcacgtc tgctgtcaac ctgtcgatcc gcaacgttcc

188581 atcttctatg ctaccgtgcg gaatagtgcc gttacgggca gcccgggcca gccgcttaag

188641 agcgatcgtg agtcgcgcct ttctgtctgc cagccaatcc tgtgggttgg aaggcacggc

188701 cagttttgca ttttcctgcg ccgcgatcat cggcaccagt acctgcttga ggtcaccata

188761 gcggcgcgaa tgagcgagcc agacatctcc ggaacgaaaa gcatcccgga ggtgaaagag

188821 taccgccact tcccaaagac gggtatctcc tttttcctga gctcgtaaat gacggttcca

188881 tttggagctg ggccgcagga aacgcctttc tggcgatgca acacctttca tctctccgat

188941 cgacaaagct gctgctacca atggtccggc gaccggcgcg gcttcgagct tcagacagcg

189001 caacatgcgg ggcgcataac gacgaaagcg atggtatccc tgcccgacat atgcaagagg

189061 ctcatcggct agcgtgttgc tgagttgagt ccctgtcgct accagttgag cgagccggtc

189121 ccatgcaacc gaactggcga cagccatctc cagcggggtt ccgtcactgc gggcctcaag

189181 caacgaagct cccagcgcgg tgaaggtacg gatcgtatcc gtgagtgtgg ctttagagcc

189241 ggaaattgtt tcgtcatgct ggcgcttcgc ttcccgccag gtttttccta cgatcctgtc

189301 atgggtttcg actatggcat cagcaatcgc cgcttcccac tccacaacac agacggcaag

189361 gatcgcccag cggcggtccg aagtgatgtc acgcaaaccg tcggtgaagt agcgttcacc

189421 ctgccgacgc agccgggcaa tgcgatgggc aggtatgctg gccaaagcac tatgattgat

189481 attcagggta cgcagaaatt cgagcctgtc gagcaaacgg ttagcagcag ccgagttgtt

189541 accaacctcg aagttgcgaa gccagatgaa acgactgata ttgccggcga gcatttcact

189601 cagaagtttg tccaggtgat cgcgaacatc cgctgttaaa ttttccgcaa tccgcgtttc

189661 aatccgccgc tcagcggcga ccagagcatc cgcgcacaag cgctcgattg tcgatactgc

189721 gggcagaatg gtggaagttt cccgacaccg cacaataaaa cgatgagcaa gatcctcgtt

189781 tgatctggca tcttcggcct ggccgaaagt ccactcccgc agatcacggg caccacggcc

189841 cgtgaaggtc ttgtagccgt aaatttcgcg cagcgtgtcc atgtgctgct gacgggtttg

189901 gcgccgtgtg gcataagtga gaagcgcatc agccggaact ccaagctgag caccgacgaa

189961 ggaaaggact tcacgcggga tcatctcacc aggagccagt gcacggcccg gatatcgtaa

190021 ggcacaaagt tgcagggcaa agccaatcct gttttccggt ctgcggcgct gcctaatgtt

190081 ttccaggtca tcatcgccca gcgtgtagaa cttcagtagc gacagttcgt ccgtgggcag

190141 atcgaacagc gctgctcgct gccgttcggt gaaaatatgg cgtcgtgaca tacaaattcg

190201 tcccttttga agtatagtct gttttggaca acagccagcc catataaatc agggcgttcc

190261 gatacaaaaa tccaggaggg ttcaattggg acatcgtgcc gccatttact gccgggtttc

190321 aacagcggat cagtcttgtg aacgccagga atttgatctg cgagccttcg ccggctgtgc

190381 cggctacgac gtggtgggaa tatttaagga aacaggttca ggaactaaac tcgaccgggc

190441 cgagcgaaag aaagtcctgg cgcttgccca gtccagacaa attgatgcaa tcctggtcac

190501 tgagctttcc cggtgggggc gctcgacgct cgatctgctc aatacgctac gtgaactgga

190561 gaactggaag gtttccgtga tagccatgaa tggaatggcg ttcgatcttt cgtcgccgta

190621 tggacgaatg ctggcgacgt ttctttccgg cattgcggag tttgagcggg atctcatcag

190681 cgagcgggtc aagtcaggcc ttgctgttgc gaaggcacgt ggtaagaggc ttggtcgtca

190741 ggccggagtg cgaccaaaat cagaccgact tttgcctaag gtggttgcga tgagggccga

190801 gggacgcagc tatcgctgga tcgcacgcga gctcggtatc agcaagaata ccgtcgctga

190861 catcgtgcaa cgacacagag ctaacgctta gggtgtcatt tcgccctcag ccggaaccga

190921 ccccgaatag gtttcgtcgc ggatgtgcca ggcttgcagc caggacagct tggcgtaggt

190981 caggccgggg ctcgactcgg ccatcttggt cagcccgagg ttgatcgcat caccgaggat

191041 tgcggacagc agcaacgtcc tgtctttggc ctcggccccg tccttcaagt gggtgaagtg

191101 gcggctgaag cccgtccagt cgtccacgtc catcagcagt tcggtgatct tgatgcgcgg

191161 cagtaactgg ctggtttggt cgatcagcgc ctgcgcccga tccggcaccg ccgcatccag

191221 cggggtgatt ttcagccctg actcggtgag gatggcatcg ggcagctcgt tgtccttggc

191281 caggcgggtg acggtggcca actgctcgtc cagcagctgc aaacgctctt ccaggtactg

191341 gtcgctgttc gggttgatcg ccaggggcag ggcctgctcg cgcttgagtg cggcgaactt

191401 ctcggccggc agcaggtagt cgtcgaagtc gcggaactgc cgcgagccct tgacccagat

191461 gtcgccggag cgcagggcgt tcttcagctc ggacagggcg cagatttcgt agaatttccg

191521 gtcgaggcct tccggggtga tcaccagcgg cttccagcgc ggcttgatga aggccgtggg

191581 tgcatcggcc ggcaccttgc gcaggttgtc ggcgttcatc tcacgcaggg tctgcacggc

191641 tgccagcacg ccttgcgcgg ccggcccgtg ctgttcccca acttttcgct cggcgaagag

191701 tatgaacatg cgccgccggc gacgaatcgc caaatctcac cgtatctccc gagcggacga

191761 tttcgcaccg gtctgcccgt cgaagggctt gcgatcgaac ggggcgacct tttctatgca

191821 tgtccgcgag ccagcgtctt ttatggcacg gcgctcgacg ccgaccttcg gacgcgcggc

191881 gtaagcacgc ttgtcatggc cgggataagc accaccggcg ttgttctttc aagcgtcgcc

191941 tgggctagtg atgcggacta cgacgtgcgt ttggtccagg actgctgcta cgacccggat

192001 cgggatgccc acgaagctct gttgcgttcc gggttcggcg gacgtgtaca ggtcgtgtaa

192061 ttttcgggcc tcgcatgatc gcggccatag ccgcaggtgg attggtgcga ggcagggcca

192121 gtccaagtca gggtgcggtc atcgcggcgc ctgcaactct caaaaaccca tcgccgtagc

192181 ggaggtcttg taatcgcatg tcattacgca cgcccgattt attgttcaca gcgatagcac

192241 ctgccatttg gggcagcacc tacattgtca ccacccaata cctgccgaac ttctcaccga

192301 tgacggtcgc gatgctgcgg gcgttgccgg cgggtttatt gctcgtgatg atcgtccgac

192361 agattccaac gggaatctgg tggatgcgca tcttcatcct cggcgcactt aatatttcgc

192421 tattctggag cttgttgttt atttcggtct accgcctgcc gggcggggtc gcggcgacgg

192481 taggcgctgt gcagccgctg atggtcgtgt tcatctctgc cgctctgcta ggtagcccga

192541 tacgattgat ggcggtcctg ggggctattt gcggaactgc gggcgtggcg ctgttggtgt

192601 tgacaccaaa cgcagcgcta gatcctgtcg gcgtcgcagc gggcctggcg ggggcggttt

192661 ccatggcgtt cggaaccgtg ctgacccgca agtggcaacc tcccgtgcct ctgctcacct

192721 ttaccgcctg gcaactggcg gccggaggac ttctgctcgt tccagtagct ttagtgtttg

192781 atccgccaat cccgatgcct acaggaacca atgttctcgg cctggcgtgg ctcggcctga

192841 tcggagcggg tttaacctac ttcctttggt tccgggggat ctcgcgactc gaacctacag

192901 ttgtttcctt actgggcttt ctcagcccgg ggaccgccgt gttgctagga tggttgttct

192961 tggatcagac gctgagtgcg cttcaaatca tcggcgtcct gctcgtgatc gggagtatct

193021 ggctgggcca acgttccaac cgcactccta gggcgcgtat agcttgccgg aagtcgcctt

193081 gacccgcatg gcataggcct atcgtttcca cgatcagcga tcggctcgtt gccctgcgcc

193141 gctccaaagc ccgcgacgca gcgccggcag gcagagcaag tagagggcag cgcctgcaat

193201 ccatgcccac ccgttccacg ttgttataga agccgcatag atcgccgtga agaggagggg

193261 tccgacgatc gaggtcaggc tggtgagcgc cgccagtgag ccttgcagct gcccctgacg

193321 ttcctcatcc acctgcctgg acaacattgc ttgcagcgcc ggcattccga tgccacccga

193381 agcaagcagg accatgatcg ggaacgccat ccatccccgt gtcgcgaagg caagcaggat

193441 gtagcctgtg ccgtcggcaa tcattccgag catgagtgcc cgcctttcgc cgagccgggc

193501 ggctacaggg ccggtgatca ttgcctgggc gagtgaatgc agaatgccaa atgcggcaag

193561 cgaaatgccg atcgtggtcg cgtcccagtg aaagcgatcc tcgccgaaaa tgacccaaag

193621 cgcggccggc acctgtccga caagttgcat gatgaagaag accgccatca gggcggcgac

193681 gacggtcatg ccccgggccc accggaacga agcgagcggg ttgagagcct cccggcgtaa

193741 cggccggcgt tcgcctttgt gcgactccgg caaaaggaaa cagcccgtca ggaaattgag

193801 gccgttcaag gctgccgcgg cgaagaacgg agcgtggggg gagaaaccgc ccatcagccc

193861 accgagcaca ggtcccgcga ccatcccgaa cccgaaacag gcgctcatga agccgaagtg

193921 ccgcgcgcgc tcatcgccat cagtgatatc ggcaatataa gcgccggcta ccgccccagt

193981 cgccccggtg atgccggcca cgatccgccc gatatagaga acccaaagga aaggcgccgt

194041 cgccatgatg gcgtagtcga cagcagcgcc ggccagcgag acgagcaaga ccggccgccg

194101 cccgaaacga tccgacagcg cgcccagcac aggtgcgcag gcaaattgca tcaacgcata

194161 cagcgccagc agaatgccat agtgggcggt gacgtcgttc gagtgaacca gatcgcgcag

194221 gaggcccggc agcaccggca taatcaggcc gatgccgaca gcgtcgagcg cgacagtgct

194281 cagaattacg atcaggggtc tgttgggttt cacgtctggc ctccggacca gcctccgctg

194341 gtccgattga acgcgcggat tctttatcac tgataagttg gtggacatat tatgtttatc

194401 agtgataaag tgtcaagcat gacaaagttg cagccgaata cagtgatccg tgccgccctg

194461 gacctgttga acgaggtcgg cgtagacggt ctgacgacac gcaaactggc ggaacggttg

194521 ggggttcagc agccggcgct ttactggcac ttcaggaaca agcgggcgct gctcgacgca

194581 ctggccgaag ccatgctggc ggagaatcat acgcattcgg tgccgagagc cgacgacgac

194641 tggcgctcat ttctgatcgg gaatgcccgc agcttcaggc aggcgctgct cgcctaccgc

194701 gatggcgcgc gcatccatgc cggcacgcga ccgggcgcac cgcagatgga aacggccgac

194761 gcgcagcttc gcttcctctg cgaggcgggt ttttcggccg gggacgccgt caatgcgctg

194821 atgacaatca gctacttcac tgttggggcc gtgcttgagg agcaggccgg cgacagcgat

194881 gccggcgagc gcggcggcac cgttgaacag gctccgctct cgccgctgtt gcgggccgcg

194941 atagacgcct tcgacgaagc cggtccggac gcagcgttcg agcagggact cgcggtgatt

195001 gtcgatggat tggcgaaaag gaggctcgtt gtcaggaacg ttgaaggacc gagaaagggt

195061 gacgattgat caggaccgct gccggagcgc aacccactca ctacagcaga gccatgtaga

195121 caacatcccc tccccctttc caccgcgtca gacgcccgta gcagcccgct acgggctttt

195181 tcatgccctg ccctagcgtc caagcctcac ggccgcgctc ggcctctctg gcggccttct

195241 ggcgctcctg ctgcggcgtc cgctcgtggg ccgtggcgcg ggtccgcgcg ccggcctcgt

195301 gcgcctggcg ctcgcggccg tcttcacgtt ctgccttgcg cagatgagat aggggagccc

195361 gcagaattcg gaaaaaatcg tacgctaagg ttttccgggc atccgtaagg gccgaaactt

195421 cccgtcttcc agtctgcggc tctgccgcca gacgtaatcg ccggttaggt tgatgtgctc

195481 ccagcccagc ggcgacagga attgcagcag ctcgccgtcc accggcttgc cggcctcgac

195541 caacccctgg gtggcgcgtt ccaggtacac cgtgttccac agcacgatag ccgccgtcac

195601 caggttgagg ccgctggccc ggtagcgctg ctgctcgaag ctccgatccc tgatttcccc

195661 aaggcggttg aagaacaccg ccctggccag cgagttgcgc gcctcacctt tgttcaggcc

195721 ggcatgcacg cggcggcgca gttcaacact ttgcagccag tccaggatga acagcgtgcg

195781 ctcgatccgg cccagctcgc gcagggccac ggccagtccg ttctggcgcg ggtagctgcc

195841 gagcttgcgc agcatcagcg aggcggtgac ggtgccctgc ttgatcgagc tggccaggcg

195901 caggatgtcg tcccagtggg cacgcacgtg cttgatgttc agggtgccgc cgatcagcgg

195961 gcgcaacgtc gggtaggctt gcacgccctg cggcacgtac agcttggttt cgccgaggtc

196021 gcggatgcgc ggcgcgaagc ggaagcctag caggtgcatc agggcaaaga cgtgatcggt

196081 gaagccggcc gtgtcggtgt agtgctcctc gatccgcagg tcggactcgt ggtacagcag

196141 gccgtcgagc acataggtgg aatcgcggac gccgacattc accacgcggg tgctgaacgg

196201 cgcgtactgg tcggagatat gggtatagaa cagccgtccc ggctcgctac cgtacttcgg

196261 gttgacgtgc ccggtgctct cgccccggcc acccgcgcgg aagcgctggc catcggagga

196321 tgaggtcgtg ccgtcgcccc agtgggcggc aaaggcgtgg cgatactggt ggttgaccag

196381 ctcggccaag gccgccgaat aggtttcgtc gcggatgtgc caggcttgca gccaggacag

196441 cttggcgtag gtcaggccgg ggctcgactc ggccatcttg gtcagcccga ggttgatcgc

196501 atcaccgagg attgcggaca gcagcaacgt cctgtctttg gcctcggccc cgtccttcaa

196561 gtgggtgaag tggcggctga agcccgtcca gtcgtccacg tccatcagca gttcggtgat

196621 cttgatgcgc ggcagtaact ggctggtttg gtcgatcagc gcctgcgccc gatccggcac

196681 cgccgcatcc agcggggtga ttttcagccc tgactcggtg aggatggcat cgggcagctc

196741 gttgtccttg gccaggcggg tgacggtggc caactgctcg tccagcagct gcaaacgctc

196801 ttccaggtac tggtcgctgt tcgggttgat cgccaggggc agggcctgct cgcgcttgag

196861 tgcggcgaac ttctcggccg gcagcaggta gtcgtcgaag tggcactgtt gcaaatagtc

196921 ggtggtgata aacttatcat ccccttttgc tgatggagct gcacatgaac ccattcaaag

196981 gccggcattt tcagcgtgac atcattctgt gggccgtacg ctggtactgc aaatacggca

197041 tcagttaccg tgagctgcag gagatgctgg ctgaacgcgg agtgaatgtc gatcactcca

197101 cgatttaccg ctgggttcag cgttatgcgc ctgaaatgga aaaacggctg cgctggtact

197161 ggcgtaaccc ttccgatctt tgcccgtggc acatggatga aacctacgtg aaggtcaatg

197221 gccgctgggc gtatctgtac cgggccgtcg acagccgggg ccgcactgtc gatttttatc

197281 tctcctcccg tcgtaacagc aaagctgcat accggtttct gggtaaaatc ctcaacaacg

197341 tgaagaagtg gcagatcccg cgattcatca acacggataa agcgcccgcc tatggtcgcg

197401 cgcttgctct gctcaaacgc gaaggccggt gcccgtctga cgttgaacac cgacagatta

197461 agtaccggaa caacgtgatt gaatgcgatc atggcaaact gaaacggata atcggcgcca

197521 cgctgggatt taaatccatg aagacggctt acgccaccat caaaggtatt gaggtgatgc

197581 gtgcactacg caaaggccag gcctcagcat tttattatgg tgatcccctg ggcgaaatgc

197641 gcctggtaag cagagttttt gaaatgtaag gcctttgaat aagacaaaag gctgcctcat

197701 cgctaacttt gcaacagtgc ctttaagcgt gcataataag ccctacacaa attgggagtt

197761 agacatcatg agcaacgcaa aaacaaagtt aggcatcaca aagtacagca tcgtgaccaa

197821 cagcaacgat tccgtcacac tgcgcctcat gactgagcat gaccttgcga tgctctatga

197881 gtggctaaat cgatctcata tcgtcgagtg gtggggcgga gaagaagcac gcccgacact

197941 tgctgacgta caggaacagt acttgccaag cgttttagcg caagagtccg tcactccata

198001 cattgcaatg ctgaatggag agccgattgg gtatgcccag tcgtacgttg ctcttggaag

198061 cggggacgga cggtgggaag aagaaaccga tccaggagta cgcggaatag accagttact

198121 ggcgaatgca tcacaactgg gcaaaggctt gggaaccaag ctggttcgag ctctggttga

198181 gttgctgttc aatgatcccg aggtcaccaa gatccaaacg gacccgtcgc cgagcaactt

198241 gcgagcgatc cgatgctacg agaaagcggg gtttgagagg caaggtaccg taaccacccc

198301 atatggtcca gccgtgtaca tggttcaaac acgccaggca ttcgagcgaa cacgcagtga

198361 tgcctaaccc ttccatcgag ggggacgtcc aagggctggc gcccttggcc gcccctcatg

198421 tcaaacgttg ggcgaacccg gagcctcatt aattgttagc cgttaaaatt aagcccttta

198481 ccaaaccaat acttattatg aaaaacacaa tacatatcaa cttcgctatt tttttaataa

198541 ttgcaaatat tatctacagc agcgccagtg catcaacaga tatctctact gttgcatctc

198601 cattatttga aggaactgaa ggttgttttt tactttacga tgcatccaca aacgctgaaa

198661 ttgctcaatt caataaagca aagtgtgcaa cgcaaatggc accagattca actttcaaga

198721 tcgcattatc acttatggca tttgatgcgg aaataataga tcagaaaacc atattcaaat

198781 gggataaaac ccccaaagga atggagatct ggaacagcaa tcatacacca aagacgtgga

198841 tgcaattttc tgttgtttgg gtttcgcaag aaataaccca aaaaattgga ttaaataaaa

198901 tcaagaatta tctcaaagat tttgattatg gaaatcaaga cttctctgga gataaagaaa

198961 gaaacaacgg attaacagaa gcatggctcg aaagtagctt aaaaatttca ccagaagaac

199021 aaattcaatt cctgcgtaaa attattaatc acaatctccc agttaaaaac tcagccatag

199081 aaaacaccat agagaacatg tatctacaag atctggataa tagtacaaaa ctgtatggga

199141 aaactggtgc aggattcaca gcaaatagaa ccttacaaaa cggatggttt gaagggttta

199201 ttataagcaa atcaggacat aaatatgttt ttgtgtccgc acttacagga aacttggggt

199261 cgaatttaac atcaagcata aaagccaaga aaaatgcgat caccattcta aacacactaa

199321 atttataaaa aatctaatgg caaaatcgcc caacccttca atcaagtcgg gacggccaaa

199381 agcaagcttt tggctcccct cgctggcgct cggcgcccct tatttcaaac gttagacggc

199441 aaagtcacag accgcgggat ctcttatgac caactacttt gatagcccct tcaaaggcaa

199501 gctgctttct gagcaagtga agaaccccaa tatcaaagtt gggcggtaca gctattactc

199561 tggctactat catgggcact cattcgatga ctgcgcacgg tatctgtttc cggaccgtga

199621 tgacgttgat aagttgatca tcggtagttt ctgctctatc gggagtgggg cttcctttat

199681 catggctggc aatcaggggc atcggtacga ctgggcatca tctttcccgt tcttttatat

199741 gcaggaagaa cctgcattct caagcgcact cgatgccttc caaaaagcag gtaatactgt

199801 cattggcaat gacgtttgga tcggctctga ggcaatggtc atgcccggaa tcaagatcgg

199861 gcacggtgcg gtgataggca gccgctcgtt ggtgacaaaa gatgtggggc actgttgcaa

199921 agttagcgat gaggcagcct tttgtcttat tcaaaggcct tacatttcaa aaactctgct

199981 taccaggcgc atttcgccca ggggatcacc ataataaaat gctgaggcct ggcctttgcg

200041 tagtgcacgc atcacctcaa tacctttgat ggtggcgtaa gccgtcttca tggatttaaa

200101 tcccagcgtg gcgccgatta tccgtttcag tttgccatga tcgcattcaa tcacgttgtt

200161 ccggtactta atctgtcggt gttcaacgtc agacgggcac cggccttcgc gtttgagcag

200221 agcaagcgcg cgaccatagg cgggcgcttt atccgtgttg atgaatcgcg ggatctgcca

200281 cttcttcacg ttgttgagga ttttacccag aaaccggtat gcagctttgc tgttacgacg

200341 ggaggagaga taaaaatcga cagtgcggcc ccggctgtcg acggcccggt acagatacgc

200401 ccagcggcca ttgaccttca cgtaggtttc atccatgtgc cacgggcaaa gatcggaagg

200461 gttacgccag taccagcgca gccgtttttc catttcaggc gcataacgct gaacccagcg

200521 gtaaatcgtg gagtgatcga cattcactcc gcgttcagcc agcatctcct gcagctcacg

200581 gtaactgatg ccgtatttgc agtaccagcg tacggcccac agaatgatgt cacgctgaaa

200641 atgccggcct ttgaatgggt tcatgtgcag ctccatcagc aaaaggggat gataagttta

200701 tcaccaccga ctatttgcaa cagtgcctct cgataggtat agtgttttgc agtttagagg

200761 agatatcgcg atgcatacgc ggaaggcaat aacggaggca attcgaaaat taggagtcca

200821 aaccggtgac ctgttgatgg tgcatgcctc acttaaagcg attggtccgg tcgaaggagg

200881 agcggagacg gtcgttgccg cgttacgctc cgcggttggg ccgactggca ctgtgatggg

200941 atacgcgtcg tgggaccgat caccctacga ggagactctg aatggcgctc ggttggatga

201001 caaagcccgc cgtacctggc cgccgttcga tcccgcaacg gccgggactt accgtgggtt

201061 cggcctgctg aatcaatttc tggttcaagc ccccggcgcg cggcgcagcg cgcaccccga

201121 tgcatcgatg gtcgcggttg gtccgctagc tgaaacgctg acggagcctc acgaactcgg

201181 tcacgccttg ggggaagggt cgcccgtcga gcggttcgtc cgccttggcg ggaaggccct

201241 gctgttgggt gcgccgctaa actccgttac cgcattgcac tacgccgagg cggttgcgga

201301 tatccccaac aaacgatggg tgacgtatga gatgccgatg cttggaagaa acggtgaagt

201361 cgcctggaaa acggcatcag aatacgattc aaacggcatt ctcgattgct ttgctatcga

201421 aggaaagccg gatgcggtcg aaactatagc aaatgcttac gtgaagctcg gtcgccatcg

201481 agaaggtgtc gtgggctttg ctcagtgcta cctgttcgac gcgcaggaca tcgtgacgtt

201541 cggcgtcacc tatcttgaga agcacttcgg agccactccg atcgtgccag cacacgaagc

201601 cgcccagcgc tcttgcgagc cttccggtta gaggccgtcg acaatgataa tctggatcaa

201661 cggacctttc ggcgccggaa agacgactct cgctgagcgg ctgcgcgatc ggcgtccgaa

201721 atcgctgatc tttgaccctg aggaaatcgg gttcgtggtg aaagaaacgg tccccatacc

201781 agcgagcgga gactatcagg atctcccctt gtggagggga cttacgatcg ctgcggtcag

201841 cgagattcgc aggaattact cgcaggacat catcatccca atgacgctcg tgcacccgga

201901 ctatctgact gagatactcg acgggttaag gcagatcgac gatcagctgc tgcacatctt

201961 tctgatgctt aacgaggacc tattgcgtca ccggatctcg aaccagacca tgcatcctga

202021 cccgaatcgg aatgcggaga ttcgagagtg gcgattagcg aatgtcgccc gatgcttggc

202081 cgcaagggaa cggcttccgt ccacaacccg tgttctcgat agtggtgcac acaccagcga

202141 tgaacttgca gcgatggtgc tcgacagact cgatcagcgc acctgatcgc cttagagctg

202201 gctcgggaaa tctgcacagc ggggataagt ggagtttctg cctgacttcg gcttagatgc

202261 cgggaacagg agagaccatg acgagacgac cgcgccggaa ccatagcccg gctttcaagg

202321 cgaaggtggc acttgccgcc atccgaggtg agcagacgct ggtggagttg tcccagcagt

202381 tcgatgtgca cgccaaccag atcaagcaat ggaaagacca gctccttgag ggggcgacag

202441 gtgtgttcgg cgatgaaacg aaagcggagc cgtcgggtcc gaccatcgat gtcaaaacgc

202501 tgcacgcgaa aatcggcgaa ctgacactgg agaacgattt tttatccggt gcgctcggca

202561 aggcgggatt gctgggcgga aagaaatgat cgaccgcgag cacaagctat ccgtcgtgcg

202621 ccaggcgaag cttctcggct tcagccgtgg cagcgtctac tatctgcctc gtccggtgtc

202681 tgacggcgat ctggccctta tgcgccggat tgacgaattg catctcgact acccctttgc

202741 cggaagtcgg atgttgcaag ggctcttgag aggagaaggt ctggagaccg ggcggcgaca

202801 cgtcgccacg ctgatgaaga agatgggcat caaggcgatc taccgccgcc cgaacacctc

202861 gaaaccagcg ccagggcaca tacgaaagct ggcagtcacc cggcccaatc aggtgtgggc

202921 aatggacctg acctacatcc ccatggcgcg gggatttgtc tatctgtgcg ccgtcgtgga

202981 ctggttcagc cggaaggtct tgtcatggcg gttgtcgatc acgatggaag cagccttctg

203041 catcgaggcg gtggaggagg cacttgcccg tcatggcagg ccggaaatct tcaataccga

203101 ccagggatcg cagttcacct ccatcgactt caccgccgtg ctgaagaggt cacagattgc

203161 catctcgatg gatggcaagg gtgcgtggcg agacaatgtc ttcgtcgagc ggctctggcg

203221 ttcgatcaaa tacgaggaag tctacctcca tgcctacaag actgtgtccg aggcacgcgc

203281 tggcatcgcc cgatatctga acttctacaa caccagacgc ccacattggc actgttgcaa

203341 agttagcgat gaggcagcct tttgtcttat tcaaaggcct tacatttcaa aaactctgct

203401 taccaggcgc atttcgccca ggggatcacc ataataaaat gctgaggcct ggcctttgcg

203461 tagtgcacgc atcacctcaa tacctttgat ggtggcgtaa gccgtcttca tggatttaaa

203521 tcccagcgtg gcgccgatta tccgtttcag tttgccatga tcgcattcaa tcacgttgtt

203581 ccggtactta atctgtcggt gttcaacgtc agacgggcac cggccttcgc gtttgagcag

203641 agcaagcgcg cgaccatagg cgggcgcttt atccgtgttg atgaatcgcg ggatctgcca

203701 cttcttcacg ttgttgagga ttttacccag aaaccggtat gcagctttgc tgttacgacg

203761 ggaggagaga taaaaatcga cagtgcggcc ccggctgtcg acggcccggt acagatacgc

203821 ccagcggcca ttgaccttca cgtaggtttc atccatgtgc cacgggcaaa gatcggaagg

203881 gttacgccag taccagcgca gccgtttttc catttcaggc gcataacgct gaacccagcg

203941 gtaaatcgtg gagtgatcga cattcactcc gcgttcagcc agcatctcct gcagctcacg

204001 gtaactgatg ccgtatttgc agtaccagcg tacggcccac agaatgatgt cacgctgaaa

204061 atgccggcct ttgaatgggt tcatgtgcag ctccatcagc aaaaggggat gataagttta

204121 tcaccaccga ctatttgcaa cagtgcccta tttttcgggg atctgattgc cctctggcaa

204181 tatcattcag cacgccatag tcggcatcat ggtccattcg ccagaaaacc gaagcaccgg

204241 catcagccag gcgtggagaa aactggtatc ccagcagcca gaaaaggcca aagacaagtt

204301 cgctggcacc tgctgtatcg gtcataattt cggttggatt cagcccggtc tcctgttcca

204361 gaagaccttc cagcacaaag atagagtccc tcagcgtccc cggtataacg atgccatgaa

204421 agccggaata ctgatcggac acaaagttgt accaggtgat ccctctgtta ttaccaaagt

204481 atttgcggtt cggtccggca ttgattgttc tgactggcgt aacaaagcgc attccatctg

204541 cagatgccac ttctcctcca ccccatatct gtgccagtgg cagcgttgcc tgaaaatcaa

204601 ccagtctggc attagcgctg gtgatagttt cagcccgcag atagttcgct tttgtccagt

204661 tcagccggtg tcgggtcagt gcaggaacat ttgatctgat cagtggttcc agaccgatat

204721 tgcaggcttc agccatcagc acggcgctga tgctgacggg cagatcatca actctggcac

204781 tggcttcact agcatggaaa aactcatcag caaatccggt atgggcgtta atttcgagca

204841 gcaactccgt taaatccacc ggagggagta gatcactgat cattttgctc agtcgtttca

204901 gactgtccgg ctcatcaaga ctggcgaggg gagaaattgt caaccggggc ttcgggccag

204961 aaacatcgag ttcgacagcc tcattttcgc aaagacgtgc agcaacctgt ctgtaacgac

205021 tatcaagctg atgacccaga gattttattg cttcctgcgg gtctgtcggg tgccccaaag

205081 aacgataaac cttaatccgg tttgcctgcc agtcagcacc ctgtagtaat cttgcacgag

205141 gatctcccca ccggttactg ccggtaacgt agacatccct ccgcctcaga ctatcctgca

205201 gtttactgag aaagcagagc gtgtatcccc tgcgggtgat atgtttttcc ttgttaatca

205261 ccagccgttt ccatgaccga ctgataattt ccgttggtgc gtcgtcaaaa aactgccgcc

205321 gtgagctgaa ctcccggctg aggtagtcac aggcattcag agtggtaacc ccggcaggtg

205381 cggatgaaaa tttaacggta ttcagcagat ggggcaggaa acgacgaacg cgcccgtact

205441 gctccaccat ttcttcatga aaattatcgt ctgagggccg ggcaatttca cggacaagcg

205501 tgatgatttc agccagcttt tgccttggga tgtagctgaa cacctcagca cgaatcgatt

205561 cgtccggtgt ttcttctttc agcaggtacg aacatgcgct ggcgagcgcc aatgcagatt

205621 tatccagatc cttcagcgag cggagccgtt ttttctgccc aatctttctg gcgtcacgga

205681 tgataacggc cagcatggcg tccagaacgt ccaatgcatc atccagcgcc agcgtttccc

205741 atgcaaggac aaaggcaacc agaaccgcca tccttttctg cggtgacatc ctggcaatat

205801 tgaacaccga agtcatacca gcataacgtg cgagattttt caggcgcaca gccgggagtg

205861 tactcaggtt ttcagcatgc aggccaaaat cgttcagagt tttccagcgt tcaattgctt

205921 cattaaacgc cggaccactg atggtcacag ggcccttttt cagtgattcc agtaaagaca

205981 ggcggctgca atcagttggc cccagcagca tctccagctg tgaacgctgt tcggctgacg

206041 gtatcagtgc cagtttgttc cacaggcgca acgtcgcctt ttcccttacc tctgaaatca

206101 accgggtcag cgtagtggct ccggggagaa taatacgatg ttgcataagc caccctgtcg

206161 ccagatcgaa aagcaggcca ggacgttcgt tgcttatcca gctccgggta tataaaagac

206221 gggtaaggcg aaatgtccag ggccaggcaa attcacgata ctgatagtgc tgacgtatca

206281 gcgctgcatg ctcacggcgg gtattttccc tctgaccgta ttctgcaaga acggtgatat

206341 cacgaatccc gagctgtctg gcggtaaaat gccggacgcc ggaaggaata tgacctggga

206401 cctacgtgcg cccgcaccga caccctcaca ccttcgagct actgttgcca ttaaggggtc

206461 gtttcgtggt gctgaatttt gacgatcggg gtaccgtcac ccatcgggcg atattggggg

206521 aaacctgtac ggtgctggag atggccgcag gaacctggca tgccgtgctg tcgctggata

206581 ccggtggcat aatttttgaa gtaaaacacg gtggctatca acccgtggct gccgatgact

206641 atgcgcactg ggctccagcg gaaggagaac caggaaccac ggagcttatg gcctggtatg

206701 cgcaagcgca ggtgggcgac agcacttttg ccgtctaagg cgataaacaa aaacggaatg

206761 agtttcccca ttccgtttcc gctattacaa accgtcggtg acgattttag ccgccgacgc

206821 taatacatcg cgacggcttt ctgccttagg ttgaggctgg gtgaagtaag tgaccagaat

206881 cagcggcgca cgatcttttg gccagatcac cgcgatatcg ttggtggtgc catagccacc

206941 gctgccggtt ttatccccca caacccagga agcaggcagt ccagcctgaa tgctcgctgc

207001 accggtggta ttgcctttca tccatgtcac cagctgcgcc cgttggctgt cgcccaatgc

207061 tttacccagc gtcagattcc gcagagtttg cgccattgcc cgaggtgaag tggtatcacg

207121 cggatcgccc ggaatggcgg tgtttaacgt cggctcggta cggtcgagac ggaacgtttc

207181 gtctcccagc tgtcgggcga acgcggtgac gctagccggg ccgccaacgt gagcaatcag

207241 cttattcatc gccacgttat cgctgtactg tagcgcggcc gcgctaagct cagccagtga

207301 catcgtccca ttgacgtgct tttccgcaat cggattatag ttaacaaggt cagatttttt

207361 gatctcaact cgctgattta acagattcgg ttcgctttca cttttcttca gcaccgcggc

207421 cgcggccatc actttactgg tgctgcacat cgcaaagcgc tcatcagcac gataaagtat

207481 ttgcgaatta tctgctgtgt taatcaatgc cacacccagt ctgcctcccg actgccgctc

207541 taattcggca agtttttgct gtacgtccgc cgtttgcgca tacagcggca cacttcctaa

207601 caacagcgtg acggttgccg tcgccatcag cgtgaactgg cgcagtgatt ttttaaccat

207661 gggattcctt attctggaag atacgaaata acaacaacat gaatagtccc taaattccac

207721 gtgtgttttt tattagcttc aaaaatcact atttcacgaa gaatttagac tgcttctcac

207781 acattgtaac attatttaca accacctttc aatcattttt gataaatcat tgatttcatc

207841 tttgctgcaa tgatacttaa taaactctgc aagttatcca cagagcaaca ctcaatttta

207901 ttgatgatat tcttattata ccagacattt ttcatacact cccttgtacg gatagttttc

207961 cgacaacttc atgattacat atcttgcggt tttgattatt tttgctgcaa gaaatacata

208021 cttcaaacga aaggtcttta tttgctgtct gtattctgaa gagtccaagg aatcaaactt

208081 gaacaacaaa aataggttat atgaaagcat catcatttga aacacggctt cattcgccca

208141 aaatgacttt agcaagagat gacccaccgc catgtcgtat ttggcttctt tgatatagtt

208201 ttcagcatta ccacgctttt catagtatat aactactttt tcagaaagca aggtagtatt

208261 tgttacaaag aaaaagtagt cgtattcgga accttctaaa agtgataatt gtgctctttc

208321 tttttctggt ttcagtacgc gagatacgac aaatcttctg tctttttccc atttaactaa

208381 ttttgtatac agttctgtag tttctctacc ttcttctcct ttaacgaata caattgatga

208441 attcgttgct tgtgaggtga gtgtagaata acttttggct ttaattaaat atttgcatcc

208501 aagagattct atcgtttcga taattttttc atcaaagtag ccactatcca ttcgaaataa

208561 aatttctaaa tcgtctgatt tgatgttagc aacaatttct ttgatcattt ccgcagcacc

208621 gtttgcagtg taagtattgc cacttcttac aaatccggta acatatgctt ttaattcgtc

208681 gcaaaatgca aattggatat tgtagcatcg gtttcccagt ttcttaggat tatatccttt

208741 tgacgcacct tcttgatgac cttctacgtt aattacacta ctatcaatat caatcgtaat

208801 ggatgtcaat ttacttttag tgagcagttt tttaaagact ttaaaattaa tgtctctaaa

208861 catttgggtt gtcttgaagt tgaagtttcc tagaaaccgt gacactgttt caggttcttt

208921 tacggaaata tcaaactcgt tgacgagggg atcattttga agtagcttta gacgttctaa

208981 cttatcaatg ccaatgaagt gaccgcagag catggtcttt atatgattca tcttgatttt

209041 atttgttgag tcattatcaa atacgaggtc attttcaata aaatcaaaaa tcccattgct

209101 ttttgcattc tcaaggagca gaaaaagacc tgcatttgat gttagattct tagctttgaa

209161 atcaatttta ttaatcataa ttagaacccc tttttactac ttttcttact attattttac

209221 catatatcga gtcataaaag ctgataattt aacatatttt tgagcacttt tctttcaccc

209281 aatgggtgaa agctgaattt cgaaggaatg catatttatc aaggctttga ttatgctttt

209341 tgaagtactg acgtagaatc taggtatgat tcatatcggt gaggaaggtg cccagaaaac

209401 ggacacatcc aatttgcagg gcaatgccca gacggttgtg atcacctctg ctttttccga

209461 taaattcctt gtctgcttca tcaaggtgaa aatatcgtgc cagctgaagc tcatccggtt

209521 caccggtgaa tctgccatag ctttcagtct gctcagtggt cagaaagtca acgggcatat

209581 cggcctccct gcctgacggg catttagtaa catttttcca accgtacgaa atgttataaa

209641 ttatcggaca tcgtaaaact gttacattaa tatgtctatt aaatcgtaaa tttgtaataa

209701 tagacatgag ttgtccgata ttcgatttaa ggtacatttt tatgcgactt tttggttacg

209761 ctcgggtctc aaccagtcag cagtctcttg atcttcaggt cagagcactc aaagacgcag

209821 gtgtgaaagc aaaccgtata tttaccgata aggcatccgg cagttcaaca gaccgggaag

209881 ggctggattt gctgaggatg aaggtggagg aaggtgatgt cattctggtt aagaagctcg

209941 accgtcttgg ccgcgacact gccgatatga tccaactgat aaaggaattt gacgctcagg

210001 gcgtggcagt ccggttcatt gatgacggga tcagtaccga cggtgatatg gggcaaatgg

210061 tggtcaccat cctgtcggct gtggcacagg ctgaacgccg gaggatccta gaacgcacga

210121 atgagggccg acaggaagca aagctgaaag gaatcaaatt tggccgcagg cgtaccgtgg

210181 acaggaacgt cgtgctgacg cttcatcaga agggcactgg tgcaacggaa attgctcatc

210241 agctcagtat tgcccgctcc acggtttata aaattcttga agacgaaagg gcctcgtgat

210301 acgcctattt ttataggtta atgtcatgat aataatggtt tcttagacgt caggtggcac

210361 ttttcgggga aatgtgcgcg gaacccctat ttgtttattt ttctaaatac attcaaatat

210421 gtatccgctc atgagacaat aaccctggta aatgcttcaa taatattgaa aaaggaagag

210481 tatgagtatt caacattttc gtgtcgccct tattcccttt tttgcggcat tttgccttcc

210541 tgtttttgct cacccagaaa cgctggtgaa agtaaaagat gctgaagatc agttgggtgc

210601 acgagtgggt tacatcgaac tggatctcaa cagcggtaag atccttgaga gttttcgccc

210661 cgaagaacgt tttccaatga tgagcacttt taaagttctg ctatgtggtg cggtattatc

210721 ccgtgttgac gccgggcaag agcaactcgg tcgccgcata cactattctc agaatgactt

210781 ggttgagtac tcaccagtca cagaaaagca tcttacggat ggcatgacag taagagaatt

210841 atgcagtgct gccataacca tgagtgataa cactgctgcc aacttacttc tgacaacgat

210901 cggaggaccg aaggagctaa ccgctttttt gcacaacatg ggggatcatg taactcgcct

210961 tgatcgttgg gaaccggagc tgaatgaagc cataccaaac gacgagcgtg acaccacgat

211021 gcctgcagca atggcaacaa cgttgcgcaa actattaact ggcgaactac ttactctagc

211081 ttcccggcaa caattaatag actggatgga ggcggataaa gttgcaggac cacttctgcg

211141 ctcggccctt ccggctggct ggtttattgc tgataaatct ggagccggtg agcgtgggtc

211201 tcgcggtatc attgcagcac tggggccaga tggtaagccc tcccgtatcg tagttatcta

211261 cacgacgggg agtcaggcaa ctatggatga acgaaataga cagatcgctg agataggtgc

211321 ctcactgatt aagcattggt aactgtcaga ccaagtttac tcatatatac tttagattga

211381 tttaaaactt catttttaat ttaaaaggat ctaggtgaag atcctttttg ataatctcat

211441 gaccaaaatc ccttaacgtg agttttcgtt ccactgagcg tcagacccct aaaagaactt

211501 tccgctaagc gatagactgt atgtaaacac agtattgcaa ggacgcggaa catgcctcat

211561 gtggcggcca ggacggccag ccgggatcgg gatactggtc gttaccagag ccaccgaccc

211621 gagcaaaccc ttctctatca gatcgttgac gagtattacc cggcattcgc tgcgcttatg

211681 gcagagcagg gaaaggaatt gccgggctat gtgcaacggg aatttgaaga atttctccaa

211741 tgcgggcggc tggagcatgg ctttctacgg gttcgctgcg agtcttgcca cgccgagcac

211801 ctggtcgctt tcagctgtaa gcgtcgcggt ttctgcccga gctgtggggc gcggcggatg

211861 gccgaaagtg ccgccttgct ggttgatgaa gtactgcctg aacaacccat gcgtcagtgg

211921 gtgttgagct tcccgtttca gctgcgtttc ctgtttgggg tcgtttgcgg gaaggggcgg

211981 aatcctacgc taaggctttg gccagcgata ttctccggtg agattgatgt gttcccaggg

212041 gataggagaa gtcgcttgat atctagtatg acgtctgtcg cacctgcttg atcgcggccg

212101 cgatagctag atcgcgttgc tcctcttctc catccgcgtt ccaagctgcg gaaaggcacc

212161 cataagcgta cgcctggtcg agcaggcgac gcggatcgac gtccagcgca cgagagaatg

212221 cgtccgccat ctgtgcaatg cgtctaggat cgagacaaag gtcgtctctg tcagccggat

212281 cgtagaacat attggcggcg ccaaagccca cttcaccgac cagaccgacg ggatctatca

212341 ccagccagcc gcgactggag aacatgatgt tttcatgatg cagatcgcca tgtagcccac

212401 gcagttccga ggcattgctc atcatttgat cggctataat cgccgcgtgg acgtagtcag

212461 tttgacaacc tgcgttttga tcatcgcgcg cccgctgaaa caaagctgca aagcgatccc

212521 ggatcgggag aagggcagaa ggcaggggtt cctcagatgc ggcatacagc ttcgccatta

212581 gttccgctgc aatttcggtc gcctggtagt cgccgtgctc ggcaacgatg tgagagagca

212641 ttcgctcccc ggcatattcg agcaacatca gattgttctc acgaccgagc aaccggactg

212701 ctcccctccc attgcgccat accagatagt cggccccgcg cagttcatca gcaatgtctt

212761 ctataggttt caatcccttg acgattgcag gagtcccgtc tggcaatgaa actttccaaa

212821 cgaggctgga aaaggtgtcc gcaatgagaa caggttgcga aacgtgccaa tgagcaggaa

212881 aaacaggcgg catgaacatc aaccccaagt cagagggtcc aatcgcagat agaaggcaag

212941 gcgttcgcgg tcgggggctt cgatccccaa tacattgaat aggacagcga aggcgcgctc

213001 tgcttcatct ggcgctgccc agttctcttc ggcgttagca atcatgagtg ccaaatcggc

213061 atagcgatct gctgttccga gccgcccaag gtcgatcaga cccgtgcatt gaagagtttt

213121 agggtccacc atgaagttcg gcatgcaggg atcaccatgg caaacaacca tatcggtgcg

213181 ctcttggtcg agccgcaccg gtagctctcg ttcgacacga gccaaaagat cgagctgcgg

213241 cgtactcttg tcctcgtccg gtaagaagtc gggattgacg gcattgcggg acaccacatc

213301 aacggcgcgt ccgaacattc gcgacagcct gcgctcaaac ggacattgat caaccgatag

213361 gctgtgaaca gcgccaagtt gctgccccat tgacggccac gctttgagca aatccgctcc

213421 agacagatca gccgccggta ctcccggaat tgccgttatc accaagcatg caccctcctg

213481 ttcctcctgc cagttgatga cctcggggca agccacacct cgacctttga gccaaatgag

213541 gcggtcacgc tctccagcga gctcaccgcg gcgggaagca ggtgcgattt tcgcgaaggc

213601 atgcccgtca ccacgtcgaa aaacaaaatc accagattct ccgcctctga caggcaacca

213661 gtcagaatgc gattcaccaa aaaaaatatt agttcgattc aatggaggtt ccttcagttt

213721 tctgatgaag cgcgaatata gagaaatatc ccgaatgtgc agttaacgaa ttcttgcggt
[truncated: 254,296 more chars]
