# Supplementary figures and images for: Coexistence of blaOXA-48 and Truncated blaNDM-1 on Different Plasmids in a Klebsiella pneumoniae Isolate in China
Source: Front Microbiol. 2017 Feb 2;8:133. doi: 10.3389/fmicb.2017.00133 (PMC5288367; doi:10.3389/fmicb.2017.00133)

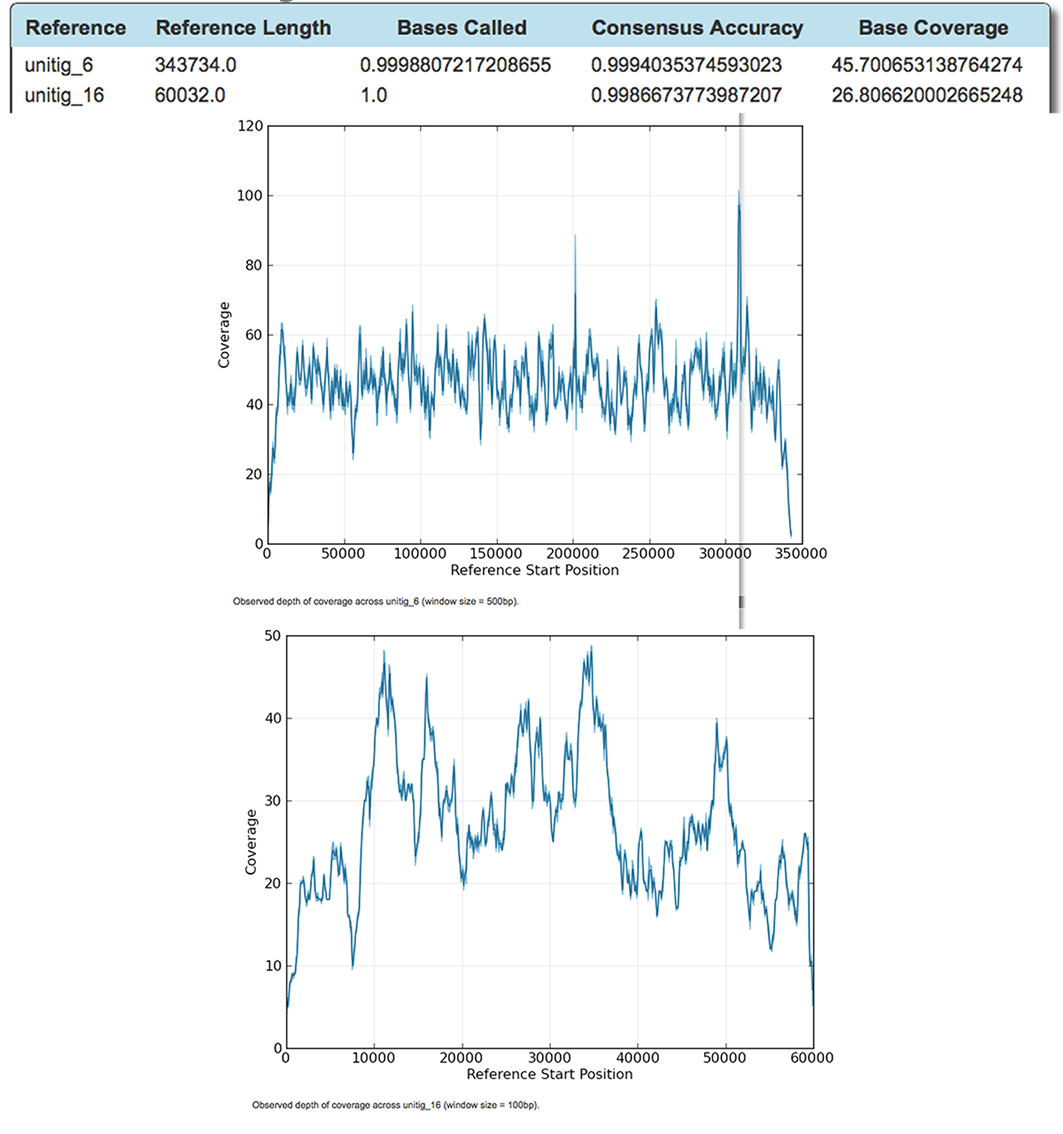

Supplement: Supplementary file 2 [file Image_1.tif]

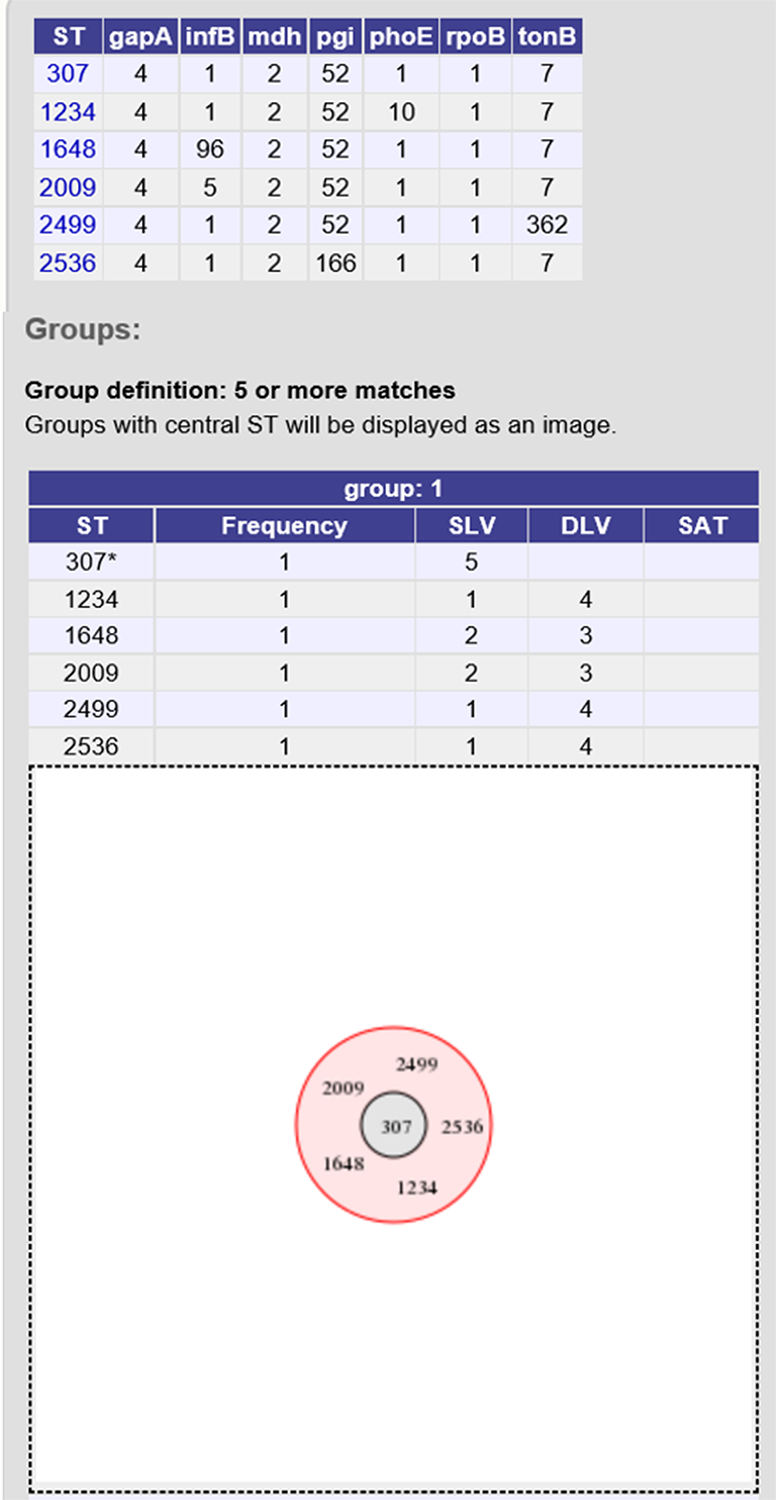

Supplement: Supplementary file 3 [file Image_2.tif]
